# D-Amino Acid-Containing Lipopeptides Derived from the Lead Peptide BP100 with Activity against Plant Pathogens

**DOI:** 10.3390/ijms22126631

**Published:** 2021-06-21

**Authors:** Àngel Oliveras, Luís Moll, Gerard Riesco-Llach, Arnau Tolosa-Canudas, Sergio Gil-Caballero, Esther Badosa, Anna Bonaterra, Emilio Montesinos, Marta Planas, Lidia Feliu

**Affiliations:** 1 LIPPSO, Department of Chemistry, Campus Montilivi, University of Girona, 17004 Girona, Spain; a.oliveras.rovira@gmail.com (À.O.); gerard.riesco@udg.edu (G.R.-L.); arnauatc_11@hotmail.com (A.T.-C.); 2 Laboratory of Plant Pathology, Institute of Food and Agricultural Technology-CIDSAV-XaRTA, Campus Montilivi, University of Girona, 17004 Girona, Spain; lluismds@hotmail.com (L.M.); esther.badosa@udg.edu (E.B.); anna.bonaterra@udg.edu (A.B.); emilio.montesinos@udg.edu (E.M.); 3 Serveis Tècnics de Recerca (NMR), Universitat de Girona, Parc Científic i Tecnològic de la UdG, Pic de Peguera 15, 17004 Girona, Spain; sergio.gil@udg.edu

**Keywords:** acylation, hemolysis, secondary structure, NMR

## Abstract

From a previous collection of lipopeptides derived from **BP100**, we selected 18 sequences in order to improve their biological profile. In particular, analogues containing a D-amino acid at position 4 were designed, prepared, and tested against plant pathogenic bacteria and fungi. The biological activity of these sequences was compared with that of the corresponding parent lipopeptides with all L-amino acids. In addition, the influence of the length of the hydrophobic chain on the biological activity was evaluated. Interestingly, the incorporation of a D-amino acid into lipopeptides bearing a butanoyl or a hexanoyl chain led to less hemolytic sequences and, in general, that were as active or more active than the corresponding all L-lipopeptides. The best lipopeptides were **BP475** and **BP485**, both incorporating a D-Phe at position 4 and a butanoyl group, with MIC values between 0.8 and 6.2 µM, low hemolysis (0 and 24% at 250 µM, respectively), and low phytotoxicity. Characterization by NMR of the secondary structure of **BP475** revealed that the D-Phe at position 4 disrupts the α-helix and that residues 6 to 10 are able to fold in an α-helix. This secondary structure would be responsible for the high antimicrobial activity and low hemolysis of this lipopeptide.

## 1. Introduction

Agriculture is currently facing major challenges in terms of food production and conservation. It is expected that the world population will rise to more than 10 billion by 2100 according to the United Nations [1]. One of the main threats are plant diseases caused by bacteria and fungi that bring about important economic losses every year [2,3]. A strategy to overcome this problem relies on employing copper compounds, antibiotics, and fungicides. Even though these compounds are efficient, they are regarded as serious environmental contaminants and their use is restricted by the current regulations. For instance, antibiotics are banned in Europe because they prompt the appearance of resistant strains. Therefore, the development of safer compounds to fight these diseases is of paramount importance.

Antimicrobial peptides have received much attention as alternative pesticides [4,5,6,7]. They display a broad spectrum of activity and their mechanism of action generally involves the perturbation of the cell membrane which limits the induction of resistance [8,9,10,11,12,13,14]. Despite these excellent properties, research has been conducted to design new antimicrobial peptides with improved biological activity profiles [15,16,17,18]. Towards this end, a large number of synthetic lipopeptides have been described [19], mainly prompted by the presence of a fatty acid chain in many natural active peptides and by the essential role of this chain for their antimicrobial activity [20,21]. In fact, acylation is recognized as an effective peptide modification to increase antimicrobial activity and proteolytic stability [22,23,24,25,26,27,28,29,30]. The fatty acid chain confers lipophilicity, enhances the peptide ability to either adopt a specific secondary structure or oligomerize upon interacting with the bacterial membranes, and, therefore, facilitates the hydrophobic interaction between peptides and membranes [30].

One important limitation associated with antimicrobial lipopeptides that precludes their use is their low cell selectivity, probably due to their hydrophobicity, which results in high hemolytic activity [31]. A strategy to overcome this drawback is the incorporation of D-amino acids. This approach has been applied to antimicrobial peptides providing sequences that are not only less hemolytic, but also similarly active and more stable than their counterparts with all L-amino acids [32,33,34,35,36,37,38,39,40,41]. In previous studies on cyclic lipopeptides, we observed this trend when the residue bearing the fatty chain was replaced with its D-enantiomer [26]. The resulting cyclic lipopeptides with a D-amino acid were as active as their L-counterparts and, interestingly, they were not hemolytic at concentrations 10- to 80-fold higher than their MIC values. Other studies have also shown that the combination of D-amino acids and a lipidic chain in a peptide constitutes an effective strategy to obtain sequences with improved biological activity [29,42,43,44,45].

In this context, we recently described a library of 36 lipopeptides derived from the linear antimicrobial undecapeptide H-Lys-Lys-Leu-Phe-Lys-Lys-Ile-Leu-Lys-Tyr-Leu-NH_2_ (**BP100**) [46]. These lipopeptides were designed by incorporating a butanoyl, hexanoyl or lauroyl chain at the N-terminus or at the side chain of a Lys residue. Lipopeptides with high antimicrobial activity and different degrees of hemolysis and phytotoxicity were identified. Taking into account the advantages of incorporating D-amino acids into the structure of lipopeptides, in the present work, we decided to evaluate the improvement of the biological profile of 18 selected lipopeptides through the replacement of one amino acid with its D-enantiomer. Thus, we prepared these 18 D-amino acid-containing lipopeptides and tested their in vitro antimicrobial activity against six plant pathogenic bacteria and two plant pathogenic fungi as well as their hemolysis and phytotoxicity. Moreover, the secondary structure of one of the best D-amino acid-containing lipopeptides was characterized by NMR spectroscopy.

## 2. Results

### 2.1. Design and Solid-Phase Synthesis of the Lipopeptides

Taking into account the advantages of incorporating a D-amino acid into a peptide sequence, we selected a set of 18 lipopeptides derived from **BP100**, previously reported by our group [46], and replaced the amino acid at position 4 for the corresponding enantiomer. This position was chosen because in previous studies we had observed that the substitution of L-Phe^4^ in **BP100** (H-Lys-Lys-Leu-Phe-Lys-Lys-Ile-Leu-Lys-Tyr-Leu-NH_2_) with a D-Phe resulted in peptide **BP143** (H-Lys-Lys-Leu-D-Phe-Lys-Lys-Ile-Leu-Lys-Tyr-Leu-NH_2_), which was more active and less hemolytic [32]. The selected lipopeptides displayed high activity but most of them were also highly hemolytic. Thus, the aim of this study was to obtain peptides with an improved biological activity profile. The sequence of the 18 lipopeptides bearing a D-amino acid is depicted in Table 1.

These lipopeptides were synthesized on solid phase following a standard 9-fluorenylmethoxycarbonyl (Fmoc)/*tert*-butyl (*t*Bu) strategy as previously described [46]. A Fmoc-Rink-MBHA resin was used as solid support. In the case of lipopeptides incorporating a Lys residue acylated at the side chain, this amino acid was incorporated as Fmoc-Lys(ivDde)-OH or Fmoc-D-Lys(ivDde)-OH. After 1-(4,4-dimethyl-2,6-dioxocyclohex-1-ylidine)-3-methylbutyl (ivDde) group removal, the *N*^Ɛ^-amino group was derivatized with butanoic, hexanoic or lauric acid. For the synthesis of **BP472** and **BP485** the N-terminus amino group was acylated with hexanoic and butanoic acid, respectively. Lipopeptides were cleaved from the support using trifluoroacetic acid (TFA)/H_2_O/triisopropylsilane (TIS) and purified by reverse-phase column chromatography (HPLC). They were obtained in >99% HPLC purity and their structure was verified by mass spectrometry (Table 1).

### 2.2. Antimicrobial Activity

**Figure 1:**
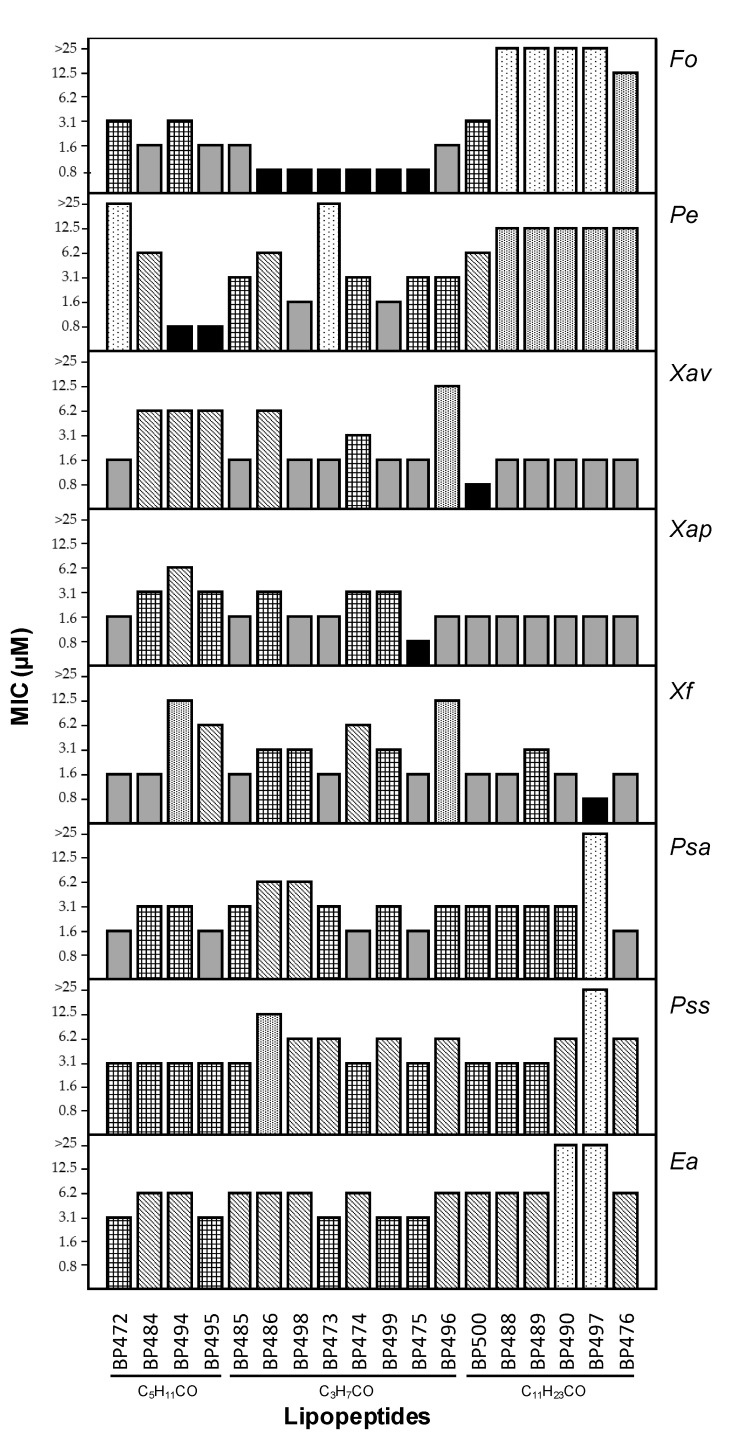
Antimicrobial activity of lipopeptides against the bacteria *E. amylovora* (*Ea*), *P. syringae* pv. syringae (*Pss*), *P. syringae* pv. actinidiae (*Psa*), *X. fragariae* (*Xf*), *X. arboricola* pv. pruni (*Xap*) and *X. axonopodis* pv. vesicatoria (*Xav*), and the fungi *P. expansum* (*Pe*) and *F. oxysporum* (*Fo*). The type of acyl group is indicated below the lipopeptides. Antimicrobial activity is given as the minimal concentration that inhibits growth (MIC). The MIC axis is in logarithmic scale, and for each sequence, the lowest values of the MIC range is represented. Data can be found in Table S1 (Supplementary Materials).

Lipopeptides were screened for in vitro growth inhibition of the plant pathogenic bacteria *Erwinia amylovora*, *Pseudomonas syringae* pv. syringae, *Pseudomonas syringae* pv. actinidiae, *Xanthomonas arboricola* pv. pruni, *Xanthomonas fragariae* and *Xanthomonas axonopodis* pv. vesicatoria, and the plant pathogenic fungi *Penicillium expansum* and *Fusarium oxysporum,* at 0.8, 1.6, 3.1, 6.2, 12.5 and 25 µM (Figure 1, Table S1).

This set of 18 lipopeptides showed high antimicrobial activity (Figure 1). Concerning the antibacterial activity, 13 sequences exhibited MIC < 12.5 µM against the six bacteria tested. The results showed that they were more active against the three *Xanthomonas* strains than against *E. amylovora* or the *Pseudomonas* species. Twelve lipopeptides displayed MIC < 6.2 µM against the three *Xanthomonas* strains, among which nine showed MIC < 3.1 µM. Remarkably, an MIC between 0.8 and 1.6 µM against one of these strains was observed for three sequences.

Regarding the *Pseudomonas* species, they displayed higher activity against *P. syringae* pv. actinidiae (15 sequences with MIC < 6.2 µM) than against *P. syringae* pv. syringae (10 sequences with MIC < 6.2 µM). Interestingly, five lipopeptides showed MIC between 1.6 and 3.1 µM against *P. syringae* pv. actinidiae. *E. amylovora* was the least sensitive bacterium towards these lipopeptides. However, 16 sequences exhibited MIC < 12.5 µM with five of them showing MIC values between 3.1 and 6.2 µM. Regarding the influence of the fatty acid chain, no correlation was observed between the antibacterial activity and the length of this chain. However, those incorporating a lauroyl group were more active against the *Xanthomonas* strains. The lipopeptides with the highest antibacterial activity were **BP472** (C_5_H_11_CO-D-F^4^), **BP473** (D-F^4^-K^6^(COC_3_H_7_)), **BP475** (D-F^4^-K^10^(COC_3_H_7_)), **BP476** (D-F^4^-K^11^(COC_11_H_23_)), **BP485** (C_3_H_7_CO-D-F^4^), **BP488** (D-K^4^(COC_11_H_23_)), and **BP500** (D-F^4^-K^3^(COC_11_H_23_)).

In the case of the antifungal activity, the lipopeptides were, in general, more active against *F. oxysporum* than against *P. expansum* (13 vs. 8 sequences with MIC < 6.2 µM, respectively) (Figure 1). Interestingly, MIC values between 0.8 and 1.6 µM were observed for six and two sequences, respectively, which incorporate a butanoyl or a hexanoyl group. In particular, **BP495** (D-F^4^-K^11^(COC_5_H_11_)), **BP498** (D-F^4^-K^5^(COC_3_H_7_)) and **BP499** (D-F^4^-K^9^(COC_3_H_7_)) were the most active, with MIC between 0.8 and 3.1 µM against both fungi.

### 2.3. Toxicity

The toxicity of lipopeptides to eukaryotic cells was determined as the ability to lyse erythrocytes in comparison to melittin, which was used as a reference peptide (Table 2 and Table S2). Lipopeptides were assayed at 50, 150, 250 and 375 µM. The results showed that 10 lipopeptides displayed ≤ 24% hemolysis at 250 µM. Among them, six sequences exhibited ≤ 10% hemolysis at this concentration, which incorporate either a butanoyl or a hexanoyl chain.

Lipopeptides were also assayed for their toxicity in tobacco leaves by infiltrating a solution of each peptide at 50, 150 and 250 µM into the mesophylls of the leaves (Table 2 and Table S3). For comparison purposes, melittin was also included in this experiment, causing a necrosis of 18 mm at 250 µM. Most lipopeptides were less toxic than melittin. In particular, 11 sequences caused a necrotic area ≤ 10 mm at 250 µM.

Interestingly, lipopeptides **BP475** (D-F^4^-K^10^(COC_3_H_7_)) and **BP485** (C_3_H_7_CO-D-F^4^), which displayed the highest antibacterial activity, and lipopeptides **BP495** (D-F^4^-K^11^(COC_5_H_11_)), **BP498** (D-F^4^-K^5^(COC_3_H_7_)) and **BP499** (D-F^4^-K^9^(COC_3_H_7_)) exhibiting high antifungal activity were also low toxic at concentrations around the MIC.

### 2.4. Structural Characterization by NMR Spectroscopy

The structure of lipopeptide **BP475** (D-F^4^-K^10^(COC_3_H_7_)), which displayed high antimicrobial activity, was characterized by ^1^H, ^1^H-^13^C and ^1^H-^15^N-NMR. In order to evaluate the influence of incorporating the D-amino acid, its analog with all L-amino acids Ac-KKLFKKILKK(COC_3_H_7_)L-NH_2_ (**BP389**) was included in this study.

**Figure 2:**
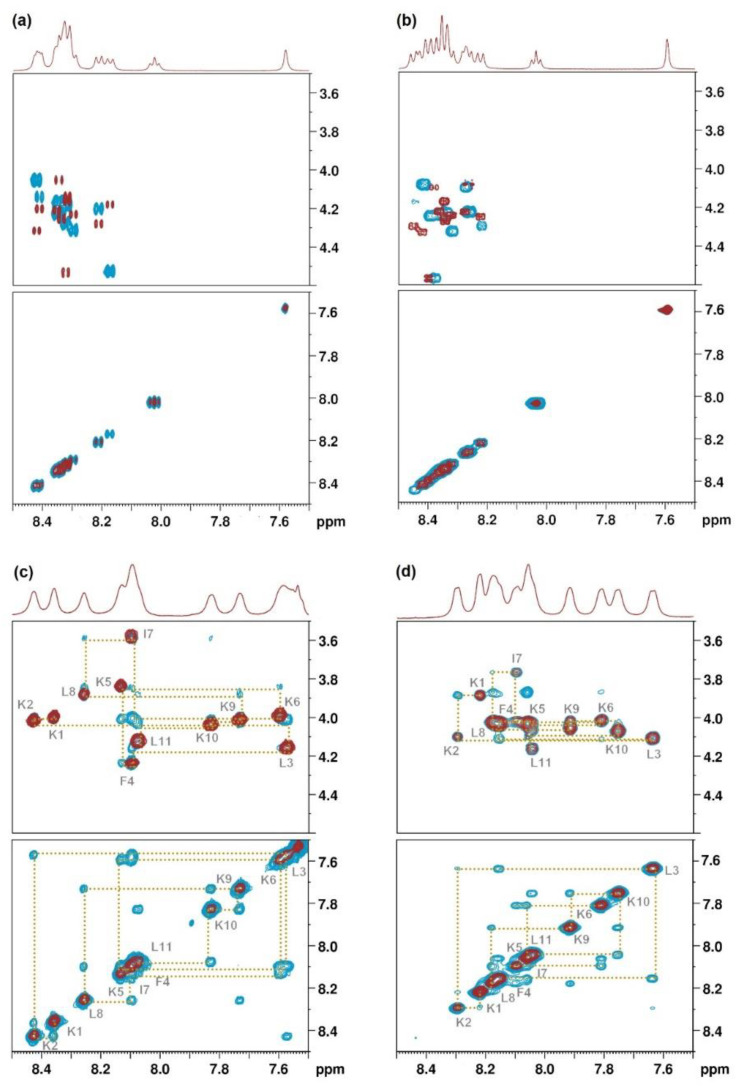
Overlay of HN-HN and HN-Hα TOCSY (red) and NOESY (blue) correlations for (**a**) **BP389** and (**b**) **BP475** in phosphate buffer; (**c**) **BP389** and (**d**) **BP475** in phosphate buffer with 30% CF_3_CD_2_OD.

1D ^1^H-NMR and 2D ^1^H-^1^H TOCSY, 2D ^1^H-^13^C HSQC, 2D ^1^H-^15^N HSQC and ^1^H-^1^H NOESY spectra were first recorded at 10 ºC in phosphate buffer at pH = 6.5 in H_2_O/D_2_O (9:1). These experiments allowed the assignment of the ^1^H, ^13^C and ^15^N signals (Tables S4–S7). 1D ^1^H and 2D ^1^H-^1^H NOESY experiments revealed that these two lipopeptides are completely unstructured in these conditions (Figure 2).

Next, the above experiments were conducted in the presence of 30% CF_3_CD_2_OD [47]. After the assignment of all the ^1^H, ^13^C and ^15^N signals, the primary structure of the peptides was confirmed based on the NOE correlation in the HN-Hα fingerprint region (Figure 2). Compared to the spectra recorded in H_2_O/D_2_O (9:1), in this case, all the HN-Hα cross-peaks could be unambiguously assigned and the analysis of the NOESY showed sequential correlations between amide protons. In addition, the “sequential walk” was achieved with the combination of NOESY Hα_(i)_-HN_(i+1)_ inter-residue correlations and TOCSY Hα_(i)_-HN_(i)_ intra-residue correlations. All these results pointed out that these lipopeptides adopt a secondary structure in the presence of CF_3_CD_2_OD.

**Figure 3:**
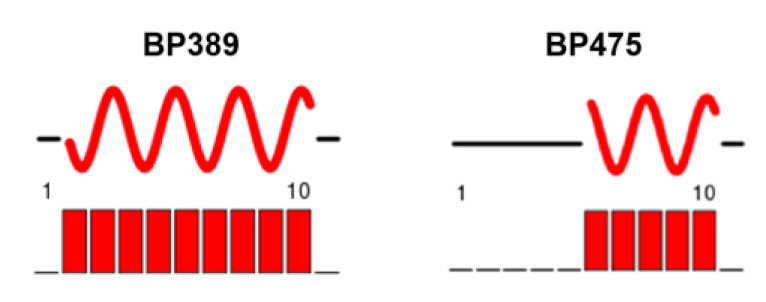
Secondary structure adopted by each peptide. Numbers indicate the position of the amino acids. The red curve represents an α-helix, while the black line stands for a random coil region.

Subsequent chemical shift index analysis was employed to identify the secondary structure of these lipopeptides (Figure 3). It was observed that while residues 2 to 10 in **BP389** form α-helical structure, in **BP475,** only residues 6 to 10 adopt this conformation. The disruption of the α-helix in **BP475** could be attributed to the presence of a D-Phe at position 4.

## 3. Discussion

Lipopeptides are a subfamily of antimicrobial peptides that have attracted attention due to their biological activity [20,23,24,27]. However, their use has been hampered by the high hemolysis that they generally display. One strategy to address this issue is the incorporation of a D-amino acid in their sequence [32,33,34,35,36,37,39,48]. In fact, natural lipopeptides bearing D-amino acids with an interesting biological activity profile have been reported, such as polymyxins, daptomycin, surfactins, iturins and fengycins [20,24,27]. In addition, synthetic D,L-amino acid-containing lipododecapeptides and ultrashort lipopeptides with high antimicrobial activity have also been described [22,42,49]. Moreover, in a previous study on cyclic lipopeptides, we observed that the replacement of an L-amino acid by its D-enantiomer led to sequences with lower hemolysis and similar antimicrobial activity [26]. Based on these reports, in this work, we describe 18 lipopeptides derived from the lead peptide **BP100** containing a D-amino acid with activity against plant pathogenic bacteria and fungi.

These 18 D-amino acid-containing lipopeptides displayed high antimicrobial activity against the pathogens tested (13 sequences with MIC < 12.5 µM against at least six pathogens). In general, the highest activity was observed against *Xanthomonas* strains and *F. oxysporum*. The length of the hydrophobic chain influenced the antimicrobial activity. Whereas lipopeptides bearing a butanoyl or a hexanoyl group were active against all bacteria and fungi, those incorporating a lauroyl group displayed high activity mainly against *Xanthomonas* species. These results and those obtained for lipopeptides with all L-amino acids [46] differed from the general trend described for the antimicrobial activity of lipopeptides [19,22,23,42,49,50,51,52]. The presence of a long acyl chain is, in general, related to high antimicrobial activity. In the present work, lipopeptides containing a lauroyl group were poorly active against fungi and, in contrast, an acyl chain of four and six carbons endowed these compounds with activity.

The length of the fatty acid also influenced the cytotoxicity against red blood cells. Lipopeptides incorporating a butanoyl or a hexanoyl group were, in general, low hemolytic, those with a 12-carbon atom lauroyl group being the ones with the highest hemolysis. The presence of a long fatty acid chain has been associated with a high cytotoxicity due to an increase of peptide hydrophobicity, which in turn results in a high erythrocyte membrane affinity [26,53,54]. In contrast, in the case of the effect of lipopeptides on the size of the lesion in infiltrated tobacco leaves, no correlation between the length of the hydrophobic chain and this effect was observed. All lipopeptides were less phytotoxic than melittin at 250 µM, concentration generally between 20 and 156-fold higher than the MIC. Similar results have been described for other lipopeptides, such as cyclolipopeptides and ultrashort cationic lipopeptides [26,55].

**Figure 4:**
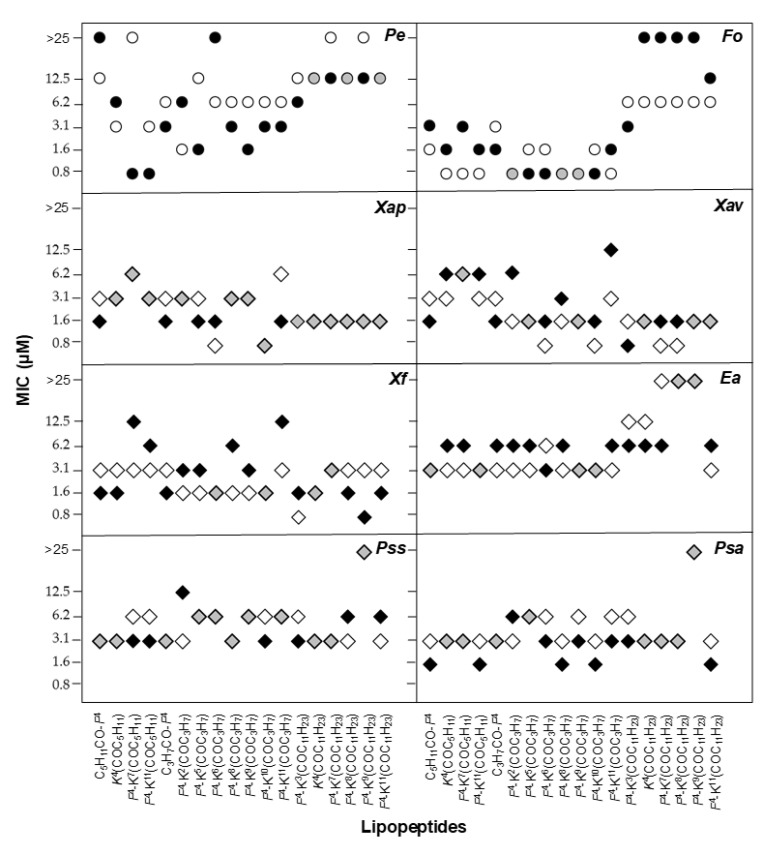
Antimicrobial activity of lipopeptides incorporating all L-amino acids or a D-amino acid against *E. amylovora* (*Ea*), *P. syringae* pv. syringae (*Pss*), *P. syringae* pv. actinidiae (*Psa*), *X. fragariae* (*Xf*), *X. arboricola* pv. pruni (*Xap*) and *X. axonopodis* pv. vesicatoria (*Xav*), and the fungi *P. expansum* (*Pe*) and *F. oxysporum* (*Fo*). The x axis includes the code for each lipopeptide. The residue that can be a L- or a D-amino acid is indicated in italics. Antimicrobial activity is given as the minimal concentration that inhibits growth (MIC). The MIC axis is in logarithmic scale and for each sequence the lowest values of the MIC range is represented. Black symbols correspond to the activity of lipopeptides with a D-amino acid, white symbols to the activity of lipopeptides with all L-amino acids, and grey symbols indicate that both lipopeptides display the same activity. Data can be found in Table S1 (Supplementary Materials).

**Figure 5:**
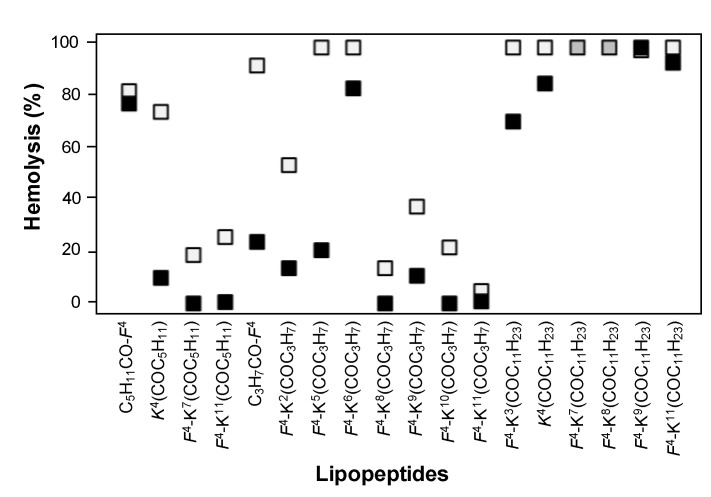
Hemolytic activity of the lipopeptides incorporating all L-amino acids or a D-amino acid. The x axis includes the code for each lipopeptide. The residue that can be an L- or a D-amino acid is indicated in italics. Hemolytic activity was measured at 250 µM and is expressed as a percentage compared to melittin as a standard. Black squares correspond to the hemolysis of lipopeptides with a D-amino acid, white squares to the hemolysis of lipopeptides with all L-amino acids, and grey squares indicate that both lipopeptides display the same hemolysis. Data can be found in Table S2 (Supplementary Materials).

**Figure 6:**
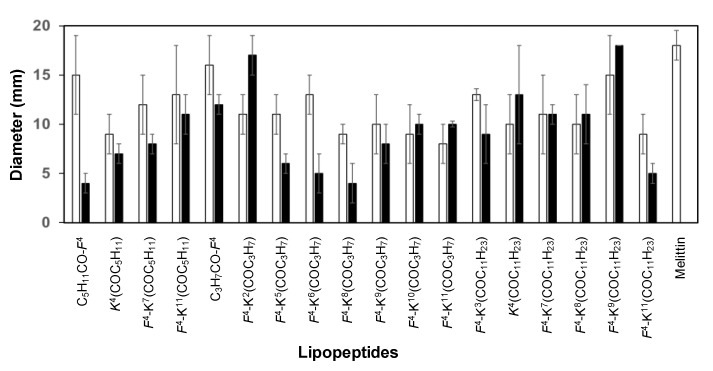
Effect of the lipopeptides incorporating all L-amino acids or a D-amino acid on the size of the lesions in infiltrated tobacco leaves at 250 µM. This effect was compared to melittin. Vertical bars within each column indicate confidence interval at the mean. The x axis includes the code for each lipopeptide. The residue that can be a L- or a D-amino acid is indicated in italics. Black bars correspond to lipopeptides with a D-amino acid and white bars to lipopeptides with all L-amino acids. Data can be found in Table S3 (Supplementary Materials).

The biological activity of the D-amino acid-containing lipopeptides was compared to that of the corresponding parent lipopeptides with all L-amino acids [46] in order to analyse the influence of incorporating a D-amino acid (Figure 4, Figure 5 and Figure 6). Regarding the antimicrobial activity, a different trend was observed depending on the fatty acid length (Figure 4). In the case of the lauroyl derivatives, the antimicrobial activity was maintained or improved against all the pathogens, except for *F. oxysporum*. The lipopeptides bearing a D-amino acid and a butanoyl or a hexanoyl chain were similarly active or even more active than the corresponding all L-lipopeptides against the two *Pseudomonas* species, *X. arboricola* pv. *pruni* and the two fungi. As expected, the incorporation of a D-amino acid resulted in a decrease of the hemolysis for all peptides bearing a butanoyl or a hexanoyl moiety (Figure 5). Remarkably, in some cases, this decrease was considerable. In contrast, the hemolysis did not improve for peptides incorporating a lauroyl group. Probably, the benefit of incorporating of a D-amino acid was not able to counteract the hydrophobicity of a 12-carbon atom lauroyl group. Concerning the phytotoxicity, a smaller size of the lesion compared to that of the L-lipopeptides was observed for the sequences bearing a butanoyl or a hexanoyl moiety (Figure 6).

All these results led us to identify **BP475** (D-F^4^-K^10^(COC_3_H_7_) and **BP485** (C_3_H_7_CO-D-F^4^) as the D-amino acid containing lipopeptides with the best biological activity profile. Both sequences contain a butanoyl group and a D-Phe at position 4. These peptides exhibited MIC values between 0.8 and 6.2 µM against 7 out of the 8 pathogens tested, were significantly less hemolytic (0 and 24% at 250 µM, respectively) than the corresponding all-L derivatives (22 and 93% at 250 µM, respectively), and were low phytotoxic. Interestingly, these lipopeptides are comparable in terms of activity to antibiotics used in agriculture for bacterial disease control, such as streptomycin, which is effective in vitro at 2 to 9 µM.

Characterization by NMR of the secondary structure of **BP475** in the presence of CF_3_CD_2_OD evidenced that, as expected, the D-Phe at position 4 disrupts the α-helix, whereas the incorporation of an acyl lysine at position 10 has no effect. Accordingly, chemical shift index analysis pointed out that residues 6 to 10 of this lipopeptide fold into an α-helix. In contrast, the whole sequence of the L-Phe-containing analog **BP389** adopts an α-helical structure. The high antimicrobial activity displayed by these two lipopeptides stresses the importance of the C-terminal α-helix in this activity. These results are in accordance with the carpet mechanism reported for the parent peptide **BP100,** which involves the insertion of its C-terminus into the hydrophobic core of the bilayer, resulting in membrane permeabilization [56]. Assuming a similar mechanism for lipopeptides **BP389** and **BP475**, the presence of the acyl group in the C-terminal α-helix region would favour their insertion into the membrane, thereby leading to a higher antimicrobial activity than **BP100**. Regarding the hemolysis, the disruption of the α-helical structure of **BP475** due to the presence of the D-Phe could explain the low hemolytic activity displayed by this peptide compared to its L-counterpart **BP389,** which is in agreement with previous reports [33].

## 4. Materials and Methods

### 4.1. General Methods

Manual peptide synthesis was performed in polypropylene syringes (2 or 5 mL) fitted with a porous polyethylene disk. Solvents and soluble reagents were removed by suction. Most chemicals were purchased from commercial suppliers Merck (Madrid, Spain), Iris Biotech GmbH (Marktredwitz, Germany), Scharlab (Sentmenat, Spain), Carlo Erba Reagents (Sabadell, Spain) or Panreac (Castellar del Vallès, Spain), and used without further purification.

Peptides were analyzed under standard analytical HPLC conditions with a Dionex liquid chromatography instrument composed of a UV/Vis Dionex UVD170U detector, a P680 Dionex pump, an ASI-100 Dionex automatic injector, and CHROMELEON 6.60 software. Detection was performed at a wavelength of 220 nm. Solvent A was 0.1% aqueous TFA and solvent B was 0.1% TFA in CH_3_CN. Analyses were carried out with a Kromasil 100 C_18_ (4.6 mm × 40 mm, 3 μm) column with a linear gradient of 2 to 100% B over 7 min at a flow rate of 1 mL/min. Peptides were also analysed with a 1260 Infinity II liquid chromatography instrument (Agilent Technologies) composed of a Diode Array Detector HS, a Quaternary Pump VL, a 1260 Vial sampler and OpenLab CDS ChemStation software. Analyses were carried out with a Kromasil 100 C_18_ (4.6 mm × 40 mm, 3 μm) column with a linear gradient of 2 to 100% B over 12 min at a flow rate of 1 mL/min.

All purifications were performed on a Combi*Flash* Rf200 automated flash chromatography system using Redi*Sep* Rf Gold reversed-phase column packed with high performance C_18_ derivatized silica.

ESI-MS analyses were performed at the Serveis Tècnics de Recerca of the University of Girona with an Esquire 6000 ESI ion Trap LC/MS (Bruker Daltonics) instrument equipped with an electrospray ion source. The instrument was operated in the positive ESI(+) ion mode. Samples (5 μL) were introduced into the mass spectrometer ion source directly through an HPLC autosampler. The mobile phase (80:20 CH_3_CN/H_2_O at a flow rate of 100 μL/min) was delivered by a 1200 Series HPLC pump (Agilent). Nitrogen was employed as both the drying and nebulising gas.

HRMS were recorded on a Bruker MicroTof-QIITM instrument using ESI ionization source at the Serveis Tècnics de Recerca of the University of Girona. Samples were introduced into the mass spectrometer ion source by direct infusion using a syringe pump and were externally calibrated using sodium formate. The instrument was operated in the positive ion mode.

### 4.2. Synthesis of Lipopeptides

These lipopeptides were synthesized manually by the solid-phase method using standard Fmoc chemistry as described previously [46]. The Fmoc-Rink-MBHA resin (0.56 mmol/g) was used as a solid support. Fmoc-Leu-OH, Fmoc-Lys(Boc)-OH, Fmoc-Lys(ivDde)-OH, Fmoc-D-Lys(ivDde)-OH, Fmoc-Phe-OH, Fmoc-Ile-OH, Fmoc-D-Phe-OH and Fmoc-Tyr(*t*Bu)-OH were used as amino acid derivatives. Peptide elongation was carried out through sequential Fmoc removal and coupling of the corresponding amino acid. Fmoc group removal was achieved with piperidine/*N,N*-dimethylformamide (DMF) (3:7, 2 + 10 min). Couplings of the Fmoc-amino acids (4 equiv.) were mediated by ethyl 2-ciano-2-(hydroxyimino)acetate (Oxyma) (4 equiv.) and *N,N*’-diisopropylcarbodiimide (DIC) (4 equiv.) in DMF at room temperature for 1 h under stirring. The completion of the reactions was checked with the Kaiser test [57]. After each coupling and deprotection step, the resin was washed with DMF (6 × 1 min) and CH_2_Cl_2_ (2 × 1 min). Once the peptide elongation was completed, the peptidyl resin was treated with piperidine/*N*-methyl-2-pyrrolidinone (NMP) (3:7, 2 + 10 min), washed with NMP (6 × 1 min), and CH_2_Cl_2_ (2 × 1 min), and air dried.

For lipopeptides **BP472** and **BP485,** the N-terminal deprotected resin was acylated by treatment with the corresponding fatty acid (3 equiv.), DIC (3 equiv.) and Oxyma (3 equiv.) in NMP under stirring overnight. After this time, the resin was washed with NMP (6 × 1 min) and CH_2_Cl_2_ (6 × 1 min), and air dried. Completion of the reaction was checked with the Kaiser test [57].

In the case of the side chain acylated derivatives, the N-terminal deprotected resin was acetylated with acetic anhydride (Ac_2_O)/pyridine/CH_2_Cl_2_ (1:1:1; 2 × 30 min), washed with NMP (6 × 1 min) and CH_2_Cl_2_ (6 × 1 min), and air dried. Completion of the reaction was checked with the Kaiser test [57]. The resulting resin was treated with NH_2_NH_2_·H_2_O/NMP (2:98, 10 × 20 min) under stirring and washed with NMP (2 × 1 min), CH_2_Cl_2_ (2 × 1 min), MeOH (2 × 1 min), and NMP (2 × 1 min). Then, the resin was acylated by treatment with the corresponding fatty acid (3 equiv.), DIC (3 equiv.) and Oxyma (3 equiv.) in NMP under stirring overnight. The resin was then washed with NMP (6 × 1 min) and CH_2_Cl_2_ (6 × 1 min), and air dried. Completion of the reaction was checked with the Kaiser test [57].

Finally, each resulting peptidyl resin was treated with TFA/H_2_O/TIS (95:2.5:2.5) for 2 h. Following TFA evaporation and diethyl ether extraction, the crude lipopeptide was purified by reverse-phase column chromatography, lyophilized, analysed by HPLC, and characterized by mass spectrometry.

### 4.3. Bacterial and Fungal Strains and Growth Conditions

The following plant pathogenic bacterial strains were used: *Erwinia amylovora* PMV6076 (Institut National de la Recherche Agronomique, Angers, France), *Pseudomonas syringae* pv. syringae EPS94 (Institut de Tecnologia Agroalimentària, University of Girona, Spain), *Xanthomonas axonopodis* pv. vesicatoria 2133–2, *Pseudomonas syringae* pv. actinidiae Psa3700.1.1, *Xanthomonas fragariae* Xf349-9A (Instituto Valenciano de Investigaciones Agrarias, Valencia, Spain), and *Xanthomonas arboricola* pv. pruni CFBP5563 (Collection Française de Bactéries Associées aux Plantes, Angers, France). All bacteria except for *X. fragariae* were stored in Luria Bertani (LB) broth supplemented with glycerol (20%) and maintained at −80 °C. For *X. fragariae*, Medium B [58] was used instead of LB. *E. amylovora*, *X. arboricola* pv. pruni, *P. syringae* pv. syringae and *P. syringae* pv. actinidiae were scrapped from the agar media after growing for 24 h at 25 °C, and *X. axonopodis* pv. vesicatoria and *X. fragariae* after growing for 48 h at 25 °C. The cell material was suspended in sterile water to obtain a suspension of 10^8^ CFU mL^−1^. The following plant pathogenic fungal strains were used: *Penicillium expansum* EPS26 (Institut de Tecnologia Agroalimentària, University of Girona, Spain) and *Fusarium oxysporum* f. sp. lycopersici FOL 3 race 2 (ATCC 201829, American Type Culture Collection, Virginia, EEUU). Strains were cultured on potato dextrose agar (PDA) plates (Difco). Conidia from *P. expansum* and microconidia from *F. oxysporum* were obtained from five- to seven-day-old PDA cultures after growth at 25 °C. Inoculum was prepared by scraping spore material from culture surfaces with a cotton swab and resuspending it in distilled water containing 0.5‰ of tween 80. The suspensions were filtered through Miracloth (Merk, Millipore) and the concentration of conidia was determined using a hemacytometer and adjusted to 10^4^ conidia mL^−1^ for *F. oxysporum* and to 10^3^ conidia mL^−1^ for *P. expansum*.

### 4.4. Antimicrobial Activity

Lyophilized peptides were solubilized in sterile Milli-Q water to a final concentration of 1 mM and filter sterilized through a 0.22-μm pore filter. For minimum inhibitory concentration (MIC) assessment, dilutions of the compounds were made to obtain a stock concentration of 250, 125, 62, 31, 16, 8 and 4 μM. For antibacterial activity, 20 μL of each dilution were mixed in a microtiter plate well with 20 μL of the corresponding suspension of the bacterial indicator, 160 μL of trypticase soy broth (TSB) (BioMèrieux, France) to a total volume of 200 μL. For antifungal activity, 20 μL of each stock solution were mixed in a microtiter plate well with 80 μL of the corresponding suspension of the fungal pathogen and 100 μL of double concentrated potato dextrose broth (PDB) to a total volume of 200 μL containing 0.003% w/v of chloramphenicol to prevent bacterial contamination. Three replicates for each combination of strain, compound and concentration were used.

Microbial growth was determined by optical density measurement at 600 nm (Bioscreen C, Labsystem, Helsinki, Finland). For antibacterial activity, microplates were incubated at 25 °C with 10 s shaking before hourly absorbance measurement for 48 h. For antifungal activity, microplates were incubated at 22 °C with 1 min shaking before absorbance measurement carried out every 2 h for seven days. The experiment was repeated twice. The MIC was taken as the lowest compound concentration with no growth at the end of the experiment.

### 4.5. Hemolytic Activity

The hemolytic activity of the compounds was evaluated by determining hemoglobin release from erythrocyte suspensions of horse blood (5% vol/vol) (Oxoid) as previously described [59]. Blood was centrifuged at 6000 g for 5 min, washed three times with tris(hydroxymethyl)aminomethane (TRIS) buffer (10 mM TRIS, 150 mM NaCl, pH 7.2) and diluted 10 times. Compounds were solubilized in TRIS buffer at 750, 500, 300 and 100 μM and mixed with horse erythrocytes (1:1 v/v). The mixture was incubated under continuous shaking for 1 h at 37 °C. Then, the tubes were centrifuged at 3500 g for 10 min, 80 μL aliquots of the supernatant transferred to 100-well microplates (Bioscreen), diluted with 80 μL water, and the absorbance measured at 540 nm (Bioscreen). Complete hemolysis was obtained by the addition of melittin at 100 μM (Sigma-Aldrich Corporation, Madrid, Spain). The percentage of hemolysis (*H*) was calculated using the equation: *H* = 100 × [(*Op*−*Ob*)/(*Om*−*Ob*)], where *Op* is the density for a given compound concentration, *Ob* for the buffer, and *Om* for the melittin-positive control.

### 4.6. Effect of Peptide Infiltration on Tobacco Leaves

The lipopeptides were evaluated for their effect upon infiltration on tobacco leaves as described previously [60]. Peptide solutions of 50, 150 and 250 μM were infiltrated (100 μL) into the mesophylls of fully expanded tobacco leaves. Infiltrations were carried out in a single leaf, and for each peptide and dose, at least three leaves randomly distributed in different plants were infiltrated. Control infiltrations with water (negative control) or melittin (positive control) at the same molar concentration were performed. The appearance of symptoms on the leaves was followed for 48 h after infiltration and measured as a lesion diameter.

### 4.7. Structural Characterization by NMR Spectroscopy

The structure of lipopeptides **BP389** and **BP475** was determined by NMR spectroscopy. NMR spectra were acquired at the Serveis Tècnics de Recerca of the University of Girona with a Ultrashield 400 MHz spectrometer equipped with an RT BBI. Each peptide was characterized with the following experiments: 1D ^1^H-NMR; 2D ^1^H-^1^H TOCSY (mixing time = 80 ms); 2D ^1^H-^1^H NOESY (mixing time = 400 ms); 2D ^1^H-^13^C multiplicity-edited HSQC; 2D ^1^H-^15^N HSQC; 2D ^1^H-^13^C HSQC-TOCSY. Water suppression was achieved with excitation sculpting or Watergate scheme. NMR spectra were processed and analyzed using TopSpin 3.6.2. All experiments were conducted at 10 °C using a shigemi tube calibrated for D_2_O. Five milligrams of sample were dissolved in 400 µL of 20 mM phosphate buffer at pH 6.5 in H_2_O/D_2_O (90:10) or in 400 µL of this buffer containing 30% of 2,2,2-trifluoroethanol-d_3_ to induce the formation of the secondary structure. From NMR assignments, the structural analysis was achieved with Chemical Shift Index 3.0 web server [61,62].

## 5. Conclusions

In summary, we designed and synthesized D-amino-containing lipopeptides derived from **BP100**. These lipopeptides displayed an improved biological activity profile compared to their L-counterparts. Remarkably, replacement of the L-Phe at position 4 with its enantiomer provided less hemolytic lipopeptides. The best derivatives—**BP475** (D-F^4^-K^10^(COC_3_H_7_) and **BP485** (C_3_H_7_CO-D-F^4^)—exhibited high antimicrobial activity (MIC between 0.8 and 6.2 µM) together with a low hemolysis (0 and 24% at 250 µM, respectively). In addition, the results from the NMR experiments of **BP475** demonstrate the importance of a C-terminal α-helix in the activity of these lipopeptides. This study provides tools for the design of new agents to control plant pathogens.

## Figures and Tables

**Table 1:** Sequences, retention times and purities on HPLC, and mass spectrometry data of lipopeptides.

Peptide	Sequence ^1^	Code	*t*_R_ (min) ^2^	Purity (%) ^3^	HRMS (ESI)		
						Calcd.	Found
**BP472**	C_5_H_11_CO-KKLfKKILKYL-NH_2_	C_5_H_11_CO-D-F^4^	6.14	>99	C_78_H_137_N_17_O_13_ [M + 2H]^2+^	760.0285	760.0260
**BP473**	Ac-KKLfKK(COC_3_H_7_)ILKYL-NH_2_	D-F^4^-K^6^(COC_3_H_7_)	5.99	>99	C_78_H_135_N_17_O_14_ [M + 2H]^2+^	767.0182	767.0158
**BP474**	Ac-KKLfKKIK(COC_3_H_7_)KYL-NH_2_	D-F^4^-K^8^(COC_3_H_7_)	5.36	>99	C_78_H_136_N_18_O_14_ [M + 2H]^2+^	774.5236	774.5208
**BP475**	Ac-KKLfKKILKK(COC_3_H_7_)L-NH_2_	D-F^4^-K^10^(COC_3_H_7_)	5.69	>99	C_75_H_138_N_18_O_13_ [M + 2H]^2+^	749.5340	749.5343
**BP476**	Ac-KKLfKKILKYK(COC_11_H_23_)-NH_2_	D-F^4^-K^11^(COC_11_H_23_)	6.67	>99	C_86_H_152_N_18_O_14_ [M + 2H]^2+^	830.5862	830.5839
**BP484**	Ac-KKLk(COC_5_H_11_)KKILKYL-NH_2_	D-K^4^(COC_5_H_11_)	6.45	>99	C_77_H_142_N_18_O_14_ [M + 2H]^2+^	771.5471	771.5477
**BP485**	C_3_H_7_CO-KKLfKKILKYL-NH_2_	C_3_H_7_CO-D-F^4^	6.79	>99	C_76_H_133_N_17_O_13_ [M + 2H]^2+^	746.0129	746.0097
**BP486**	Ac-KK(COC_3_H_7_)LfKKILKYL-NH_2_	D-F^4^-K^2^(COC_3_H_7_)	6.17	>99	C_78_H_134_N_17_O_14_ [M + H]^+^	1533.0291	1533.0266
**BP488**	Ac-KKLk(COC_11_H_23_)KKILKYL-NH_2_	D-K^4^(COC_11_H_23_)	6.80	>99	C_83_H_153_N_18_O_14_ [M + H]^+^	1626.1808	1626.1787
**BP489**	Ac-KKLfKKK(COC_11_H_23_)LKYL-NH_2_	D-F^4^-K^7^(COC_11_H_23_)	6.76	>99	C_86_H_152_N_18_O_14_ [M + 2H]^2+^	830.5862	830.5825
**BP490**	Ac-KKLfKKIK(COC_11_H_23_)KYL-NH_2_	D-F^4^-K^8^(COC_11_H_23_)	7.12	>99	C_86_H_151_N_18_O_14_ [M + H]^+^	1660.1652	1660.1635
**BP494**	Ac-KKLfKKK(COC_5_H_11_)LKYL-NH_2_	D-F^4^-K^7^(COC_5_H_11_)	5.42	>99	C_80_H_139_N_18_O_14_ [M + H]^+^	1576.0713	1576.0683
**BP495**	Ac-KKLfKKILKYK(COC_5_H_11_)-NH_2_	D-F^4^-K^11^(COC_5_H_11_)	5.44	>99	C_80_H_139_N_18_O_14_ [M + H]^+^	1576.0713	1576.0683
**BP496**	Ac-KKLfKKILKYK(COC_3_H_7_)-NH_2_	D-F^4^-K^11^(COC_3_H_7_)	5.11	>99	C_78_H_135_N_18_O_14_ [M + H]^+^	1548.0400	1548.0367
**BP497**	Ac-KKLfKKILK(COC_11_H_23_)YL-NH_2_	D-F^4^-K^9^(COC_11_H_23_)	7.21	>99	C_86_H_150_N_17_O_14_ [M + H]^+^	1645.1543	1645.1516
**BP498**	Ac-KKLfK(COC_3_H_7_)KILKYL-NH_2_	D-F^4^-K^5^(COC_3_H_7_)	6.12	>99	C_78_H_135_N_17_O_14_ [M + 2H]^2+^	767.0182	767.0147
**BP499**	Ac-KKLfKKILK(COC_3_H_7_)YL-NH_2_	D-F^4^-K^9^(COC_3_H_7_)	5.89	>99	C_78_H_134_N_17_O_14_ [M + H]^+^	1533.0291	1533.0269
**BP500**	Ac-KKK(COC_11_H_23_)fKKILKYL-NH_2_	D-F^4^-K^3^(COC_11_H_23_)	6.80	>99	C_86_H_151_N_18_O_14_ [M + H]^+^	1661.1684	1661.1667

^1^ COC_3_H_7_, butanoyl; COC_5_H_11_, hexanoyl; COC_11_H_23_, lauroyl; lower case letters correspond to D-amino acids. ^2^ HPLC retention time. ^3^ Percentage determined by HPLC at 220 nm after purification.

**Table 2:** Hemolytic activity and size of the lesion in infiltrated tobacco leaves of lipopeptides.

Peptide	Code	Hemolysis (%) ^1^ 250 µM	Size of the Lesion (mm) ^2^ 250 µM
**BP472**	C_5_H_11_CO-D-F^4^	78 ± 5	4 ± 1
**BP473**	D-F^4^-K^6^(COC_3_H_7_)	84 ± 11	5 ± 2
**BP474**	D-F^4^-K^8^(COC_3_H_7_)	0 ± 0	4 ± 2
**BP475**	D-F^4^-K^10^(COC_3_H_7_)	0 ± 0	10 ± 1
**BP476**	D-F^4^-K^11^(COC_11_H_23_)	94 ± 10	5 ± 0.5
**BP484**	D-K^4^(COC_5_H_11_)	10 ± 2	7 ± 1
**BP485**	C_3_H_7_CO-D-F^4^	24 ± 9	12 ± 1
**BP486**	D-F^4^-K^2^(COC_3_H_7_)	14 ± 5	17 ± 2
**BP488**	D-K^4^(COC_11_H_23_)	86 ± 14	13 ± 5
**BP489**	D-F^4^-K^7^(COC_11_H_23_)	100 ± 6	11 ± 1
**BP490**	D-F^4^-K^8^(COC_11_H_23_)	100 ± 6	11 ±3
**BP494**	D-F^4^-K^7^(COC_5_H_11_)	0.2 ± 0.2	8 ± 1
**BP495**	D-F^4^-K^11^(COC_5_H_11_)	0.6 ± 1	11 ± 2
**BP496**	D-F^4^-K^11^(COC_3_H_7_)	1 ± 1	10 ± 3
**BP497**	D-F^4^-K^9^(COC_11_H_23_)	100 ± 2	18 ± 0
**BP498**	D-F^4^-K^5^(COC_3_H_7_)	21 ± 3	6 ± 1
**BP499**	D-F^4^-K^9^(COC_3_H_7_)	11 ± 1	8 ± 2
**BP500**	D-F^4^-K^3^(COC_11_H_23_)	71 ± 8	9 ± 3

^1^ Percent hemolysis plus confidence interval (α = 0.05). ^2^ Effect on the size of the lesion in infiltrated tobacco leaves plus confidence interval.

## Data Availability

Samples of the compounds are available from the authors.

## Supplementary Materials

The following are available online at https://www.mdpi.com/article/10.3390/ijms22126631/s1: biological activity of lipopeptides; Synthesis of lipopeptides; HPLC of crude and purified lipopeptides; ESI-MS and HRMS of purified lipopeptides; NMR experiments of lipopeptides **BP389** and **BP475**.
