# Supplementary material for: D-Amino Acid-Containing Lipopeptides Derived from the Lead Peptide BP100 with Activity against Plant Pathogens
Source: Int J Mol Sci. 2021 Jun 21;22(12):6631. doi: 10.3390/ijms22126631 (PMC8233901; doi:10.3390/ijms22126631)
Supplement: Supplementary file 1 [file ijms-22-06631-s001.zip › ijms-1258606-supplementary.pdf]

Supporting Information

D-Amino Acid-Containing Lipopeptides Derived from the Lead Peptide BP100 with Activity against Plant Pathogens

Àngel Oliveras [1], Luís Moll [2], Gerard Riesco-Llach [1], Arnau Tolosa-Canudas [1], Sergio Gil-Caballero [3], Esther Badosa [2], Anna Bonaterra [2], Emilio Montesinos [2], Marta Planas [1,*] and Lidia Feliu [1,*]

Table of contents:

1. Biological activity of lipopeptides 2
2. Synthesis of lipopeptides 5
3. HPLC of crude and purified lipopeptides, ESI-MS and HRMS of purified lipopeptides 11
4. NMR experiments of lipopeptides **BP389** and **BP475** 81

1. Biological activity of lipopeptides

Table S1. Antimicrobial activity (MIC) of lipopeptides

Peptide[a]	MIC (µM)							
	***Ea*[b]**	***Pss*[b]**	***Xap*[b]**	***Xf*[b]**	***Psa*[b]**	***Xav*[b]**	***Pe*[b]**	***Fo*[b]**
**BP367**	3.1-6.2	3.1-6.2	3.1-6.2	3.1-6.2	3.1-6.2	3.1-6.2	12.5-25	1.6-3.1
**BP472**	3.1-6.2	3.1-6.2	1.6-3.1	1.6-3.1	1.6-3.1	1.6-3.1	>25	3.1-6.2
**BP371**	3.1-6.2	3.1- 6.2	3.1- 6.2	3.1-6.2	3.1-6.2	3.1-6.2	3.1-6.2	0.8-1.6
**BP484**	6.2-12.5	3.1-6.2	3.1-6.2	1.6-3.1	3.1-6.2	6.2-12.5	6.2-12.5	1.6-3.1
**BP374**	3.1-6.2	6.2-12.5	6.2-12.5	3.1-6.2	3.1-6.2	6.2-12.5	>25	0.8-1.6
**BP494**	6.2-12.5	3.1-6.2	6.2-12.5	12.5-25	3.1-6.2	6.2-12.5	0.8-1.6	3.1-6.2
**BP378**	3.1-6.2	6.2-12.5	3.1-6.2	3.1-6.2	3.1-6.2	3.1-6.2	3.1-6.2	0.8-1.6
**BP495**	3.1-6.2	3.1-6.2	3.1-6.2	6.2-12.5	1.6-3.1	6.2-12.5	0.8-1.6	1.6-3.1
**BP379**	3.1-6.2	3.1-6.2	3.1-6.2	3.1-6.2	3.1-6.2	3.1-6.2	6.2-12.5	3.1-6.2
**BP485**	6.2-12.5	3.1-6.2	1.6-3.1	1.6-3.1	3.1-6.2	1.6-3.1	3.1-6.2	1.6-3.1
**BP381**	3.1-6.2	3.1-6.2	3.1-6.2	1.6-3.1	3.1-6.2	1.6-3.1	1.6-3.1	0.8-1.6
**BP486**	6.2-12.5	12.5-25	3.1-6.2	3.1-6.2	6.2-12.5	6.2-12.5	6.2-12.5	0.8-1.6
**BP384**	3.1-6.2	6.2-12.5	3.1-6.2	1.6-3.1	6.2-12.5	1.6-3.1	12.5-25	1.6-3.1
**BP498**	6.2-12.5	6.2-12.5	1.6-3.1	3.1-6.2	6.2-12.5	1.6-3.1	1.6-3.1	0.8-1.6
**BP385**	6.2-12.5	6.2-12.5	0.8-1.6	1.6-3.1	6.2-12.5	0.8-1.6	6.2-12.5	1.6-3.1
**BP473**	3.1-6.2	6.2-12.5	1.6-3.1	1.6-3.1	3.1-6.2	1.6-3.1	>25	0.8-1.6
**BP387**	3.1-6.2	3.1-6.2	3.1-6.2	1.6-3.1	3.1-6.2	1.6-3.1	6.2-12.5	0.8-1.6
**BP474**	6.2-12.5	3.1-6.2	3.1-6.2	6.2-12.5	1.6-3.1	3.1-6.2	3.1-6.2	0.8-1.6
**BP388**	3.1-6.2	6.2-12.5	3.1-6.2	1.6-3.1	6.2-12.5	1.6-3.1	6.2-12.5	0.8-1.6
**BP499**	3.1-6.2	6.2-12.5	3.1-6.2	3.1-6.2	3.1-6.2	1.6-3.1	1.6-3.1	0.8-1.6
**BP389**	3.1-6.2	6.2-12.5	0.8-1.6	1.6-3.1	3.1-6.2	0.8-1.6	6.2-12.5	1.6-3.1
**BP475**	3.1-6.2	3.1-6.2	0.8-1.6	1.6-3.1	1.6-3.1	1.6-3.1	3.1-6.2	0.8-1.6
**BP390**	3.1-6.2	6.2-12.5	6.2-12.5	3.1-6.2	6.2-12.5	3.1-6.2	6.2-12.5	0.8-1.6
**BP496**	6.2-12.5	6.2-12.5	1.6-3.1	12.5-25	3.1-6.2	12.5-25	3.1-6.2	1.6-3.1
**BP394**	12.5-25	6.2-12.5	1.6-3.1	0.8- 1.6	6.2-12.5	1.6-3.1	12.5-25	6.2-12.5
**BP500**	6.2-12.5	3.1-6.2	1.6-3.1	1.6-3.1	3.1-6.2	0.8-1.6	6.2-12.5	3.1-6.2
**BP395**	12.5-25	3.1-6.2	1.6-3.1	1.6-3.1	3.1-6.2	1.6-3.1	12.5-25	6.2-12.5
**BP488**	6.2-12.5	3.1-6.2	1.6-3.1	1.6-3.1	3.1-6.2	1.6-3.1	12.5-25	>25
**BP398**	>25	3.1-6.2	1.6-3.1	3.1-6.2	3.1-6.2	0.8-1.6	>25	6.2-12.5
**BP489**	6.2-12.5	3.1-6.2	1.6-3.1	3.1-6.2	3.1-6.2	1.6-3.1	12.5-25	>25
**BP399**	>25	3.1-6.2	1.6-3.1	3.1-6.2	3.1-6.2	0.8-1.6	12.5-25	6.2-12.5
**BP490**	>25	6.2-12.5	1.6-3.1	1.6-3.1	3.1-6.2	1.6-3.1	12.5-25	>25
**BP400**	>25	>25	1.6-3.1	3.1-6.2	>25	1.6-3.1	>25	6.2-12.5
**BP497**	>25	>25	1.6-3.1	0.8-1.6	>25	1.6-3.1	12.5-25	>25
**BP402**	3.1-6.2	3.1-6.2	1.6-3.1	3.1-6.2	3.1-6.2	1.6-3.1	12.5-25	6.2-12.5
**BP476**	6.2-12.5	6.2-12.5	1.6-3.1	1.6-3.1	1.6-3.1	1.6-3.1	12.5-25	12.5-25

[a]For each group of peptides, the top sequence corresponds to the all-L derivative

[b]*Ea*, *Erwinia amylovora*; *Pss*, *Pseudomonas syringae* pv. syringae; *Xap*, *Xanthomonas arboricola* pv. pruni; *Xf*, *Xanthomonas fragariae*; *Psa*, *Pseudomonas syringae* pv. actinidiae; *Xav*, *Xanthomonas axonopodis* pv. vesicatoria; *Pe*, *Penicillium expansum*; *Fo*, *Fusarium oxysporum*.

Table S2. Hemolytic activity

Lipopeptide[a]	Hemolysis (%)[b]			
	50 µM	150 µM	250 µM	375 µM
**BP367**	89 ± 2	87 ± 14	83 ± 6	95 ± 13
**BP472**	21 ± 5	66 ± 3	78 ± 5	90 ± 3
**BP371**	22 ± 4	47 ± 2	75 ± 4	95 ± 3
**BP484**	3 ± 0.1	5 ± 0.9	10 ± 2	15 ± 4
**BP374**	4 ± 2	8 ± 0.4	19 ± 3	28 ± 2
**BP494**	0 ± 0.5	0.3 ± 0.8	0.2 ± 0.2	0.4 ± 0.5
**BP378**	15 ± 7	21 ± 2	26 ± 0.4	52 ± 6
**BP495**	0 ± 0.4	0 ± 0.4	0.6 ± 1	1 ± 2
**BP379**	60 ± 10	86 ± 2	93 ± 0.8	96 ± 1
**BP485**	9 ± 0.4	16 ± 4	24 ± 9	46 ± 5
**BP381**	11 ± 2	30 ± 3	54 ± 6	76 ± 2
**BP486**	3 ± 2	6 ± 2	14 ± 5	14 ± 2
**BP384**	41 ± 15	97 ± 10	100 ± 4	100 ± 2
**BP498**	6 ± 1	17 ± 2	21 ± 3	36 ± 2
**BP385**	42 ± 3	98 ± 8	100 ± 3	100 ± 4
**BP473**	7 ± 2	41 ± 4	84 ± 11	75 ± 12
**BP387**	4 ± 0.9	11 ± 5	14 ± 0.5	18 ± 1
**BP474**	0 ± 0	0 ± 0	0 ± 0	0 ± 0
**BP388**	10 ± 1	32 ± 3	38 ± 4	89 ± 10
**BP499**	3 ± 0.8	7 ± 0.3	11 ± 1	17 ± 1
**BP389**	9 ± 4	17 ± 0.7	22 ± 2	39 ± 3
**BP475**	0 ± 0	0 ± 0	0 ± 0	0 ± 0
**BP390**	1 ± 0.2	2 ± 0.2	5 ± 0.3	7 ± 0.9
**BP496**	0 ± 1	0 ± 0.2	1 ± 1	1 ± 0.5
**BP394**	100 ± 7	100 ± 7	100 ± 1	100 ± 2
**BP500**	74 ± 5	71 ± 2	71 ± 8	77 ± 5
**BP395**	100 ± 2	100 ± 2	100 ± 2	100 ± 2
**BP488**	71 ± 2	84 ± 9	86 ± 14	82 ± 7
**BP398**	70 ± 18	100 ± 9	100 ± 3	100 ± 5
**BP489**	50 ± 5	83 ± 15	100 ± 6	100 ± 1
**BP399**	94 ± 8	100 ± 5	100 ± 5	100 ± 6
**BP490**	100 ± 3	92 ± 17	100 ± 6	98 ± 2
**BP400**	43 ± 3	56 ± 1	99 ± 0.4	100 ± 14
**BP497**	88 ± 14	100 ± 2	100 ± 2	100 ± 4
**BP402**	95 ± 6	93 ± 13	100 ± 4	100 ± 0.5
**BP476**	69 ± 3	79 ± 7	94 ± 10	94 ± 67

[a]For each group of peptides, the top sequence corresponds to the all-L derivative

[b]Percent hemolysis plus confidence interval ($\alpha = 0.05$)

Table S3. Size of the lesion in infiltrated tobacco leaves

Lipopeptide[a]	Size of the lesion (mm)[b]		
	50 µM	150 µM	250 µM
**BP367**	6 ± 0.4	9 ± 2	15 ± 4
**BP472**	2 ± 1	4 ± 1	4 ± 1
**BP371**	2 ± 0.5	-	9 ± 2
**BP484**	0 ± 0	2 ± 2	7 ± 1
**BP374**	3 ± 0.6	9 ± 2	12 ± 3
**BP494**	1 ± 1	6 ± 1	8 ± 1
**BP378**	4 ± 0.7	12 ± 3	13 ± 5
**BP495**	3 ± 0	6 ± 2	11 ± 2
**BP379**	7 ± 1	13 ± 3	16 ± 3
**BP485**	4 ± 0.7	7 ± 0.7	12 ± 1
**BP381**	5 ± 2	11 ± 2	11 ± 2
**BP486**	4 ± 1	8 ± 1	17 ± 2
**BP384**	8 ± 1	11 ± 3	11 ± 2
**BP498**	4 ± 3	6 ± 2	6 ± 1
**BP385**	8 ± 1	13 ± 2	13 ± 2
**BP473**	2 ± 2	4 ± 0.9	5 ± 2
**BP387**	3 ± 0.7	8 ± 1	9 ± 1
**BP474**	0 ± 0	2 ± 2	4 ± 2
**BP388**	8 ± 2	11 ± 0.4	10 ± 3
**BP499**	6 ± 1	8 ± 2	8 ± 2
**BP389**	4 ± 1	7 ± 1	9 ± 3
**BP475**	5 ± 1	7 ± 2	10 ± 1
**BP390**	3 ± 0.7	7 ± 1	8 ± 2
**BP496**	3 ± 0.5	4 ± 0.5	10 ± 3
**BP394**	3 ± 0.7	12 ± 1	13 ± 0.6
**BP500**	3 ± 2	6 ± 2	9 ± 3
**BP395**	8 ± 0.7	11 ± 2	10 ± 3
**BP488**	4 ± 0.7	5 ± 1	13 ± 5
**BP398**	5 ± 0.5	9 ± 2	11 ± 4
**BP489**	2 ± 0.5	6 ± 2	11 ± 1
**BP399**	5 ± 0.8	8 ± 2	10 ± 3
**BP490**	4 ± 0.7	6 ± 2	11 ± 3
**BP400**	7 ± 1	11 ± 2	15 ± 4
**BP497**	5 ± 1	18 ± 1	18 ± 0
**BP402**	4 ± 1	10 ± 2	9 ± 2
**BP476**	3 ± 0.6	3 ± 0.5	5 ± 0.5

[a]For each group of peptides, the top sequence corresponds to the all-L derivative
[b]Effect on the size of the lesion in infiltrated tobacco leaves plus confidence interval

2. Synthesis of lipopeptides

C_5H_{11}CO-Lys-Lys-Leu-D-Phe-Lys-Lys-Ile-Leu-Lys-Tyr-Leu-NH_2 (BP472)

This lipopeptide was prepared following the procedure described in the manuscript using hexanoic acid. Acidolytic cleavage of the resulting resin and purification eluting with H_2O/CH_3CN (75:25) afforded C_5H_{11}CO-Lys-Lys-Leu-D-Phe-Lys-Lys-Ile-Leu-Lys-Tyr-Leu-NH_2 (**BP472**) in >99% purity. t_R = 6.14 min. MS (ESI) *m/z*: 760.1 $[M + 2H]^{2+}$, 1519.1 $[M + H]^{+}$; HRMS (ESI) *m/z*: calcd for $C_{78}H_{137}N_{17}O_{13}$ $[M + 2H]^{2+}$ 760.0285, found 760.0260; calcd for $C_{78}H_{136}N_{17}O_{13}$ $[M + H]^{+}$ 1519.0498, found 1519.0511.

Ac-Lys-Lys-Leu-D-Phe-Lys-Lys(COC_3H_7)-Ile-Leu-Lys-Tyr-Leu-NH_2 (BP473)

This lipopeptide was prepared following the procedure described in the manuscript using butyric acid. Acidolytic cleavage of the resulting resin and purification eluting with H_2O/CH_3CN (75:25) afforded Ac-Lys-Lys-Leu-D-Phe-Lys-Lys(COC_3H_7)-Ile-Leu-Lys-Tyr-Leu-NH_2 (**BP473**) in >99% purity. t_R = 5.99 min. MS (ESI) *m/z*: 767.1 $[M + 2H]^{2+}$, 1533.1 $[M + H]^{+}$, 1555.1 $[M + Na]^{+}$; HRMS (ESI) *m/z*: calcd for $C_{78}H_{135}N_{17}O_{14}$ $[M + 2H]^{2+}$ 767.0182, found 767.0158; calcd for $C_{78}H_{134}N_{17}O_{14}$ $[M + H]^{+}$ 1533.0291, found 1533.0240.

Ac-Lys-Lys-Leu-D-Phe-Lys-Lys-Ile-Lys(COC_3H_7)-Lys-Tyr-Leu-NH_2 (BP474)

This lipopeptide was prepared following the procedure described in the manuscript using butyric acid. Acidolytic cleavage of the resulting resin and purification eluting with H_2O/CH_3CN (85:15) afforded Ac-Lys-Lys-Leu-D-Phe-Lys-Lys-Ile-Lys(COC_3H_7)-Lys-Tyr-Leu-NH_2 (**BP474**) in >99% purity. t_R = 5.36 min. MS (ESI) *m/z*: 774.6 $[M + 2H]^{2+}$, 1548.1 $[M + H]^{+}$, 1570.1 $[M + Na]^{+}$; HRMS (ESI) *m/z*: calcd for $C_{78}H_{136}N_{18}O_{14}$ $[M + 2H]^{2+}$ 774.5236, found 774.5208; calcd for $C_{78}H_{135}N_{18}O_{14}$ $[M + H]^{+}$ 1548.0400, found 1548.0381.

Ac-Lys-Lys-Leu-D-Phe-Lys-Lys-Ile-Leu-Lys-Lys(COC_3H_7)-Leu-NH_2 (BP475)

This lipopeptide was prepared following the procedure described in the manuscript using butyric acid. Acidolytic cleavage of the resulting resin and purification eluting with H_2O/CH_3CN (80:20) afforded

Ac-Lys-Lys-Leu-D-Phe-Lys-Lys-Ile-Leu-Lys-Lys(COC_3H_7)-Leu-NH_2 (**BP475**) in >99% purity. t_R = 5.69 min. MS (ESI) *m/z*: 750.1 $[M + 2H]^{2+}$, 1498.2 $[M + H]^+$, 1520.1 $[M + Na]^+$; HRMS (ESI) *m/z*: calcd for $C_{75}H_{138}N_{18}O_{13}$ $[M + 2H]^{2+}$ 749.5340, found 749.5343; calcd for $C_{75}H_{137}N_{18}O_{13}$ $[M + H]^+$ 1498.0607, found 1498.0610; calcd for $C_{75}H_{136}N_{18}O_{13}Na$ $[M + Na]^+$ 1520.0426, found 1520.0424.

Ac-Lys-Lys-Leu-D-Phe-Lys-Lys-Ile-Leu-Lys-Tyr-Lys($COC_{11}H_{23}$)-NH_2 (BP476)

This lipopeptide was prepared following the procedure described in the manuscript using lauric acid. Acidolytic cleavage of the resulting resin and purification eluting with H_2O/CH_3CN (80:20) afforded Ac-Lys-Lys-Leu-D-Phe-Lys-Lys-Ile-Leu-Lys-Tyr-Lys($COC_{11}H_{23}$)-NH_2 (**BP476**) in >99% purity. t_R = 6.67 min. MS (ESI) *m/z*: 830.6 $[M + 2H]^{2+}$, 1660.3 $[M + H]^+$, 1682.2 $[M + Na]^+$; HRMS (ESI) *m/z*: calcd for $C_{86}H_{152}N_{18}O_{14}$ $[M + 2H]^{2+}$ 830.5862, found 830.5839; calcd for $C_{86}H_{151}N_{18}O_{14}$ $[M + H]^+$ 1660.1652, found 1660.1578.

Ac-Lys-Lys-Leu-D-Lys(COC_5H_{11})-Lys-Lys-Ile-Leu-Lys-Tyr-Leu-NH_2 (BP484)

This lipopeptide was prepared following the procedure described in the manuscript using hexanoic acid. Acidolytic cleavage of the resulting resin and purification eluting with H_2O/CH_3CN (80:20) afforded Ac-Lys-Lys-Leu-D-Lys(COC_5H_{11})-Lys-Lys-Ile-Leu-Lys-Tyr-Leu-NH_2 (**BP484**) in >99% purity. t_R = 6.45 min. MS (ESI) *m/z*: 771.5 $[M + 2H]^{2+}$, 1542.0 $[M + H]^+$; HRMS (ESI) *m/z*: calcd for $C_{77}H_{142}N_{18}O_{14}$ $[M + 2H]^{2+}$ 771.5471, found 771.5477; calcd for $C_{77}H_{141}N_{18}O_{14}$ $[M + H]^+$ 1542.0869, found 1542.0812.

C_3H_7CO-Lys-Lys-Leu-D-Phe-Lys-Lys-Ile-Leu-Lys-Tyr-Leu-NH_2 (BP485)

This lipopeptide was prepared following the procedure described in the manuscript using butyric acid. Acidolytic cleavage of the resulting resin and purification eluting with H_2O/CH_3CN (75:25) afforded C_3H_7CO-Lys-Lys-Leu-D-Phe-Lys-Lys-Ile-Leu-Lys-Tyr-Leu-NH_2 (**BP485**) in >99% purity. t_R = 6.79 min. MS (ESI) *m/z*: 746.4 $[M + 2H]^{2+}$, 1491.0 $[M + H]^+$, 1513.1 $[M + Na]^+$; HRMS (ESI) *m/z*: calcd for $C_{76}H_{133}N_{17}O_{13}$ $[M + 2H]^{2+}$ 746.0129, found 746.0097; calcd for $C_{76}H_{132}N_{17}O_{13}$ $[M + H]^+$ 1491.0185, found 1491.0154.

Ac-Lys-Lys(COC_3H_7)-Leu-D-Phe-Lys-Lys-Ile-Leu-Lys-Tyr-Leu-NH_2 (BP486)

This lipopeptide was prepared following the procedure described in the manuscript using butyric acid. Acidolytic cleavage of the resulting resin and purification eluting with H_2O/CH_3CN (75:25) afforded Ac-Lys-Lys(COC_3H_7)-Leu-D-Phe-Lys-Lys-Ile-Leu-Lys-Tyr-Leu-NH_2 (**BP486**) in >99% purity. t_R = 6.17 min. MS (ESI) *m/z*: 767.1 $[M + 2H]^{2+}$, 1533.1 $[M + H]^+$; HRMS (ESI) *m/z*: calcd for $C_{78}H_{134}N_{17}O_{14}$ $[M + H]^+$ 1533.0291, found 1533.0266; calcd for $C_{78}H_{133}N_{17}O_{14}Na$ $[M + Na]^+$ 1555.0110, found 1555.0071.

Ac-Lys-Lys-Leu-D-Lys($COC_{11}H_{23}$)-Lys-Lys-Ile-Leu-Lys-Tyr-Leu-NH_2 (BP488)

This lipopeptide was prepared following the procedure described in the manuscript using lauric acid. Acidolytic cleavage of the resulting resin and purification eluting with H_2O/CH_3CN (75:25) afforded Ac-Lys-Lys-Leu-D-Lys($COC_{11}H_{23}$)-Lys-Lys-Ile-Leu-Lys-Tyr-Leu-NH_2 (**BP488**) in >99% purity. t_R = 6.80 min. MS (ESI) *m/z*: 814.1 $[M + 2H]^{2+}$, 1626.2 $[M + H]^+$; HRMS (ESI) *m/z*: calcd for $C_{83}H_{153}N_{18}O_{14}$ $[M + H]^+$ 1626.1808, found 1626.1787; calcd for $C_{83}H_{152}N_{18}O_{14}Na$ $[M + Na]^+$ 1648.1628, found 1648.1600.

Ac-Lys-Lys-Leu-D-Phe-Lys-Lys-Lys($COC_{11}H_{23}$)-Leu-Lys-Tyr-Leu-NH_2 (BP489)

This lipopeptide was prepared following the procedure described in the manuscript using lauric acid. Acidolytic cleavage of the resulting resin and purification eluting with H_2O/CH_3CN (75:25) afforded Ac-Lys-Lys-Leu-D-Phe-Lys-Lys-Lys($COC_{11}H_{23}$)-Leu-Lys-Tyr-Leu-NH_2 (**BP489**) in >99% purity. t_R = 6.76 min. MS (ESI) *m/z*: 554.4 $[M + 3H]^{3+}$, 831.1 $[M + 2H]^{2+}$, 1660.2 $[M + H]^+$; HRMS (ESI) *m/z*: calcd for $C_{86}H_{152}N_{18}O_{14}$ $[M + 2H]^{2+}$ 830.5862, found 830.5825; calcd for $C_{86}H_{151}N_{18}O_{14}$ $[M + H]^+$ 1660.1652, found 1660.1598.

Ac-Lys-Lys-Leu-D-Phe-Lys-Lys-Ile-Lys($COC_{11}H_{23}$)-Lys-Tyr-Leu-NH_2 (BP490)

This lipopeptide was prepared following the procedure described in the manuscript using lauric acid. Acidolytic cleavage of the resulting resin and purification eluting with H_2O/CH_3CN (75:25) afforded Ac-Lys-Lys-Leu-D-Phe-Lys-Lys-Ile-Lys($COC_{11}H_{23}$)-Lys-Tyr-Leu-NH_2 (**BP490**) in >99% purity. t_R

= 7.12 min. MS (ESI) *m/z*: 554.4 $[M + 3H]^{3+}$, 831.1 $[M + 2H]^{2+}$, 1660.2 $[M + H]^{+}$; HRMS (ESI) *m/z*: calcd for $C_{86}H_{151}N_{18}O_{14}$ $[M + H]^{+}$ 1660.1652, found 1660.1635; calcd for $C_{86}H_{150}N_{18}O_{14}Na$ $[M + Na]^{+}$ 1682.1471, found 1682.1449.

Ac-Lys-Lys-Leu-D-Phe-Lys-Lys-Lys(COC_5H_{11})-Leu-Lys-Tyr-Leu-NH_2 (BP494)

This lipopeptide was prepared following the procedure described in the manuscript using hexanoic acid. Acidolytic cleavage of the resulting resin and purification eluting with H_2O/CH_3CN (80:20) afforded Ac-Lys-Lys-Leu-D-Phe-Lys-Lys-Lys(COC_5H_{11})-Leu-Lys-Tyr-Leu-NH_2 (**BP494**) in >99% purity. t_R = 5.42 min. MS (ESI) *m/z*: 788.6 $[M + 2H]^{2+}$, 1576.1 $[M + H]^{+}$; HRMS (ESI) *m/z*: calcd for $C_{80}H_{139}N_{18}O_{14}$ $[M + H]^{+}$ 1576.0713, found 1576.0683; calcd for $C_{80}H_{138}N_{18}O_{14}Na$ $[M + Na]^{+}$ 1598.0532, found 1598.0490.

Ac-Lys-Lys-Leu-D-Phe-Lys-Lys-Ile-Leu-Lys-Tyr-Lys(COC_5H_{11})-NH_2 (BP495)

This lipopeptide was prepared following the procedure described in the manuscript using hexanoic acid. Acidolytic cleavage of the resulting resin and purification eluting with H_2O/CH_3CN (80:20) afforded Ac-Lys-Lys-Leu-D-Phe-Lys-Lys-Ile-Leu-Lys-Tyr-Lys(COC_5H_{11})-NH_2 (**BP495**) in >99% purity. t_R = 5.44 min. MS (ESI) *m/z*: 789.1 $[M + 2H]^{2+}$, 1576.1 $[M + H]^{+}$; HRMS (ESI) *m/z*: calcd for $C_{80}H_{139}N_{18}O_{14}$ $[M + H]^{+}$ 1576.0713, found 1576.0683; calcd for $C_{80}H_{138}N_{18}O_{14}Na$ $[M + Na]^{+}$ 1598.0532, found 1598.0499.

Ac-Lys-Lys-Leu-D-Phe-Lys-Lys-Ile-Leu-Lys-Tyr-Lys(COC_3H_7)-NH_2 (BP496)

This lipopeptide was prepared following the procedure described in the manuscript using butyric acid. Acidolytic cleavage of the resulting resin and purification eluting with H_2O/CH_3CN (80:20) afforded Ac-Lys-Lys-Leu-D-Phe-Lys-Lys-Ile-Leu-Lys-Tyr-Lys(COC_3H_7)-NH_2 (**BP496**) in >99% purity. t_R = 5.11 min. MS (ESI) *m/z*: 775.1 $[M + 2H]^{2+}$, 1548.2 $[M + H]^{+}$, 1570.1 $[M + Na]^{+}$; HRMS (ESI) *m/z*: calcd for $C_{86}H_{135}N_{18}O_{14}$ $[M + H]^{+}$ 1548.0400, found 1548.0367; calcd for $C_{78}H_{134}N_{18}O_{14}Na$ $[M + Na]^{+}$ 1570.0219, found 1570.0179.

Ac-Lys-Lys-Leu-D-Phe-Lys-Lys-Ile-Leu-Lys($COC_{11}H_{23}$)-Tyr-Leu-NH_2 (BP497)

This lipopeptide was prepared following the procedure described in the manuscript using lauric acid. Acidolytic cleavage of the resulting resin and purification eluting with H_2O/CH_3CN (75:25) afforded Ac-Lys-Lys-Leu-D-Phe-Lys-Lys-Ile-Leu-Lys($COC_{11}H_{23}$)-Tyr-Leu-NH_2 (**BP497**) in >99% purity. t_R = 7.21 min. MS (ESI) *m/z*: 823.1 $[M + 2H]^{2+}$, 1645.1 $[M + H]^+$; HRMS (ESI) *m/z*: calcd for $C_{86}H_{150}N_{17}O_{14}$ $[M + H]^+$ 1645.1543, found 1645.1516; calcd for $C_{86}H_{149}N_{17}O_{14}Na$ $[M + Na]^+$ 1667.1362, found 1667.1311.

Ac-Lys-Lys-Leu-D-Phe-Lys(COC_3H_7)-Lys-Ile-Leu-Lys-Tyr-Leu-NH_2 (BP498)

This lipopeptide was prepared following the procedure described in the manuscript using butyric acid. Acidolytic cleavage of the resulting resin and purification eluting with H_2O/CH_3CN (70:30) afforded Ac-Lys-Lys-Leu-D-Phe-Lys(COC_3H_7)-Lys-Ile-Leu-Lys-Tyr-Leu-NH_2 (**BP498**) in >99% purity. t_R = 6.12 min. MS (ESI) *m/z*: 767.0 $[M + 2H]^{2+}$, 1533.1 $[M + H]^+$; HRMS (ESI) *m/z*: calcd for $C_{78}H_{135}N_{17}O_{14}$ $[M + 2H]^{2+}$ 767.0182, found 767.0147; calcd for $C_{78}H_{134}N_{17}O_{14}$ $[M + H]^+$ 1533.0291, found 1533.0225; calcd for $C_{78}H_{133}N_{17}O_{14}Na$ $[M + Na]^+$ 1555.0110, found 1555.0032.

Ac-Lys-Lys-Leu-D-Phe-Lys-Lys-Ile-Leu-Lys(COC_3H_7)-Tyr-Leu-NH_2 (BP499)

This lipopeptide was prepared following the procedure described in the manuscript using butyric acid. Acidolytic cleavage of the resulting resin and purification eluting with H_2O/CH_3CN (75:25) afforded Ac-Lys-Lys-Leu-D-Phe-Lys-Lys-Ile-Leu-Lys(COC_3H_7)-Tyr-Leu-NH_2 (**BP499**) in >99% purity. t_R = 5.89 min. MS (ESI) *m/z*: 766.8 $[M + 2H]^{2+}$, 1533.4 $[M + H]^+$, 1555.1 $[M + Na]^+$; HRMS (ESI) *m/z*: : calcd for $C_{78}H_{134}N_{17}O_{14}$ $[M + H]^+$ 1533.0291, found 1533.0269; calcd for $C_{78}H_{133}N_{17}O_{14}Na$ $[M + Na]^+$ 1555.0110, found 1555.0097.

Ac-Lys-Lys-Lys($COC_{11}H_{23}$)-D-Phe-Lys-Lys-Ile-Leu-Lys-Tyr-Leu-NH_2 (BP500)

This lipopeptide was prepared following the procedure described in the manuscript using lauric acid. Acidolytic cleavage of the resulting resin and purification eluting with H_2O/CH_3CN (70:30) afforded Ac-Lys-Lys-Lys($COC_{11}H_{23}$)-D-Phe-Lys-Lys-Ile-Leu-Lys-Tyr-Leu-NH_2 (**BP500**) in >99% purity. t_R

= 6.80 min. MS (ESI) *m/z*: 554.4 $[M + 3H]^{3+}$, 830.7 $[M + 2H]^{2+}$; HRMS (ESI) *m/z*: calcd for $C_{86}H_{151}N_{18}O_{14}$ $[M + H]^+$ 1661.1684, found 1661.1667; calcd for $C_{86}H_{150}N_{18}O_{14}Na$ $[M + Na]^+$ 1683.1503, found 1683.1487.

3. HPLC of crude and purified lipopeptides, ESI-MS and HRMS of purified lipopeptides

$C_5H_{11}CO$-Lys-Lys-Leu-D-Phe-Lys-Lys-Ile-Leu-Lys-Tyr-Leu-NH_2 (BP472)

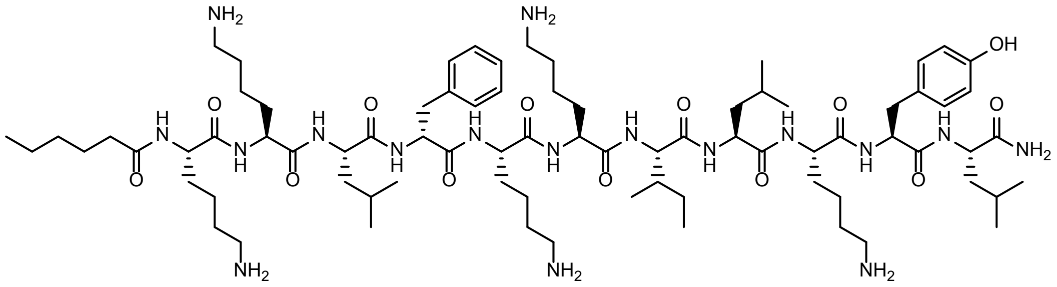

HPLC of crude peptide (λ=220 nm)

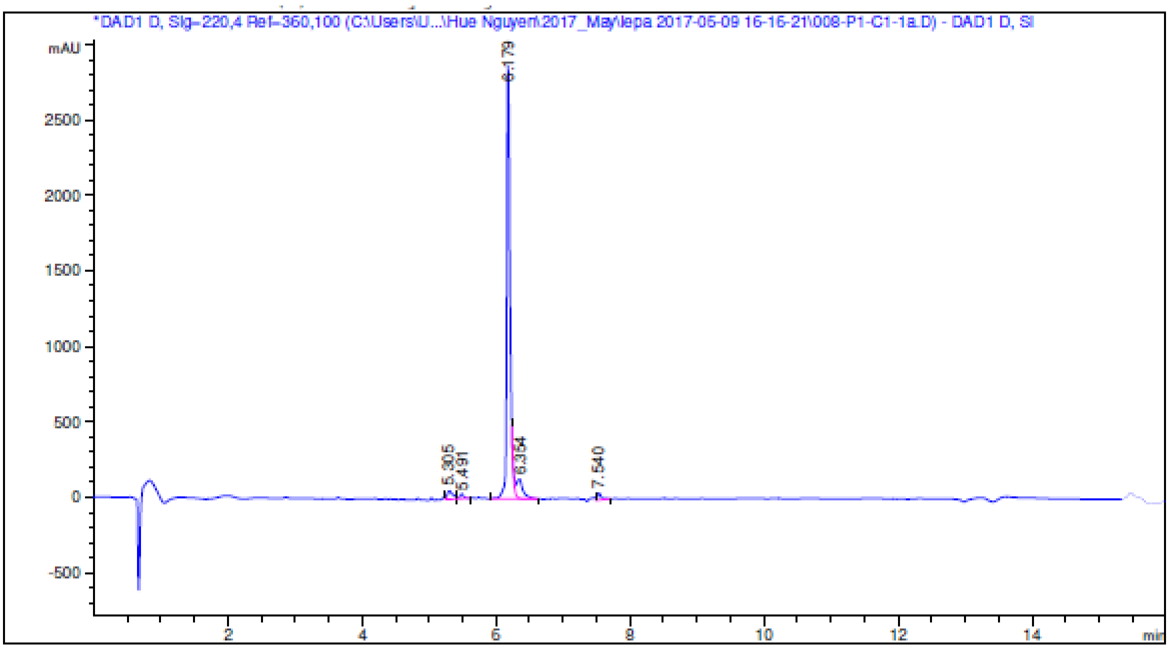

Peak #	RetTime [min]	Type	Width [min]	Area [mAU*s]	Height [mAU]	Area %
1	5.305	VV	0.0871	334.45099	54.91665	2.8671
2	5.491	VB	0.0577	135.30244	32.39536	1.1599
3	6.179	BV R	0.0529	1.00572e4	2871.68335	86.2160
4	6.354	VB E	0.0962	953.29431	126.04663	8.1722
5	7.540	VV R	0.0572	184.87265	46.71790	1.5848

HPLC of purified peptide (λ=220 nm)

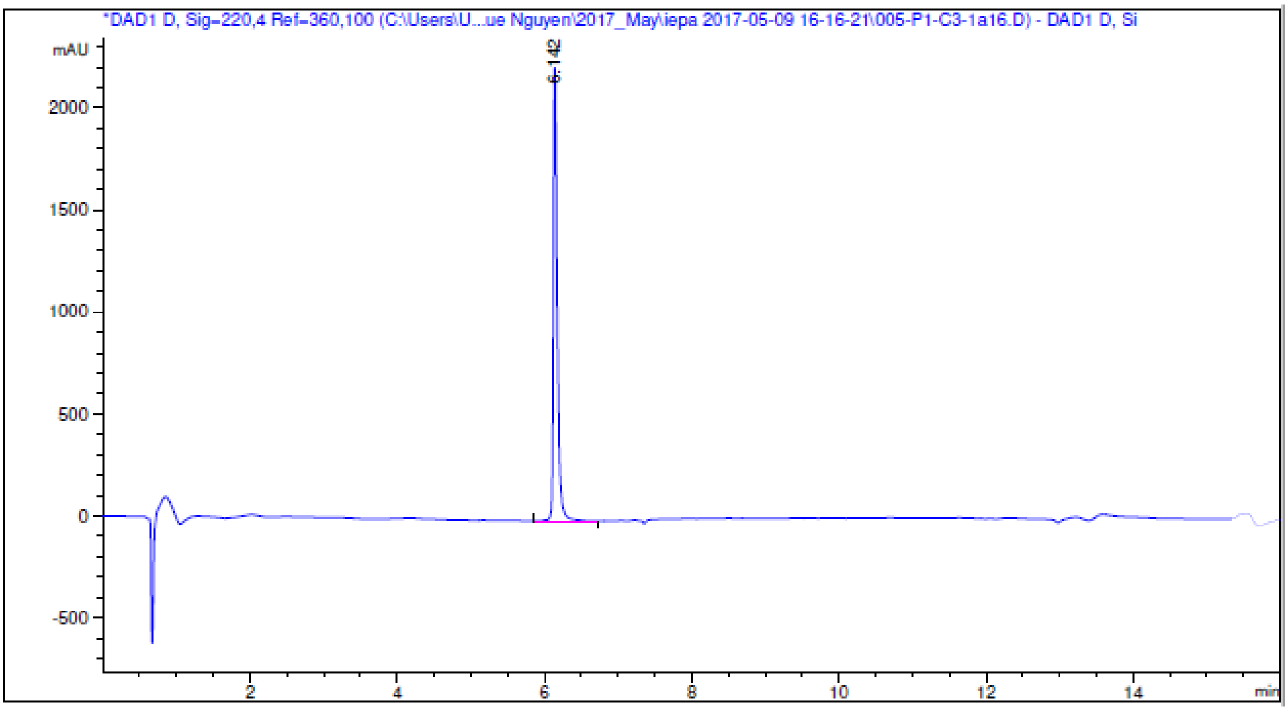

Peak #	RetTime [min]	Type	Width [min]	Area [mAU*s]	Height [mAU]	Area %
1	6.142	BB	0.0530	8007.58594	2222.06152	100.0000

ESI-MS (*m/z*)

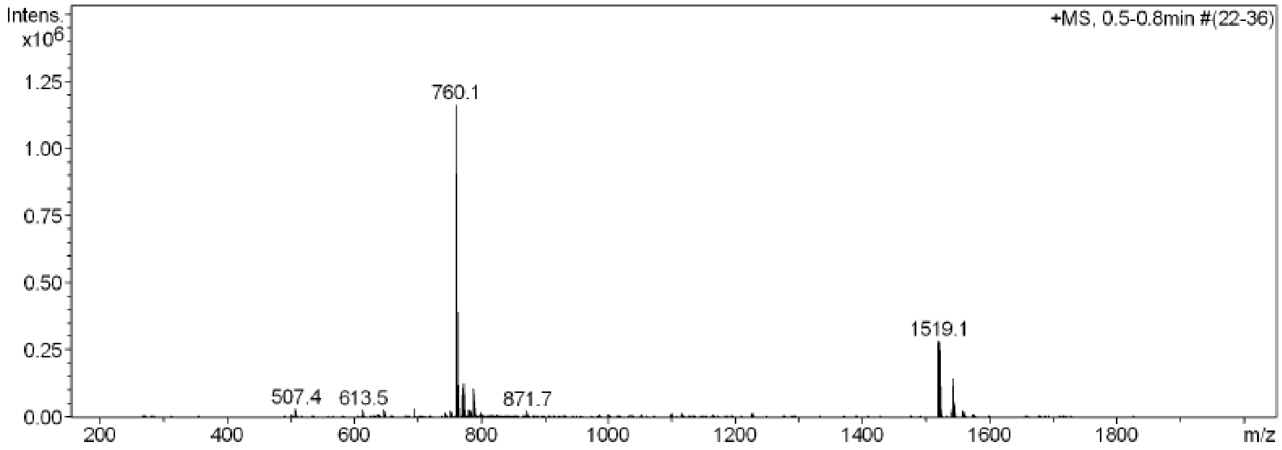

HRMS (*m/z*)

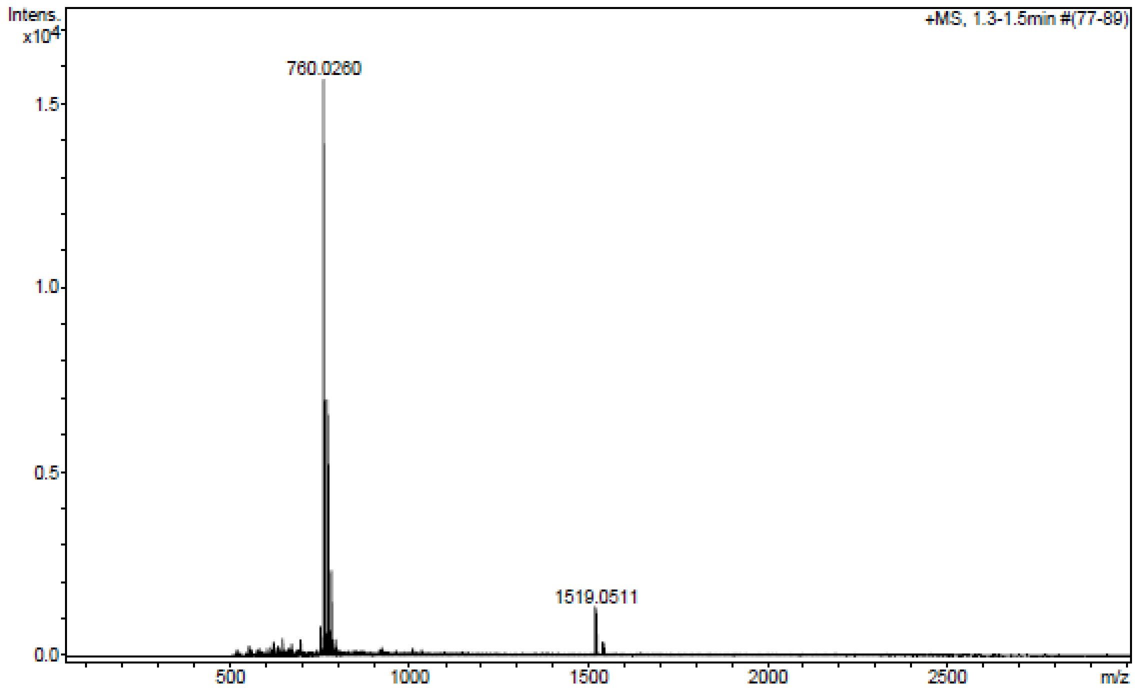

Intens.
x10^4
+MS, 1.3-1.5min #(77-89)
760.0260
1519.0511
1.5
1.0
0.5
0.0
500
1000
1500
2000
2500
m/z

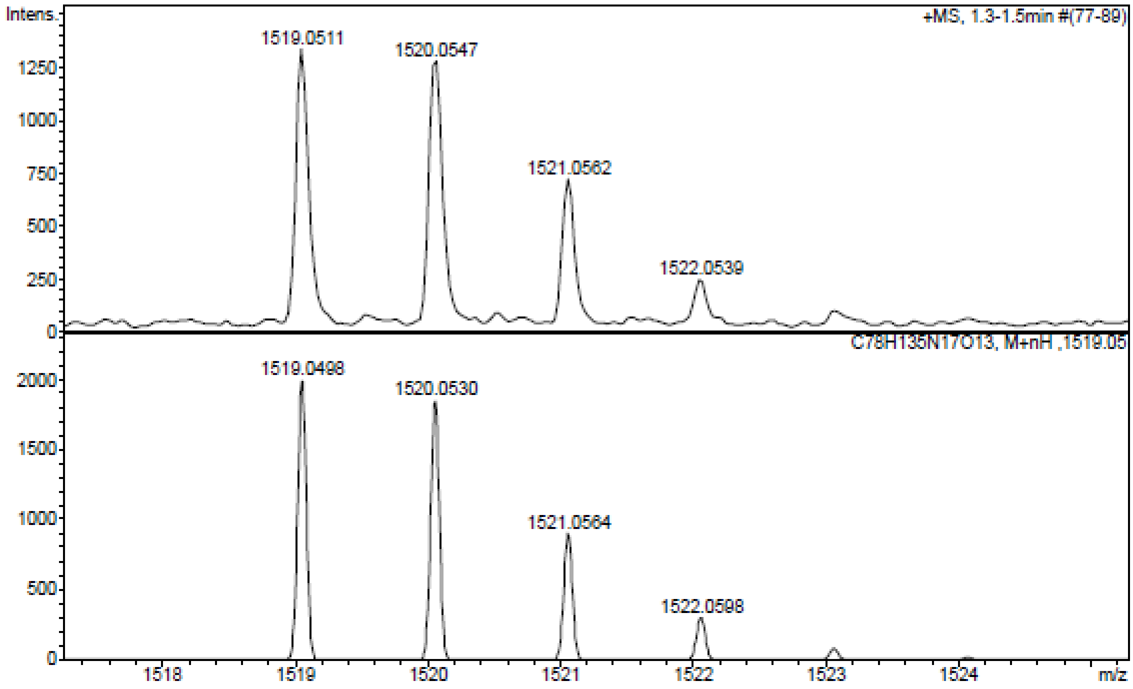

Intens.
+MS, 1.3-1.5min #(77-89)
1519.0511
1520.0547
1521.0562
1522.0539
C78H135N17O13, M+nH ,1519.05
1519.0498
1520.0530
1521.0564
1522.0598
1250
1000
750
500
250
0
2000
1500
1000
500
0
1518
1519
1520
1521
1522
1523
1524
m/z

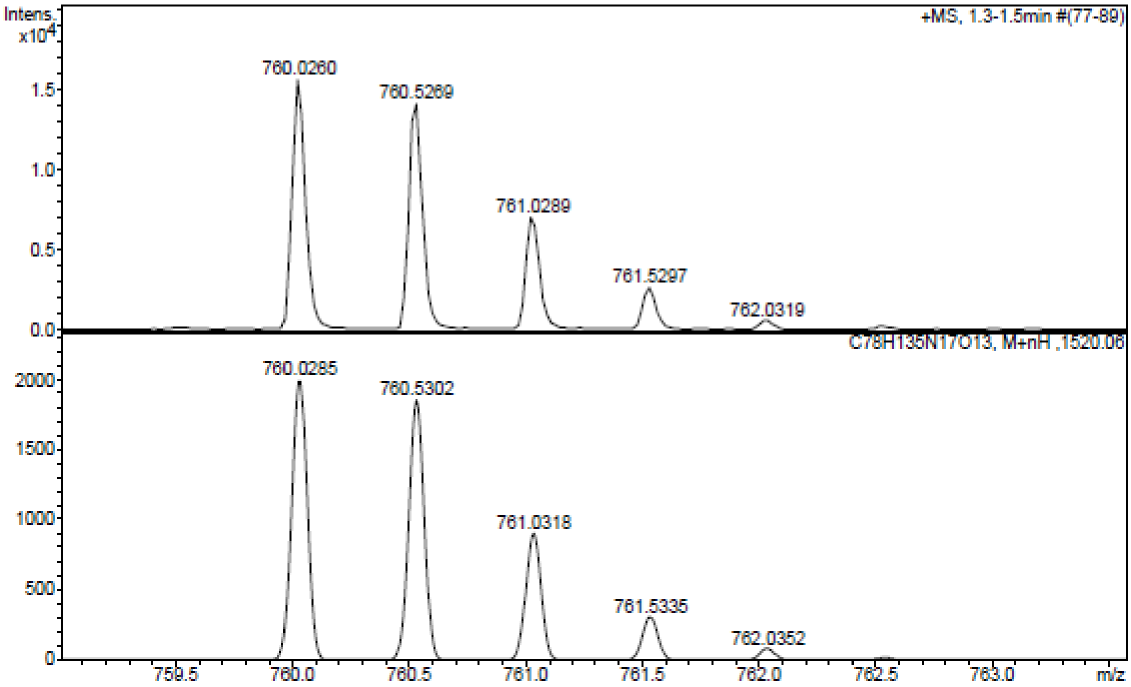
Intens.
x10^4
+MS, 1.3-1.5min #(77-89)
760.0260
760.5269
761.0289
761.5297
762.0319
C78H135N17O13, M+nH ,1520.06
760.0285
760.5302
761.0318
761.5335
762.0352
759.5
760.0
760.5
761.0
761.5
762.0
762.5
763.0
m/z

Ac-Lys-Lys-Leu-D-Phe-Lys-Lys(COC_3H_7)-Ile-Leu-Lys-Tyr-Leu-NH_2 (BP473)

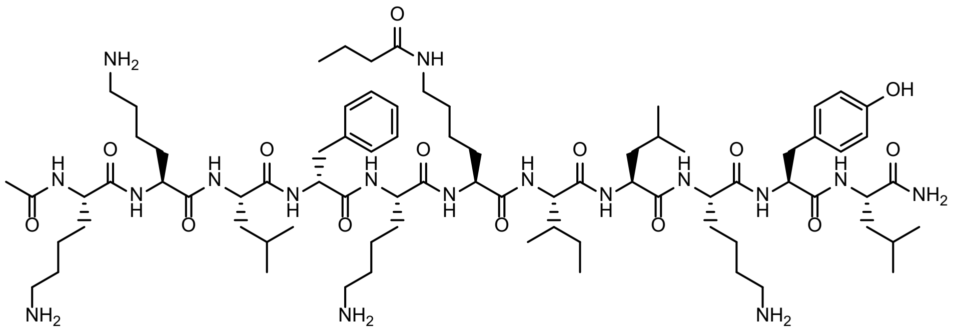

HPLC of crude peptide (λ=220 nm)

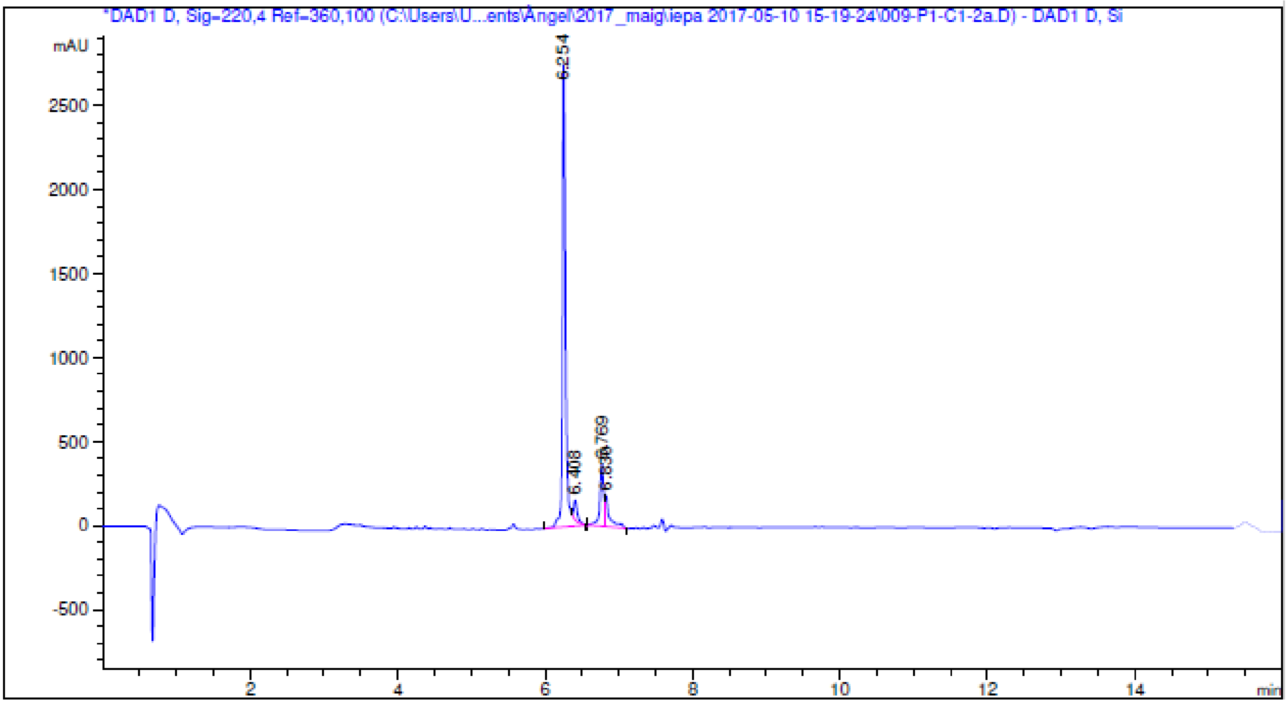

Peak #	RetTime [min]	Type	Width [min]	Area [mAU*s]	Height [mAU]	Area %
1	6.254	BV R	0.0464	8589.29297	2749.92847	77.4871
2	6.408	VB E	0.0496	383.71664	115.83309	3.4616
3	6.769	BV	0.0511	1300.70288	369.00027	11.7341
4	6.830	VV R	0.0589	811.08887	182.18036	7.3171

HPLC of purified peptide (λ=220 nm)

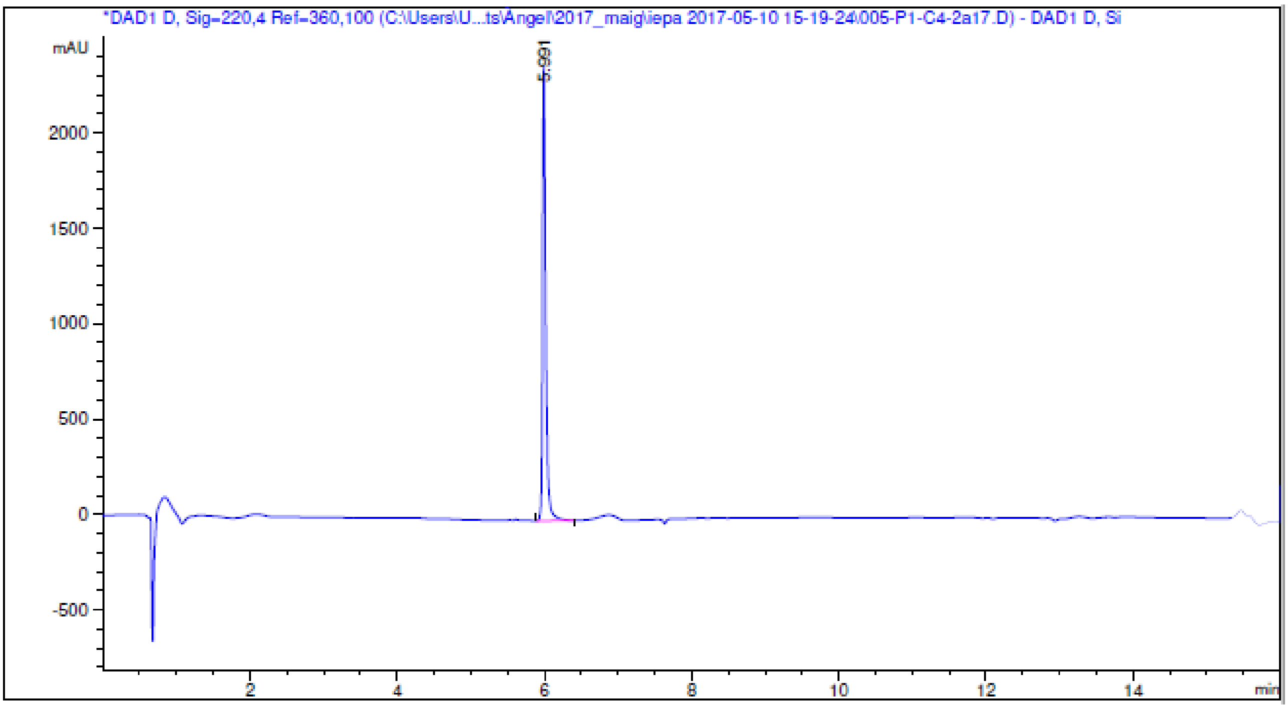

Peak #	RetTime [min]	Type	Width [min]	Area [mAU*s]	Height [mAU]	Area %
1	5.991	BB	0.0488	7708.68848	2381.31323	100.0000

ESI-MS (*m/z*)

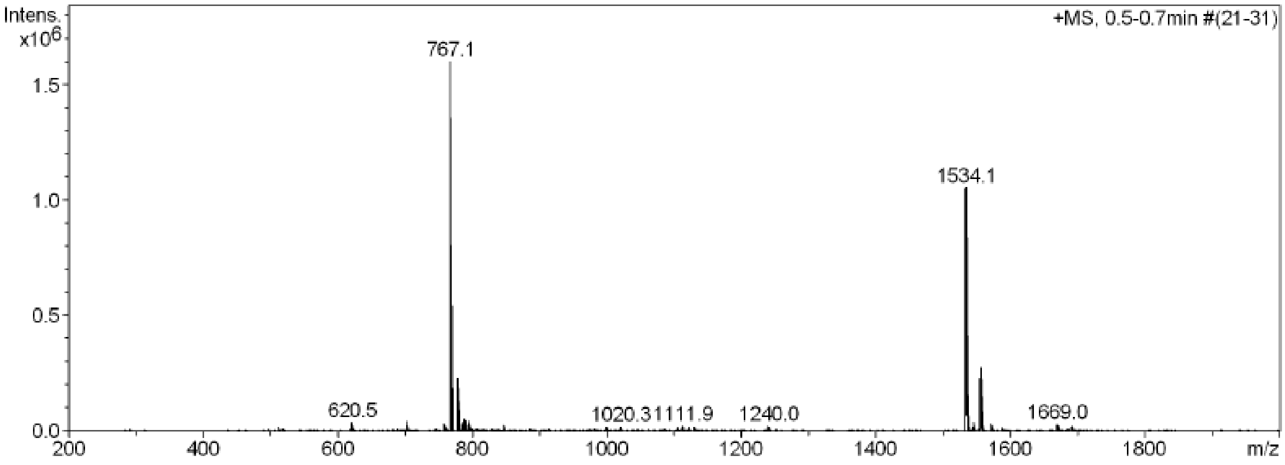

HRMS (m/z)

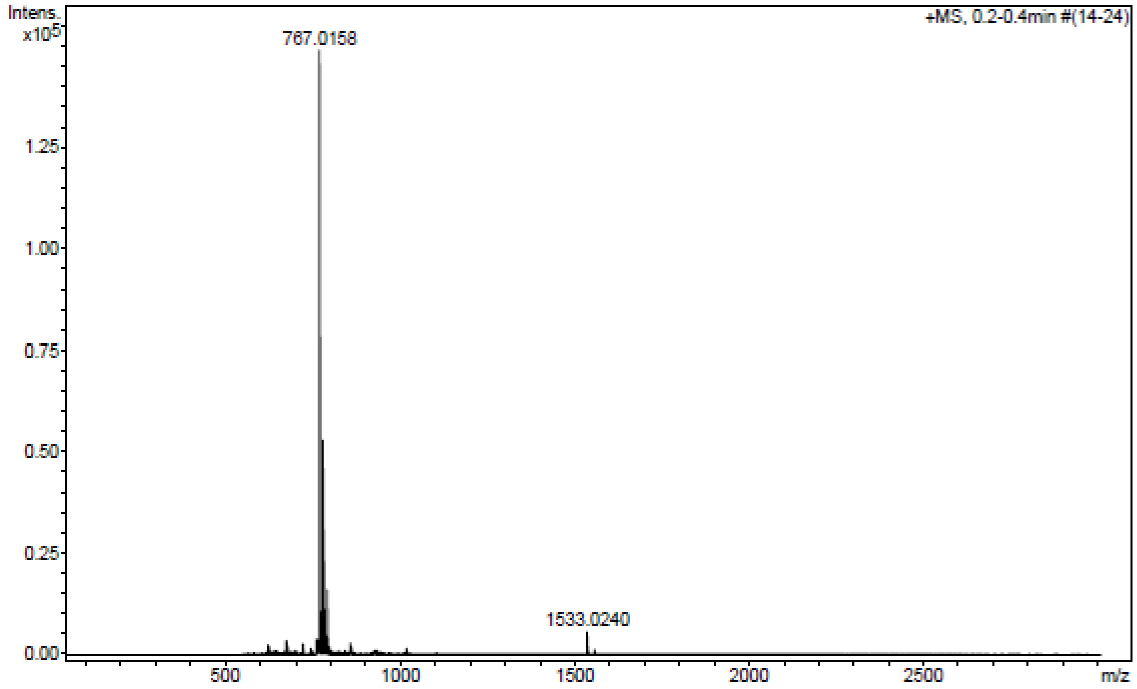

Intens.
x10^5
+MS, 0.2-0.4min #(14-24)
767.0158
1533.0240
1.25
1.00
0.75
0.50
0.25
0.00
500
1000
1500
2000
2500
m/z

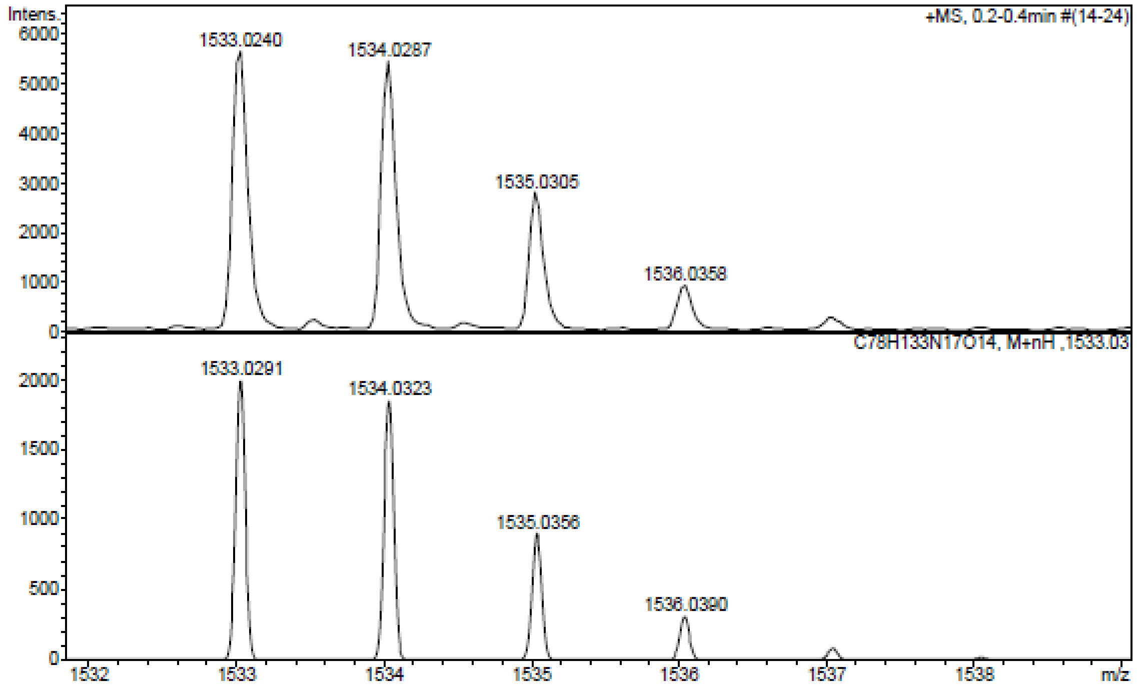

Intens.
+MS, 0.2-0.4min #(14-24)
1533.0240
1534.0287
1535.0305
1536.0358
C78H133N17O14, M+nH ,1533.03
1533.0291
1534.0323
1535.0356
1536.0390
1532
1533
1534
1535
1536
1537
1538
m/z

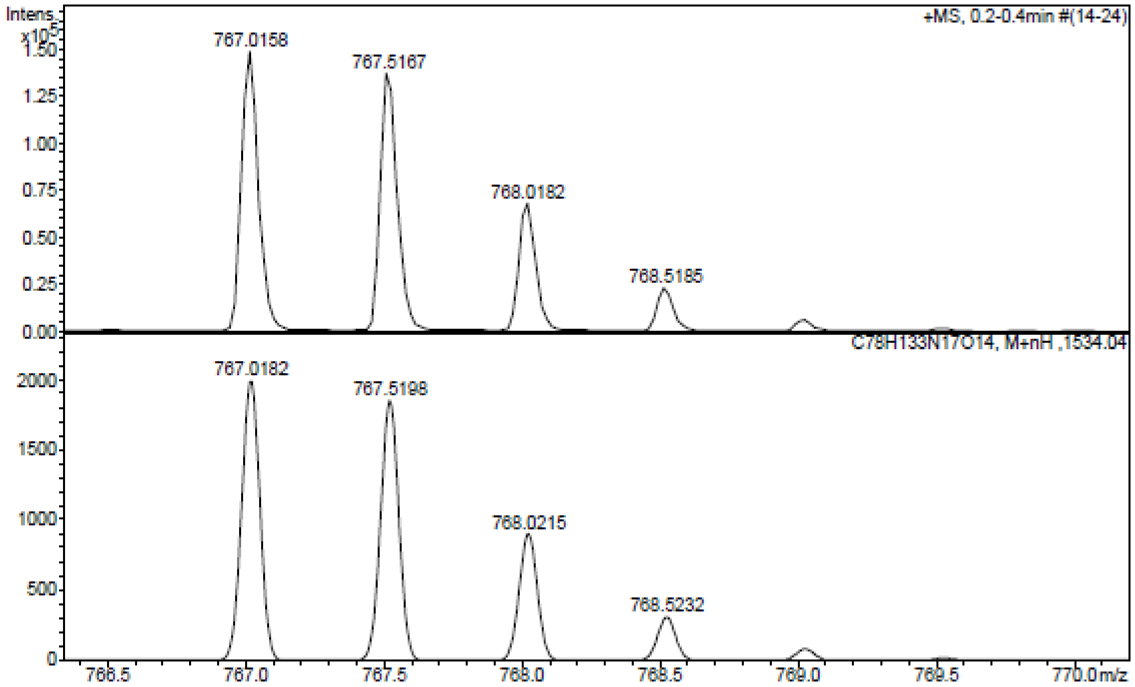
Intens.
x10^5
+MS, 0.2-0.4min #(14-24)
767.0158
767.5167
768.0182
768.5185
C78H133N17O14, M+nH ,1534.04
767.0182
767.5198
768.0215
768.5232
m/z

Ac-Lys-Lys-Leu-D-Phe-Lys-Lys-Ile-Lys(COC$_3$H$_7$)-Lys-Tyr-Leu-NH$_2$ (BP474)

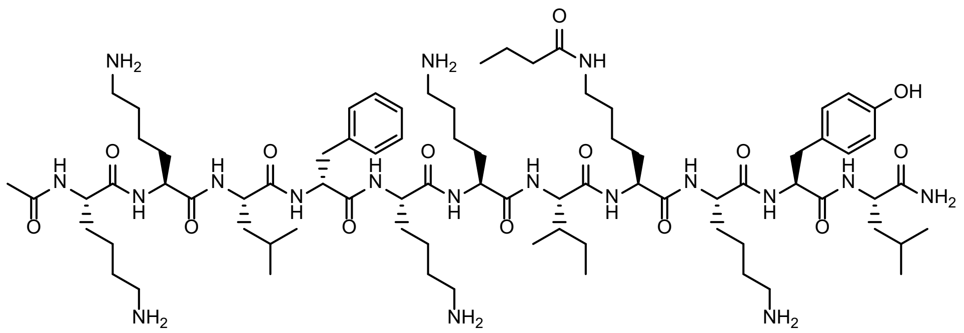

HPLC of crude peptide (λ=220 nm)

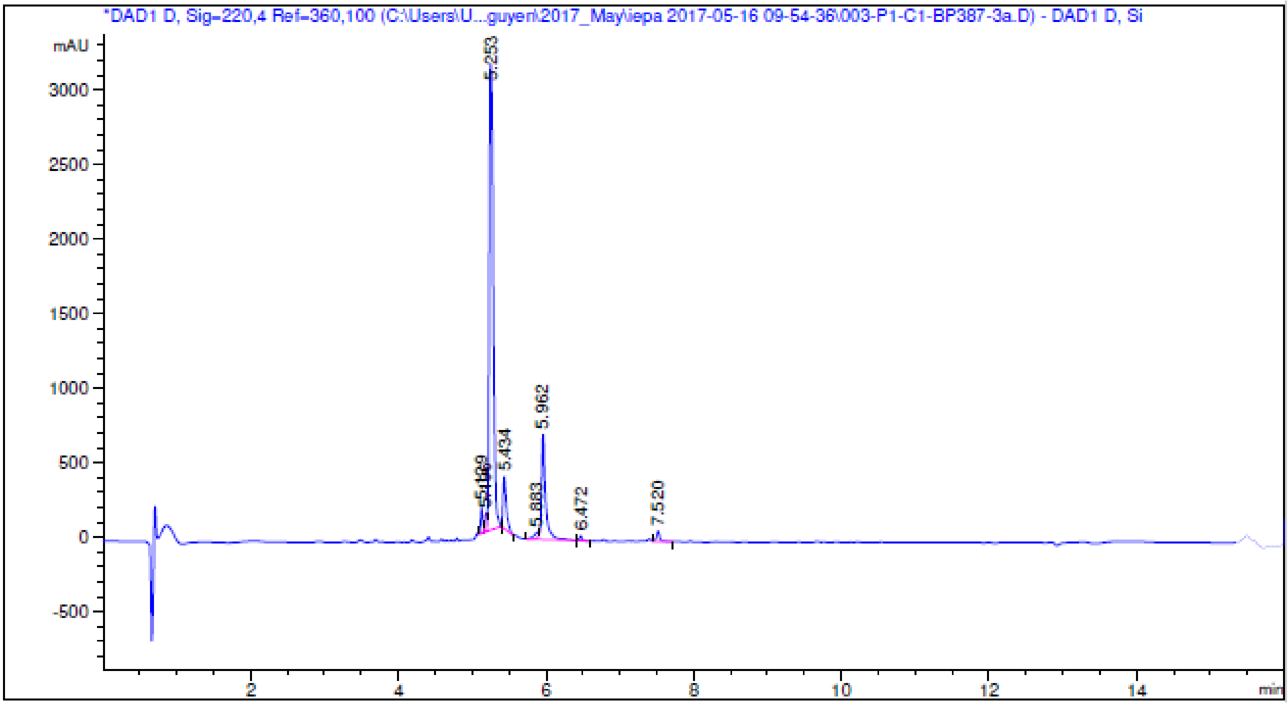

Peak #	RetTime [min]	Type	Width [min]	Area [mAU*s]	Height [mAU]	Area %
1	5.129	BV E	0.0323	377.93021	181.34412	2.2820
2	5.196	VV E	0.0268	212.25146	119.26482	1.2816
3	5.253	VB R	0.0595	1.16522e4	3125.37207	70.3588
4	5.434	BB	0.0460	1109.19092	349.34921	6.6976
5	5.883	BV E	0.0659	192.02771	38.04109	1.1595
6	5.962	VV R	0.0532	2679.02954	707.11737	16.1766
7	6.472	BB	0.0410	95.51791	34.79841	0.5768
8	7.520	VB R	0.0507	242.96144	71.34217	1.4671

HPLC of purified peptide (λ=220 nm)

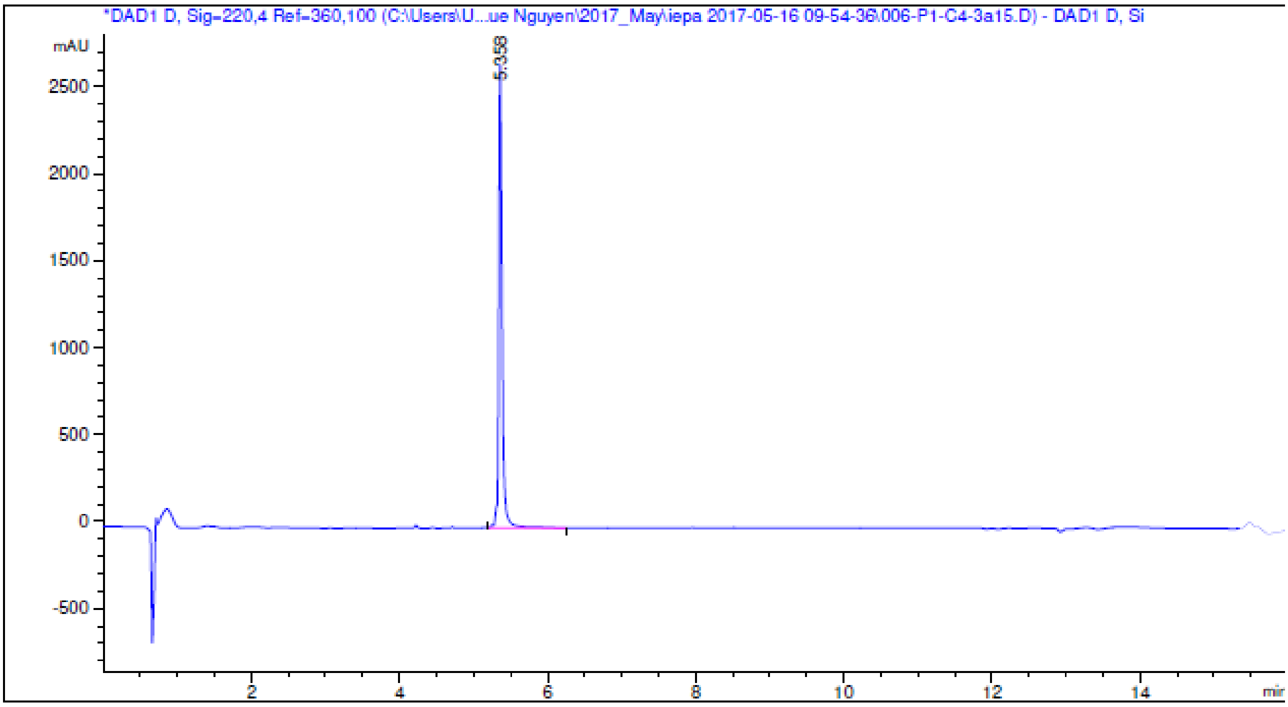

Peak #	RetTime [min]	Type	Width [min]	Area [mAU*s]	Height [mAU]	Area %
1	5.358	VB R	0.0460	8253.83496	2673.69556	100.0000

ESI-MS (*m/z*)

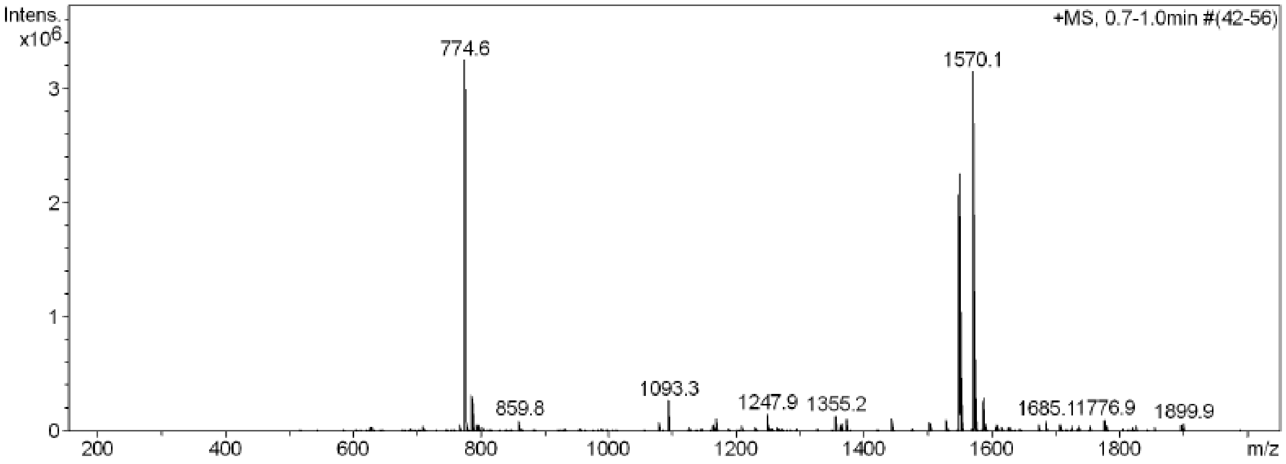

HRMS (*m/z*)

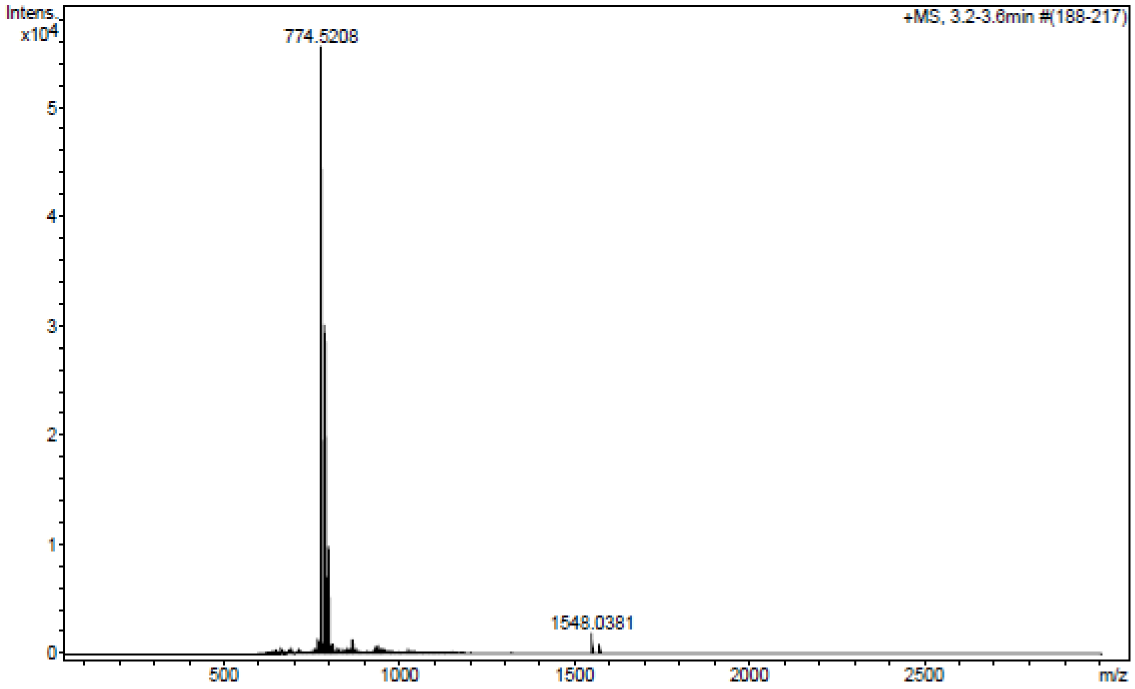

Intens.
x10^4
+MS, 3.2-3.6min #(188-217)
774.5208
1548.0381
5
4
3
2
1
0
500
1000
1500
2000
2500
m/z

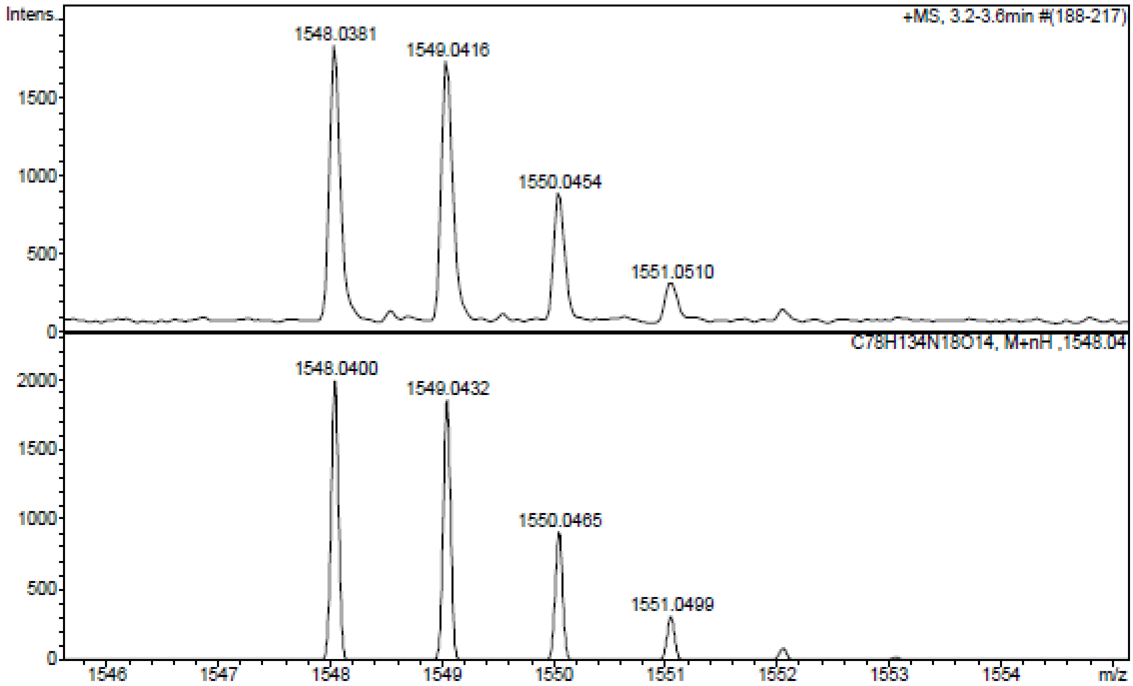

Intens.
+MS, 3.2-3.6min #(188-217)
1548.0381
1549.0416
1550.0454
1551.0510
1500
1000
500
0
C78H134N18O14, M+nH ,1548.04
1548.0400
1549.0432
1550.0465
1551.0499
2000
1500
1000
500
0
1546
1547
1548
1549
1550
1551
1552
1553
1554
m/z

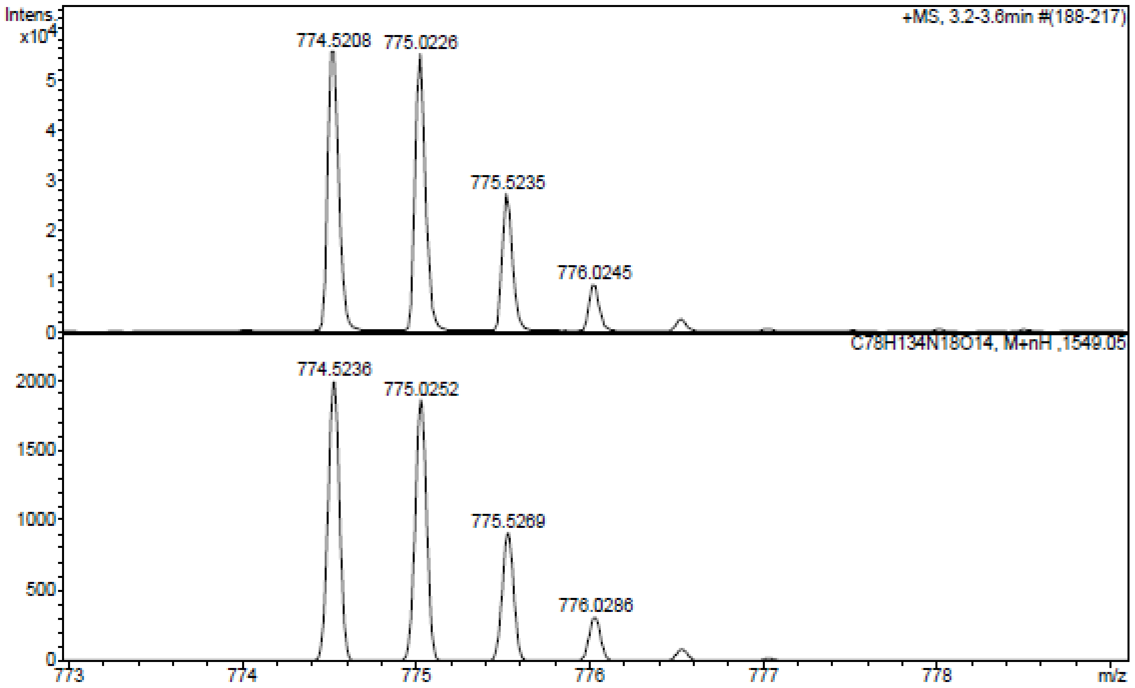

Intens.
x10^4
+MS, 3.2-3.6min #(188-217)
774.5208
775.0226
775.5235
776.0245
C78H134N18O14, M+nH ,1549.05
774.5236
775.0252
775.5269
776.0286
m/z

Ac-Lys-Lys-Leu-D-Phe-Lys-Lys-Ile-Leu-Lys-Lys(COC_3H_7)-Leu-NH_2 (BP475)

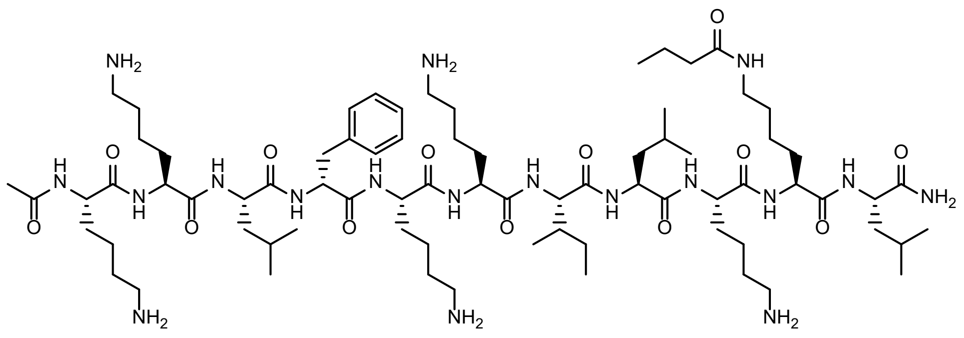

HPLC of crude peptide (λ=220 nm)

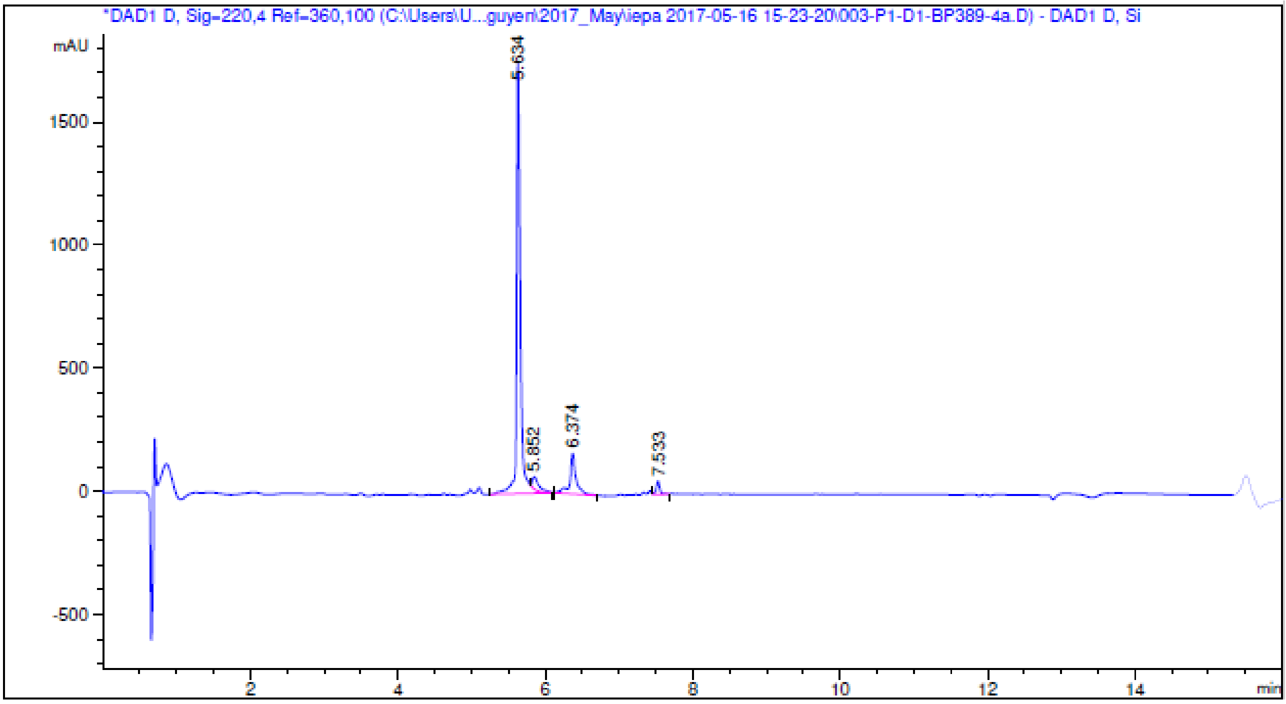

Peak #	RetTime [min]	Type	Width [min]	Area [mAU*s]	Height [mAU]	Area %
1	5.634	VV R	0.0514	6206.26611	1748.02429	81.1357
2	5.852	VB E	0.0914	327.03192	50.62853	4.2753
3	6.374	VB R	0.0767	938.12811	166.53392	12.2643
4	7.533	VV R	0.0512	177.81927	52.87458	2.3247

HPLC of purified peptide (λ=220 nm)

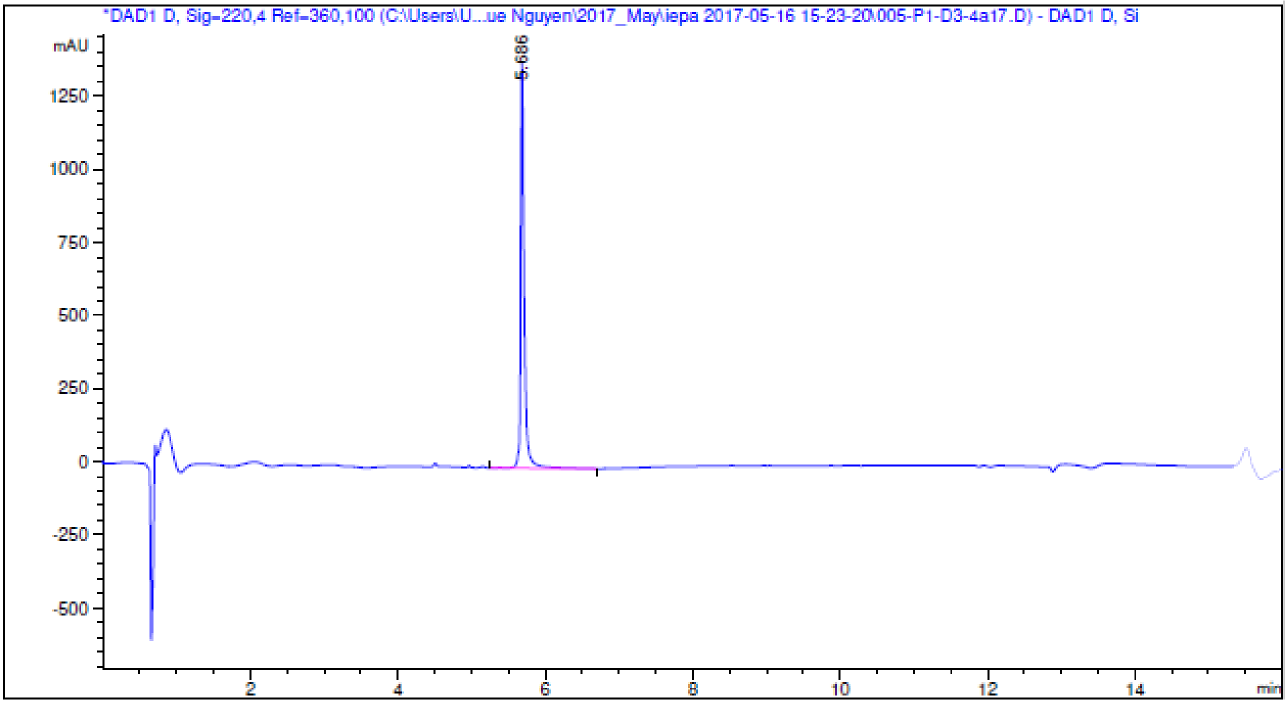

Peak #	RetTime [min]	Type	Width [min]	Area [mAU*s]	Height [mAU]	Area %
1	5.686	VB R	0.0485	4586.54346	1388.06421	100.0000

ESI-MS (*m/z*)

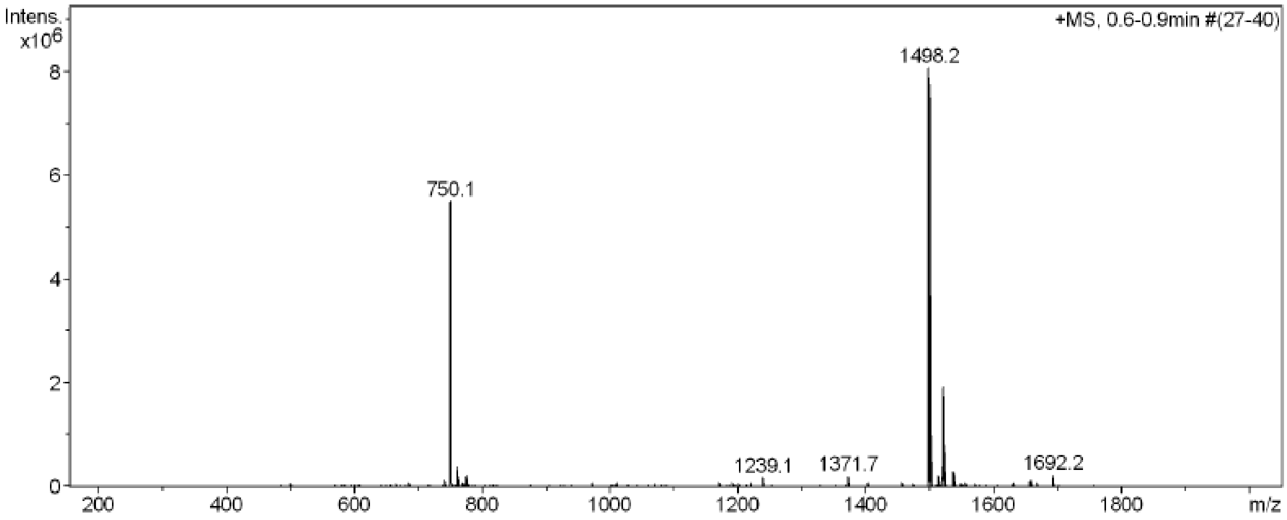

HRMS (*m/z*)

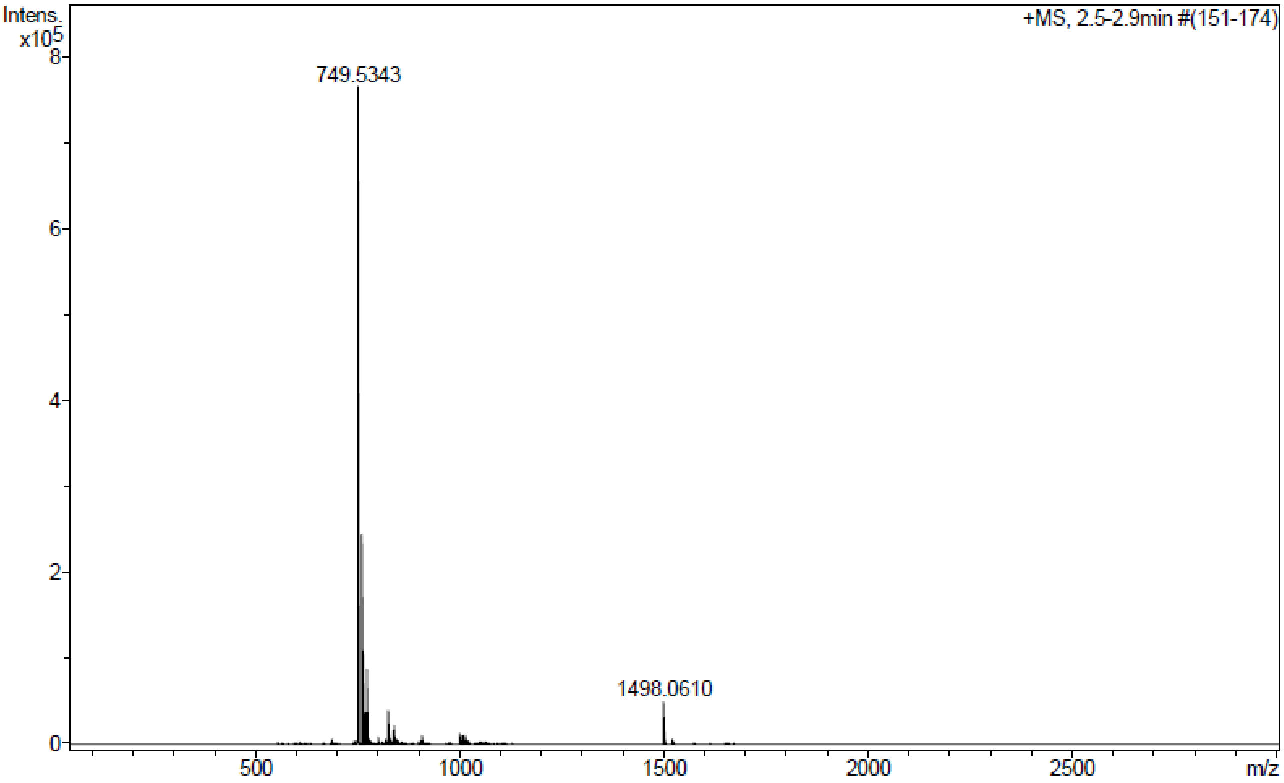

Intens.
x10^5
+MS, 2.5-2.9min #(151-174)
749.5343
1498.0610
m/z

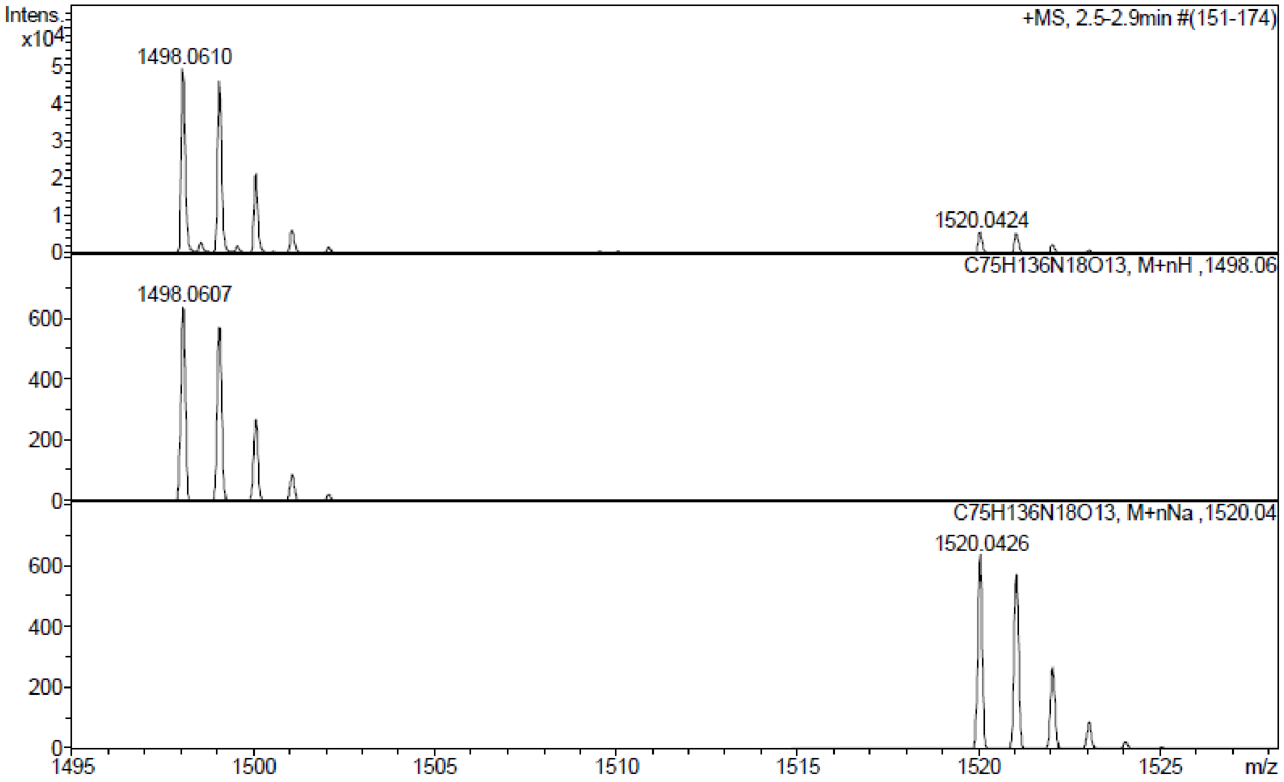

Intens.
x10^4
+MS, 2.5-2.9min #(151-174)
1498.0610
1520.0424
C75H136N18O13, M+nH ,1498.06
1498.0607
C75H136N18O13, M+nNa ,1520.04
1520.0426
m/z

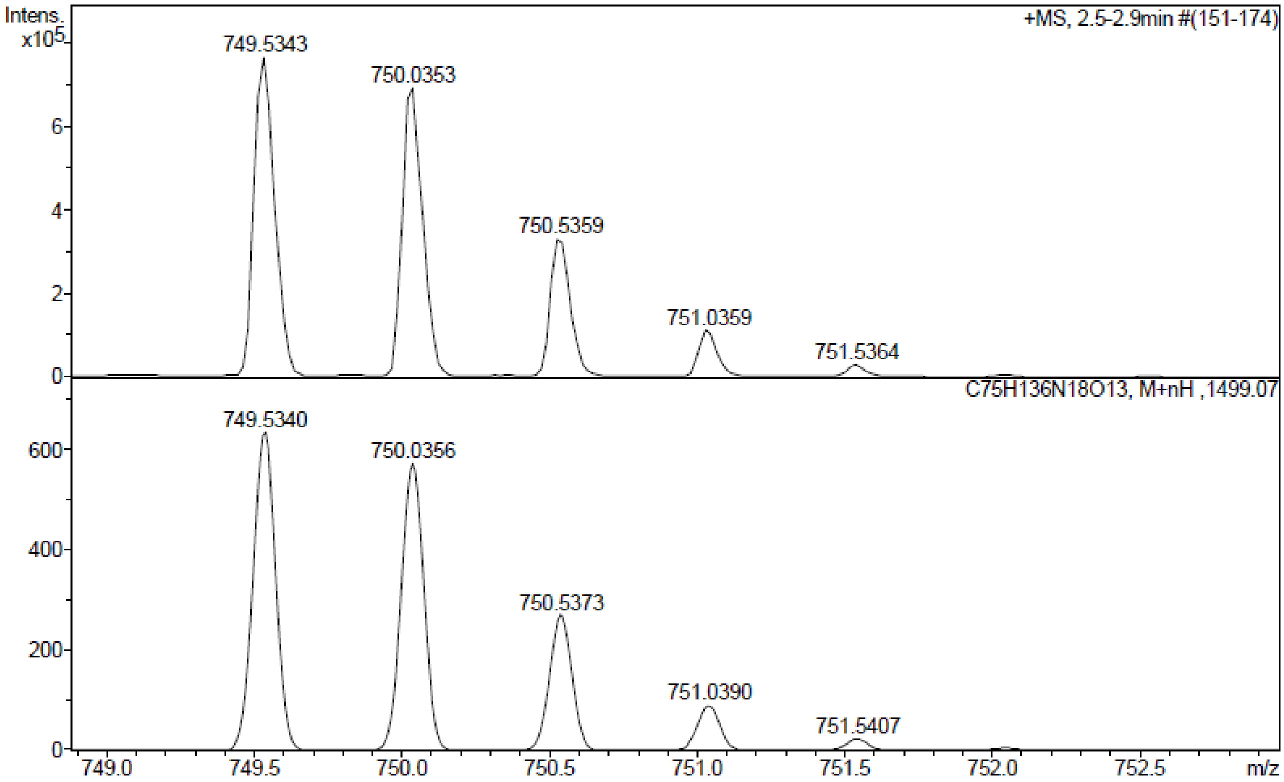

Intens.
x10^5
+MS, 2.5-2.9min #(151-174)
749.5343
750.0353
750.5359
751.0359
751.5364
6
4
2
0
C75H136N18O13, M+nH ,1499.07
749.5340
750.0356
750.5373
751.0390
751.5407
600
400
200
0
749.0
749.5
750.0
750.5
751.0
751.5
752.0
752.5
m/z

Ac-Lys-Lys-Leu-D-Phe-Lys-Lys-Ile-Leu-Lys-Tyr-Lys($COC_{11}H_{23}$)-NH_2 (BP476)

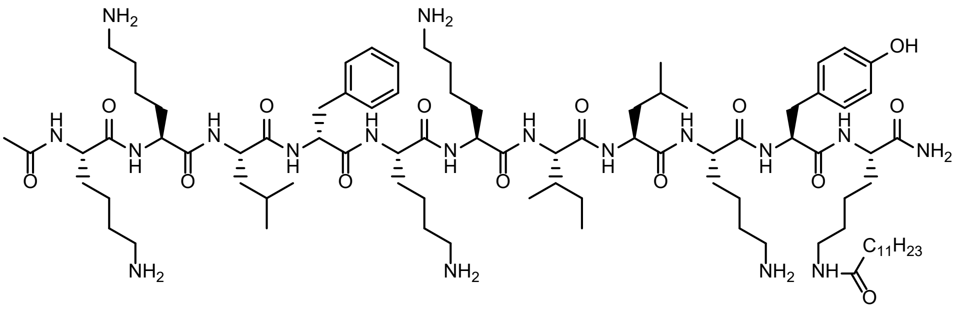

HPLC of crude peptide (λ=220 nm)

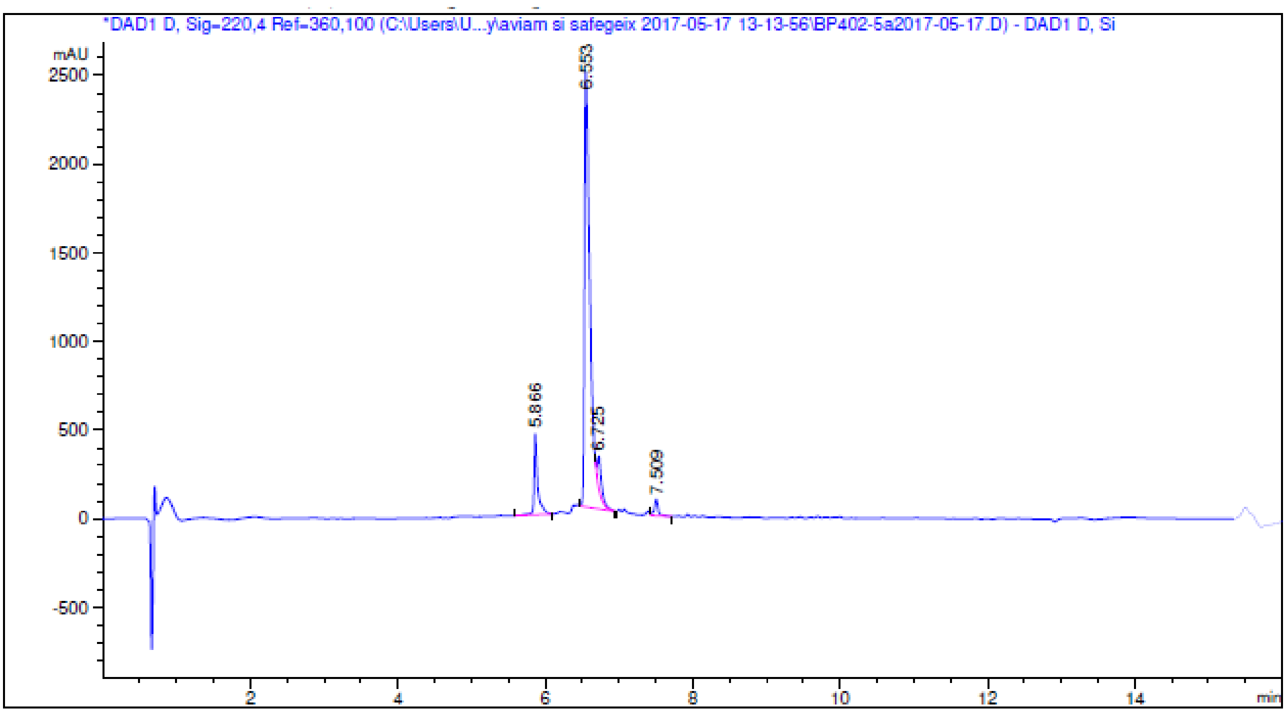

Peak #	RetTime [min]	Type	Width [min]	Area [mAU*s]	Height [mAU]	Area %
1	5.866	VB R	0.0513	1669.44141	460.88354	10.5820
2	6.553	VV R	0.0791	1.30655e4	2452.72852	82.8174
3	6.725	VB E	0.0570	698.40192	177.29459	4.4269
4	7.509	VB R	0.0537	342.92166	93.65472	2.1737

HPLC of purified peptide (λ=220 nm)

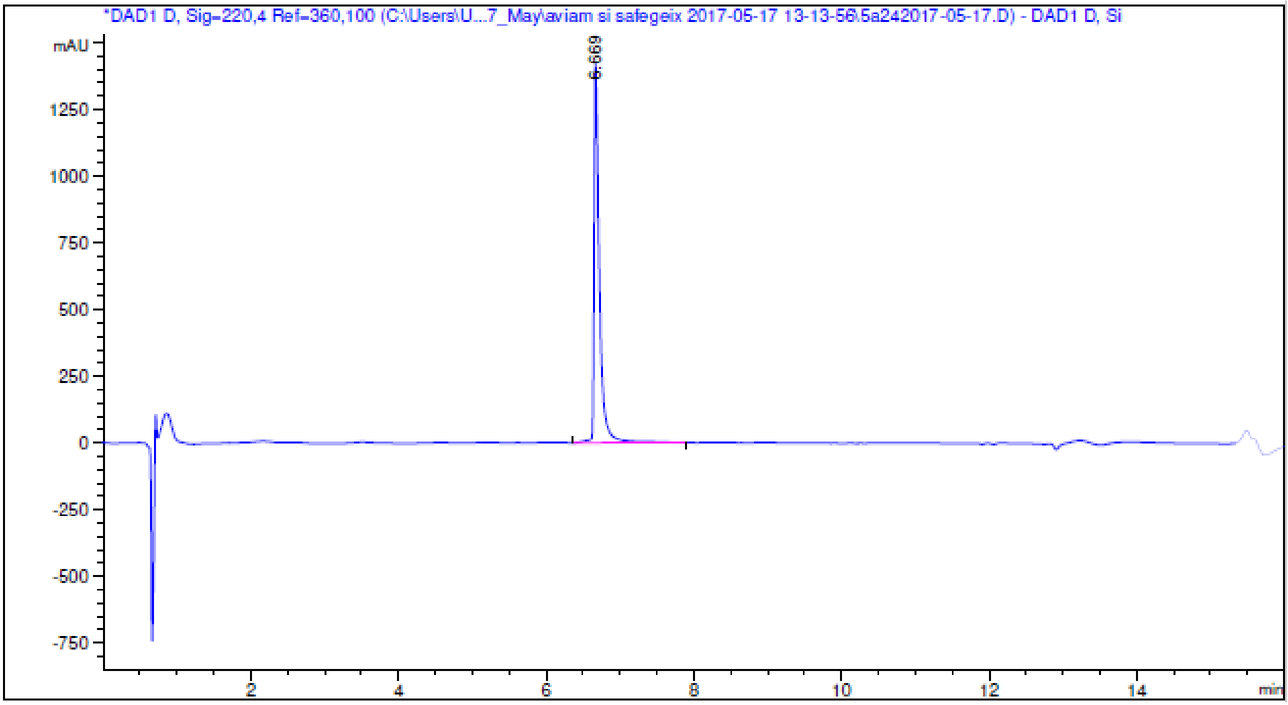

Peak #	RetTime [min]	Type	Width [min]	Area [mAU*s]	Height [mAU]	Area %
1	6.669	BB	0.0692	6972.58691	1421.61267	100.0000

ESI-MS (*m/z*)

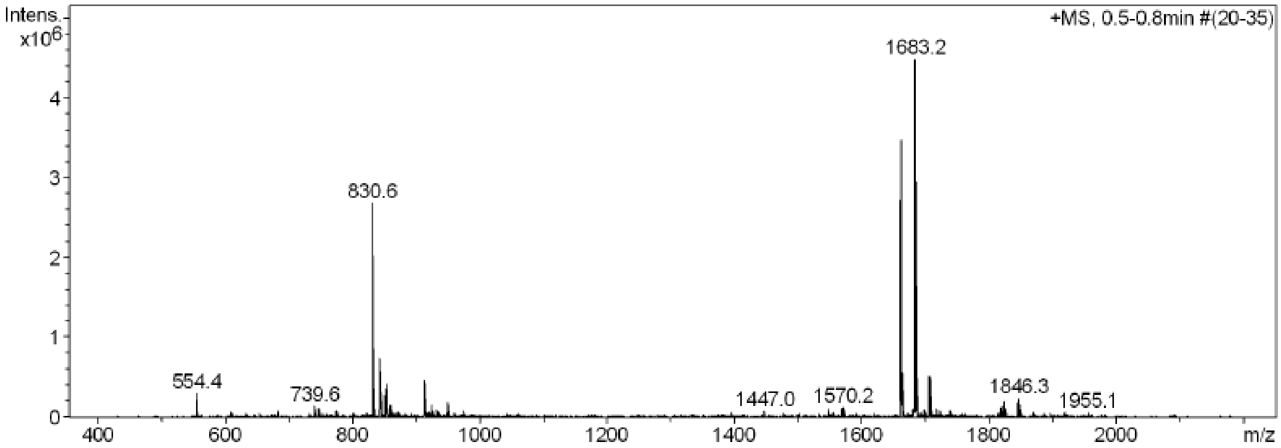

HRMS (*m/z*)

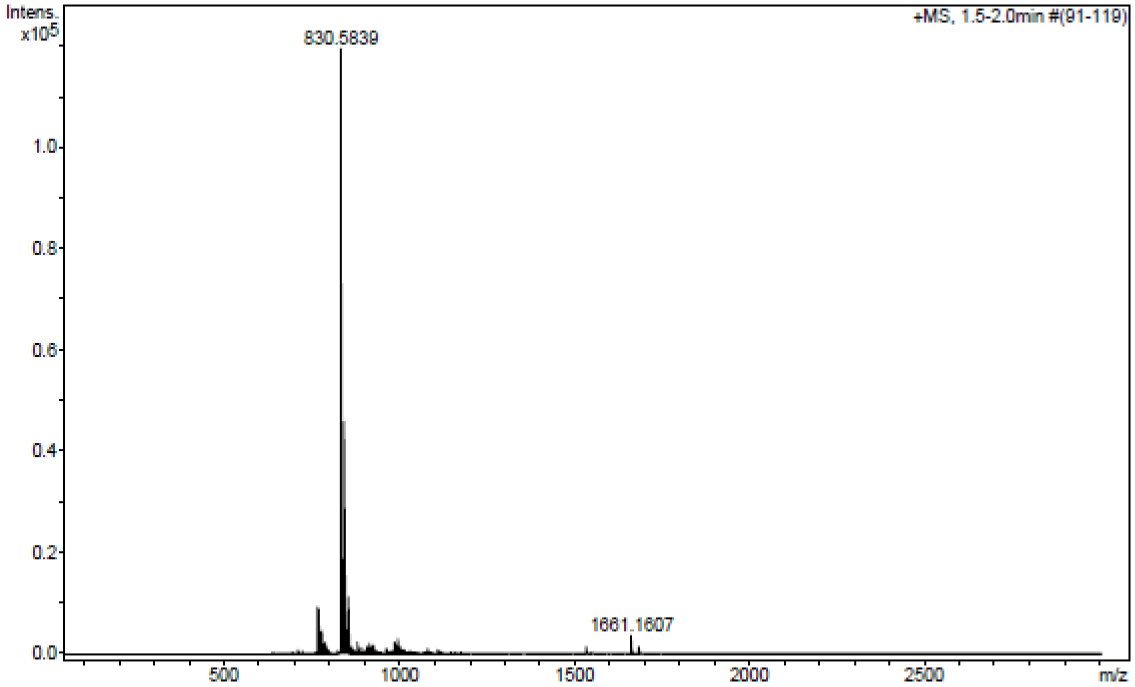

Intens.
x10^5
+MS, 1.5-2.0min #(91-119)
830.5839
1661.1607
1.0
0.8
0.6
0.4
0.2
0.0
500
1000
1500
2000
2500
m/z

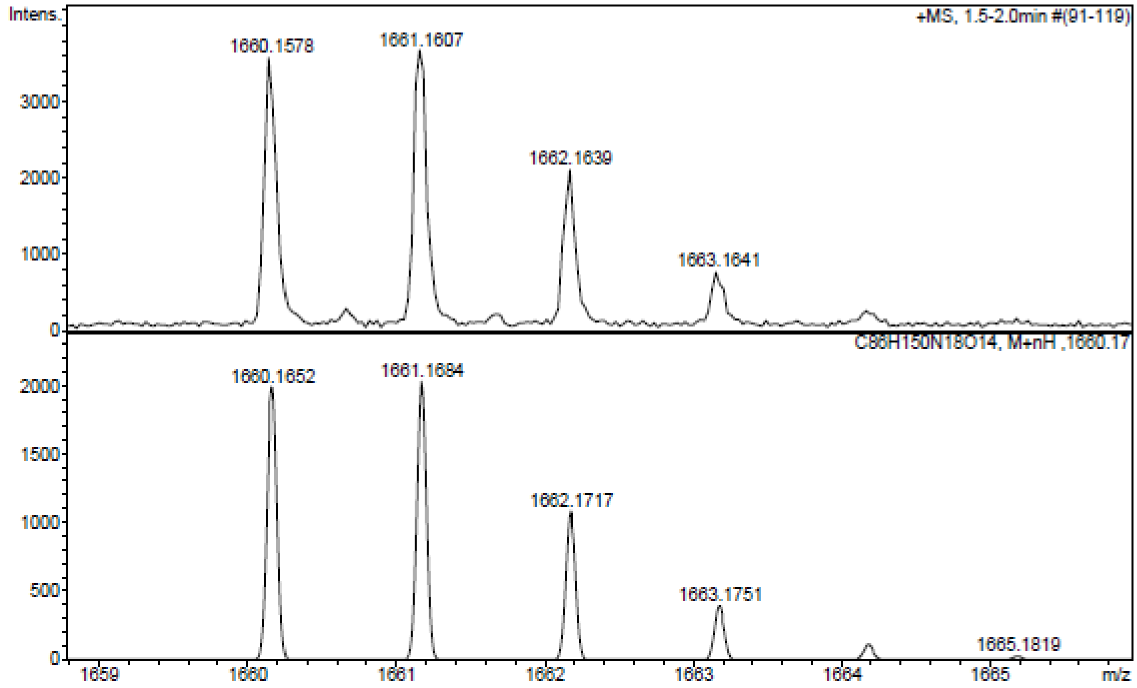

Intens.
+MS, 1.5-2.0min #(91-119)
1660.1578
1661.1607
1662.1639
1663.1641
3000
2000
1000
0
C86H150N18O14, M+nH ,1660.17
1660.1652
1661.1684
1662.1717
1663.1751
1665.1819
2000
1500
1000
500
0
1659
1660
1661
1662
1663
1664
1665
m/z

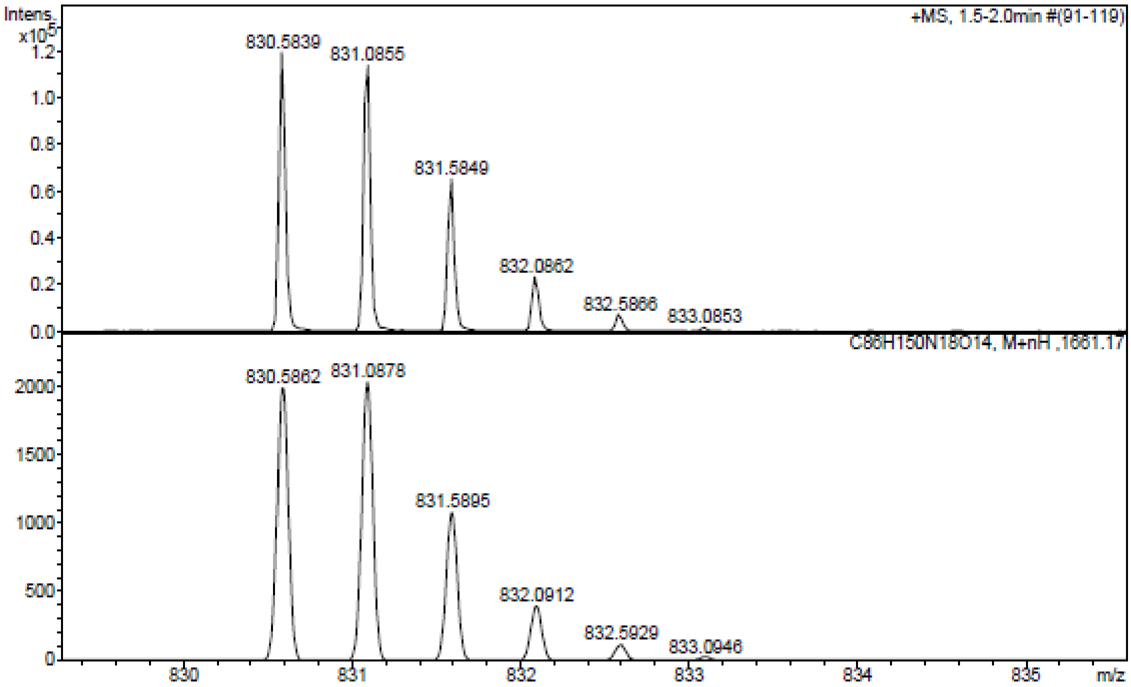
Intens.
x10^5
1.2
1.0
0.8
0.6
0.4
0.2
0.0
+MS, 1.5-2.0min #(91-119)
830.5839
831.0855
831.5849
832.0862
832.5866
833.0853
C86H150N18O14, M+nH ,1661.17
2000
1500
1000
500
0
830.5862
831.0878
831.5895
832.0912
832.5929
833.0946
830
831
832
833
834
835
m/z

Ac-Lys-Lys-Leu-D-Lys(COC_5H_{11})-Lys-Lys-Ile-Leu-Lys-Tyr-Leu-NH_2 (BP484)

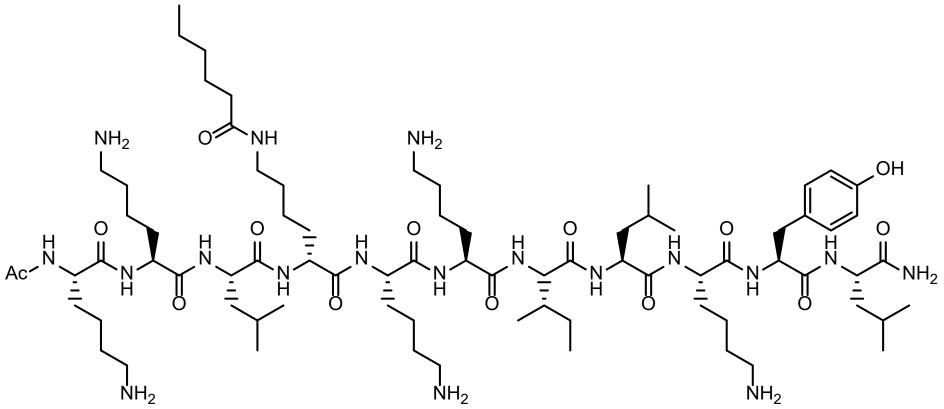

HPLC of crude peptide (λ=220 nm)

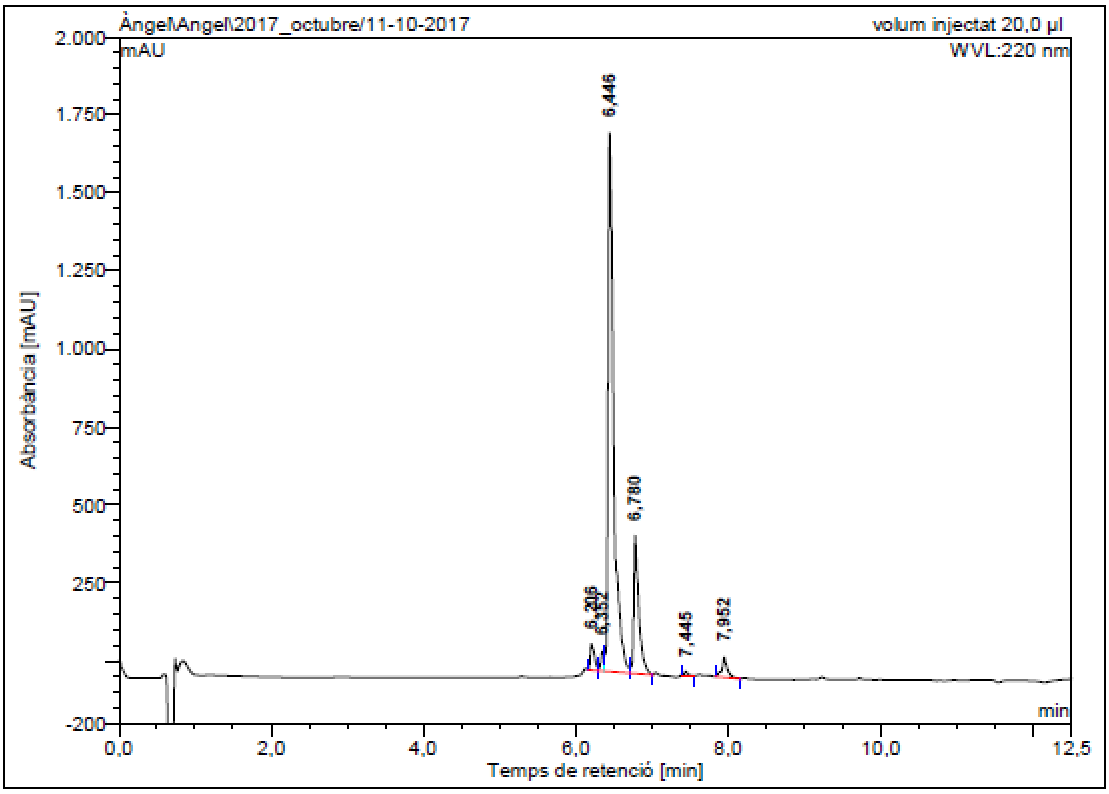

No.	Ret.Time (detected) min	Height mAU	Area mAU*min	Rel.Area %
1	6,21	83,361	5,410	2,75
2	6,35	62,693	3,161	1,61
3	6,45	1725,101	147,916	75,14
4	6,78	441,976	34,458	17,50
5	7,45	13,446	0,734	0,37
6	7,95	63,681	5,177	2,63
Total:		2390,257	196,857	100,00

HPLC of purified peptide (λ=220 nm)

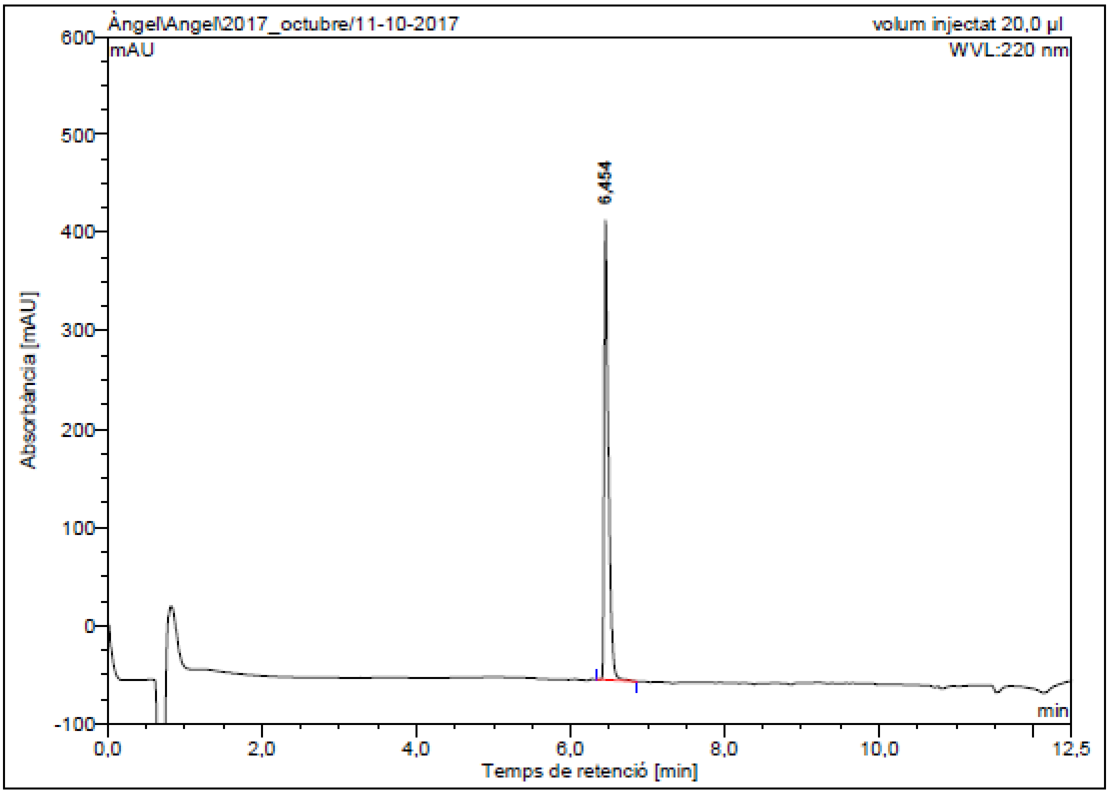

No.	Ret.Time (detected) min	Height mAU	Area mAU*min	Rel.Area %
1	6,45	467,768	30,006	100,00
Total:		467,768	30,006	100,00

ESI-MS (*m/z*)

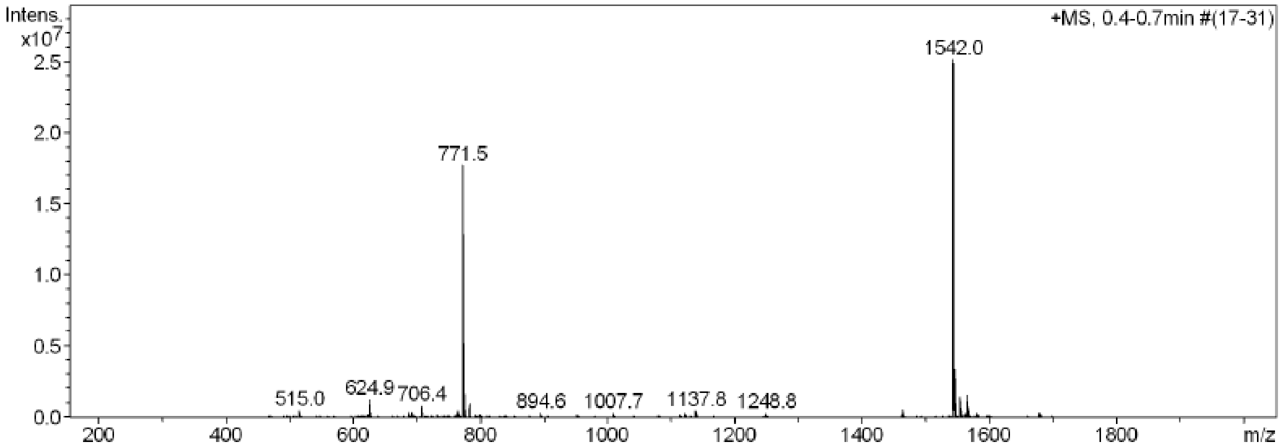

HRMS (*m/z*)

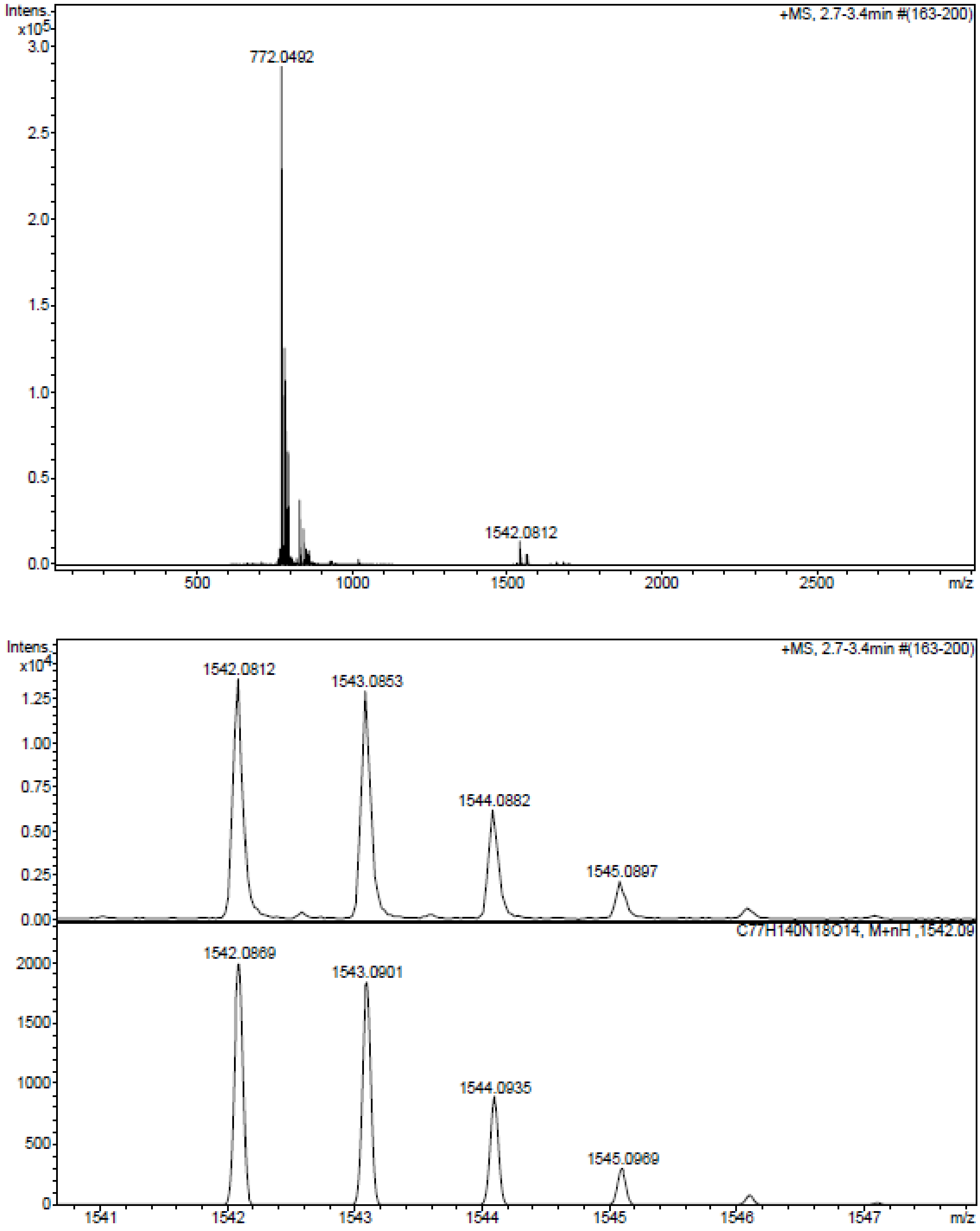
Intens.
x10^5
+MS, 2.7-3.4min #(163-200)
772.0492
1542.0812
m/z
Intens.
x10^4
+MS, 2.7-3.4min #(163-200)
1542.0812
1543.0853
1544.0882
1545.0897
C77H140N18O14, M+nH ,1542.09
1542.0869
1543.0901
1544.0935
1545.0969
m/z

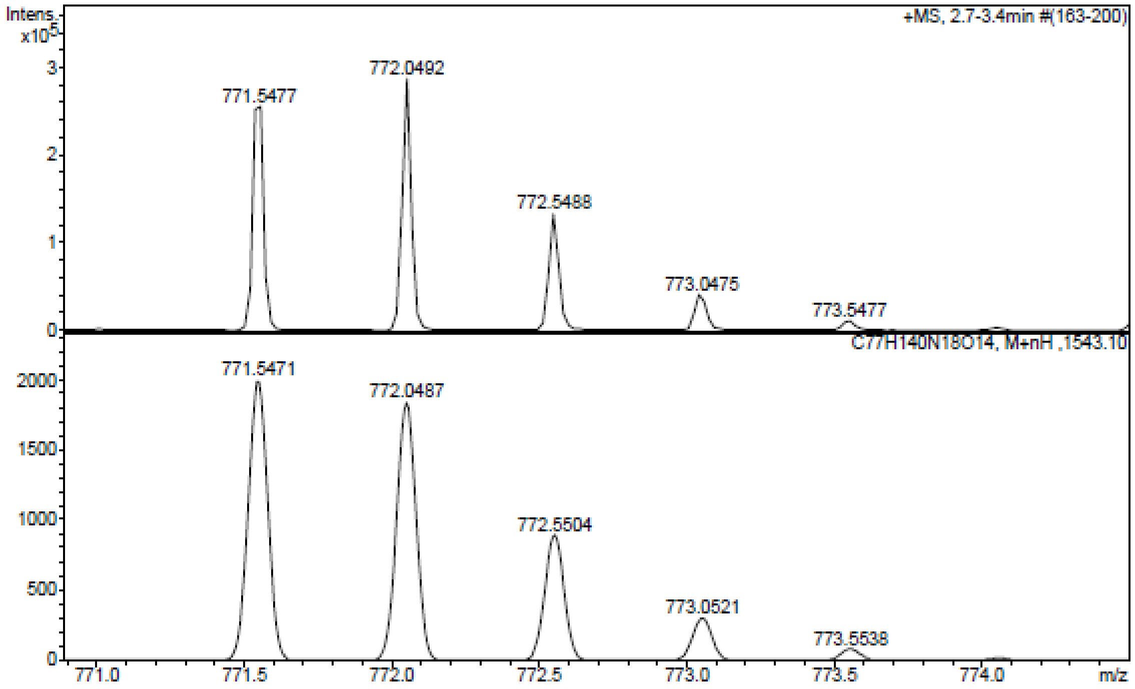
Intens.
x10^5
+MS, 2.7-3.4min #(163-200)
771.5477
772.0492
772.5488
773.0475
773.5477
C77H140N18O14, M+nH ,1543.10
771.5471
772.0487
772.5504
773.0521
773.5538
m/z

C_3H_7CO-Lys-Lys-Leu-D-Phe-Lys-Lys-Ile-Leu-Lys-Tyr-Leu-NH_2 (BP485)

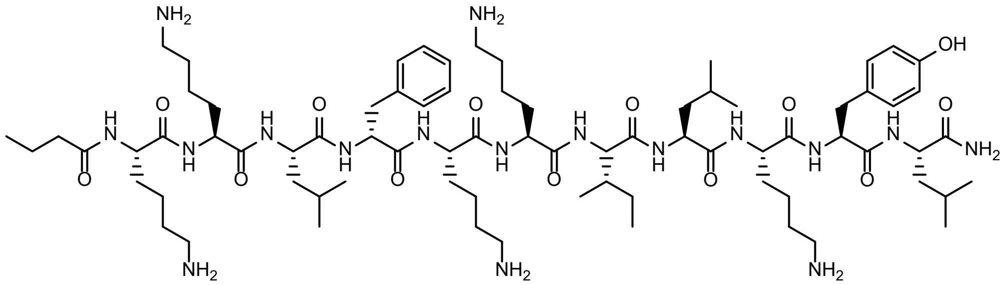

HPLC of crude peptide (λ=220 nm)

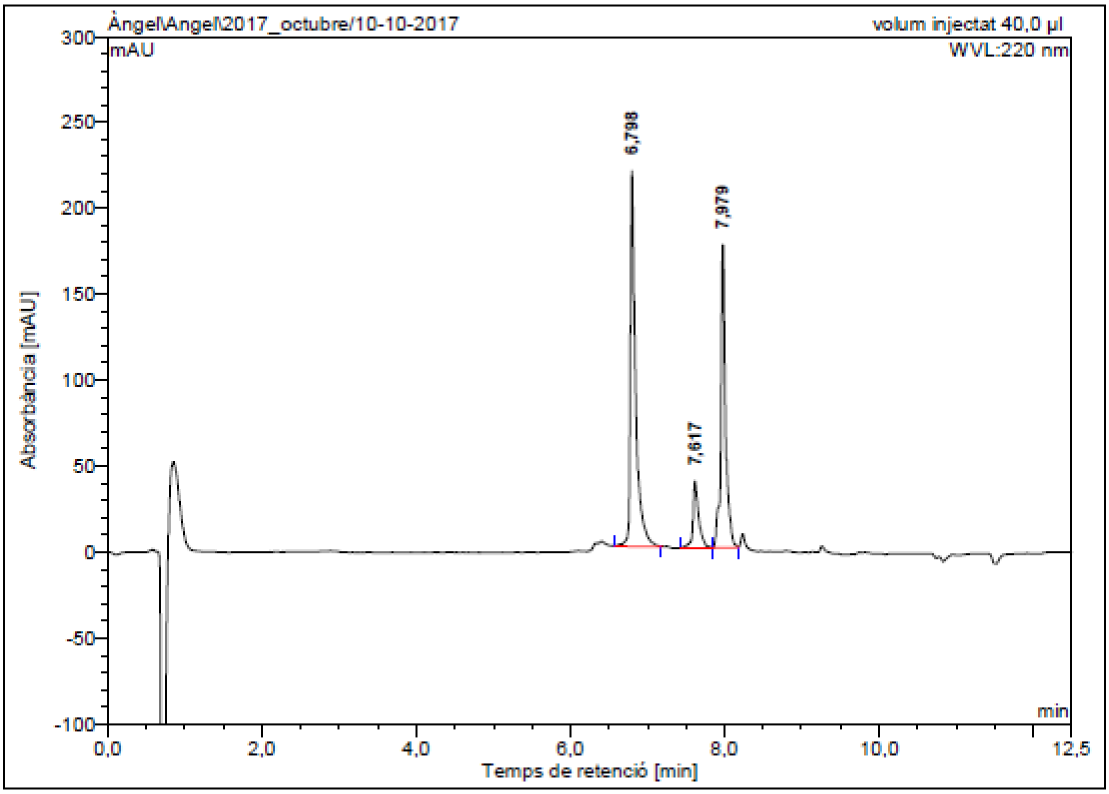

No.	Ret.Time (detected) min	Height mAU	Area mAU*min	Rel.Area %
1	6,80	218,320	17,258	52,25
2	7,62	38,563	3,368	10,20
3	7,98	176,249	12,401	37,55
Total:		433,132	33,026	100,00

HPLC of purified peptide (λ=220 nm)

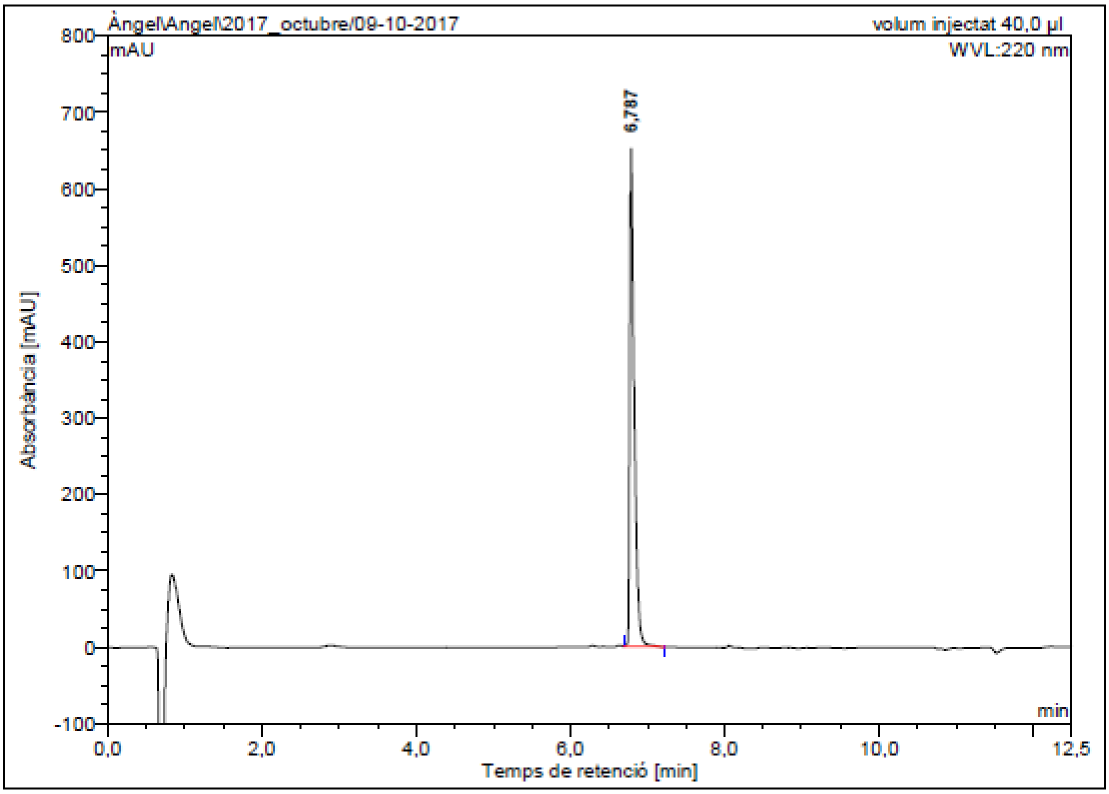

No.	Ret.Time (detected) min	Height mAU	Area mAU*min	Rel.Area %
1	6,79	651,352	44,377	100,00
Total:		651,352	44,377	100,00

ESI-MS (*m/z*)

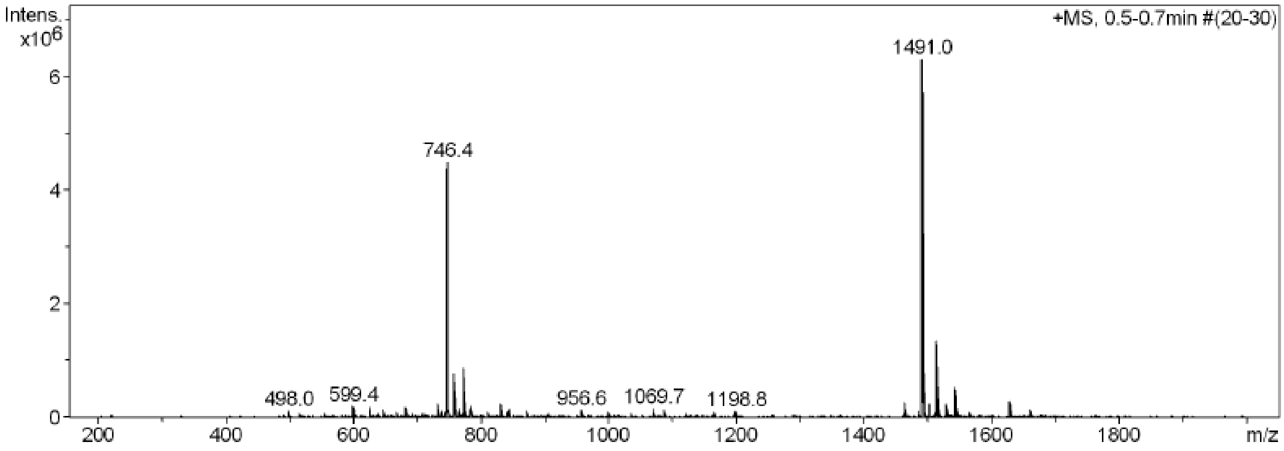

HRMS (*m/z*)

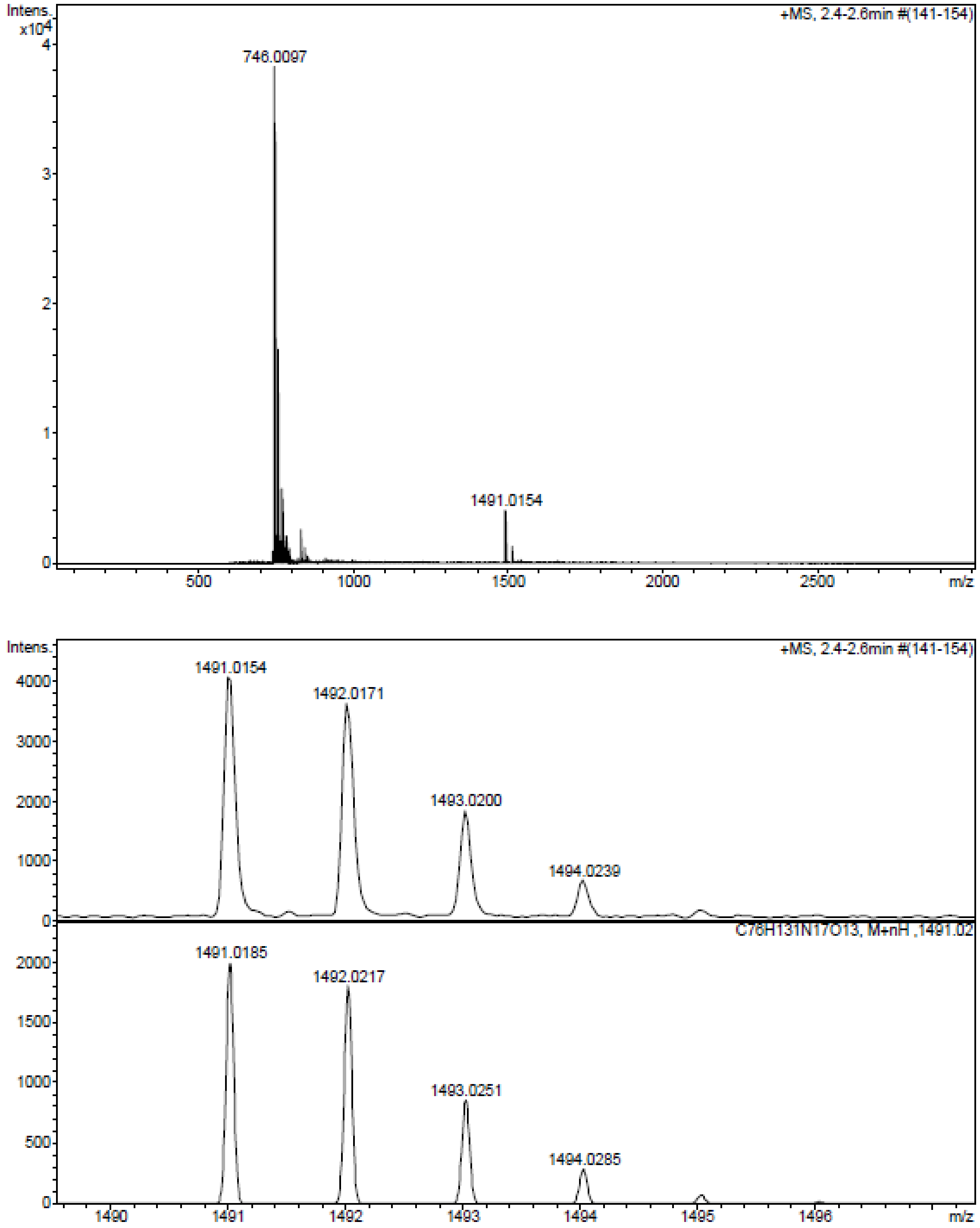
Intens.
x10^4
+MS, 2.4-2.6min #(141-154)
746.0097
1491.0154
500
1000
1500
2000
2500
m/z
Intens.
+MS, 2.4-2.6min #(141-154)
1491.0154
1492.0171
1493.0200
1494.0239
C76H131N17O13, M+nH ,1491.02
1491.0185
1492.0217
1493.0251
1494.0285
1490
1491
1492
1493
1494
1495
1496
m/z

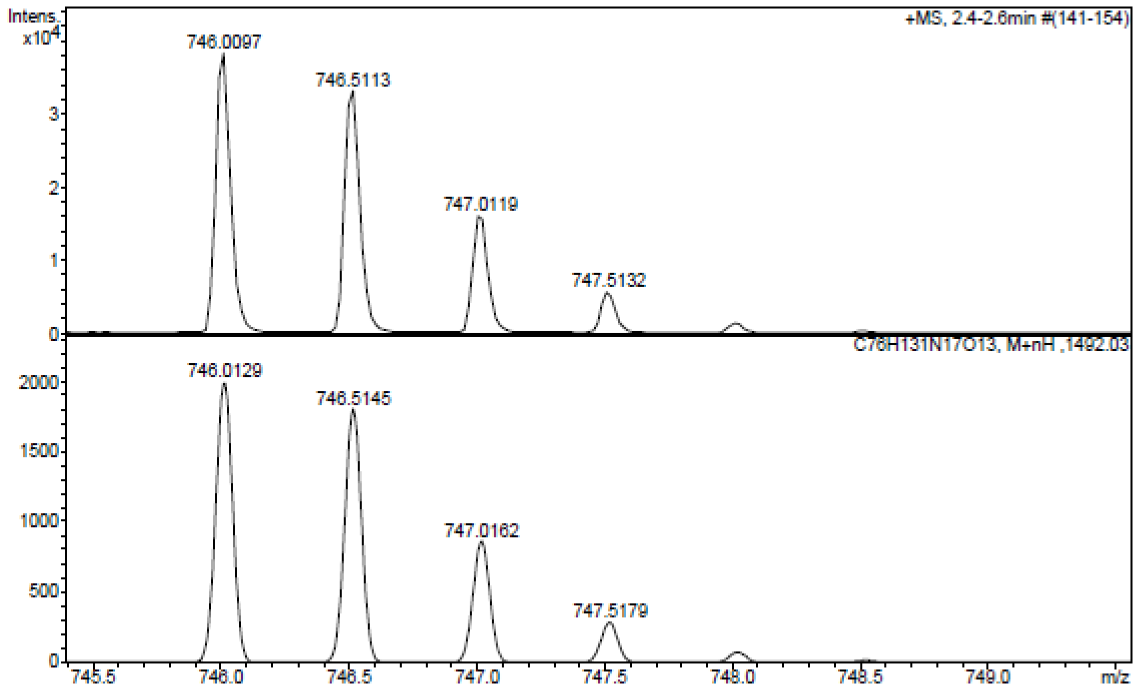
Intens.
x10^4
+MS, 2.4-2.6min #(141-154)
746.0097
746.5113
747.0119
747.5132
C76H131N17O13, M+nH ,1492.03
746.0129
746.5145
747.0162
747.5179
745.5
746.0
746.5
747.0
747.5
748.0
748.5
749.0
m/z

Ac-Lys-Lys(COC$_3$H$_7$)-Leu-D-Phe-Lys-Lys-Ile-Leu-Lys-Tyr-Leu-NH$_2$ (BP486)

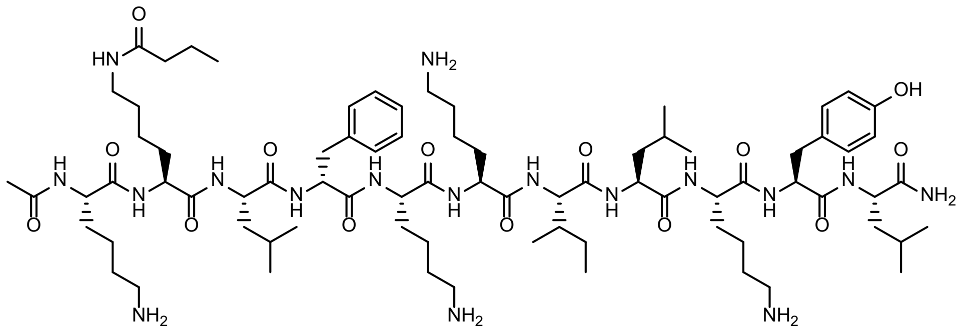

HPLC of crude peptide (λ=220 nm)

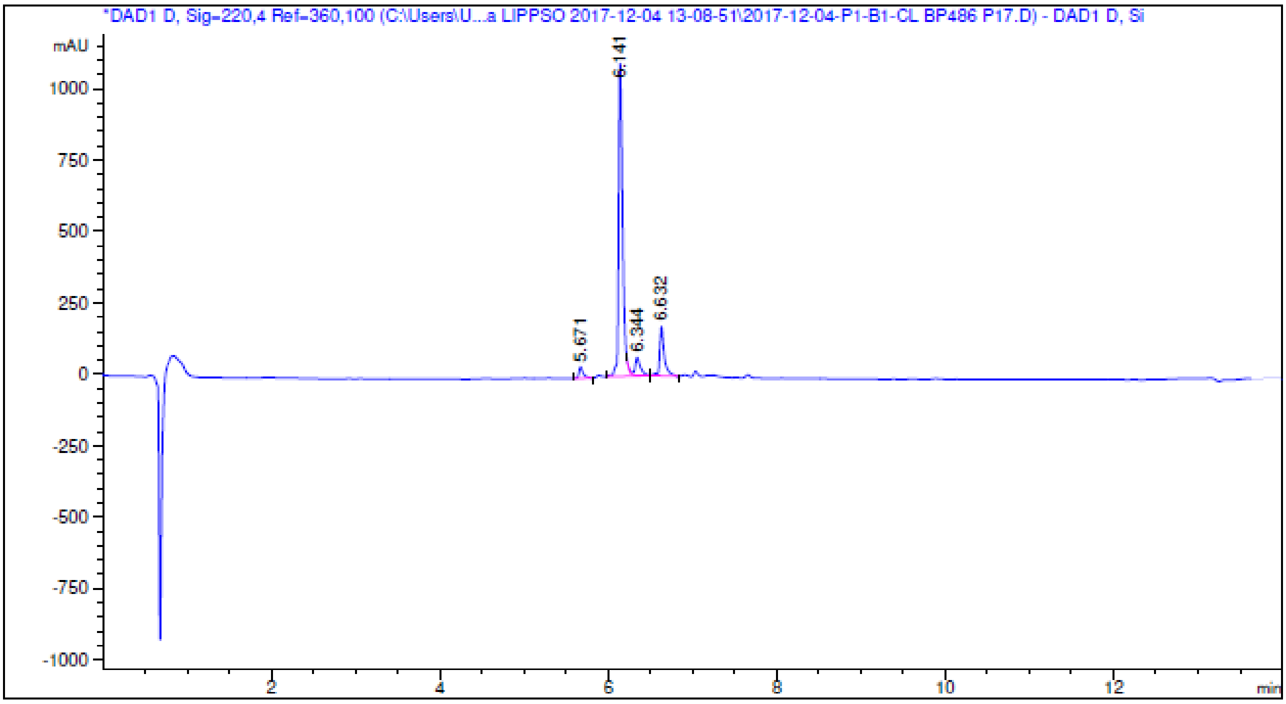

Peak #	RetTime [min]	Type	Width [min]	Area [mAU*s]	Height [mAU]	Area %
1	5.671	BB	0.0522	143.78764	39.70819	3.1093
2	6.141	VV R	0.0479	3568.24097	1098.94568	77.1617
3	6.344	VB E	0.0628	284.22037	63.95224	6.1461
4	6.632	VB R	0.0526	628.11932	172.08841	13.5828

Totals : 4624.36830 1374.69452

HPLC of purified peptide (λ=220 nm)

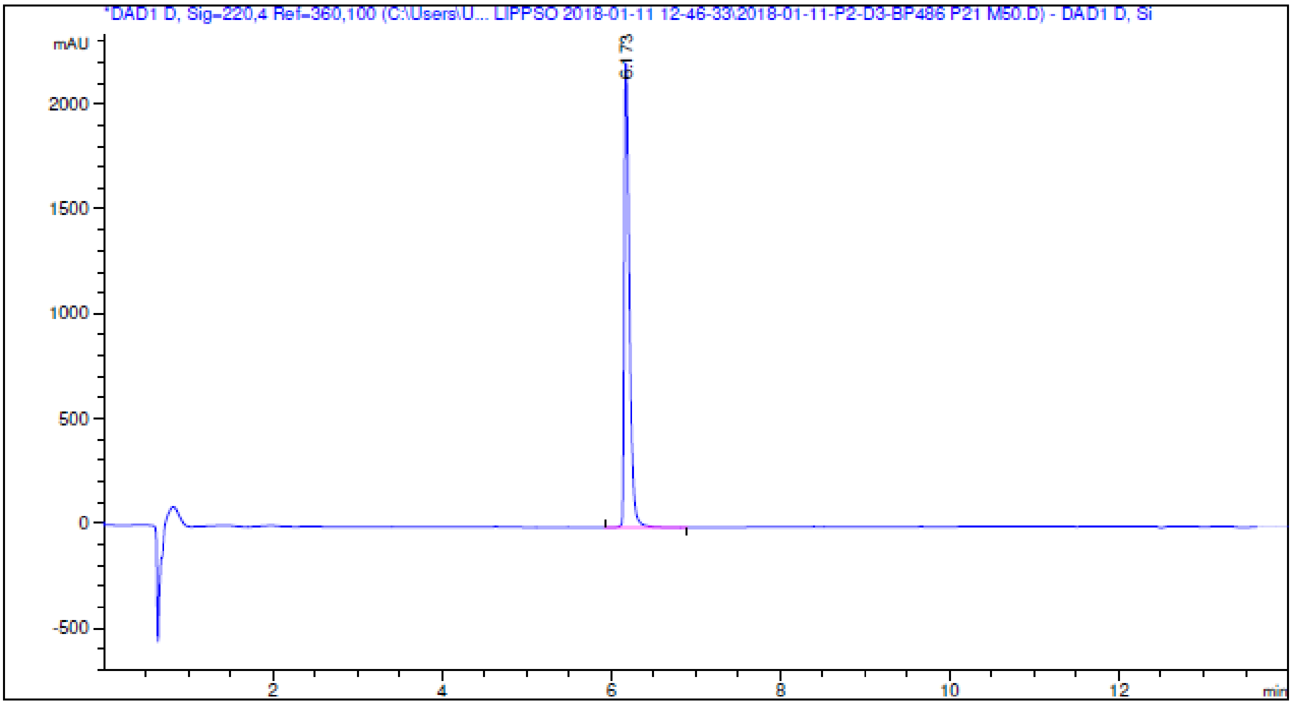

Peak #	RetTime [min]	Type	Width [min]	Area [mAU*s]	Height [mAU]	Area %
1	6.173	BB	0.0634	9405.44824	2219.42041	100.0000
Totals :				9405.44824	2219.42041	

ESI-MS (*m/z*)

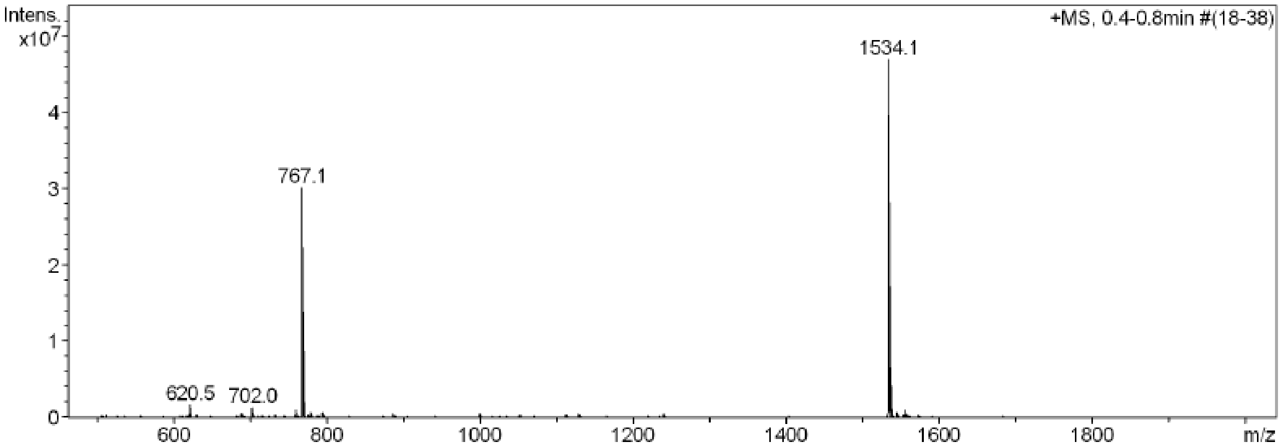

HRMS (m/z)

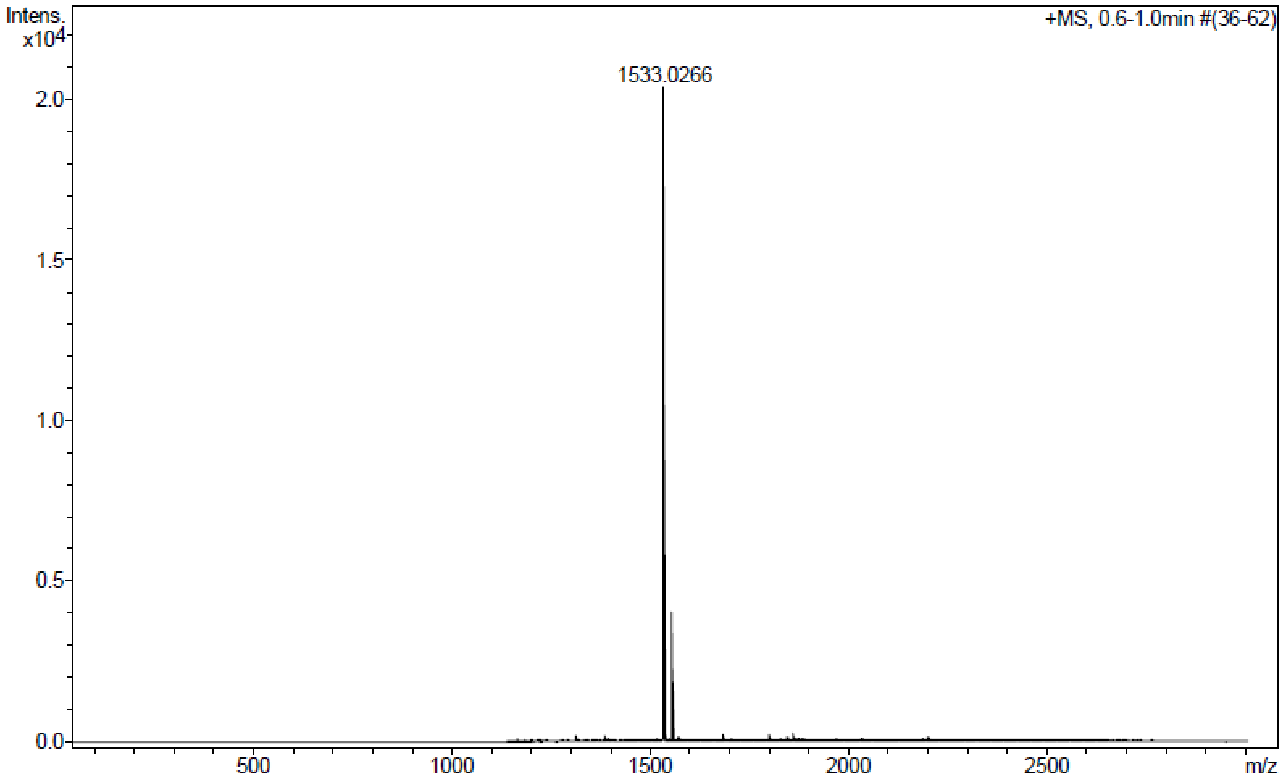

Intens.
x10^4
+MS, 0.6-1.0min #(36-62)
1533.0266
2.0
1.5
1.0
0.5
0.0
500
1000
1500
2000
2500
m/z

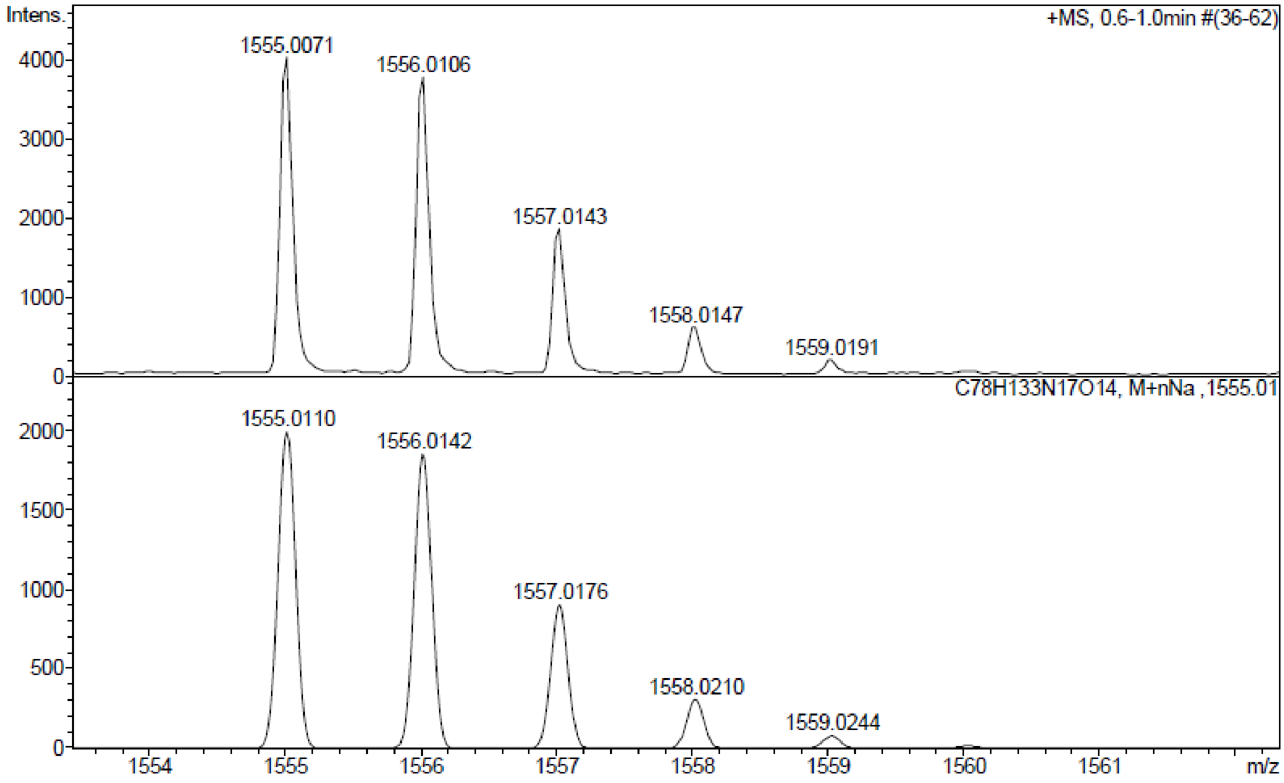

Intens.
+MS, 0.6-1.0min #(36-62)
1555.0071
1556.0106
1557.0143
1558.0147
1559.0191
4000
3000
2000
1000
0
C78H133N17O14, M+nNa ,1555.01
1555.0110
1556.0142
1557.0176
1558.0210
1559.0244
2000
1500
1000
500
0
1554
1555
1556
1557
1558
1559
1560
1561
m/z

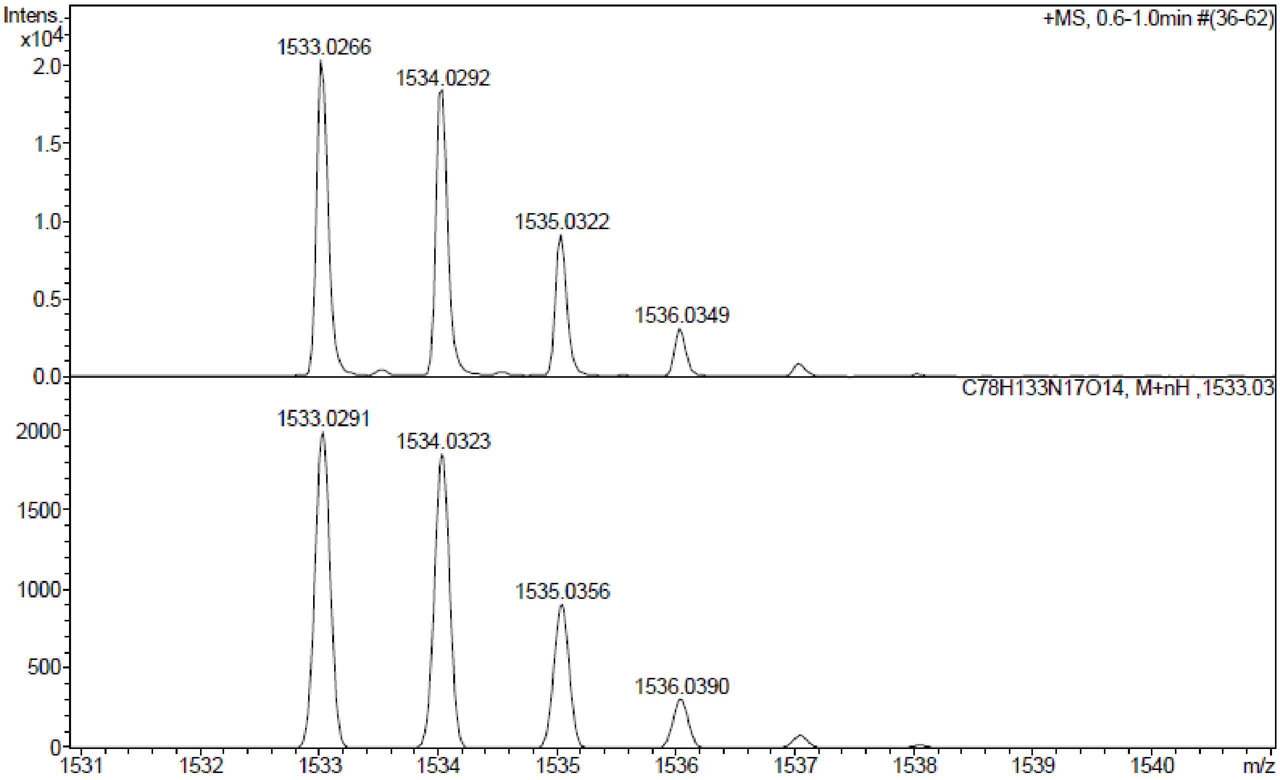
Intens.
x10^4
+MS, 0.6-1.0min #(36-62)
1533.0266
1534.0292
1535.0322
1536.0349
C78H133N17O14, M+nH ,1533.03
1533.0291
1534.0323
1535.0356
1536.0390
m/z

Ac-Lys-Lys-Leu-D-Lys($COC_{11}H_{23}$)-Lys-Lys-Ile-Leu-Lys-Tyr-Leu-NH_2 (BP488)

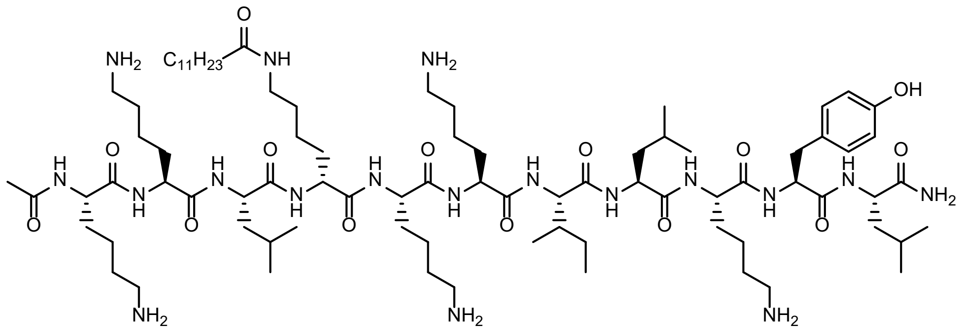

HPLC of crude peptide (λ=220 nm)

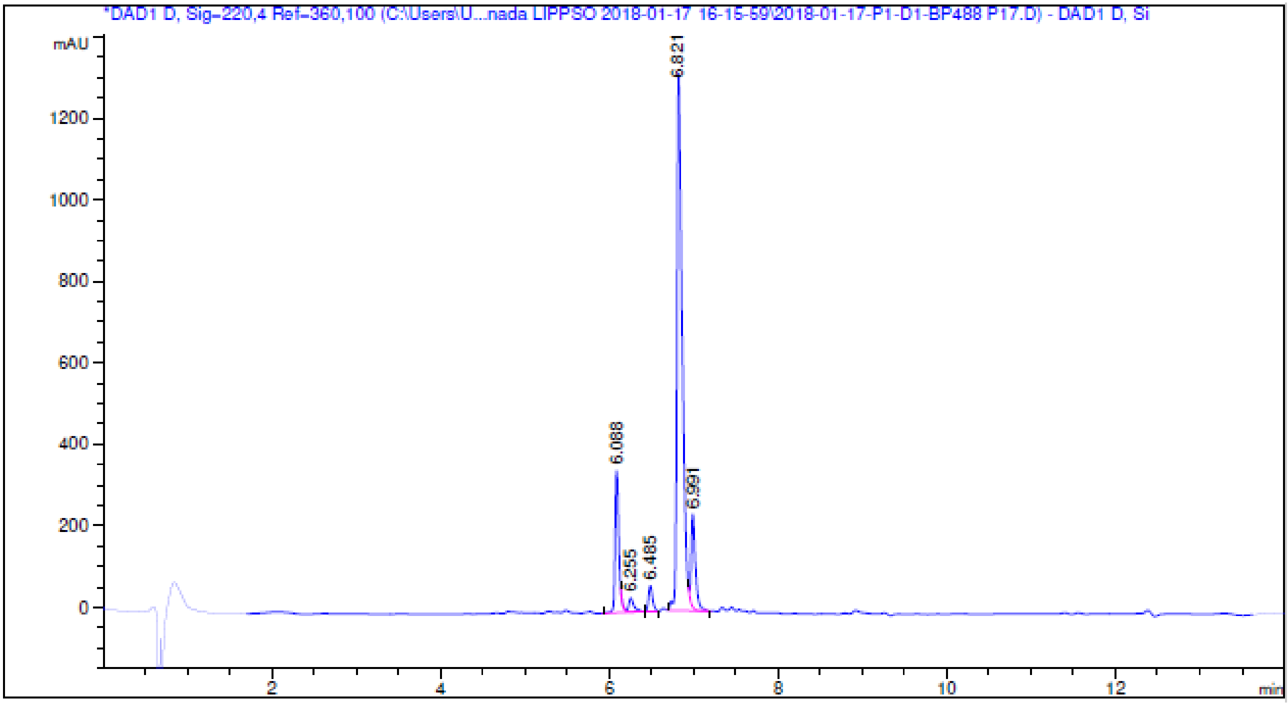

Peak #	RetTime [min]	Type	Width [min]	Area [mAU*s]	Height [mAU]	Area %
1	6.088	BV R	0.0441	1046.76038	347.67551	12.7178
2	6.255	VB E	0.0670	154.87279	33.46687	1.8817
3	6.485	BB	0.0487	202.15788	62.57148	2.4562
4	6.821	VV R	0.0686	6048.61426	1316.82983	73.4888
5	6.991	VB E	0.0507	778.25232	228.61604	9.4555

Totals : 8230.65762 1989.15973

HPLC of purified peptide (λ=220 nm)

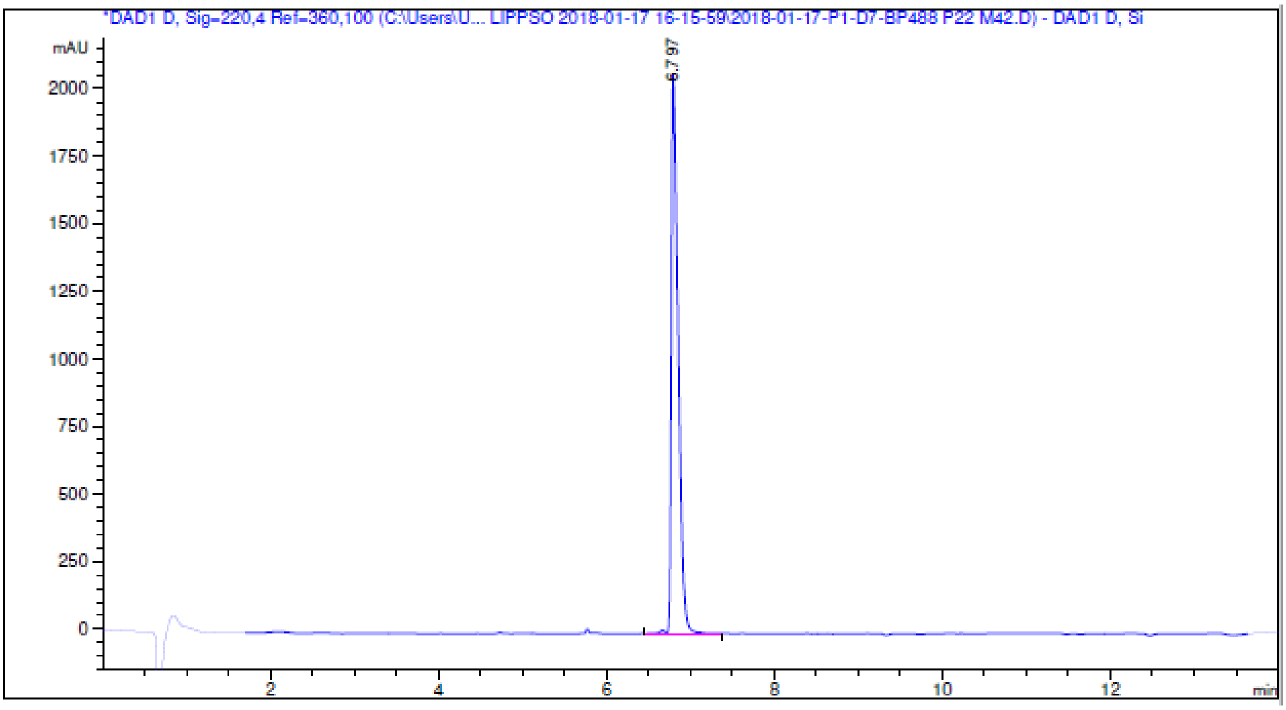

Peak #	RetTime [min]	Type	Width [min]	Area [mAU*s]	Height [mAU]	Area %
1	6.797	VB R	0.0858	1.14854e4	2066.89282	100.0000
Totals :				1.14854e4	2066.89282	

ESI-MS (*m/z*)

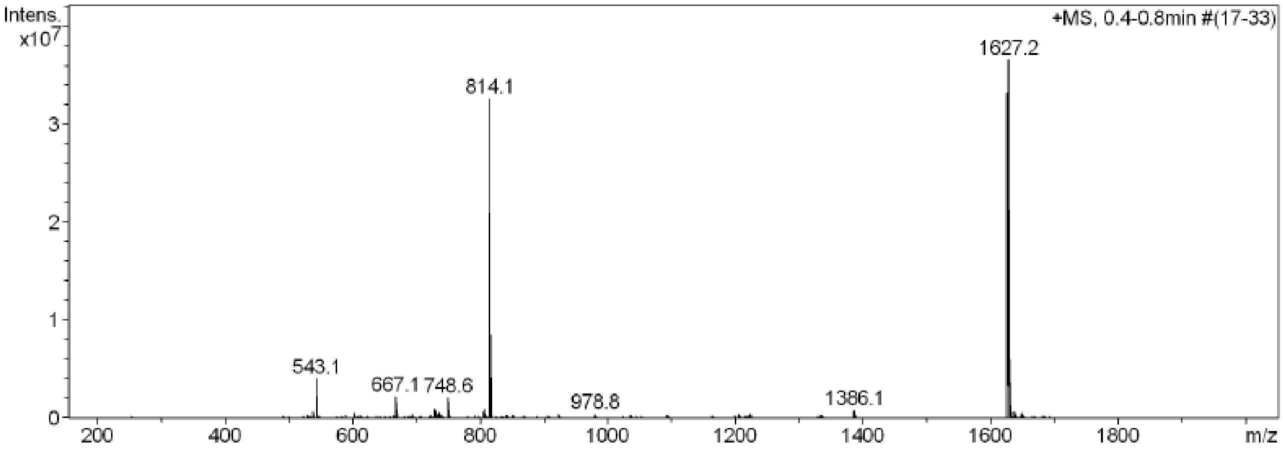

HRMS (m/z)

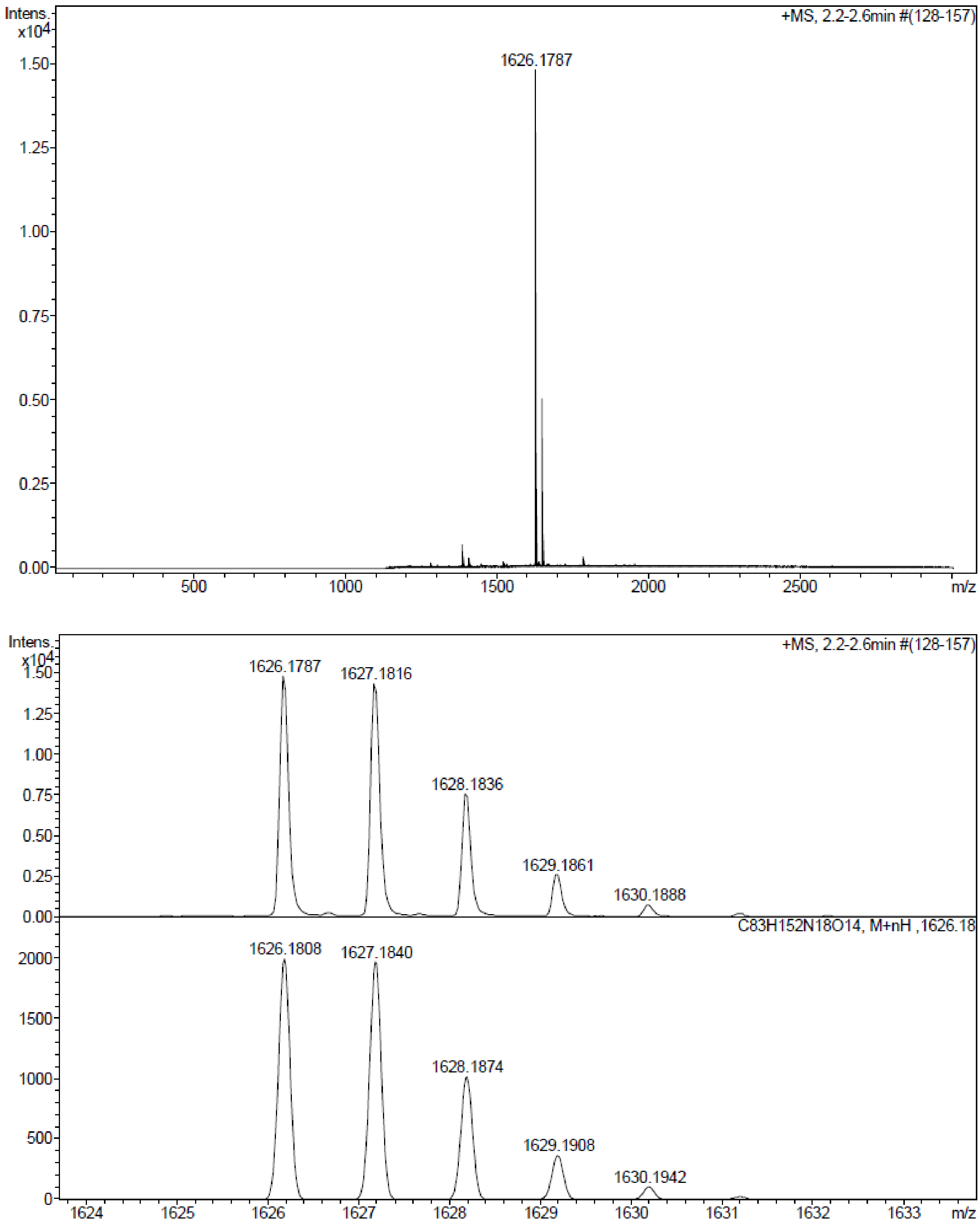

Intens.
x10^4
+MS, 2.2-2.6min #(128-157)
1626.1787
1.50
1.25
1.00
0.75
0.50
0.25
0.00
500
1000
1500
2000
2500
m/z
Intens.
x10^4
+MS, 2.2-2.6min #(128-157)
1626.1787
1627.1816
1628.1836
1629.1861
1630.1888
C83H152N18O14, M+nH ,1626.18
1626.1808
1627.1840
1628.1874
1629.1908
1630.1942
2000
1500
1000
500
0
1624
1625
1626
1627
1628
1629
1630
1631
1632
1633
m/z

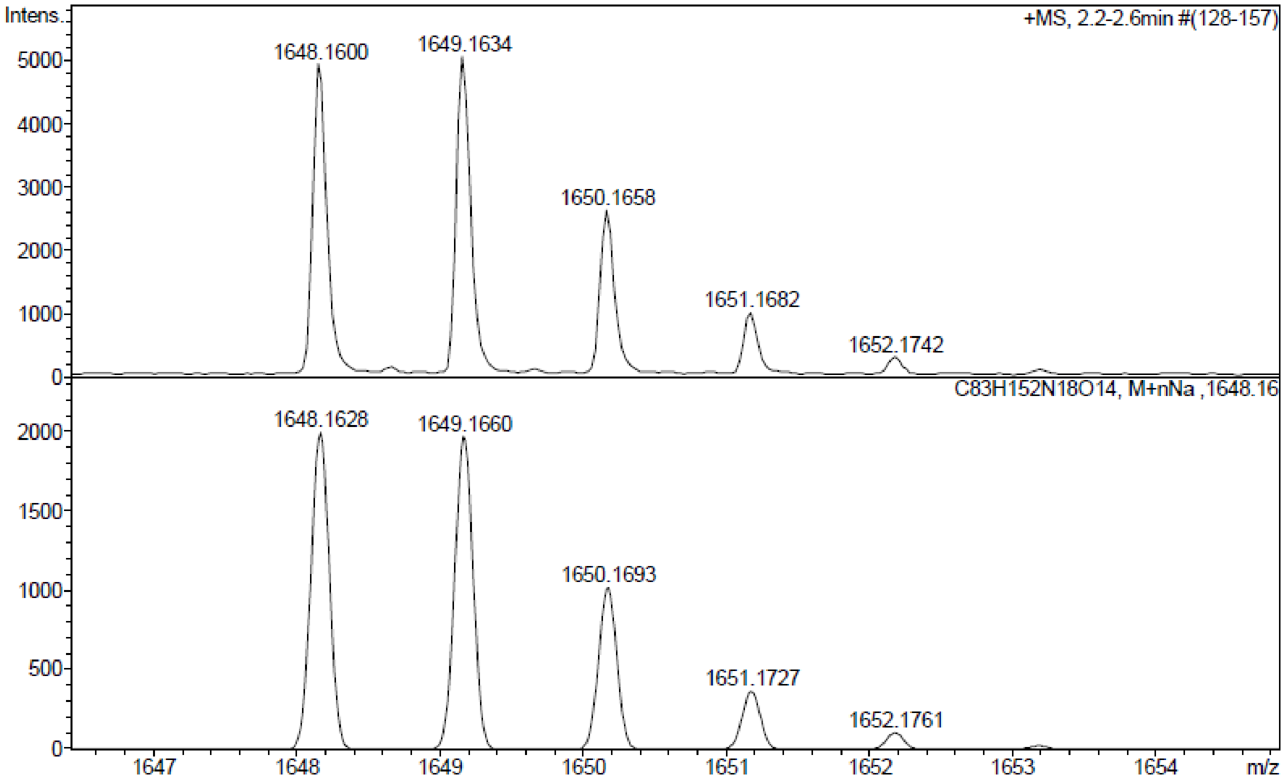
Intens.
+MS, 2.2-2.6min #(128-157)
1648.1600
1649.1634
1650.1658
1651.1682
1652.1742
C83H152N18O14, M+nNa ,1648.16
1648.1628
1649.1660
1650.1693
1651.1727
1652.1761
m/z

Ac-Lys-Lys-Leu-D-Phe-Lys-Lys-Lys($COC_{11}H_{23}$)-Leu-Lys-Tyr-Leu-NH_2 (BP489)

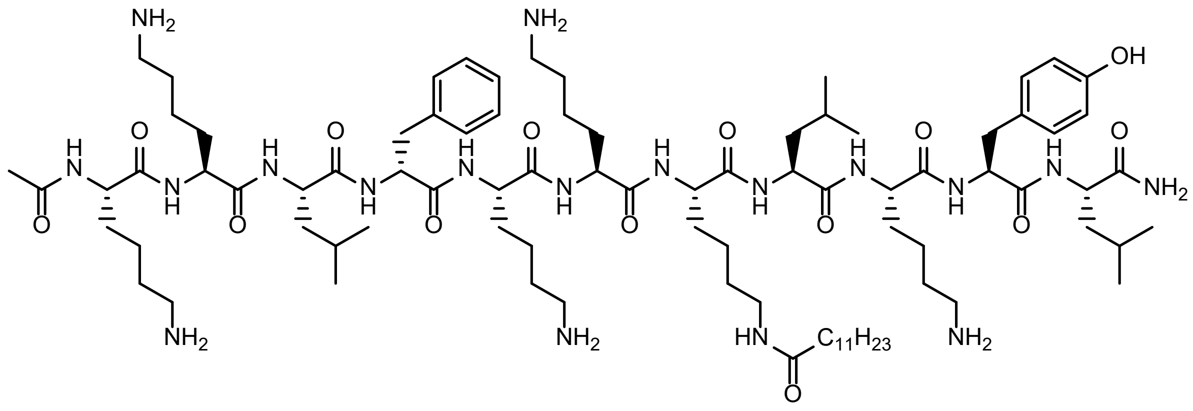

HPLC of crude peptide (λ=220 nm)

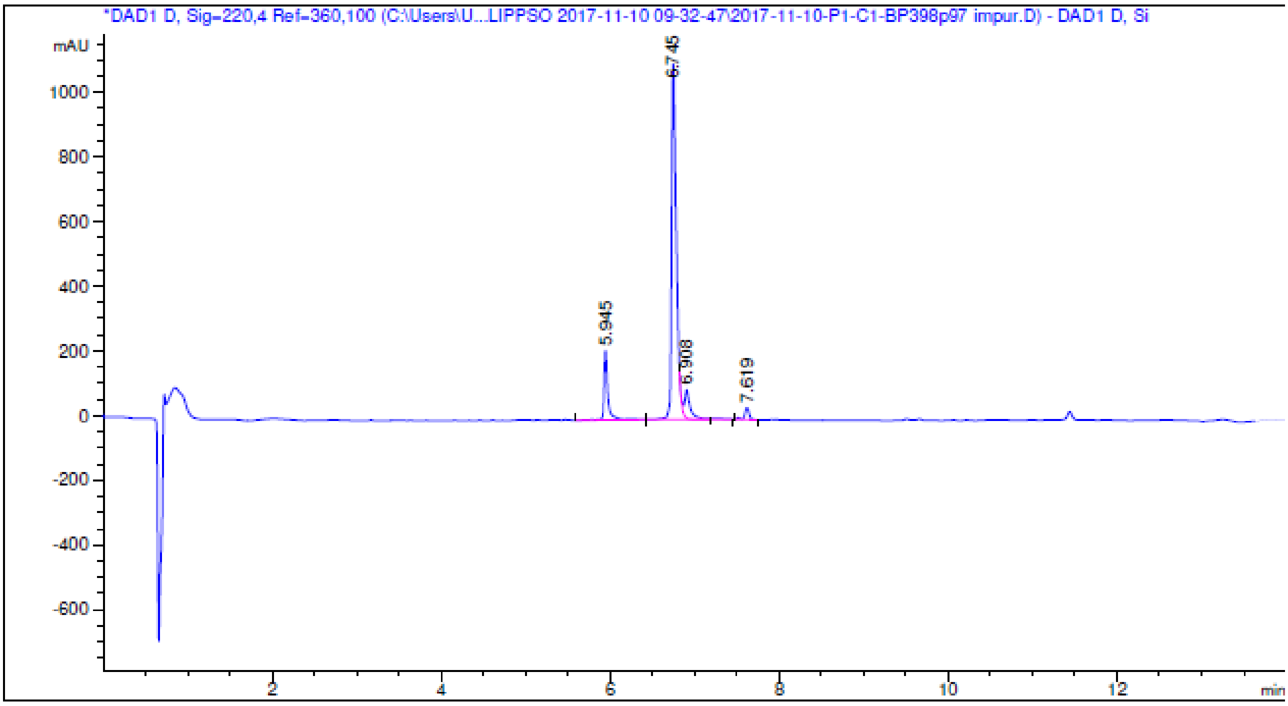

Peak #	RetTime [min]	Type	Width [min]	Area [mAU*s]	Height [mAU]	Area %
1	5.945	VV R	0.0468	694.77289	214.59721	12.4485
2	6.745	BV R	0.0579	4328.80762	1099.50281	77.5611
3	6.908	VV E	0.0672	426.74368	87.06355	7.6461
4	7.619	VB R	0.0553	130.83649	36.04453	2.3443

Totals : 5581.16068 1437.20810

HPLC of purified peptide (λ=220 nm)

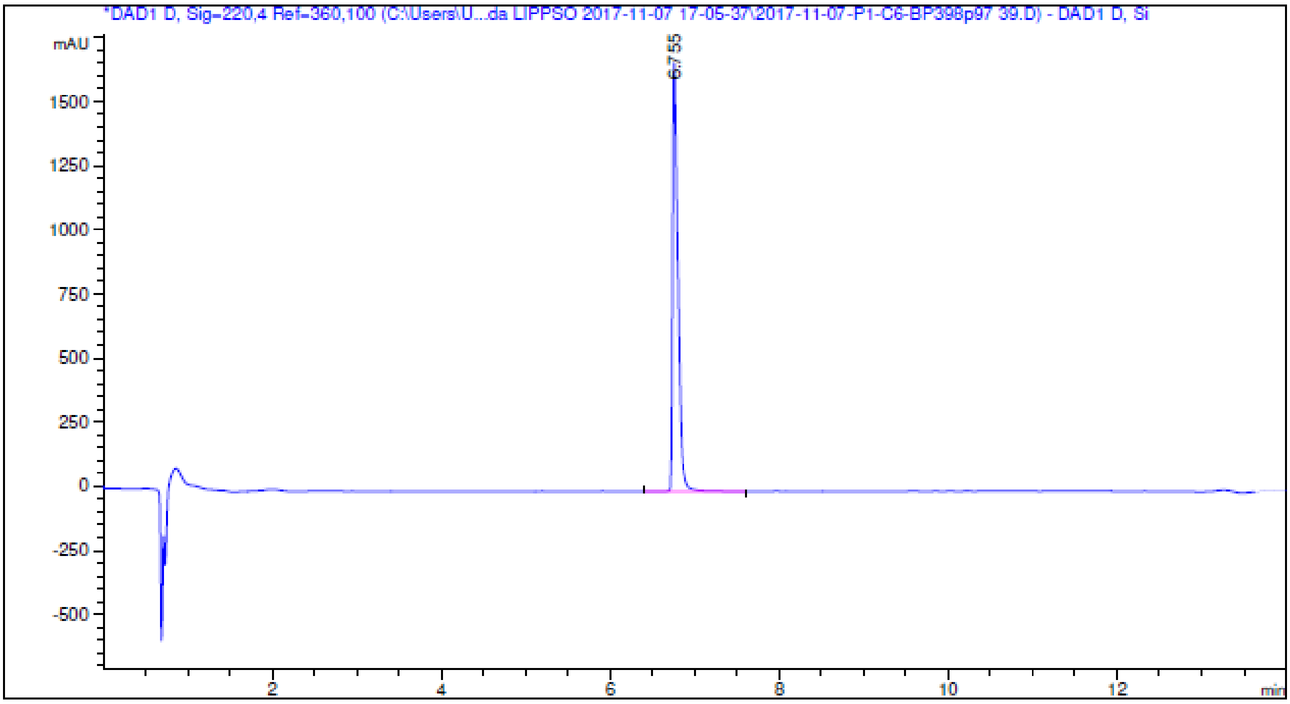

Peak #	RetTime [min]	Type	Width [min]	Area [mAU*s]	Height [mAU]	Area %
1	6.755	BB	0.0665	7391.91846	1673.21887	100.0000
Totals :				7391.91846	1673.21887	

ESI-MS (*m/z*)

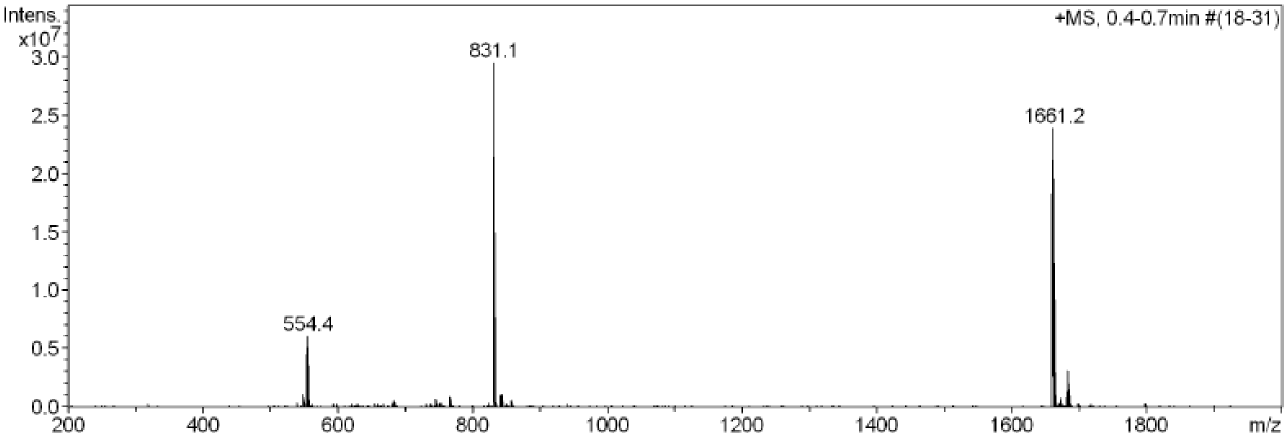

HRMS (*m/z*)

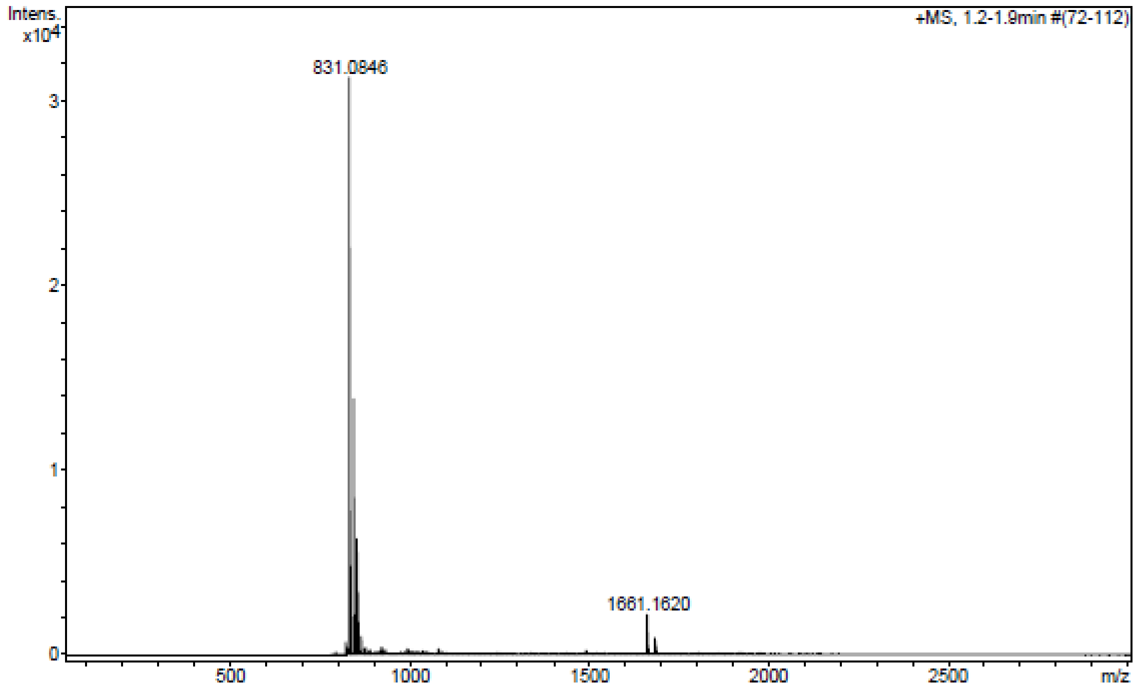
Intens.
x10^4
+MS, 1.2-1.9min #(72-112)
831.0846
1661.1620
0
1
2
3
500
1000
1500
2000
2500
m/z

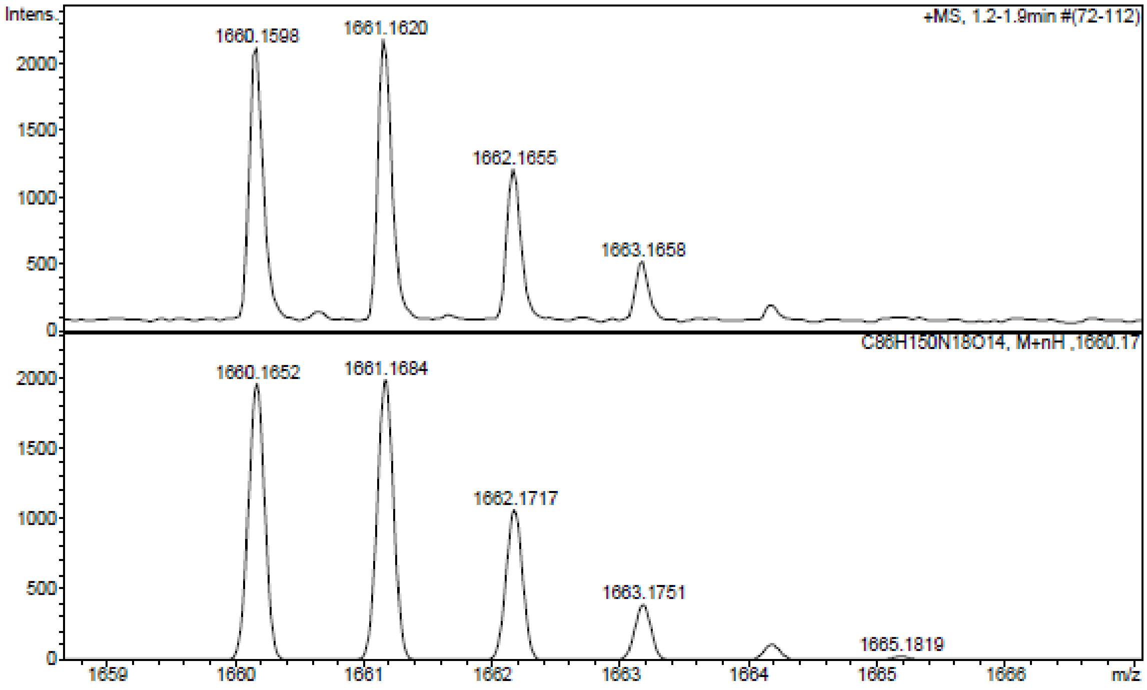
Intens.
+MS, 1.2-1.9min #(72-112)
1660.1598
1661.1620
1662.1655
1663.1658
C86H150N18O14, M+nH ,1660.17
1660.1652
1661.1684
1662.1717
1663.1751
1665.1819
0
500
1000
1500
2000
1659
1660
1661
1662
1663
1664
1665
1666
m/z

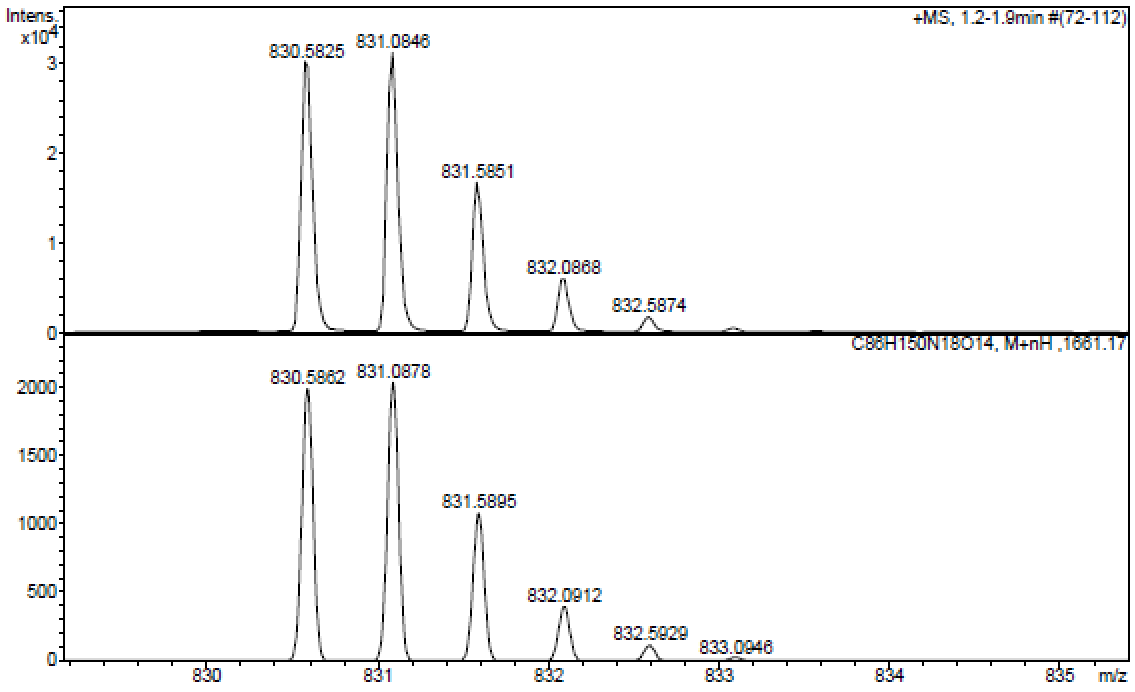

Intens.
x10^4
+MS, 1.2-1.9min #(72-112)
830.5825
831.0846
831.5851
832.0868
832.5874
C86H150N18O14, M+nH ,1661.17
830.5862
831.0878
831.5895
832.0912
832.5929
833.0946
m/z

Ac-Lys-Lys-Leu-D-Phe-Lys-Lys-Ile-Lys($COC_{11}H_{23}$)-Lys-Tyr-Leu-NH_2 (BP490)

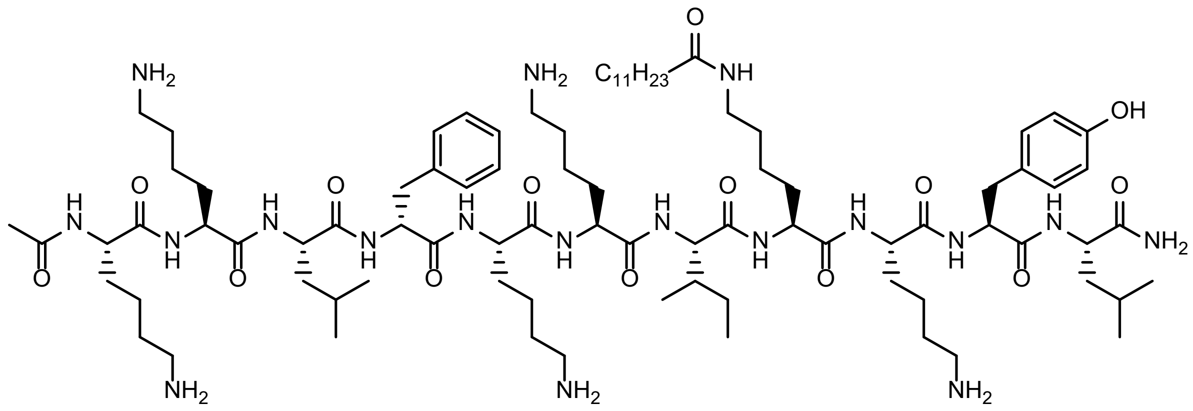

HPLC of crude peptide (λ=220 nm)

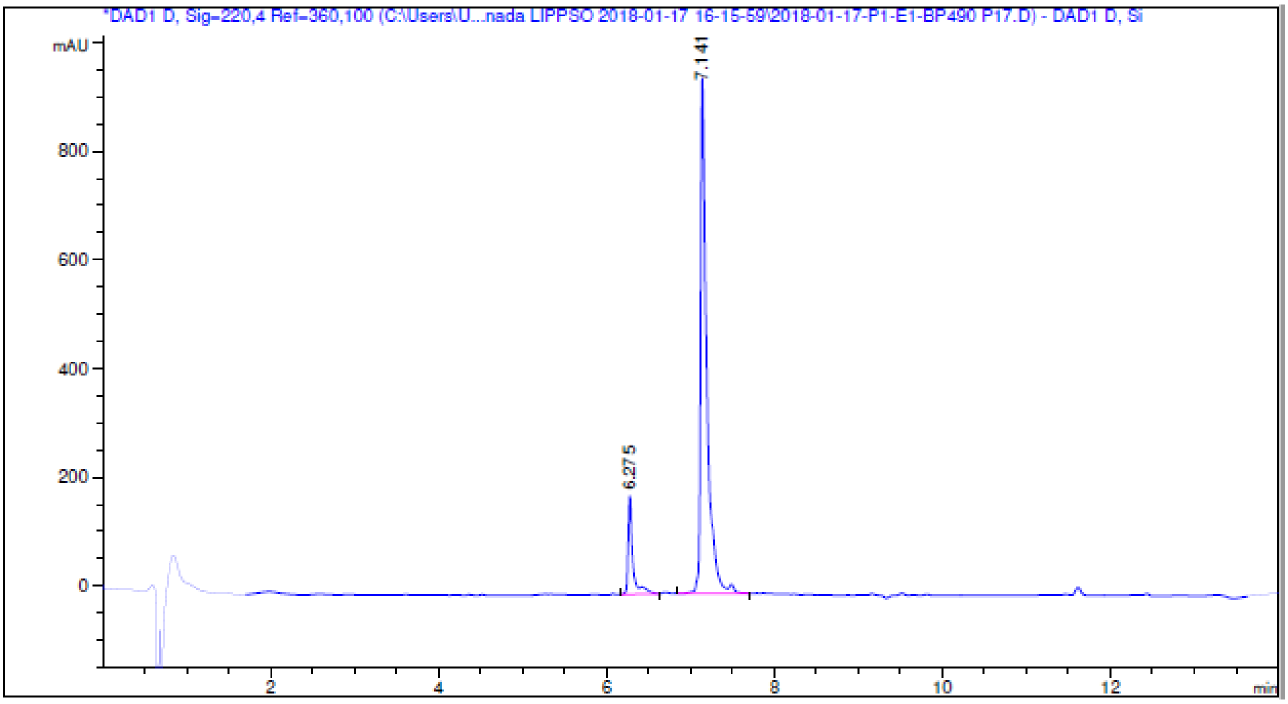

Peak #	RetTime [min]	Type	Width [min]	Area [mAU*s]	Height [mAU]	Area %
1	6.275	BV R	0.0575	723.08496	181.44606	12.9286
2	7.141	BV R	0.0729	4869.80566	948.12061	87.0714

Totals : 5592.89063 1129.56667

HPLC of purified peptide (λ=220 nm)

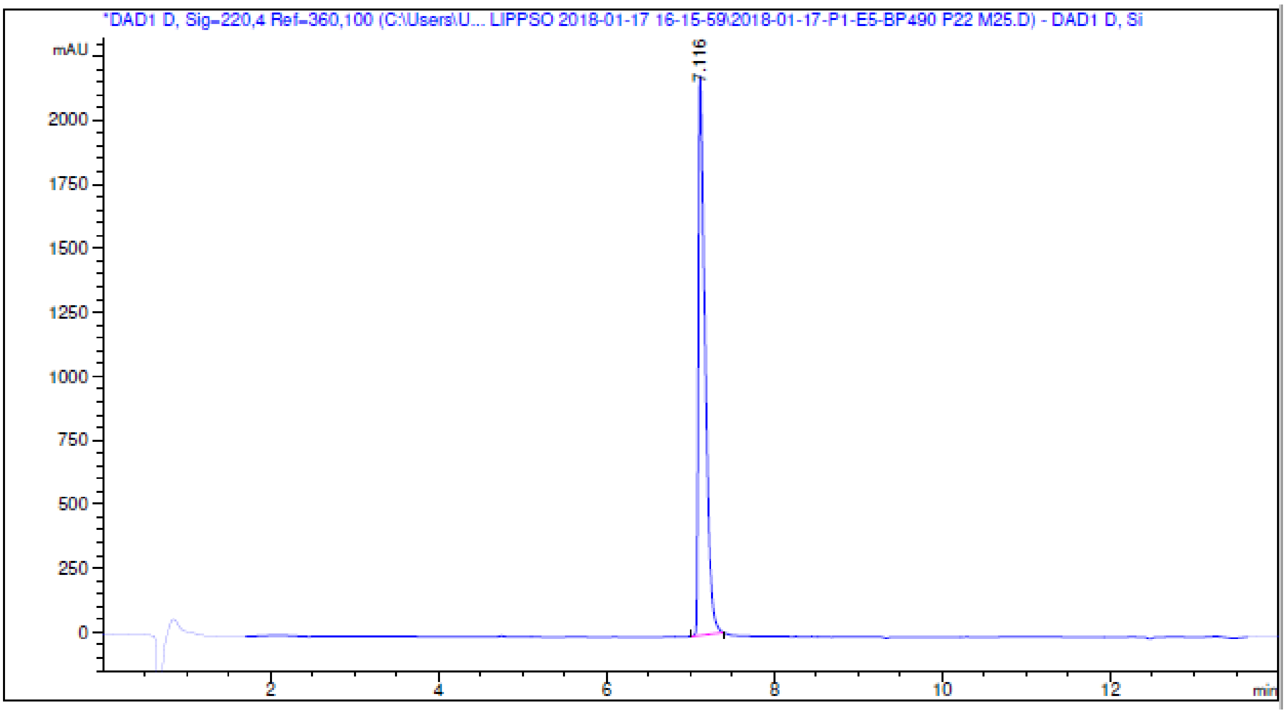

Peak #	RetTime [min]	Type	Width [min]	Area [mAU*s]	Height [mAU]	Area %
1	7.116	BBA	0.0821	1.20145e4	2186.71582	100.0000

Totals : 1.20145e4 2186.71582

ESI-MS (*m/z*)

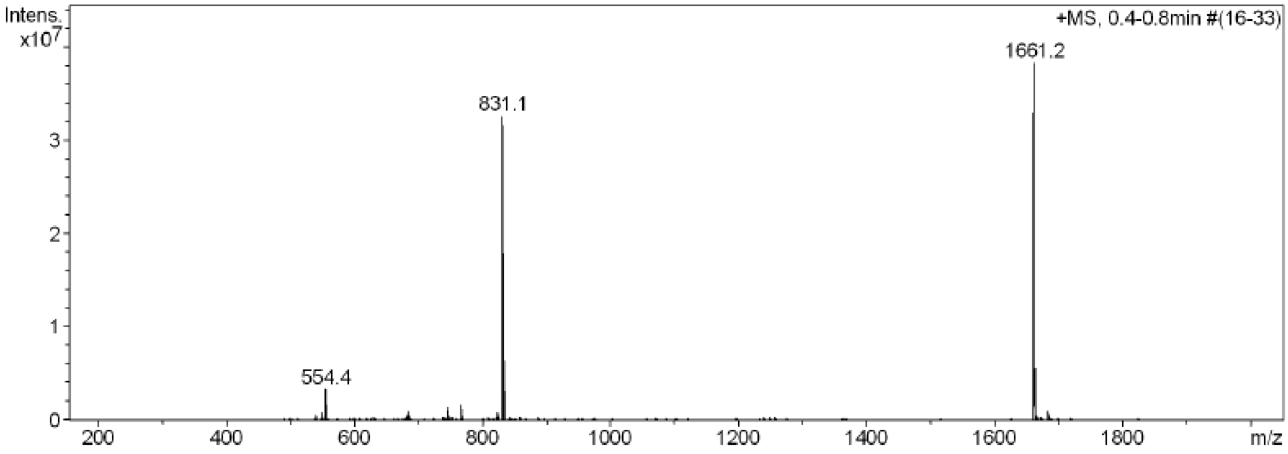

HRMS (m/z)

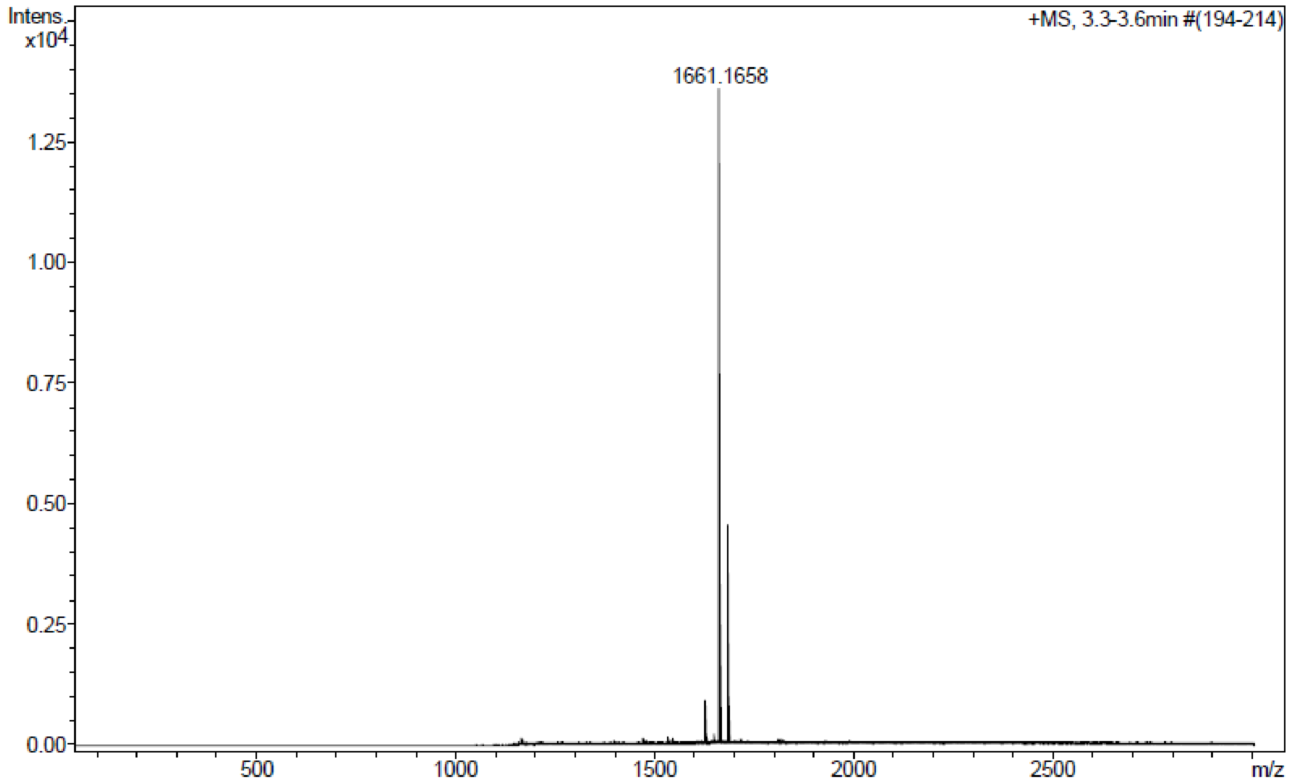
Intens.
x10^4
+MS, 3.3-3.6min #(194-214)
1661.1658
1.25
1.00
0.75
0.50
0.25
0.00
500
1000
1500
2000
2500
m/z

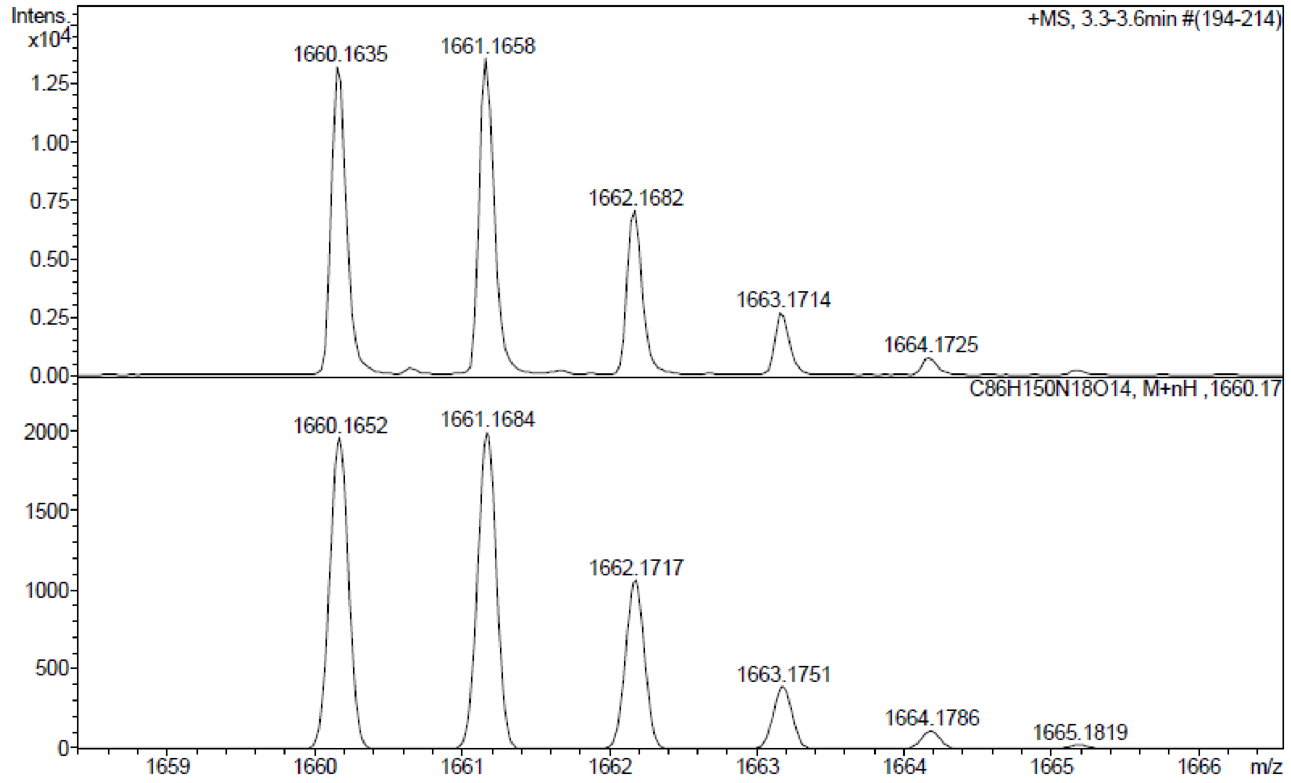
Intens.
x10^4
+MS, 3.3-3.6min #(194-214)
1660.1635
1661.1658
1662.1682
1663.1714
1664.1725
1.25
1.00
0.75
0.50
0.25
0.00
C86H150N18O14, M+nH ,1660.17
1660.1652
1661.1684
1662.1717
1663.1751
1664.1786
1665.1819
2000
1500
1000
500
0
1659
1660
1661
1662
1663
1664
1665
1666
m/z

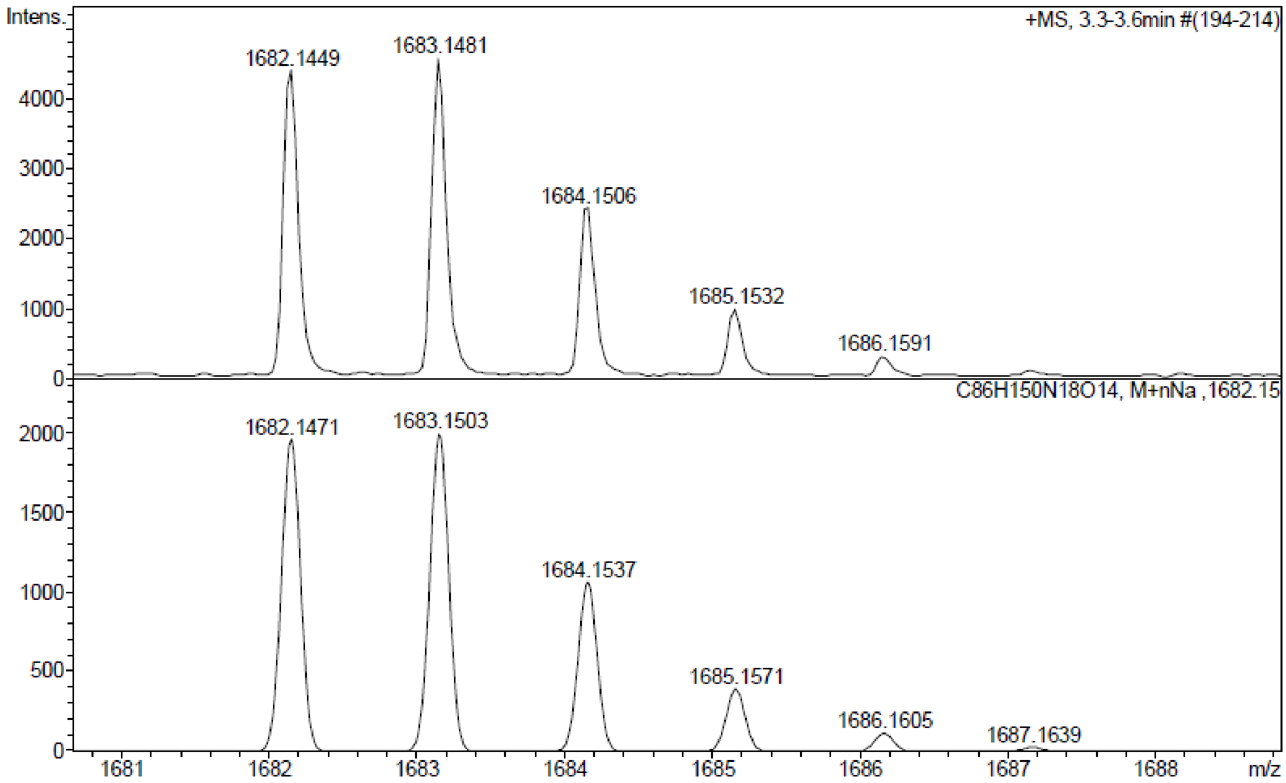

Intens.
+MS, 3.3-3.6min #(194-214)
1682.1449
1683.1481
1684.1506
1685.1532
1686.1591
C86H150N18O14, M+nNa ,1682.15
1682.1471
1683.1503
1684.1537
1685.1571
1686.1605
1687.1639
m/z

Ac-Lys-Lys-Leu-D-Phe-Lys-Lys-Lys(COC_5H_{11})-Leu-Lys-Tyr-Leu-NH_2 (BP494)

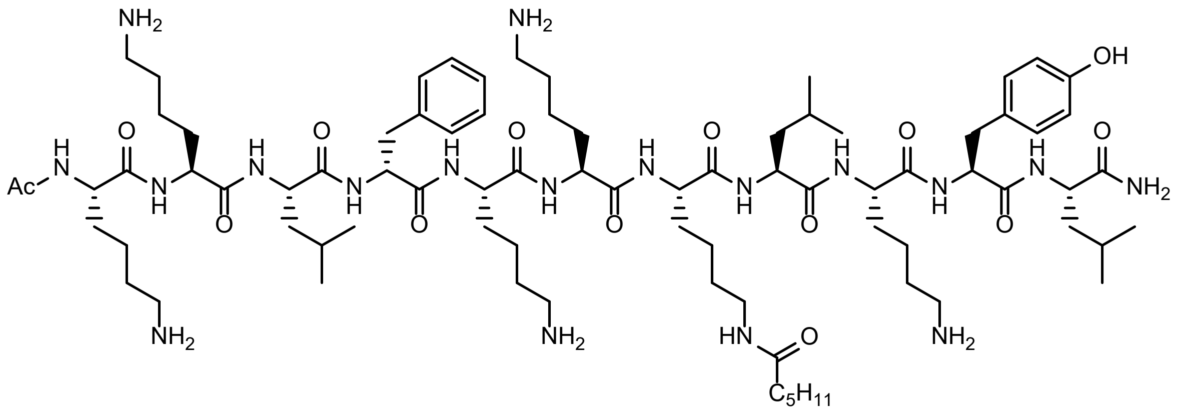

HPLC of crude peptide (λ=220 nm)

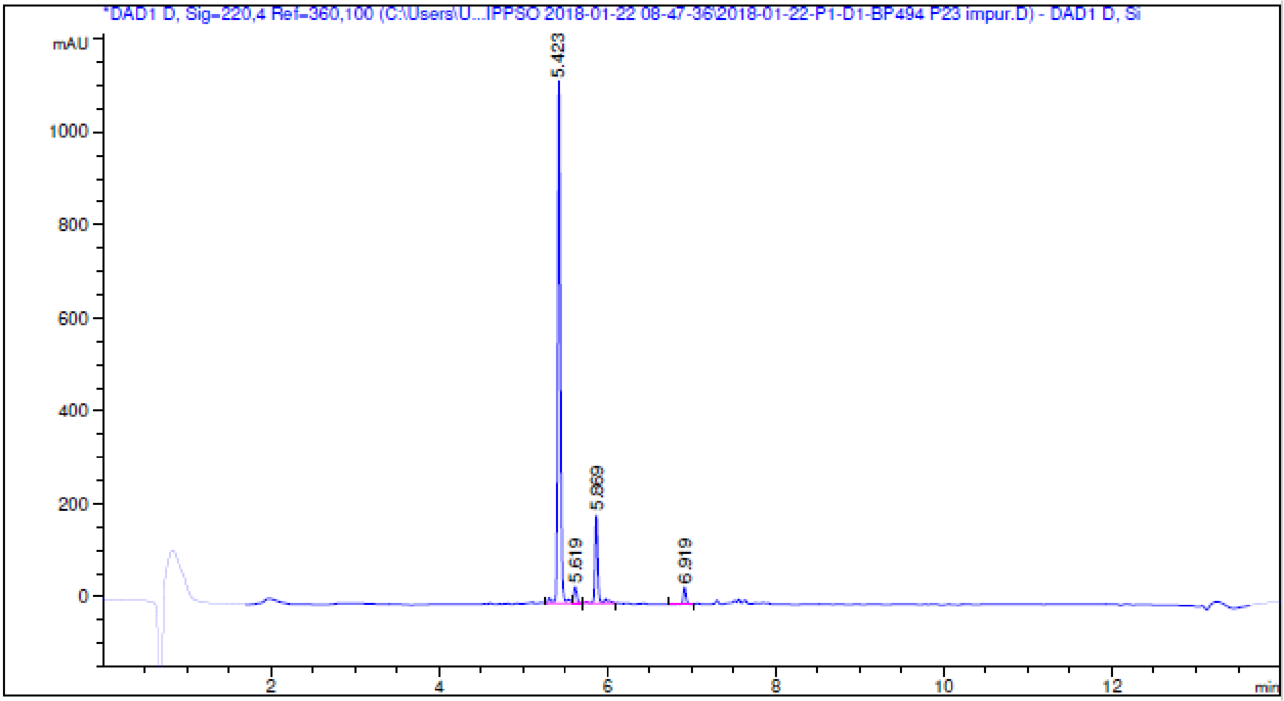

Peak #	RetTime [min]	Type	Width [min]	Area [mAU*s]	Height [mAU]	Area %
1	5.423	VV R	0.0340	2517.67651	1130.30176	80.8293
2	5.619	VB E	0.0374	84.14298	34.50963	2.7014
3	5.869	VV R	0.0346	432.04156	189.79099	13.8706
4	6.919	VV R	0.0340	80.94740	36.32489	2.5988
Totals :				3114.80846	1390.92727	

HPLC of purified peptide (λ=220 nm)

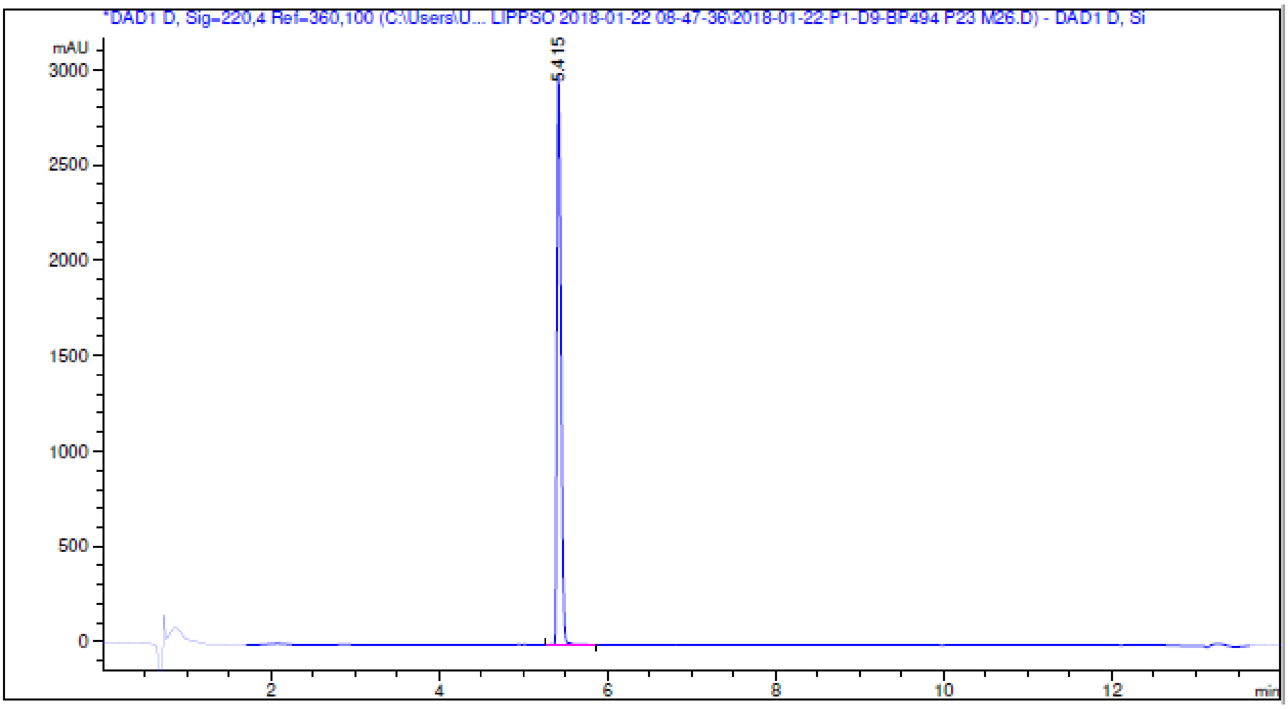

Peak #	RetTime [min]	Type	Width [min]	Area [mAU*s]	Height [mAU]	Area %
1	5.415	VV R	0.0536	9889.14844	2990.65039	100.0000
Totals :				9889.14844	2990.65039	

ESI-MS (*m/z*)

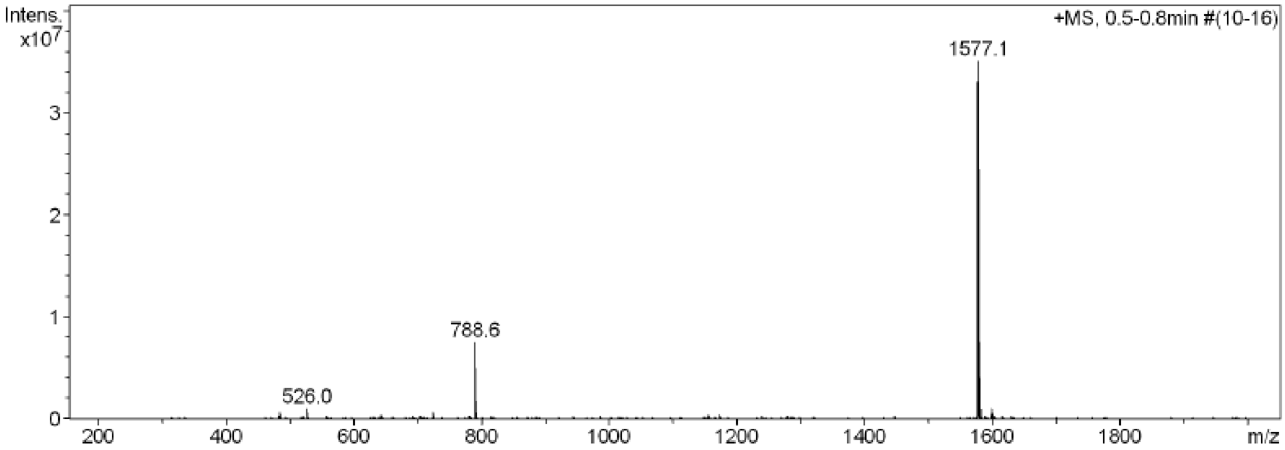

HRMS (*m/z*)

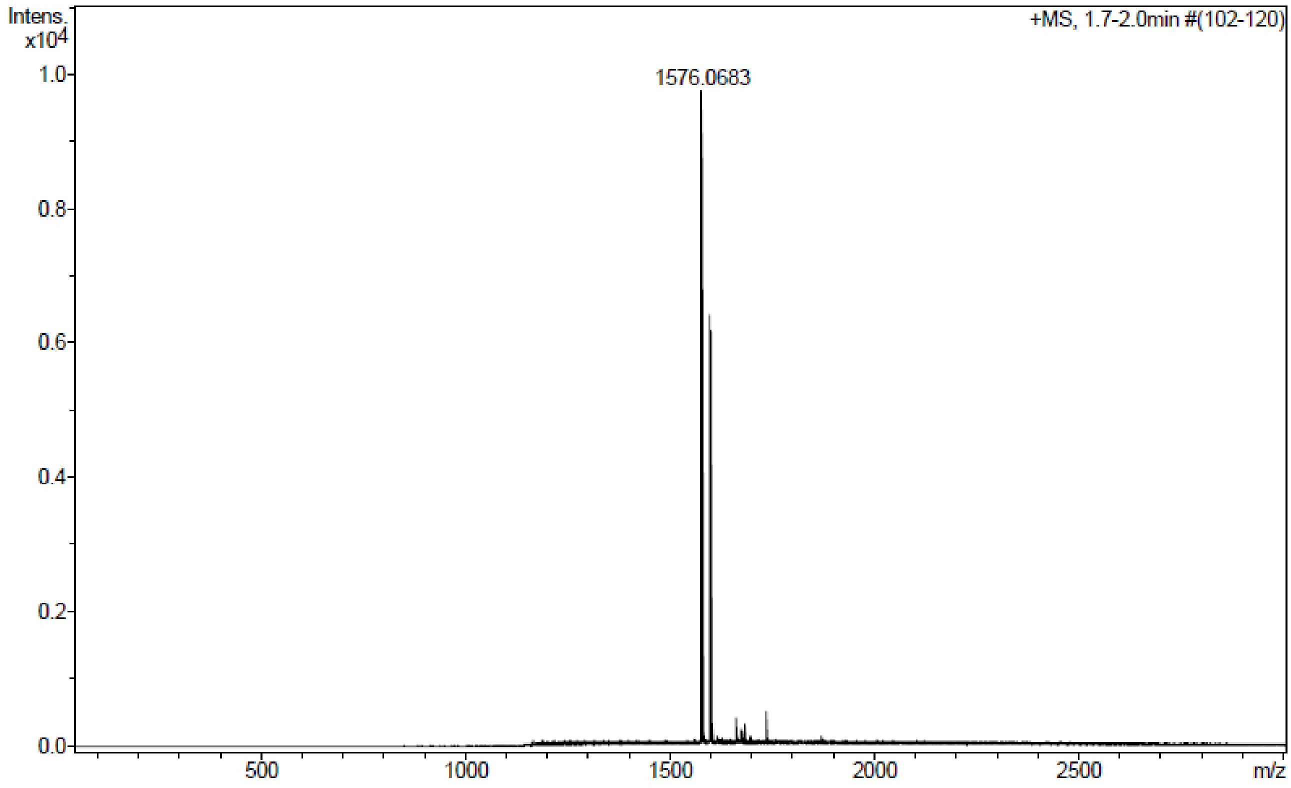
Intens.
x10^4
+MS, 1.7-2.0min #(102-120)
1576.0683
1.0
0.8
0.6
0.4
0.2
0.0
500
1000
1500
2000
2500
m/z

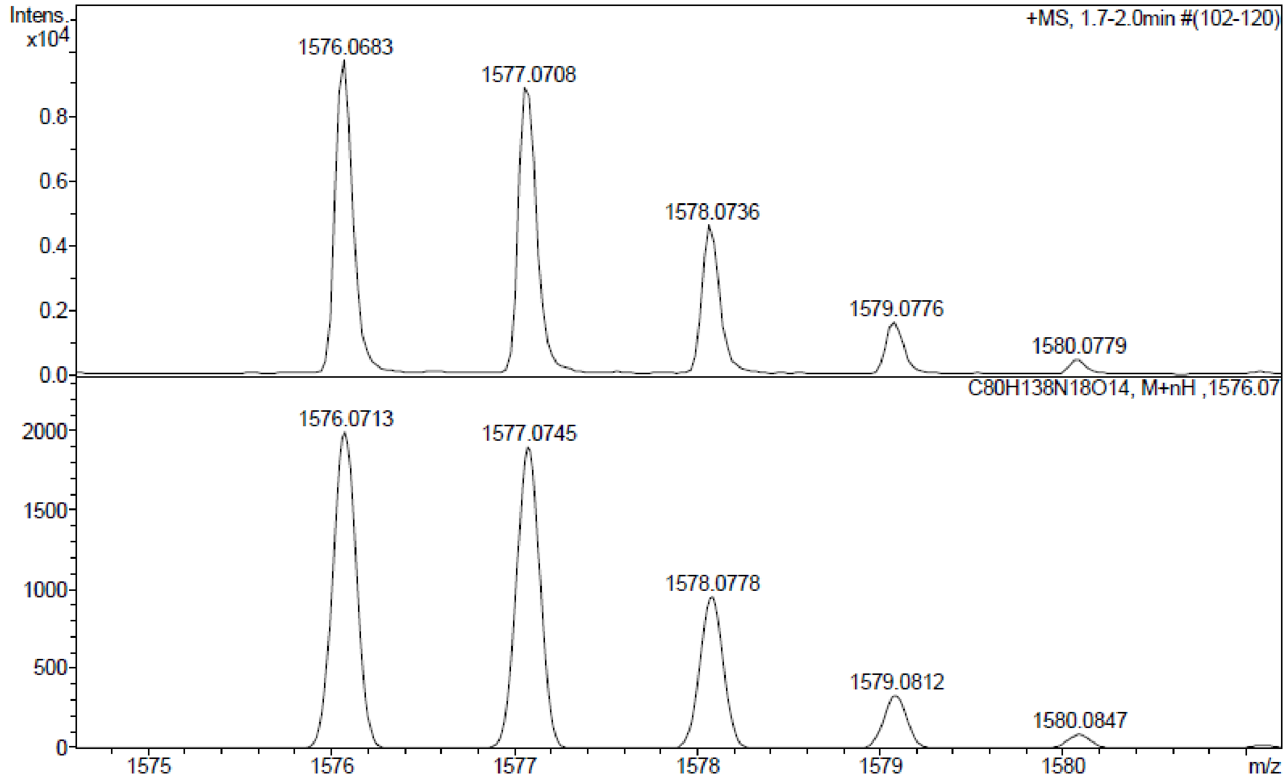
Intens.
x10^4
+MS, 1.7-2.0min #(102-120)
1576.0683
1577.0708
1578.0736
1579.0776
1580.0779
C80H138N18O14, M+nH ,1576.07
1576.0713
1577.0745
1578.0778
1579.0812
1580.0847
0.8
0.6
0.4
0.2
0.0
2000
1500
1000
500
0
1575
1576
1577
1578
1579
1580
m/z

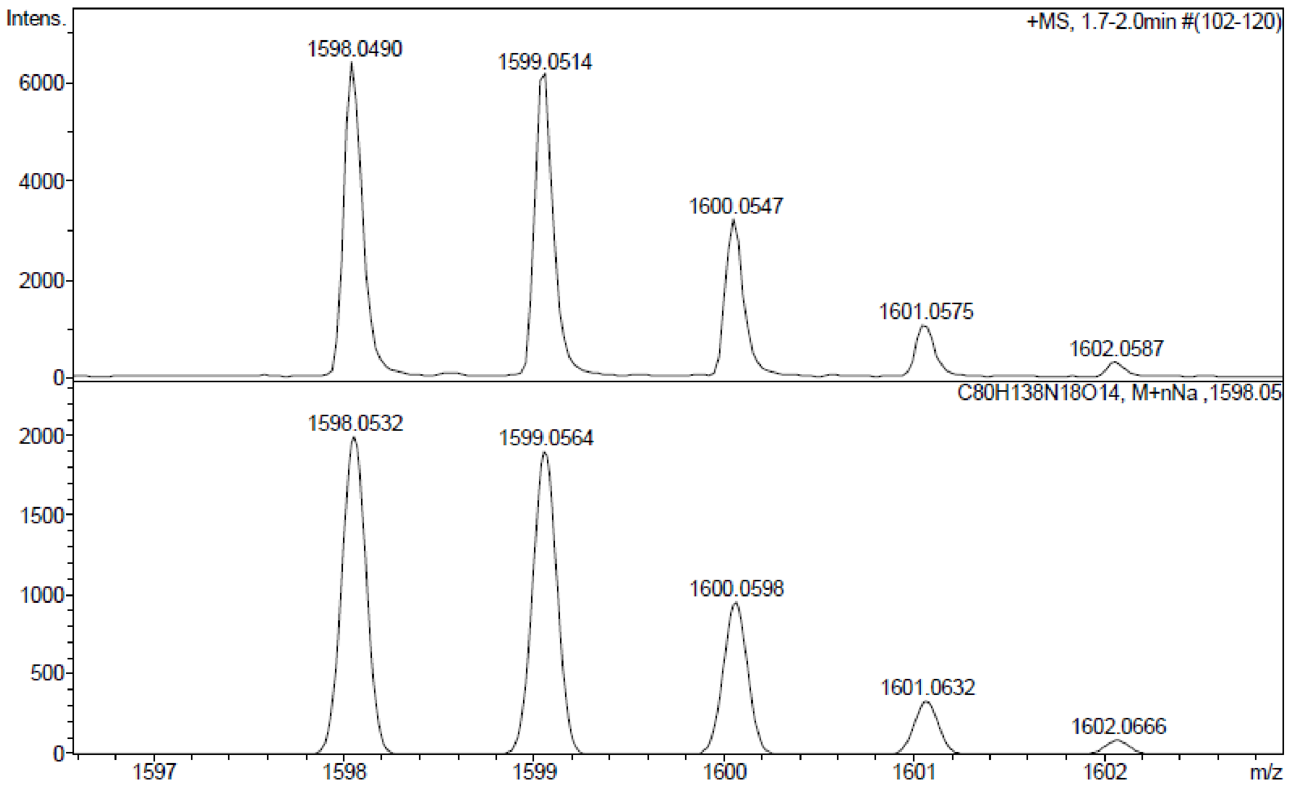
Intens.
+MS, 1.7-2.0min #(102-120)
1598.0490
1599.0514
1600.0547
1601.0575
1602.0587
C80H138N18O14, M+nNa ,1598.05
1598.0532
1599.0564
1600.0598
1601.0632
1602.0666
m/z

Ac-Lys-Lys-Leu-D-Phe-Lys-Lys-Ile-Leu-Lys-Tyr-Lys(COC$_5$H$_{11}$)-NH$_2$ (BP495)

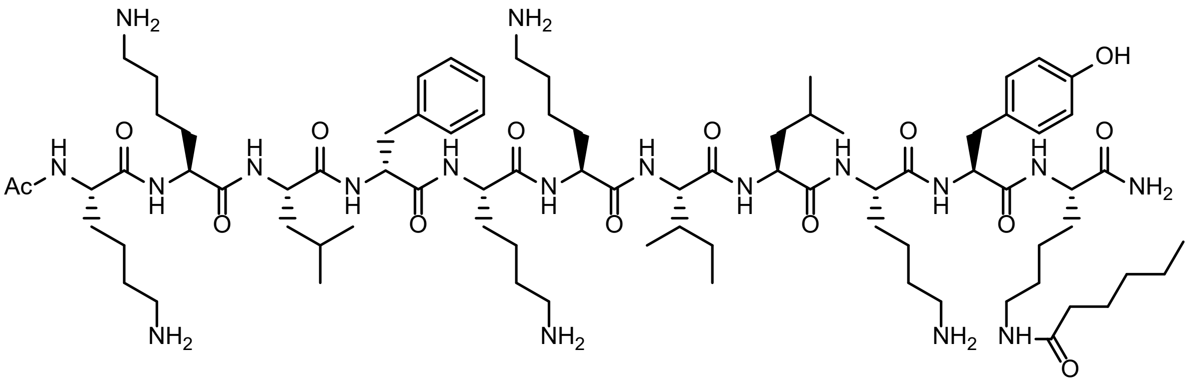

HPLC of crude peptide (λ=220 nm)

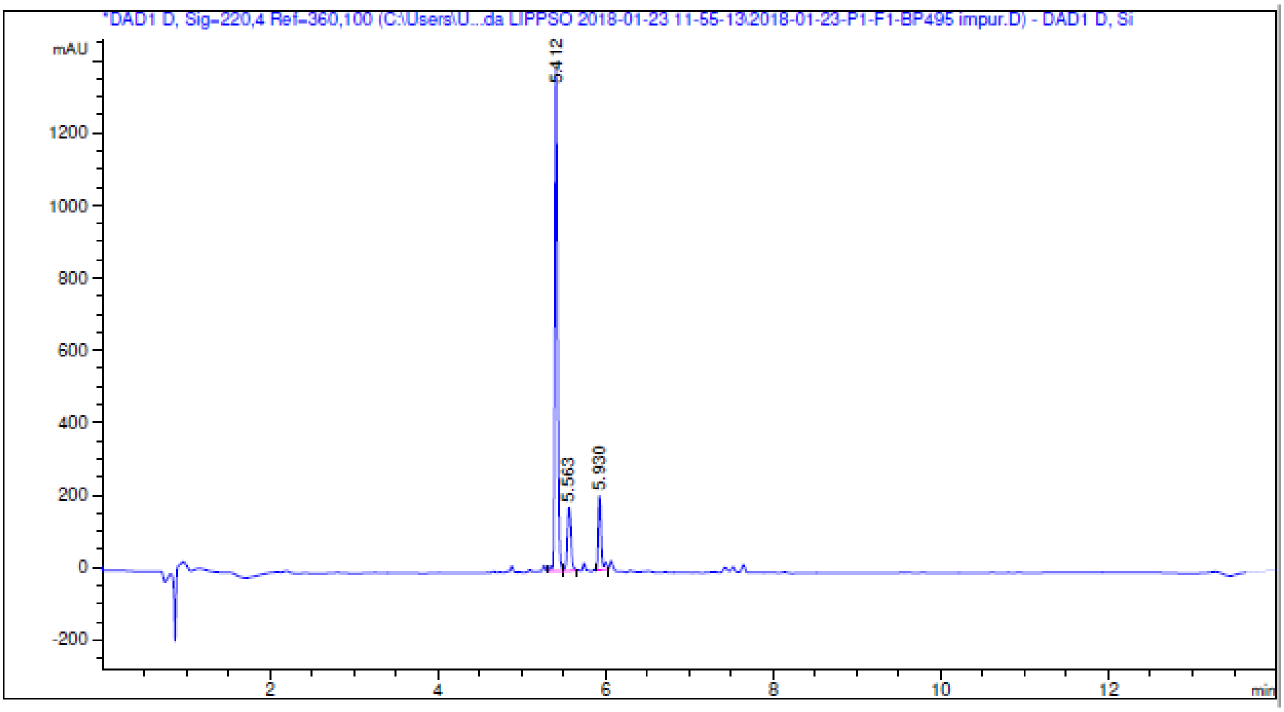

Peak #	RetTime [min]	Type	Width [min]	Area [mAU*s]	Height [mAU]	Area %
1	5.412	VV R	0.0356	3169.20239	1389.56665	75.7298
2	5.563	VB	0.0487	535.45313	174.93906	12.7949
3	5.930	BV R	0.0353	480.22589	204.92200	11.4753

Totals : 4184.88141 1769.42770

HPLC of purified peptide (λ=220 nm)

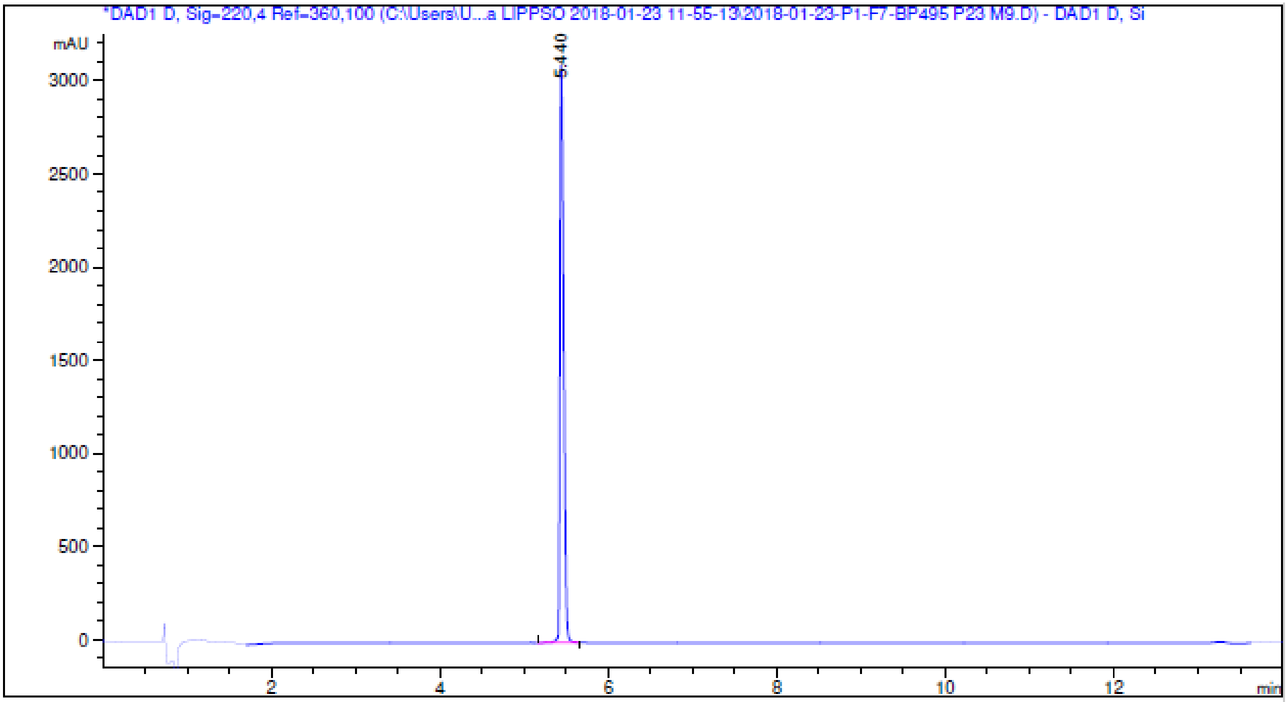

Peak #	RetTime [min]	Type	Width [min]	Area [mAU*s]	Height [mAU]	Area %
1	5.440	VB R	0.0545	1.04920e4	3101.66650	100.0000
Totals :				1.04920e4	3101.66650	

ESI-MS (*m/z*)

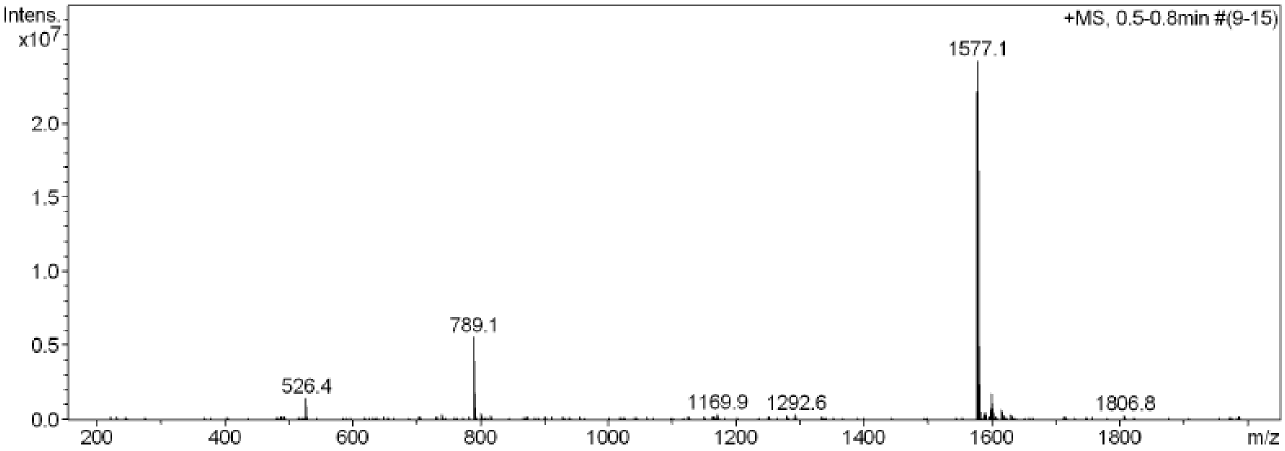

HRMS (*m/z*)

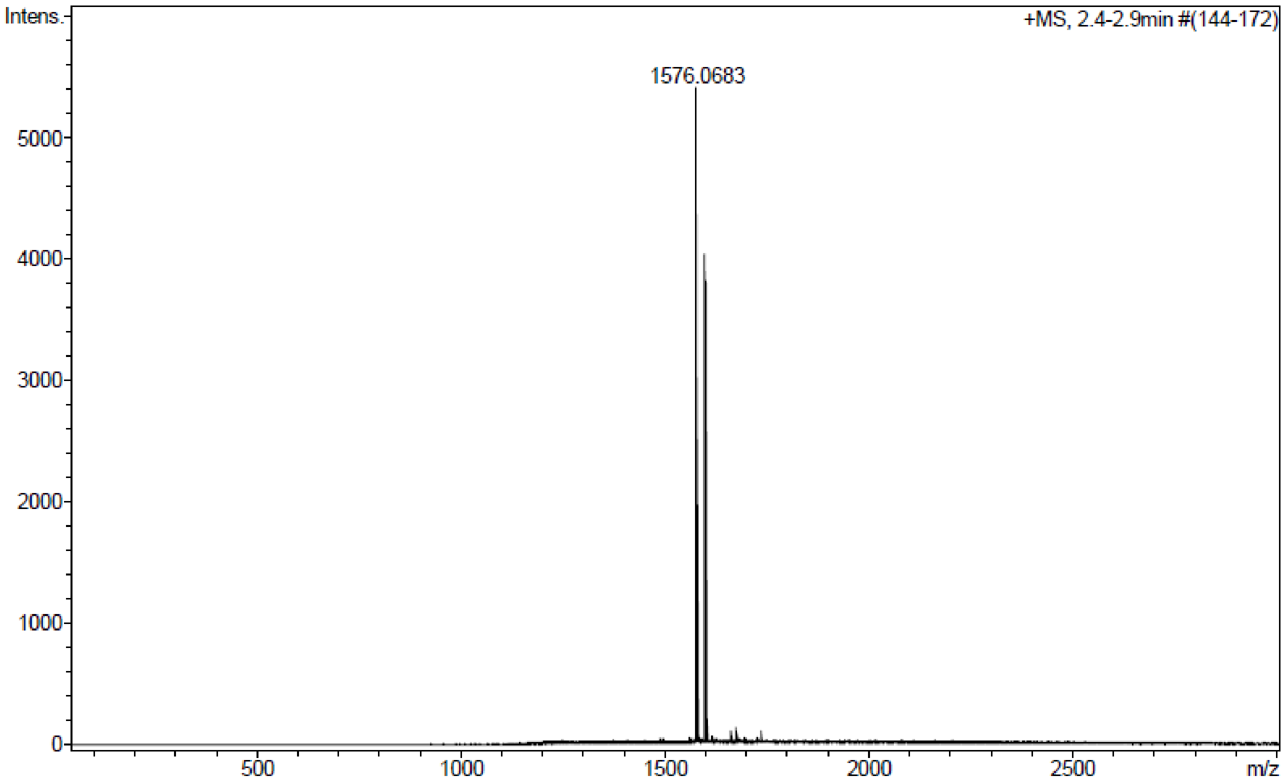
Intens.
+MS, 2.4-2.9min #(144-172)
1576.0683
5000
4000
3000
2000
1000
0
500
1000
1500
2000
2500
m/z

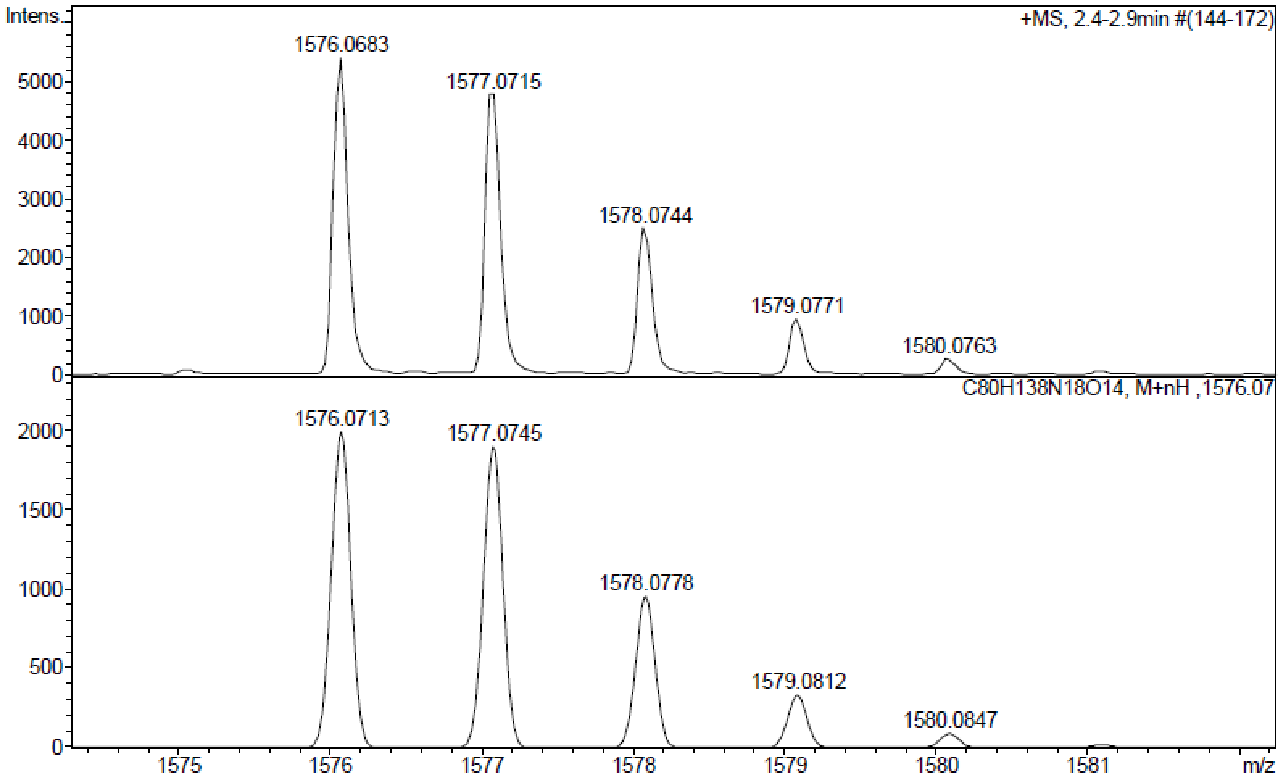
Intens.
+MS, 2.4-2.9min #(144-172)
1576.0683
1577.0715
1578.0744
1579.0771
1580.0763
C80H138N18O14, M+nH ,1576.07
1576.0713
1577.0745
1578.0778
1579.0812
1580.0847
5000
4000
3000
2000
1000
0
2000
1500
1000
500
0
1575
1576
1577
1578
1579
1580
1581
m/z

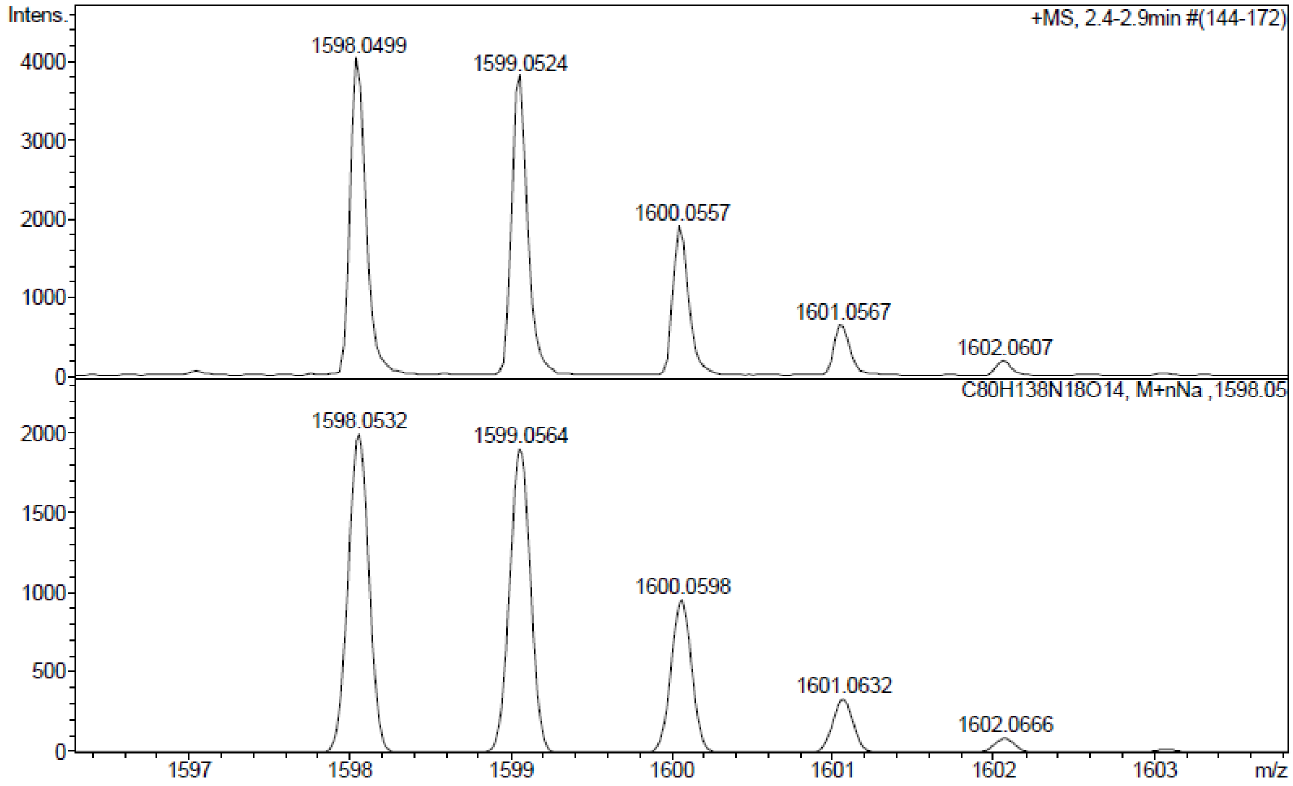

Intens.
+MS, 2.4-2.9min #(144-172)
4000
3000
2000
1000
0
1598.0499
1599.0524
1600.0557
1601.0567
1602.0607
C80H138N18O14, M+nNa ,1598.05
2000
1500
1000
500
0
1598.0532
1599.0564
1600.0598
1601.0632
1602.0666
1597
1598
1599
1600
1601
1602
1603
m/z

Ac-Lys-Lys-Leu-D-Phe-Lys-Lys-Ile-Leu-Lys-Tyr-Lys(COC_3H_7)-NH_2 (BP496)

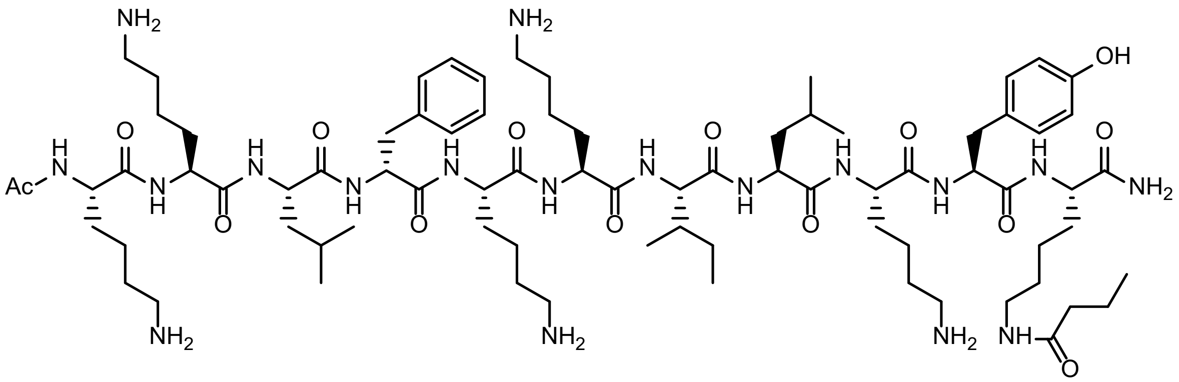

HPLC of crude peptide (λ=220 nm)

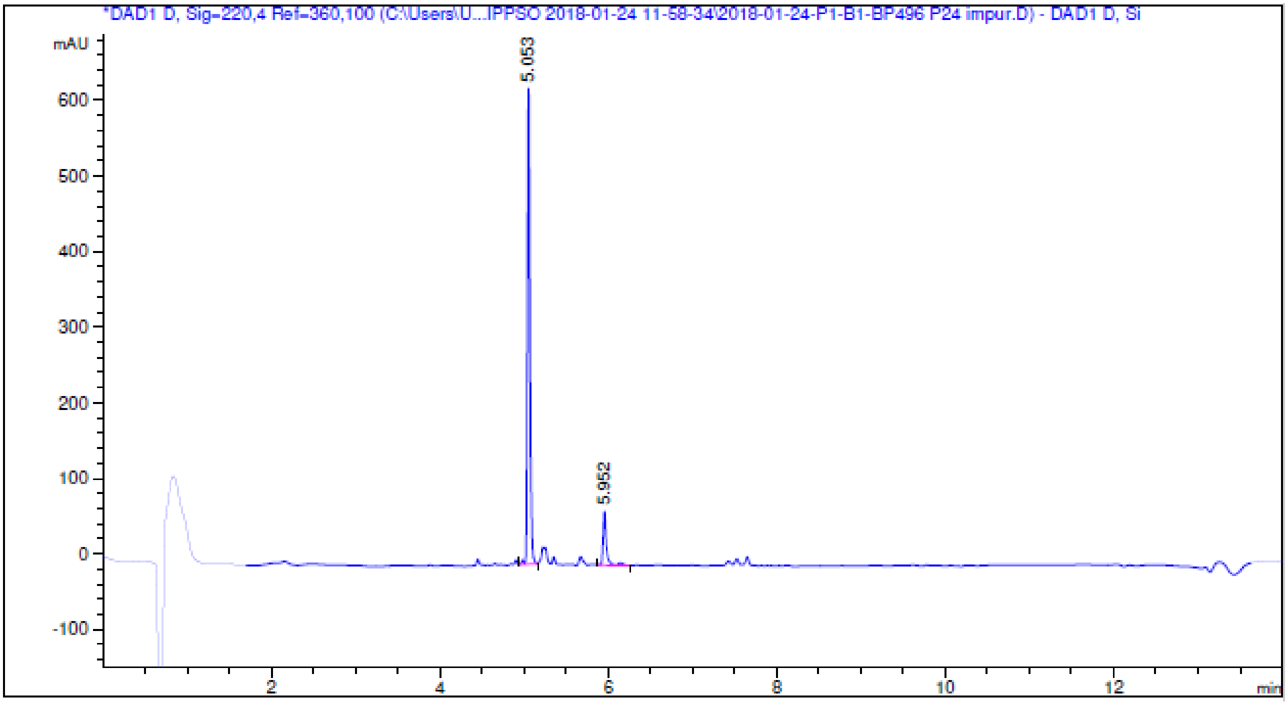

Peak #	RetTime [min]	Type	Width [min]	Area [mAU*s]	Height [mAU]	Area %
1	5.053	VB R	0.0325	1323.29224	630.92993	86.8886
2	5.952	VV R	0.0419	199.68292	70.85992	13.1114
Totals :				1522.97516	701.78985	

HPLC of purified peptide (λ=220 nm)

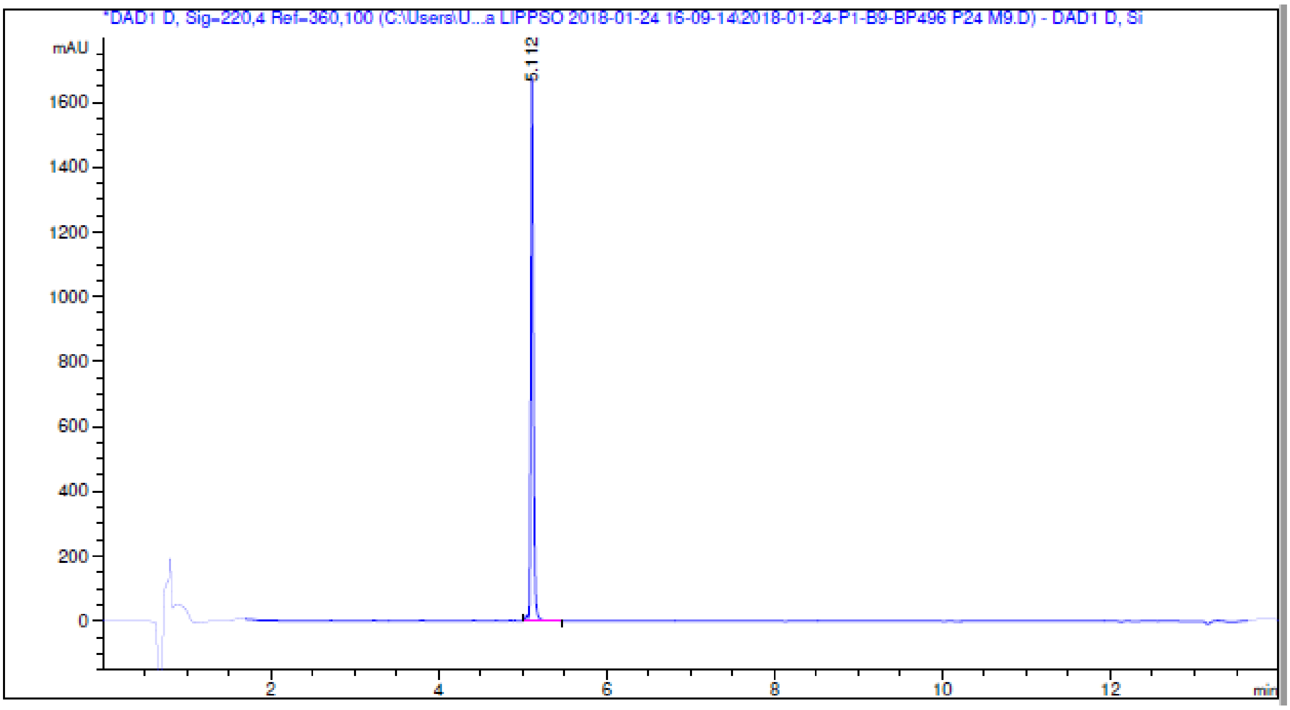

Peak #	RetTime [min]	Type	Width [min]	Area [mAU*s]	Height [mAU]	Area %
1	5.112	VV R	0.0361	3886.18408	1673.98193	100.0000
Totals :				3886.18408	1673.98193	

ESI-MS (*m/z*)

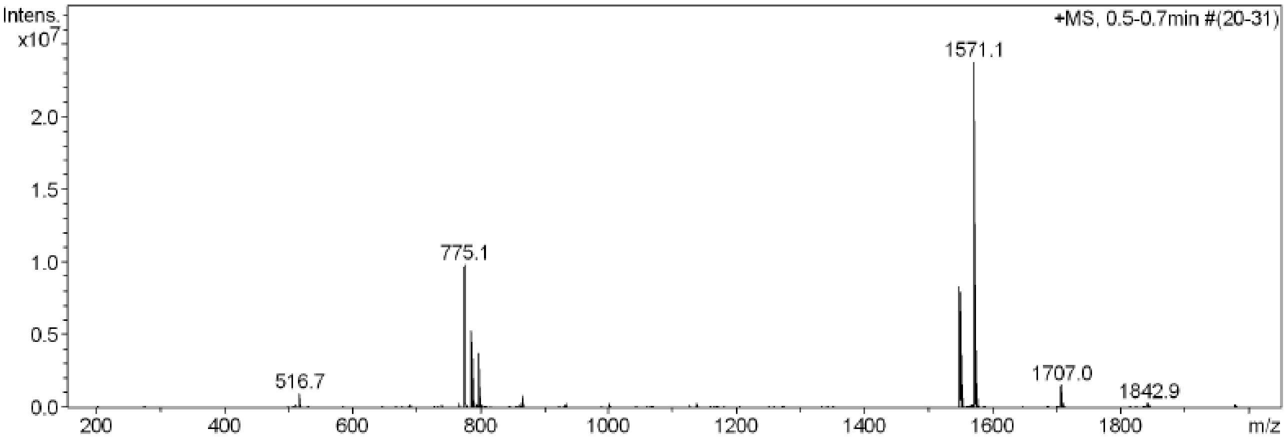

HRMS (*m/z*)

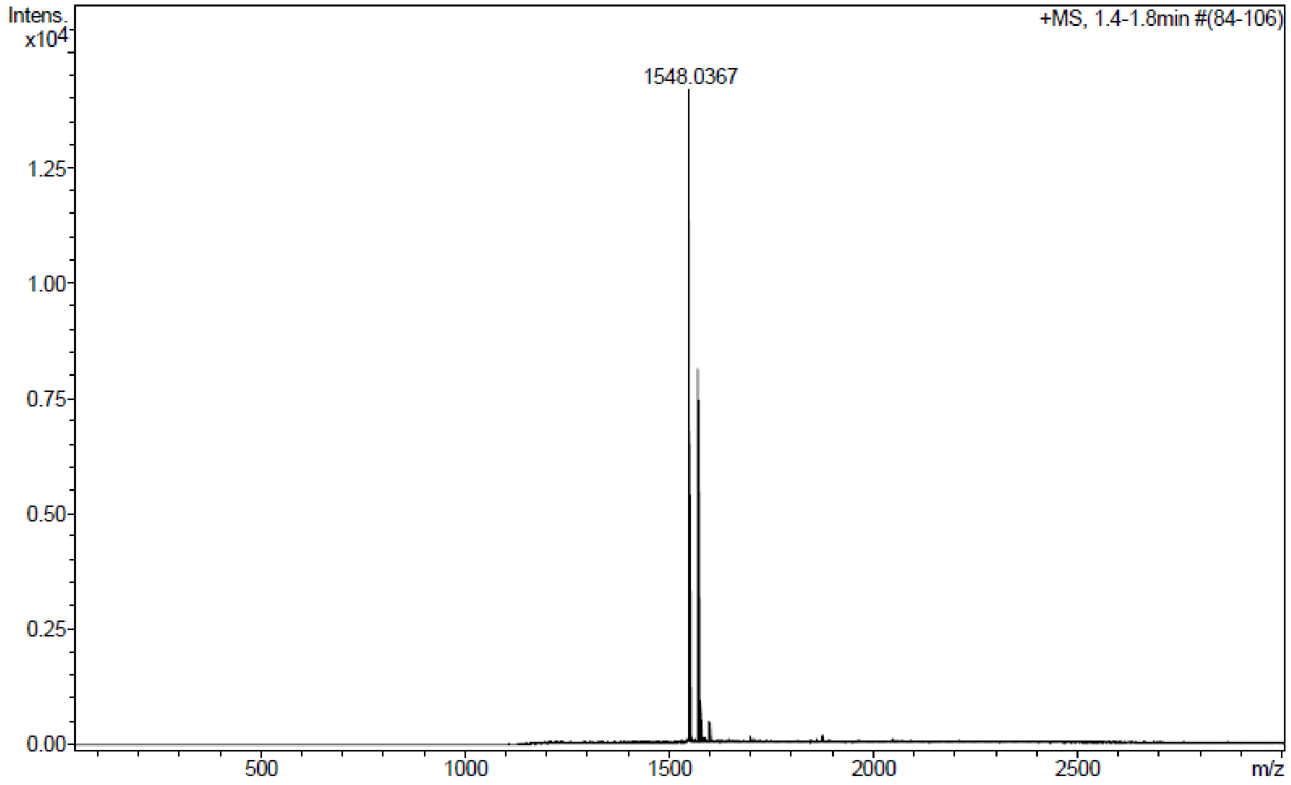

Intens.
x10^4
+MS, 1.4-1.8min #(84-106)
1548.0367
1.25
1.00
0.75
0.50
0.25
0.00
500
1000
1500
2000
2500
m/z

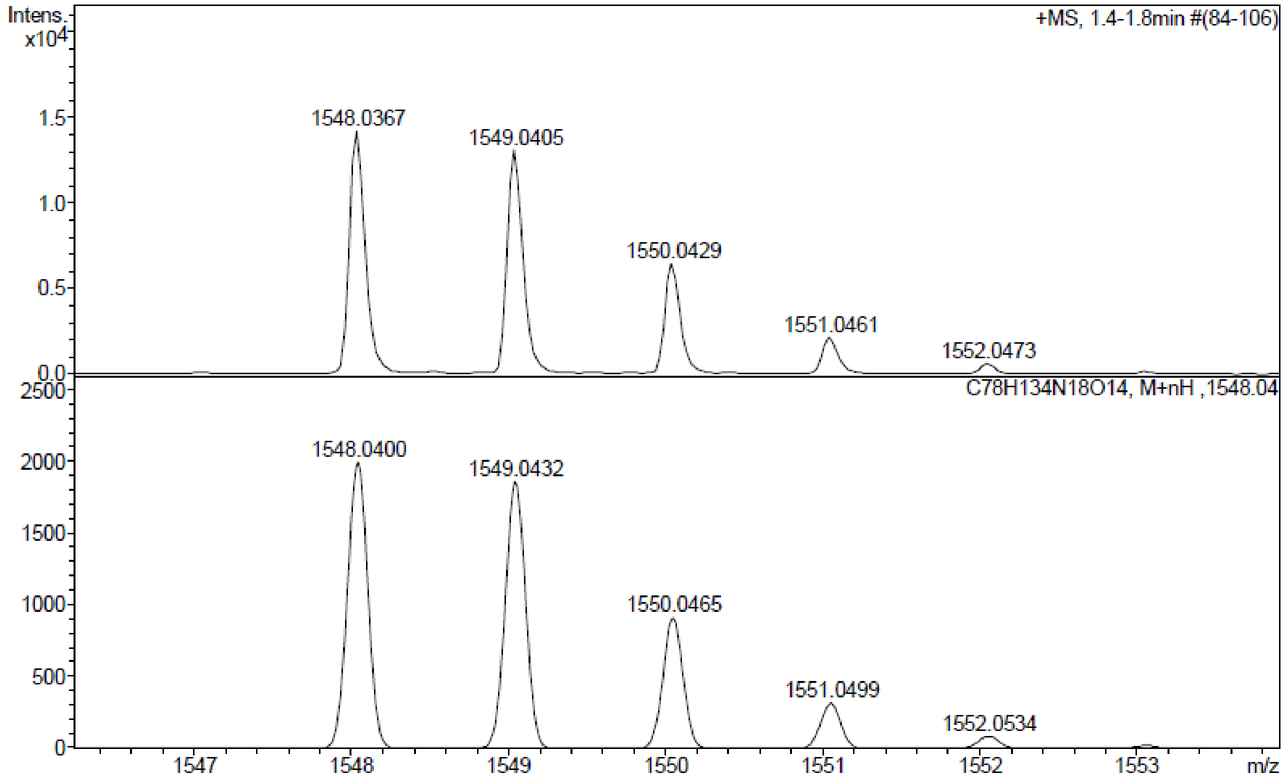

Intens.
x10^4
+MS, 1.4-1.8min #(84-106)
1548.0367
1549.0405
1550.0429
1551.0461
1552.0473
C78H134N18O14, M+nH ,1548.04
1548.0400
1549.0432
1550.0465
1551.0499
1552.0534
1.5
1.0
0.5
0.0
2500
2000
1500
1000
500
0
1547
1548
1549
1550
1551
1552
1553
m/z

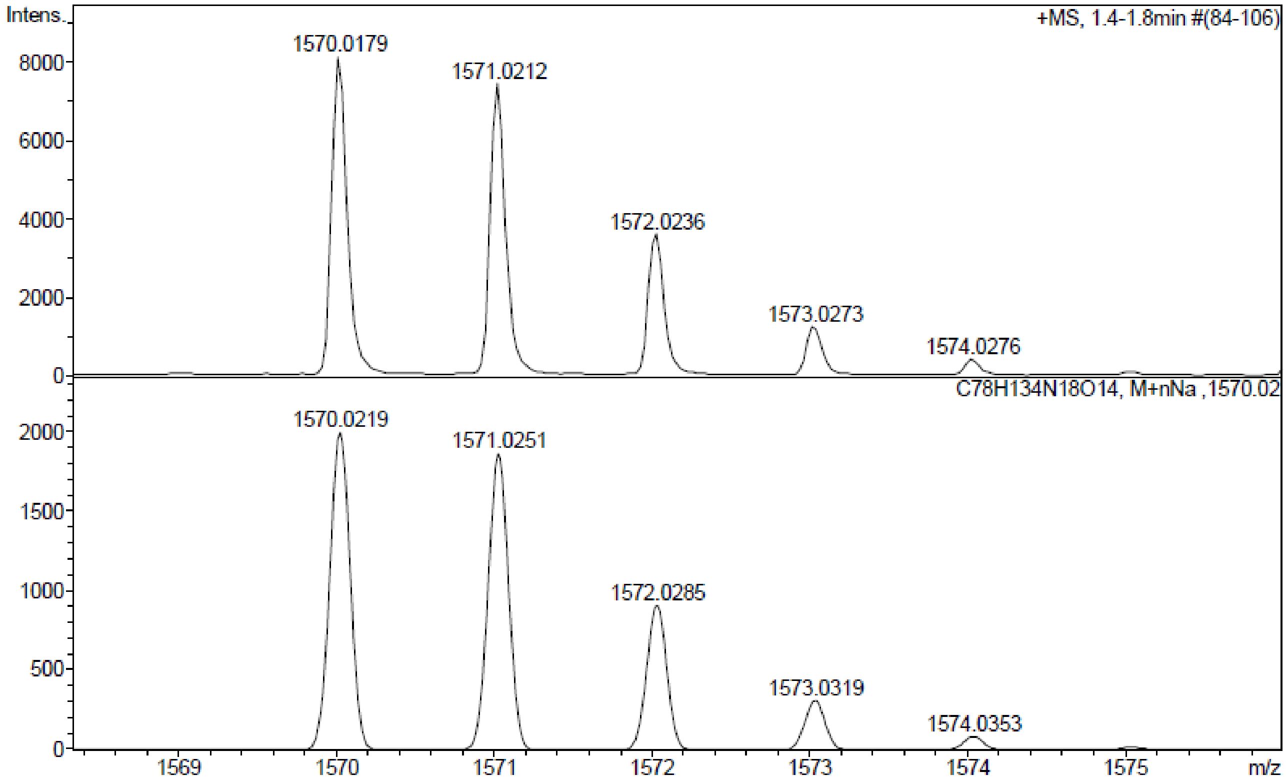
Intens.
+MS, 1.4-1.8min #(84-106)
1570.0179
1571.0212
1572.0236
1573.0273
1574.0276
8000
6000
4000
2000
0
C78H134N18O14, M+nNa ,1570.02
1570.0219
1571.0251
1572.0285
1573.0319
1574.0353
2000
1500
1000
500
0
1569
1570
1571
1572
1573
1574
1575
m/z

Ac-Lys-Lys-Leu-D-Phe-Lys-Lys-Ile-Leu-Lys($COC_{11}H_{23}$)-Tyr-Leu-NH_2 (BP497)

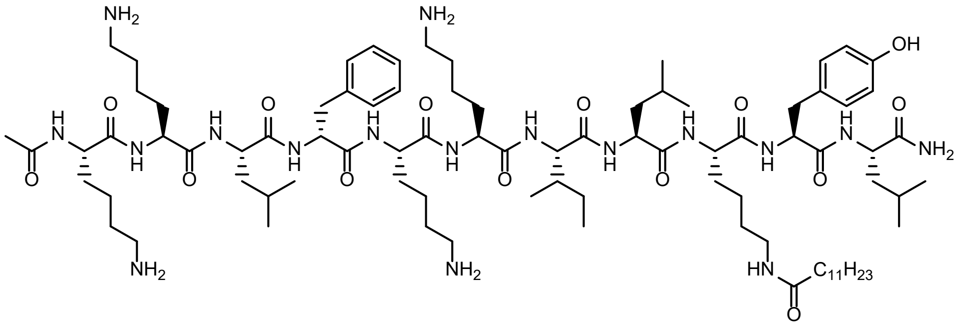

HPLC of crude peptide (λ=220 nm)

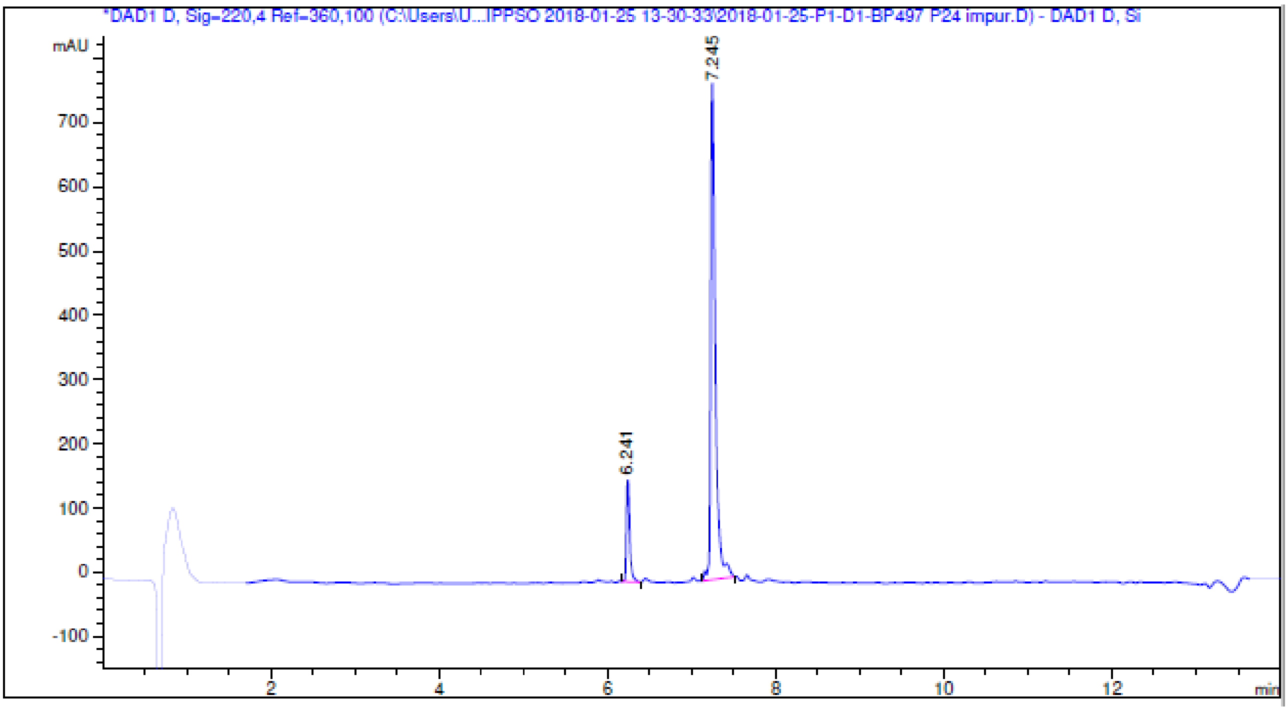

Peak #	RetTime [min]	Type	Width [min]	Area [mAU*s]	Height [mAU]	Area %
1	6.241	VB R	0.0399	431.05203	157.88124	12.7336
2	7.245	VV R	0.0556	2954.10278	772.97687	87.2664

Totals : 3385.15482 930.85811

HPLC of purified peptide (λ=220 nm)

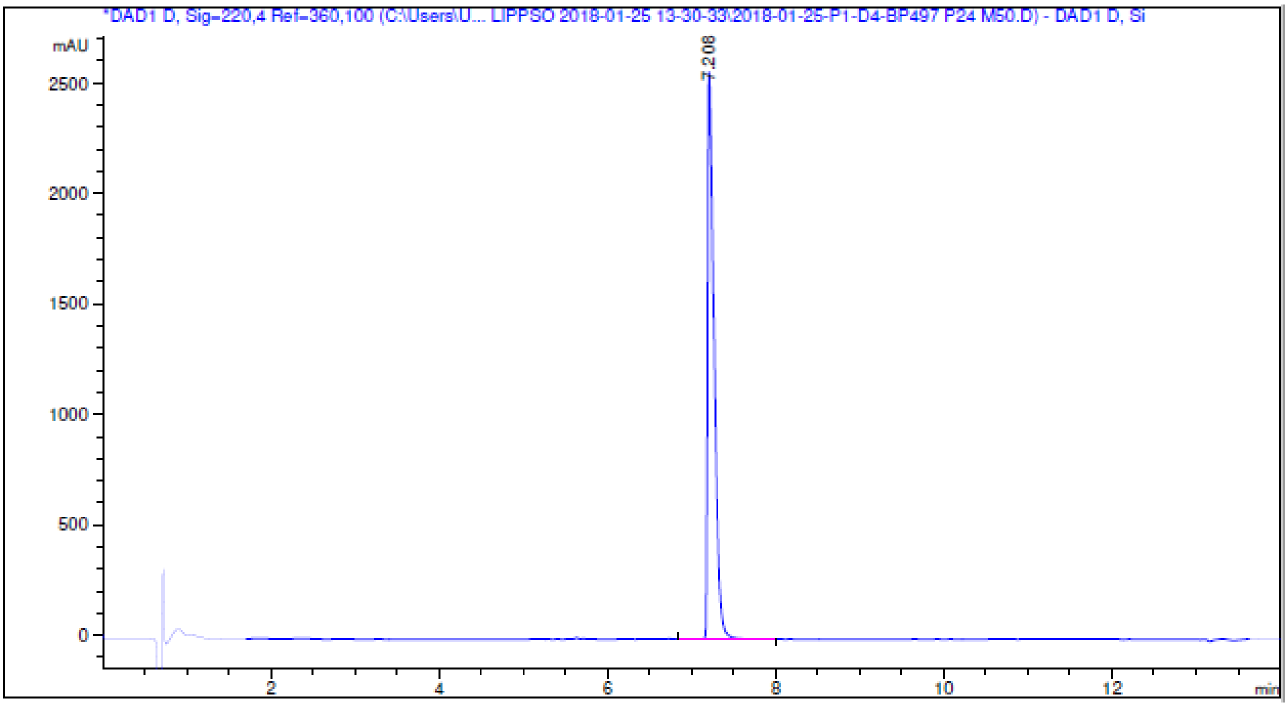

Peak #	RetTime [min]	Type	Width [min]	Area [mAU*s]	Height [mAU]	Area %
1	7.208	VB R	0.0804	1.37346e4	2568.37622	100.0000
Totals :				1.37346e4	2568.37622	

ESI-MS (*m/z*)

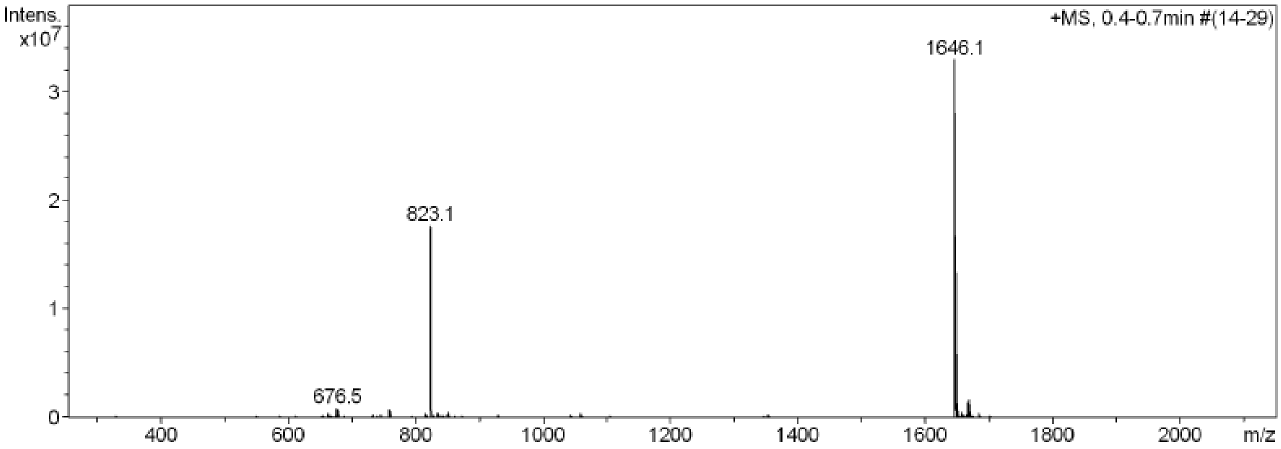

HRMS (m/z)

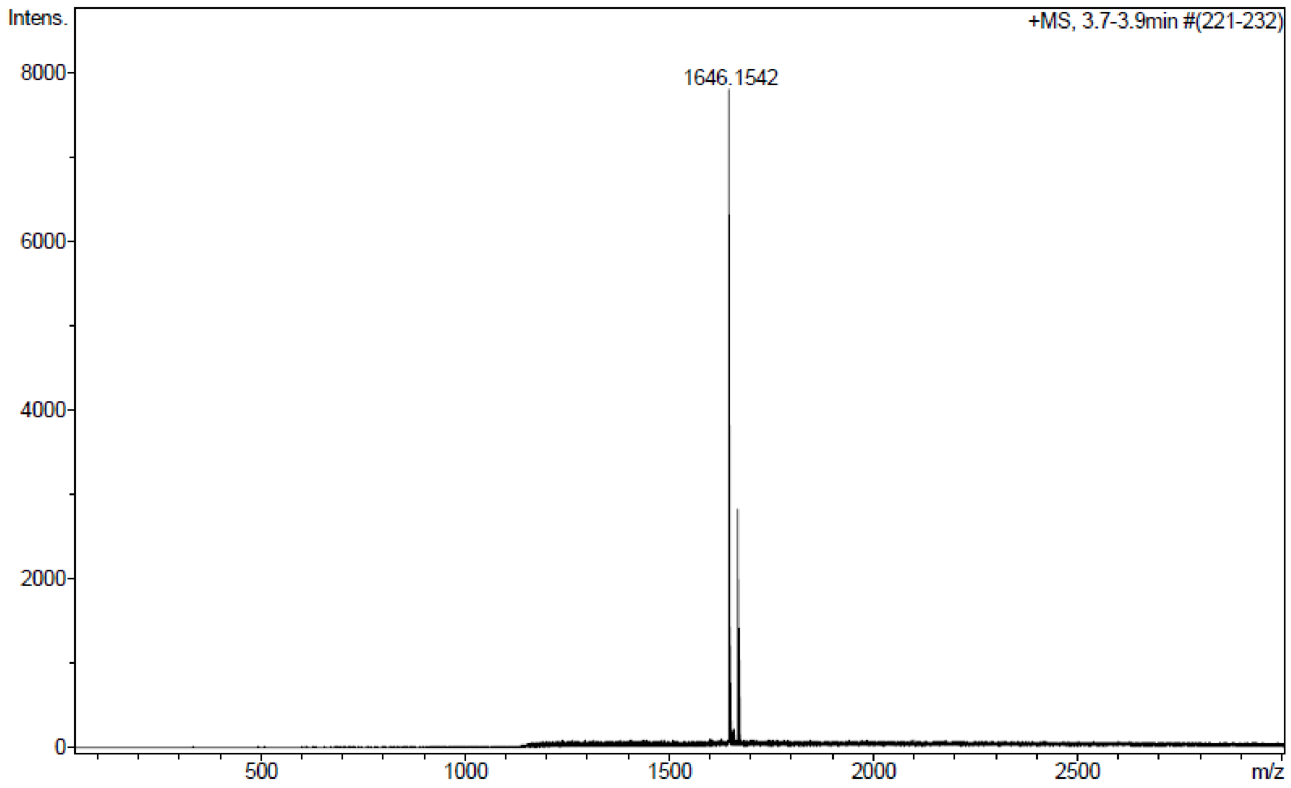

Intens.
+MS, 3.7-3.9min #(221-232)
1646.1542
8000
6000
4000
2000
0
500
1000
1500
2000
2500
m/z

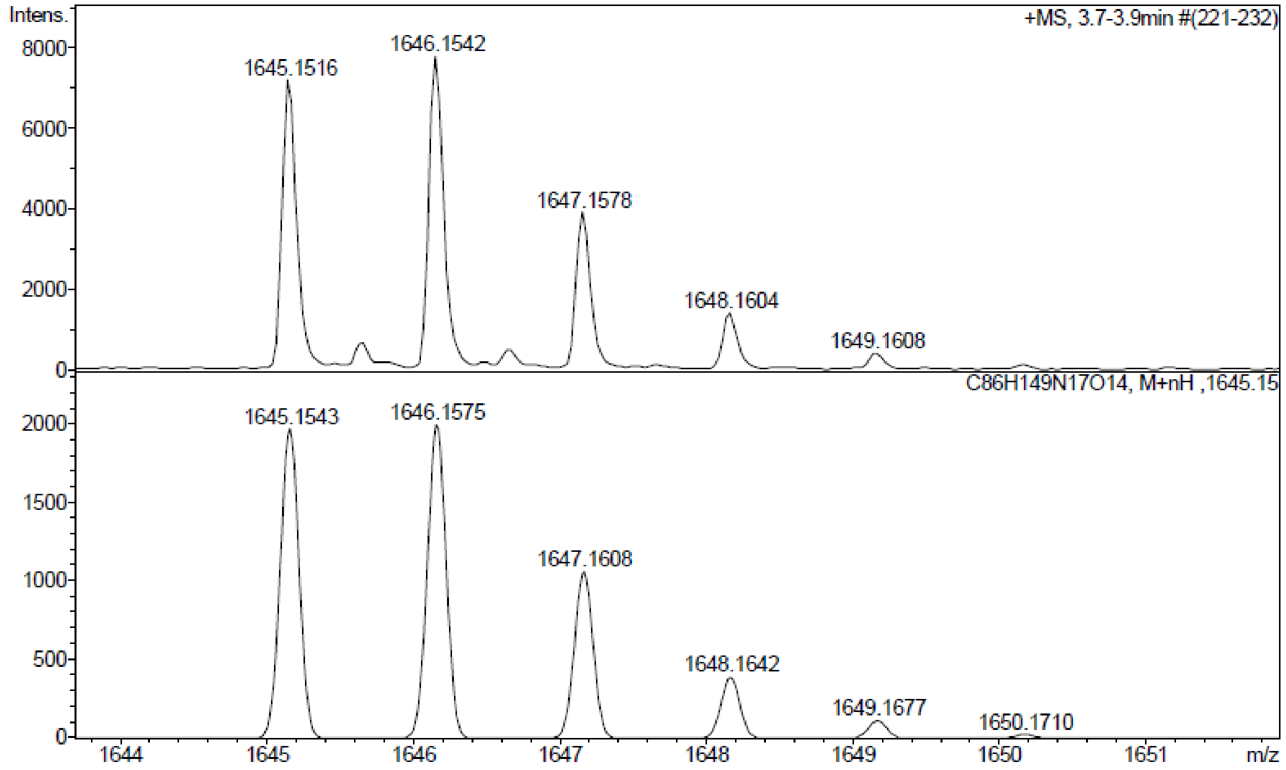

Intens.
+MS, 3.7-3.9min #(221-232)
1645.1516
1646.1542
1647.1578
1648.1604
1649.1608
C86H149N17O14, M+nH ,1645.15
1645.1543
1646.1575
1647.1608
1648.1642
1649.1677
1650.1710
1644
1645
1646
1647
1648
1649
1650
1651
m/z

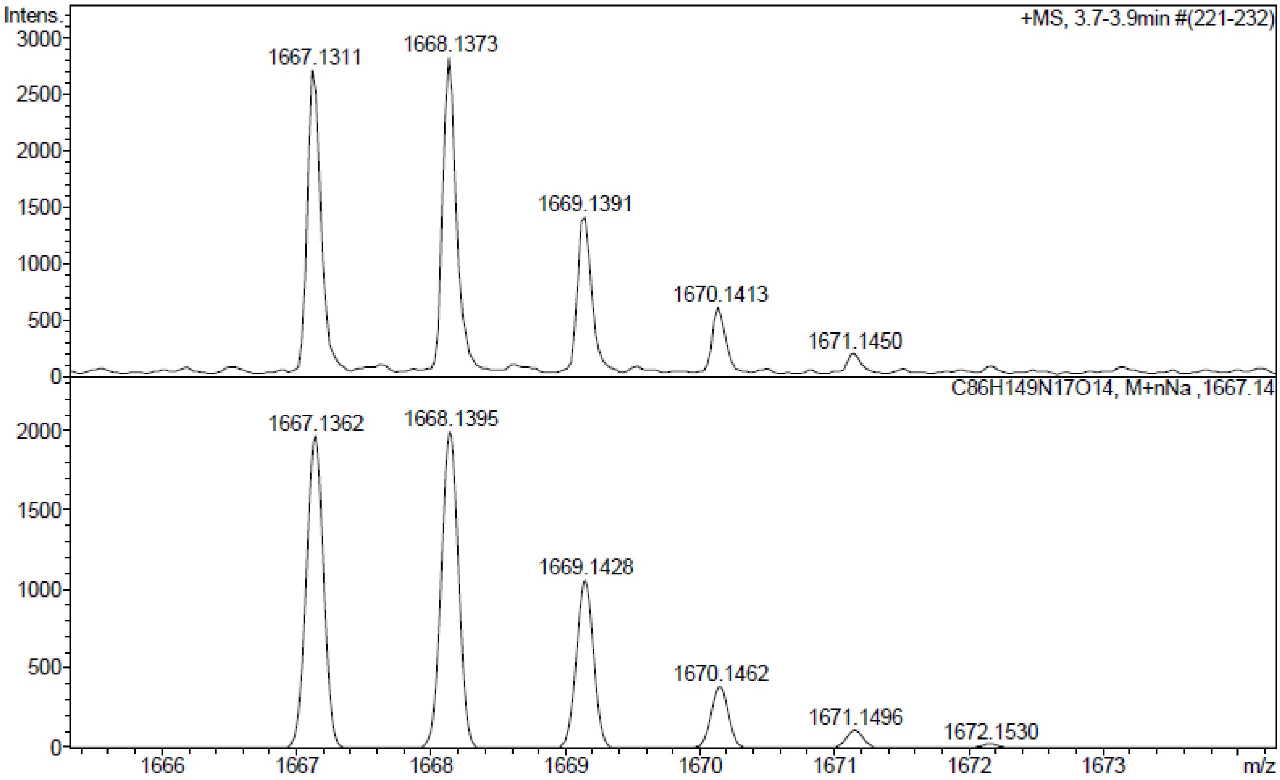
Intens.
+MS, 3.7-3.9min #(221-232)
1667.1311
1668.1373
1669.1391
1670.1413
1671.1450
C86H149N17O14, M+nNa ,1667.14
1667.1362
1668.1395
1669.1428
1670.1462
1671.1496
1672.1530
m/z

Ac-Lys-Lys-Leu-D-Phe-Lys(COC_3H_7)-Lys-Ile-Leu-Lys-Tyr-Leu-NH_2 (BP498)

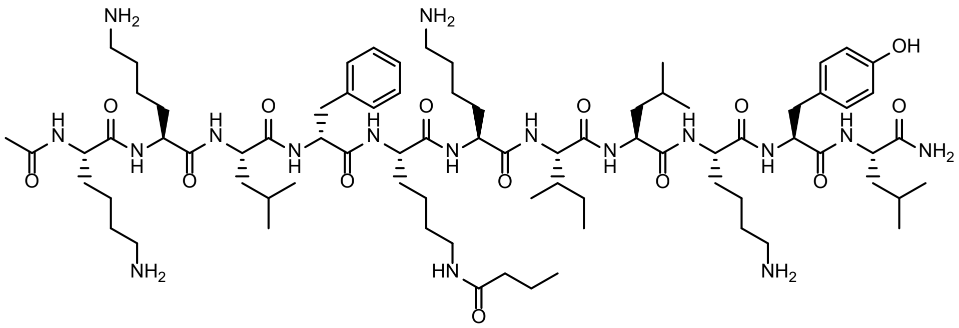

HPLC of crude peptide (λ=220 nm)

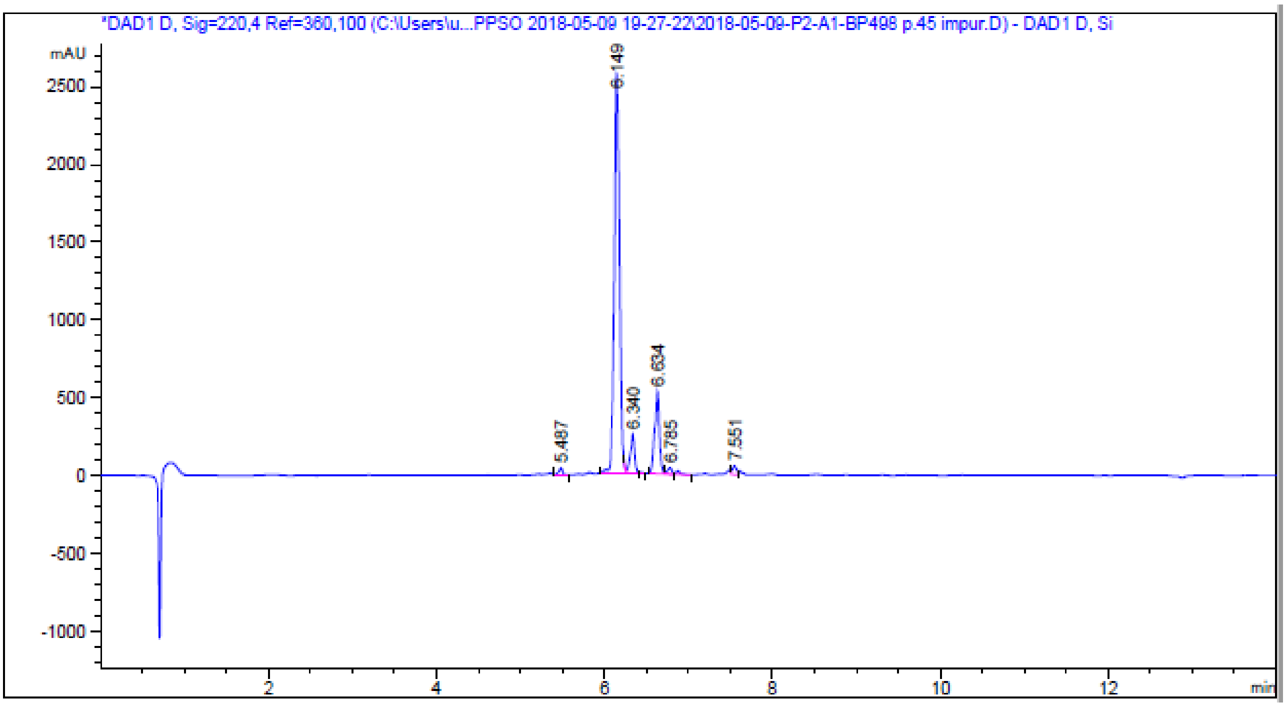

Peak #	RetTime [min]	Type	Width [min]	Area [mAU*s]	Height [mAU]	Area %
1	5.487	VB R	0.0464	148.45491	45.10777	1.0160
2	6.149	VV R	0.0632	1.10937e4	2578.84717	75.9247
3	6.340	VV E	0.0510	902.96692	250.56866	6.1799
4	6.634	BV R	0.0567	2112.34912	528.10651	14.4569
5	6.785	VV E	0.0489	152.63356	43.54723	1.0446
6	7.551	VV	0.0518	201.33051	57.53705	1.3779

Totals : 1.46114e4 3503.71439

HPLC of purified peptide (λ=220 nm)

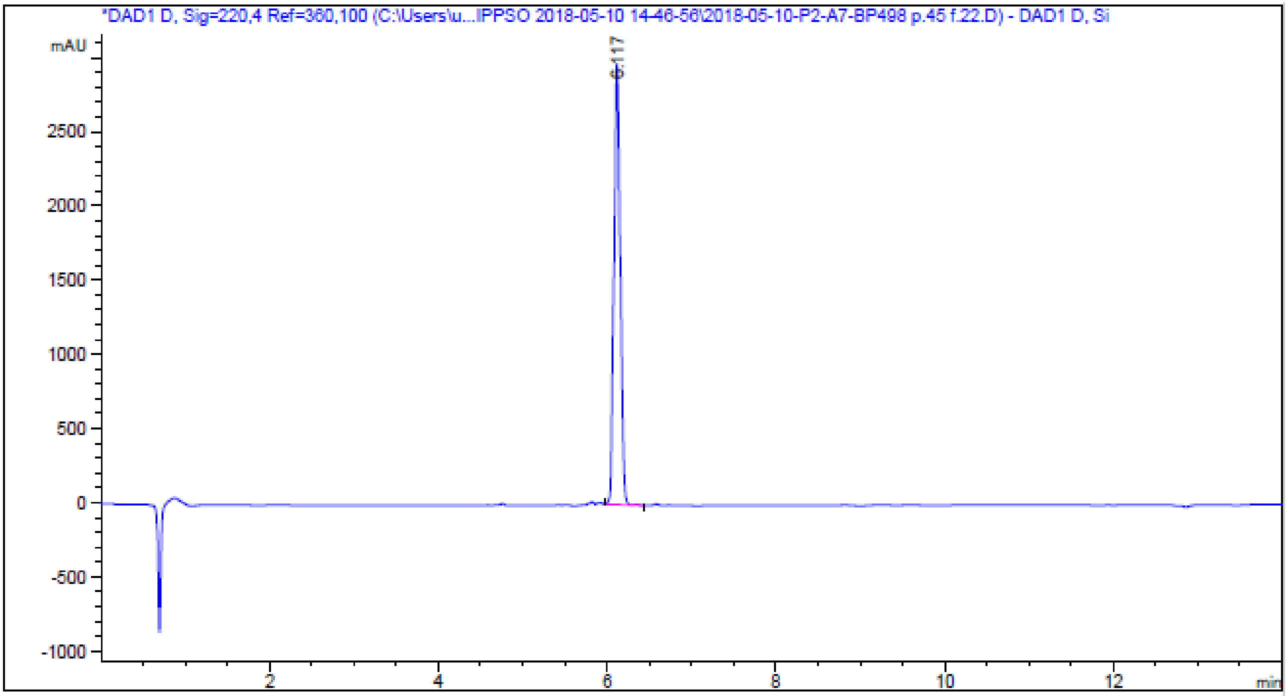

Peak #	RetTime [min]	Type	Width [min]	Area [mAU*s]	Height [mAU]	Area %
1	6.117	BV R	0.0755	1.51550e4	2973.04297	100.0000

Totals : 1.51550e4 2973.04297

ESI-MS (*m/z*)

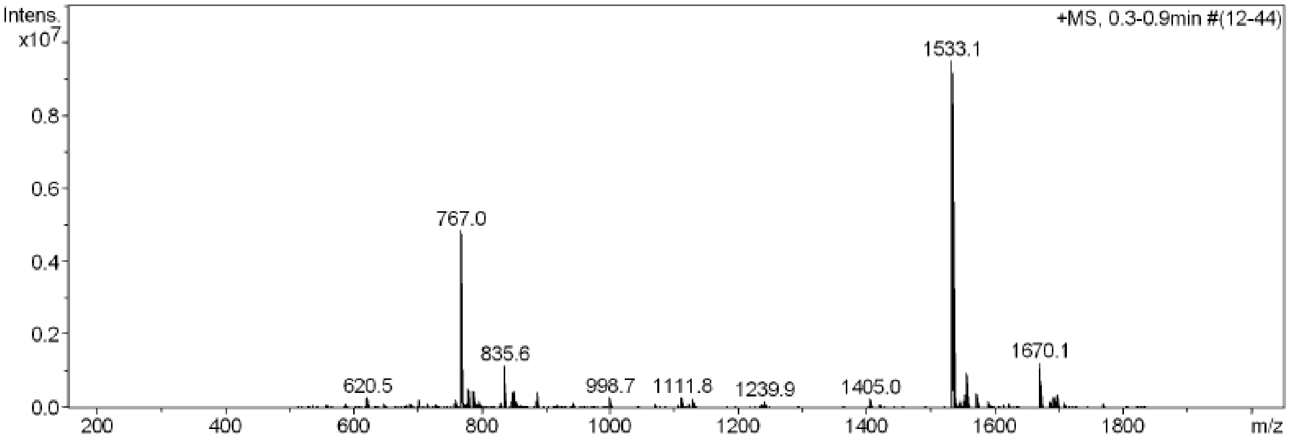

HRMS (*m/z*)

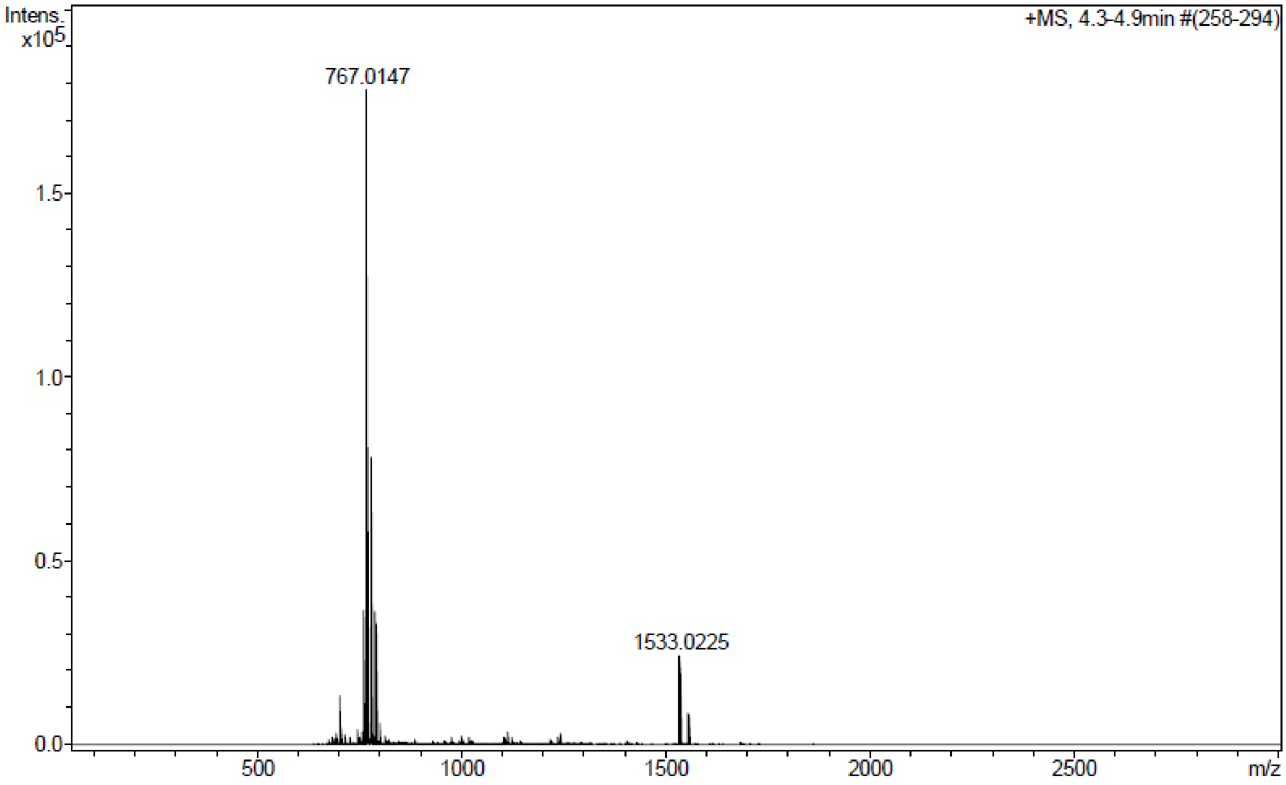

Intens.
x10^5
+MS, 4.3-4.9min #(258-294)
767.0147
1533.0225
1.5
1.0
0.5
0.0
500
1000
1500
2000
2500
m/z

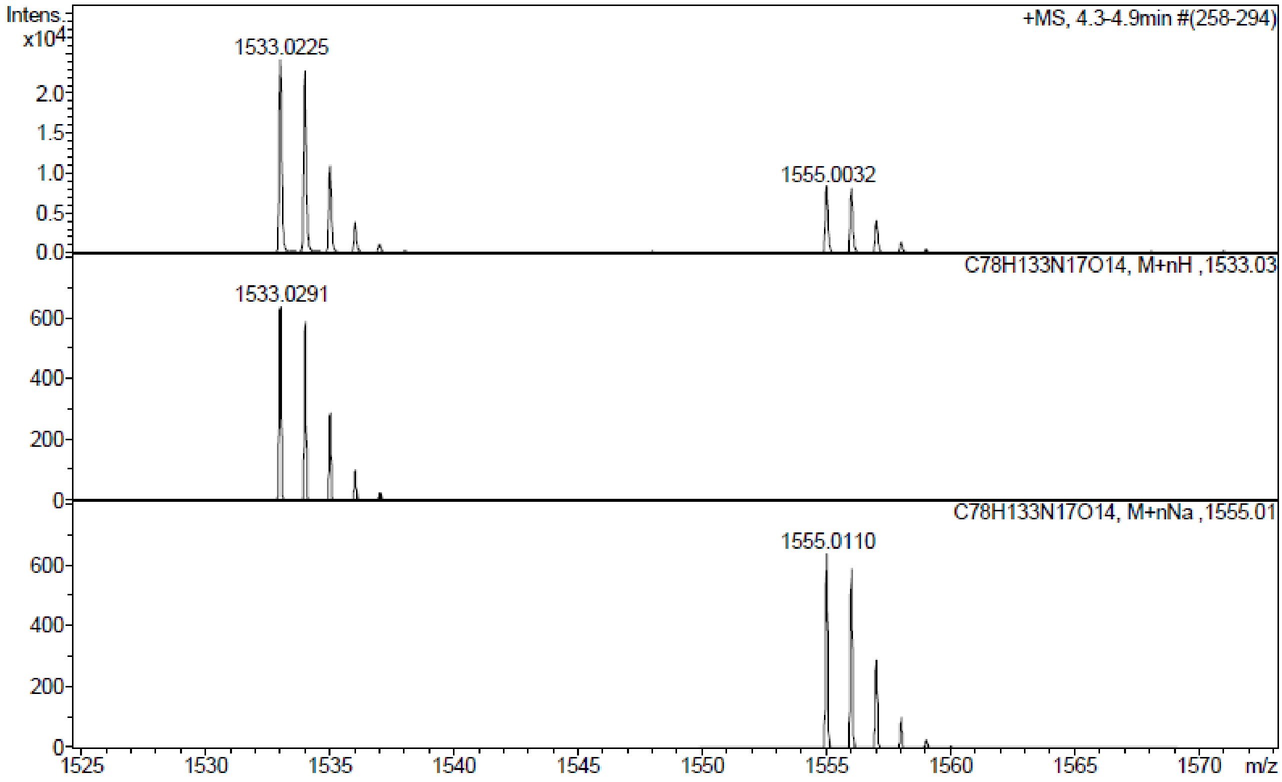

Intens.
x10^4
+MS, 4.3-4.9min #(258-294)
1533.0225
1555.0032
2.0
1.5
1.0
0.5
0.0
C78H133N17O14, M+nH ,1533.03
1533.0291
600
400
200
0
C78H133N17O14, M+nNa ,1555.01
1555.0110
1525
1530
1535
1540
1545
1550
1555
1560
1565
1570
m/z

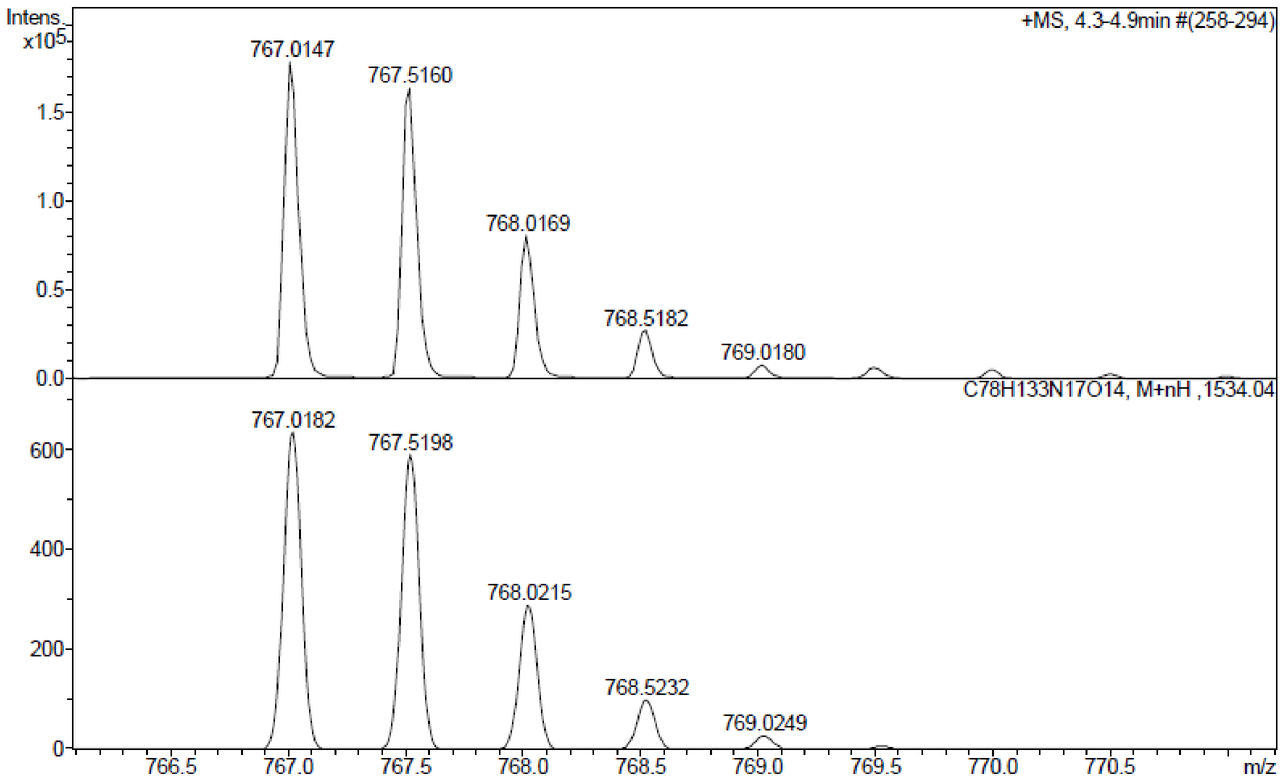
Intens.
x10^5
+MS, 4.3-4.9min #(258-294)
767.0147
767.5160
768.0169
768.5182
769.0180
C78H133N17O14, M+nH ,1534.04
767.0182
767.5198
768.0215
768.5232
769.0249
m/z

Ac-Lys-Lys-Leu-D-Phe-Lys-Lys-Ile-Leu-Lys(COC_3H_7)-Tyr-Leu-NH_2 (BP499)

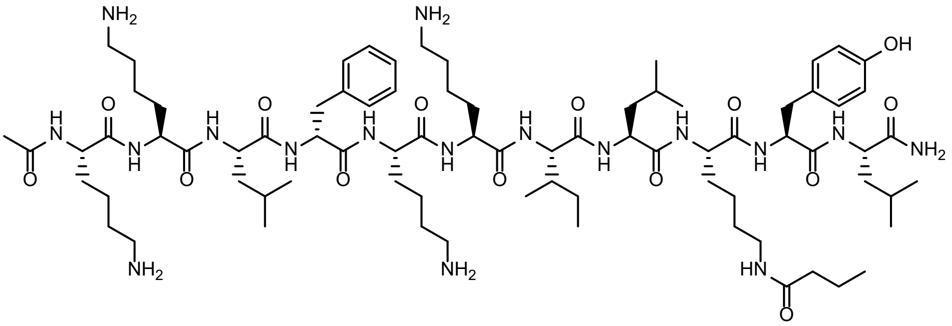

HPLC of crude peptide (λ=220 nm)

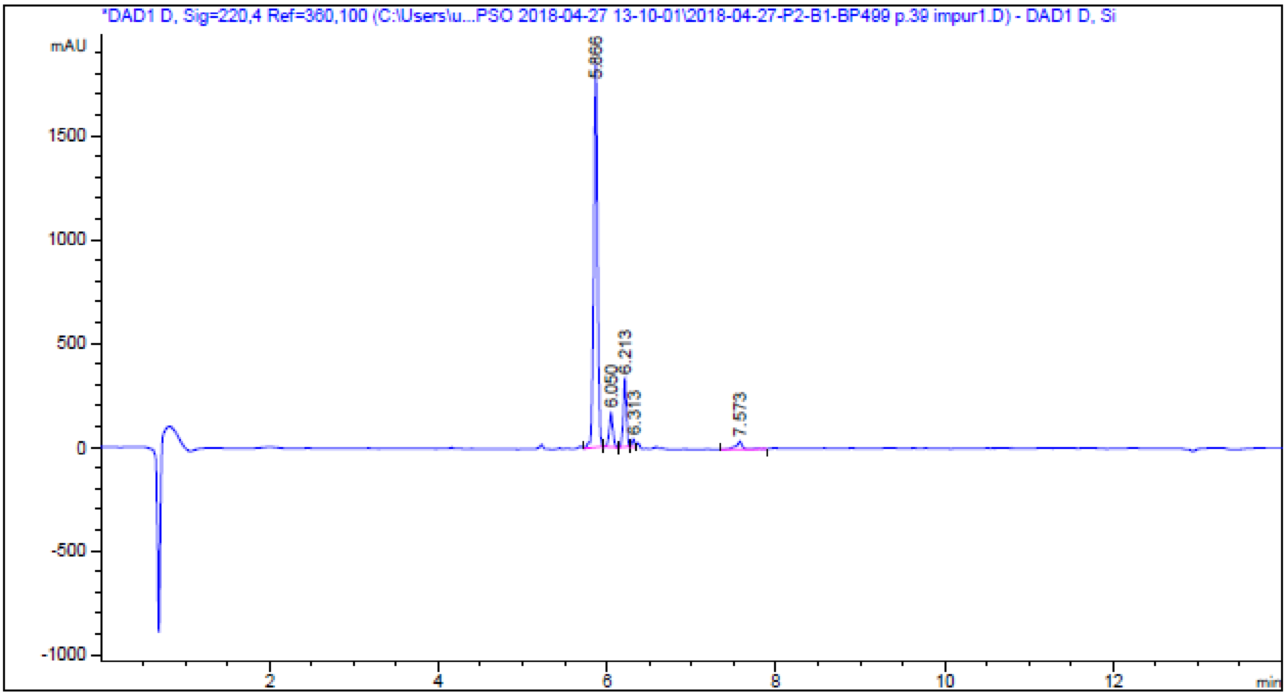

Peak #	RetTime [min]	Type	Width [min]	Area [mAU*s]	Height [mAU]	Area %
1	5.866	VB R	0.0443	5598.51953	1849.33667	77.5439
2	6.050	BB	0.0433	478.38898	162.94824	6.6261
3	6.213	BB	0.0390	849.00946	329.78500	11.7594
4	6.313	BV	0.0370	90.36411	37.69422	1.2516
5	7.573	BV R	0.0764	203.52594	36.29560	2.8190

Totals : 7219.80802 2416.05974

HPLC of purified peptide (λ=220 nm)

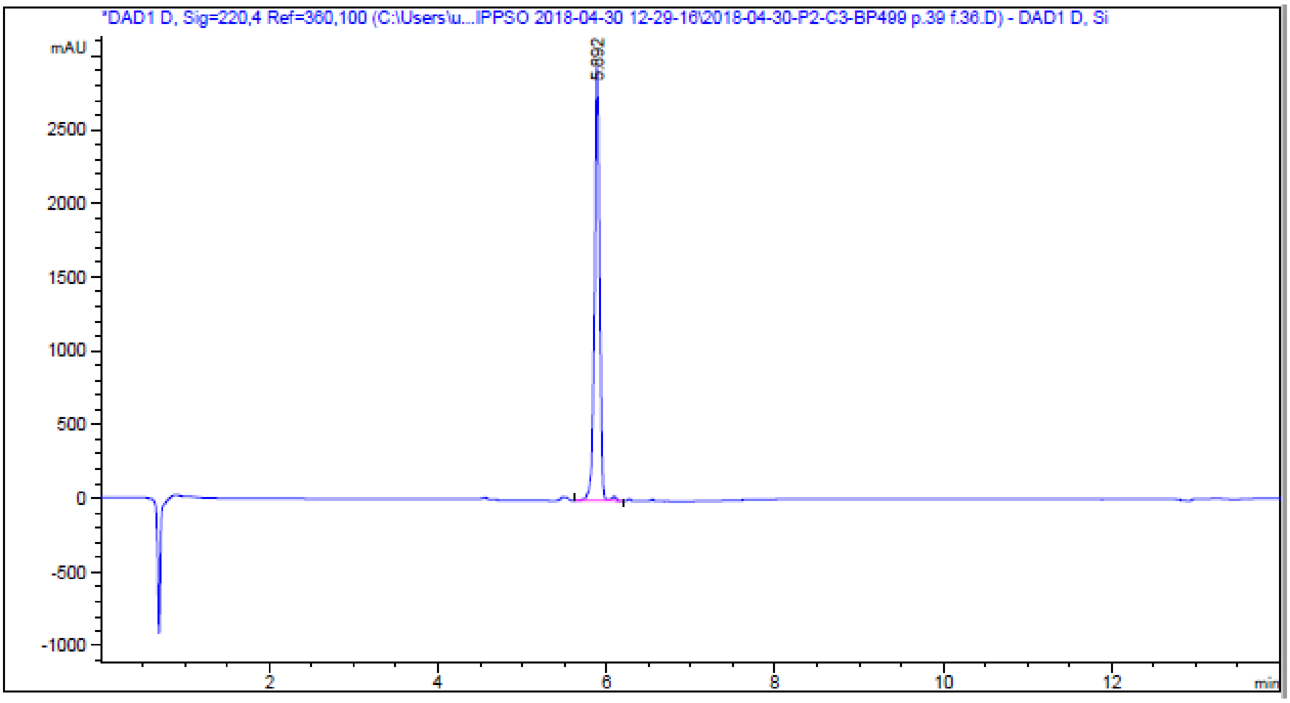

Peak #	RetTime [min]	Type	Width [min]	Area [mAU*s]	Height [mAU]	Area %
1	5.892	BV R	0.0617	1.23197e4	2951.55957	100.0000

Totals : 1.23197e4 2951.55957

ESI-MS (*m/z*)

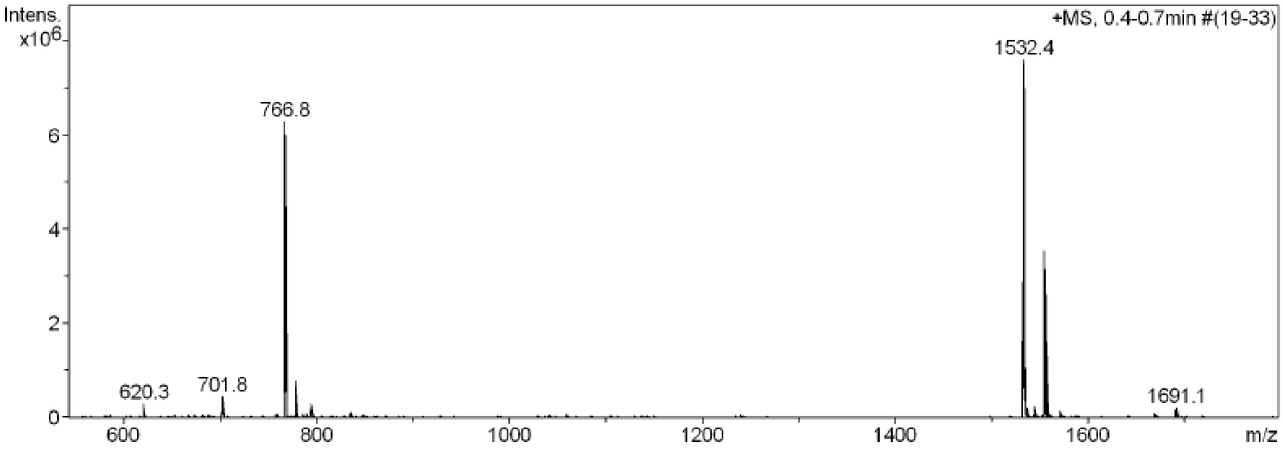

HRMS (m/z)

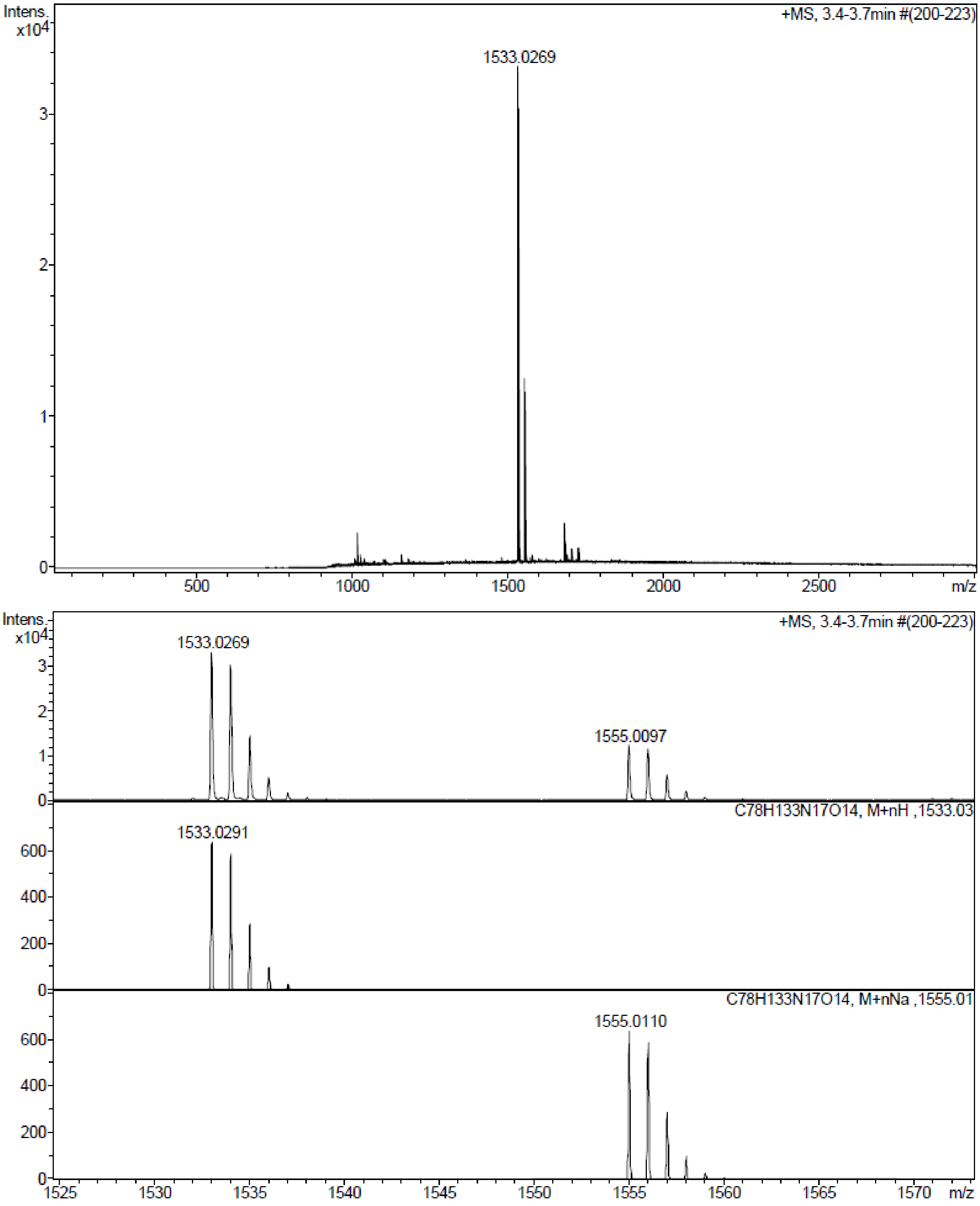

Intens.
x10^4
+MS, 3.4-3.7min #(200-223)
1533.0269
3
2
1
0
500
1000
1500
2000
2500
m/z
Intens.
x10^4
+MS, 3.4-3.7min #(200-223)
1533.0269
1555.0097
C78H133N17O14, M+nH ,1533.03
1533.0291
600
400
200
C78H133N17O14, M+nNa ,1555.01
1555.0110
1525
1530
1535
1540
1545
1550
1555
1560
1565
1570
m/z

Ac-Lys-Lys($COC_{11}H_{23}$)-Leu-D-Phe-Lys-Lys-Ile-Leu-Lys-Tyr-Leu-NH_2 (BP500)

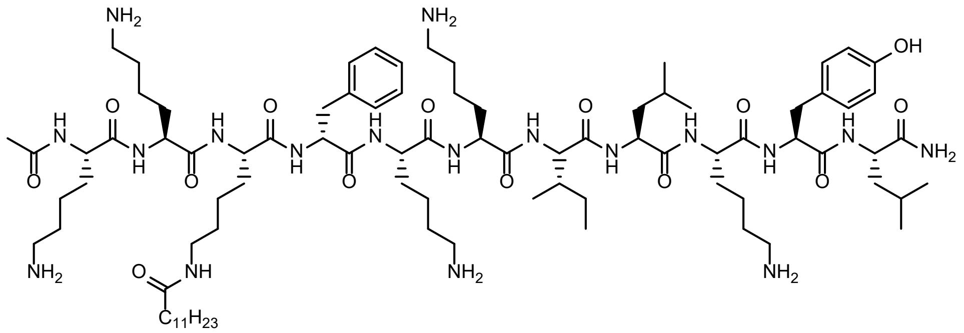

HPLC of crude peptide (λ=220 nm)

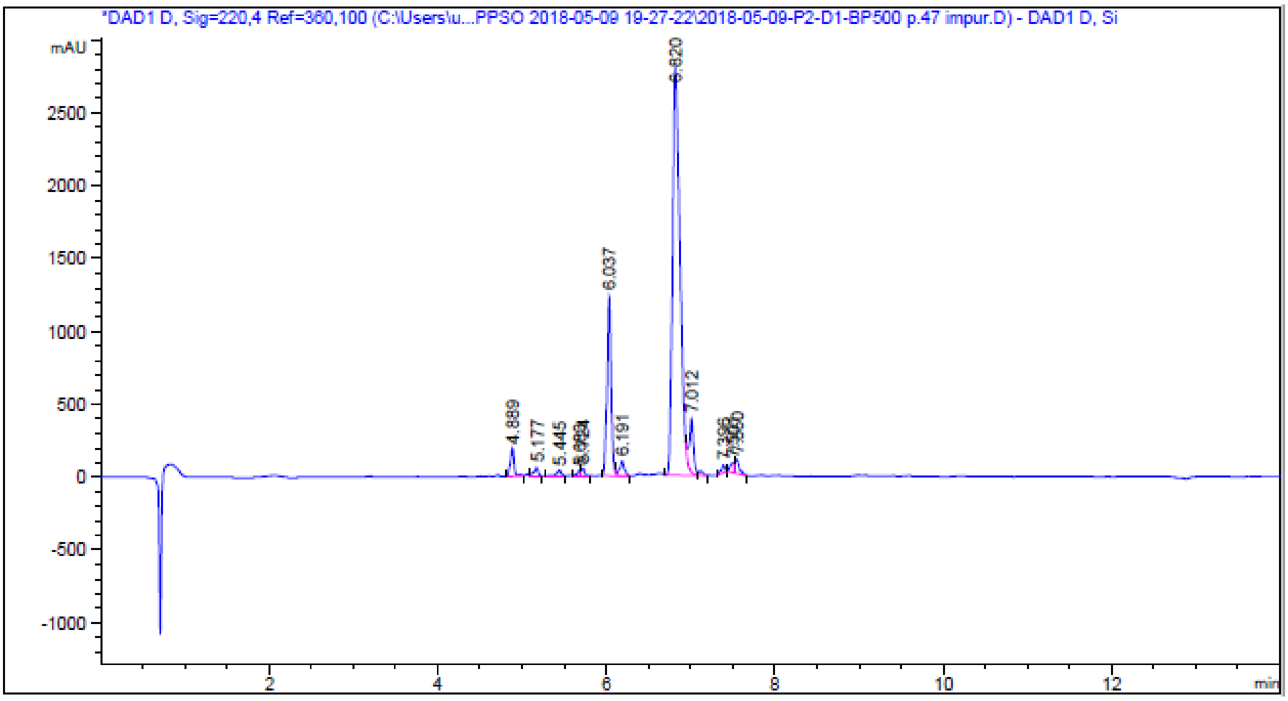

Peak #	RetTime [min]	Type	Width [min]	Area [mAU*s]	Height [mAU]	Area %
1	4.889	BV R	0.0428	573.98651	192.62531	2.2259
2	5.177	VB	0.0478	194.40025	56.93281	0.7539
3	5.445	VB R	0.0522	155.32494	41.03360	0.6023
4	5.683	BV	0.0402	88.94859	32.20647	0.3449
5	5.724	VB	0.0343	124.03711	52.92344	0.4810
6	6.037	BV R	0.0531	4459.11426	1236.56287	17.2920
7	6.191	VB E	0.0551	397.45981	102.88657	1.5413
8	6.820	BV R	0.0960	1.77881e4	2804.05859	68.9804
9	7.012	VV E	0.0503	1283.47070	371.63638	4.9772
10	7.396	BB	0.0442	159.22202	52.83167	0.6174
11	7.501	BV	0.0502	202.72284	65.27905	0.7861
12	7.550	VB	0.0532	360.41031	97.30477	1.3976

Totals : 2.57872e4 5106.28153

HPLC of purified peptide (λ=220 nm)

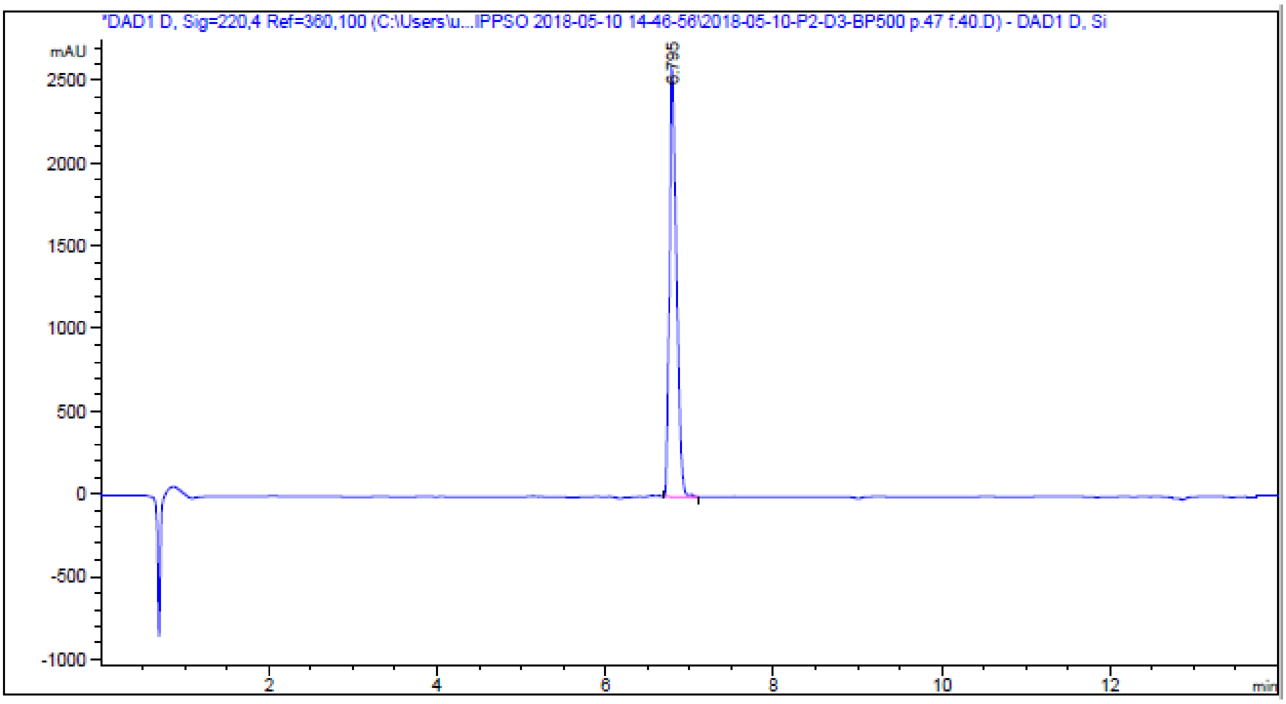

Peak #	RetTime [min]	Type	Width [min]	Area [mAU*s]	Height [mAU]	Area %
1	6.795	BV R	0.0854	1.51681e4	2586.43701	100.0000

Totals : 1.51681e4 2586.43701

ESI-MS (*m/z*)

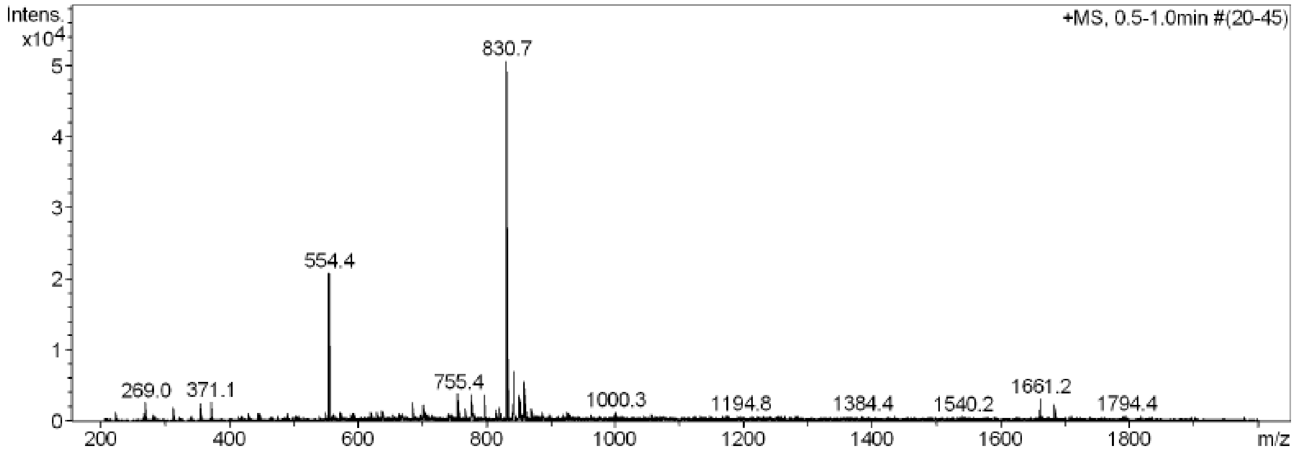

HRMS (*m/z*)

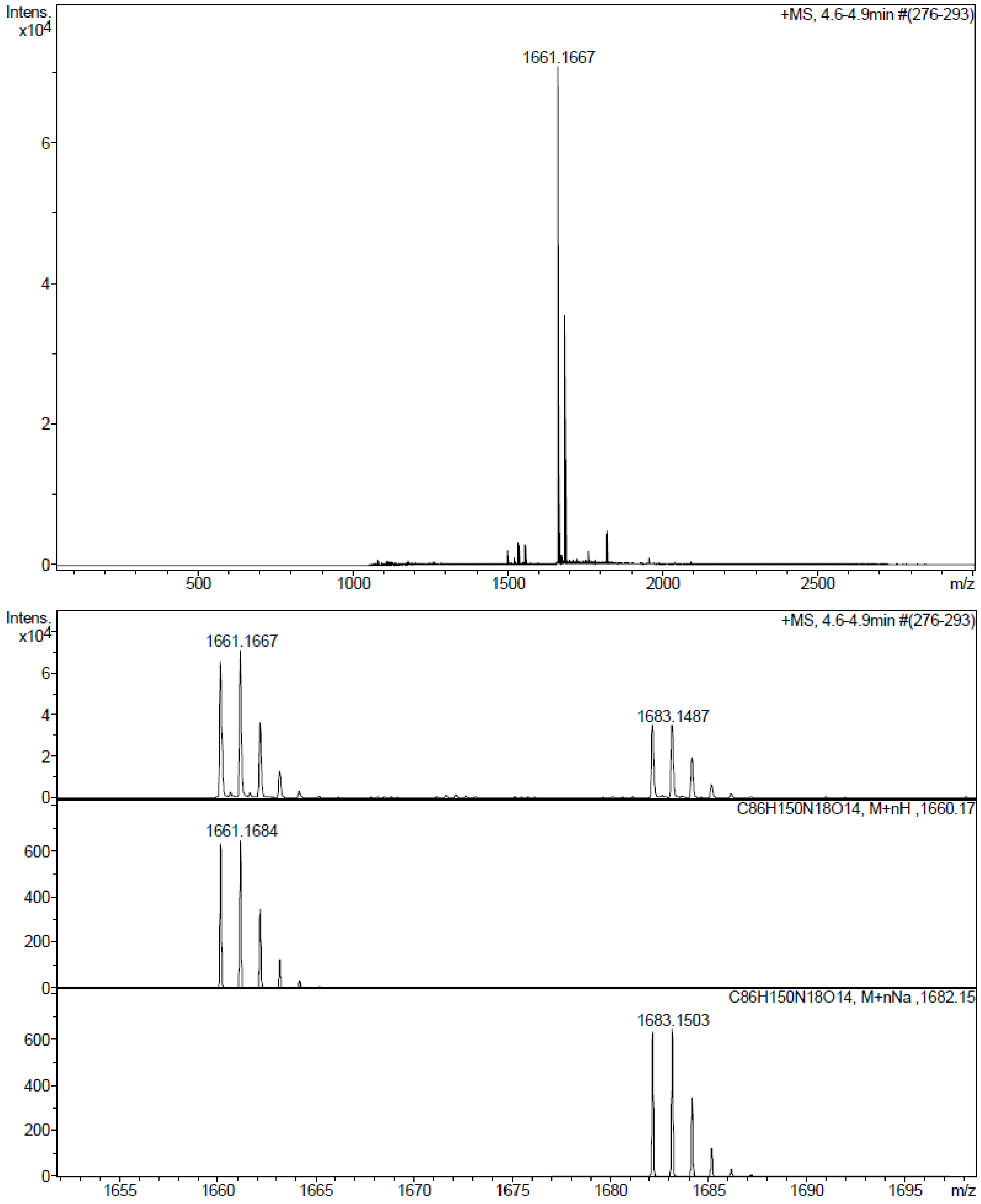

Intens.
x10^4
+MS, 4.6-4.9min #(276-293)
1661.1667
500
1000
1500
2000
2500
m/z
Intens.
x10^4
+MS, 4.6-4.9min #(276-293)
1661.1667
1683.1487
C86H150N18O14, M+nH ,1660.17
1661.1684
C86H150N18O14, M+nNa ,1682.15
1683.1503
1655
1660
1665
1670
1675
1680
1685
1690
1695
m/z

4. NMR experiments of lipopeptides BP389 and BP475

BP389

^{1}H-NMR (400 MHz, 20 mM phosphate buffer pH = 6.5 H_2O/D_2O (90:10)) *δ* (ppm): 0.77-0.89 (m, 29H, CH_3-butanoyl, δ-CH_3-Ile, 6 × δ-CH_3-Leu, γ-CH_3-Ile, δ-CH_2-Lys), 1.22-1.81 (m, 48 H, β-CH_2-butanoyl, β-CH_2-Ile, γ-CH_2-Ile, 3 × β-CH_2-Leu, 3 × γ-CH-Leu, 6 × β-CH_2-Lys, 6 × γ-CH_2-Lys, 5 × δ-CH_2-Lys), 1.97 (s, 3H, CH_3CO), 2.14 (t, *J* = 7.57 Hz, 2H, α-CH_2-butanoyl), 2.88-3.02 (m, 12H, 5 × ε-CH_2-Lys, β-CH_2-Phe), 3.09-3.16 (m, 2H, ε-CH_2-Lys^{10}), 4.03-4.07 (m, 1H, α-CH-Ile), 4.12-4.33 (m, 5H, 5 x α-CH-Lys, α-CH-Lys$^{10.}$ 3 × α-CH-Leu), 4.52-4.55 (m, α-CH-Phe), 7.09 (s, 1H, $CONH_2$), 7.18 (d, *J* = 6.96 Hz, 2H, 2 × *o*-CH-Phe_{arom}), 7.22-7.31 (m, 3H, 2 × *m*-CH-Phe_{arom}, *p*-CH-Phe_{arom}), 7.58 (s, 1H, $CONH_2$), 8.02 (t, *J* = 5.94 Hz, 1H, ζ-NH-Lys^{10}), 8.16-8.22 (m, 3H, α-NH-Phe, α-NH-Leu, α-NH-Lys), 8.29-8.36 (m, 6H, 4 × α-NH-Lys, α-NH-Ile, α-NH-Leu) 8.40-8.42 (m, 2H, α-NH-Lys^{10}, α-NH-Leu).

^{1}H-NMR (400 MHz, 20 mM phosphate buffer at pH = 6.5 with H_2O/D_2O (90:10) containing 30% CF_3CD_2OD) δ (ppm): 0.69-0.72 (m, 6H, 2 × δ-CH_3-Leu^8), 0.77-0.92 (m, 18H, 2 × δ-CH_3-Leu^{11}, δ-CH_3-Ile^7, δ-CH_3-Leu^3, CH_3-butanoyl, δ'-CH_3-Leu^3), 1.03-1.11 (m, 3H, γ-CH_3-Ile^7), 1.35-1.98 (m, 48H, γ-CH-Leu^8, δ-CH_2-Lys^1, δ-CH_2-Lys^2, δ-CH_2-Lys^5, δ-CH_2-Lys^6, δ-CH_2-Lys^9, δ-CH_2-Lys^{10}, γ-CH-Leu^{11}, β-CH_2-butanoyl, γ-CH-Leu^3, γ-CH_2-Lys^1, γ-CH_2-Lys^2, γ-CH_2-Lys^5, γ-CH_2-Lys^6, γ-CH_2-Lys^9, γ-CH_2-Lys^{10}, β-CH-Leu^8, γ-CH_2-Ile^7, β-CH-Leu^3, β-CH_2-Lys^1, β-CH_2-Lys^2, β-CH_2-Lys^5, β-CH_2-Lys^6, β-CH_2-Lys^9, β-CH_2-Lys^{10}, β-CH_2-Leu^{11}, β-CH-Ile^7), 2.03 (s, 3H, CH_3CO), 2.13 (t, *J* = 7.48 Hz, 2H, α-CH_2-butanoyl), 2.91-3.00 (m, 10 H, ε-CH_2-Lys^1, ε-CH_2-Lys^2, ε-CH_2-Lys^5, ε-CH_2-Lys^6, ε-CH_2-Lys^9), 3.08-3.16 (m, 4 H, ε-CH_2-Lys^{10}, β-CH_2-Phe^4), 3.59 (br, 1H, α-CH-Ile^7), 3.84-4.24 (m, 10H, α-CH-Lys^5, α-CH-Leu^8, α-CH-Lys^6, α-CH-Lys^1, α-CH-Lys^2, α-CH-Lys^9, α-CH-Lys^{10}, α-CH-Leu^{11}, α-CH-Leu^3, α-CH-Phe^4), 6.96 (s, 1H, $CONH_2$), 7.10 (d, *J* = 7.01 Hz, 2H, 2 × *o*-CH-Phe_{arom}), 7.16-7.21 (m, 4 H, *p*-CH-Phe_{arom}, 2 × *m*-CH-Phe_{arom}, $CONH_2$), 7.57 (br, 1H, α-NH-Leu^3), 7.59 (br, 1H, α-NH-Lys^6), 7.73 (br, 1H, α-NH-Lys^9), 7.83 (br, 1H, α-NH-Lys^{10}), 8.07-8.10 (m, 3H, α-NH-Leu^{11}, α-NH-Ile^7, α-NH-Phe^4), 8.13 (br, 1H, α-NH-Lys^5), 8.26 (br, 1H, α-NH-Leu^8), 8.36 (br, 1H, α-NH-Lys^1), 8.42 (br, 1H, α-NH-Lys^2).

BP475

^{1}H-NMR (400 MHz, 20 mM phosphate buffer pH = 6.5 H_2O/D_2O (90:10)) *δ* (ppm): 0.75-0.90 (m, 24H, CH_3-butanoyl, δ-CH_3-Ile, 6 × δ-CH_3-Leu), 0.99-1.16 (m, 5H, γ-CH_3-Ile, δ-CH_2-Lys), 1.25-1.82 (m, 48 H, β-CH_2-butanoyl, β-CH_2-Ile, γ-CH_2-Ile, 3 × β-CH_2-Leu, 3 × γ-CH-Leu, 6 × β-CH_2-Lys, 6 × γ-CH_2-Lys, 5 × δ-CH_2-Lys), 1.98 (s, 3H, CH_3CO), 2.15 (t, *J* = 7.48 Hz, 2H, α-CH_2-butanoyl), 2.85-3.00 (m, 11H, 5 × ε-CH_2-Lys, β-CH_2-Phe), 3.06-3.16 (m, 3H, ε-CH_2-$Lys^{10.}$ β-CH_2-Phe), 4.06-4.13 (m, 2H, α-CH-Ile, α-CH-Lys), 4.15-4.34 (m, 5H, 4 x α-CH-Lys, α-CH-$Lys^{10.}$ 3 × α-CH-Leu), 4.54-4.57 (m, α-CH-Phe), 7.11 (s, 1H, $CONH_2$), 7.21 (d, *J* = 7.16 Hz, 2H, 2 × *o*-CH-Phe_{arom}), 7.26-7.36 (m, 3H, 2 × *m*-CH-Phe_{arom}, *p*-CH-Phe_{arom}), 7.59 (s, 1H, $CONH_2$), 8.04 (t, *J* = 5.82Hz, 1H, ζ-NH-Lys^{10}), 8.21-8.29 (m, 3H, α-NH-Ile, α-NH-Leu, α-NH-Lys), 8.32-8.46 (m, 6H, α-NH-Lys^{10}, 4 × α-NH-Lys, α-NH-Phe, 2 × α-NH-Leu).

^{1}H-NMR (400 MHz, 20 mM phosphate buffer at pH = 6.5 with H_2O/D_2O (90:10) containing 30% CF_3CD_2OD)) *δ* (ppm): 0.82-0.94 (m, 24H, CH_3-butanoyl, 2 × δ-CH_3-Leu^3, 2 × δ-CH_3-Leu^8, 2 × δ-CH_3-Leu^{11}, δ-CH_3-Ile), 1.08-1.31 (m, 3H, γ-CH_3-Ile), 1.33-1.85 (m, 24H, β-CH_2-butanoyl, β-CH_2-Lys^6, β-CH_2-Leu^8, β-CH_2-Leu^{11}, δ-CH_2-Lys^1, δ-CH_2-Lys^2, δ-CH_2-Lys^6, δ-CH_2-Lys^{10}, γ-CH_2-Lys^1, γ-CH_2-Lys^2, γ-CH_2-Lys^6, γ-CH_2-Ile), 1.95 (s, 7H, β-CH_2-Lys^2, β-CH_2-Lys^{10}, CH_3CO), 2.14 (t, *J* = 7.66 Hz, 2H, α-CH_2-butanoyl), 2.90-2.99 (m, 7 H, ε-CH_2-Lys^1, ε-CH_2-Lys^2, ε-CH_2-Lys^6, β-CH_2-Phe), 3.15 (m, 3H, ε-CH_2-Lys^{10}, β-CH_2-Phe), 3.77 (br, 1H, α-CH-Ile), 3.85-4.19 (m, 8 H, α-CH-Lys^1, α-CH-Lys^2, α-CH-Lys^6, α-CH-Lys^{10}, α-CH-Leu^3, α-CH-Leu^8, α-CH-Leu^{11}, α-CH-Phe), 6.94 (s, 1H, $CONH_2$), 7.08 (d, *J* = 6.36 Hz, 2H, 2 × *o*-CH-Phe_{arom}), 7.27-7.18 (m, 4 H, *p*-CH-Phe_{arom}, 2 × *m*-CH-Phe_{arom}, $CONH_2$), 7.46-7.43 (m, 1H, ζ-NH-Lys^{10}), 7.64 (br, 1H, α-NH-Leu^3), 7.76 (br, 1H, α-NH-Lys^{10}), 7.81 (br, 1H, α-NH-Lys^6), 7.92 (br, 1H, α-NH-Lys^9), 8.03-8.11 (m, 3H, α-NH-Lys^5, α-NH-Ile, α-NH-Leu^{11}), 8.15-8.19 (m, 2H, α-NH-Phe, α-NH-Leu^8), 8.22 (br, 1H, α-NH-Lys^1), 8.30 (br, 1H, α-NH-Lys^2).

BP389

Table S4. NMR data of lipopeptide BP389 recorded at 400 MHz in 20 mM phosphate buffer, pH = 6.5 H_2O/D_2O (90:10)

Amino acid	δ (ppm)					
	CO-N*H*	CO-*N*H	α-C*H*	α-*C*H	β-C*H*	β-*C*H
**Phe^4**	8.32	123.8	4.53	54.8	2.97 / 3.01	36.9
**Ile^7**	8.34	124.1	4.06	57.9	1.78	35.7
**Lys^{10}**	8.41	122.5	4.21	53.2	1.66	38.9
**5 × Lys**	8.30	123.0	4.19	53.1	1.61	30.5
	8.32	127.0	4.24	53.3	1.70	30.3
	8.32	121.9	4.18	53.5	1.67	30.2
	8.35	123.7	4.13	53.8	1.65	30.2
	8.17	124.0	4.20	53.6	1.46	30.3
**3 × Leu**	8.21	123.6	4.26	51.8	1.51	39.6
	8.34	124.1	4.29	52.0	1.54	39.6
	8.42	127.8	4.33	52.1	1.61	39.5

Table S5. NMR data of lipopeptide BP389 recorded at 400 MHz in 20 mM phosphate buffer, pH = 6.5 H_2O/D_2O (90:10), containing 30% CF_3CD_2OD

Amino acid	δ (ppm)					
	CO-N*H*	CO-*N*H	α-C*H*	α-*C*H	β-C*H*	β-*C*H
**Lys^1**	8.36	126.1	4.00	56.6	1.82	29.4
**Lys^2**	8.42	116.8	4.02	56.5	1.82	29.7
**Leu^3**	7.57	124.6	4.15	54.3	1.73	26.6
**Phe^4**	8.10	118.2	4.24	57.8	3.10	35.7
**Lys^5**	8.13	116.0	3.84	57.0	1.88	29.4
**Lys^6**	7.59	118.7	3.99	56.8	2.01	29.5
**Ile^7**	8.10	120.2	3.59	62.2	1.92	34.8
**Leu^8**	8.26	118.7	3.88	55.1	1.66	26.5
**Lys^9**	7.73	117.9	4.02	55.7	1.95	29.2
**Lys^{10}**	7.83	119.5	4.04	55.9	1.90	28.0
**Leu^{11}**	8.07	118.4	4.12	53.0	1.79	

BP475

Table S6. NMR data of lipopeptide BP475 recorded at 400 MHz in 20 mM phosphate buffer, pH = 6.5 H_2O/D_2O (90:10)

Amino acid	δ (ppm)					
	CO-N*H*	CO-*N*H	α-C*H*	α-*C*H	β-C*H*	β-*C*H
**Phe[4]**	8.40	122.0	4.56	55.3	3.08 / 2.98	36.8
**Ile[7]**	8.26	122.3	4.08	57.9	1.79	35.6
**Lys[10]**	8.32	122.9	4.24	53.4	1.72	30.3
**5 × Lys**	8.27	122.7	4.22	53.5	1.68	30.2
	8.34	124.0	4.17	53.8	1.72	30.3
	8.38	124.2	4.10	53.4	1.49	30.1
	8.39	122.7	4.22	53.6	1.72	38.3
	8.45	122.3	4.29	53.2	1.68	30.1
**3 × Leu**	8.22	123.5	4.25	52.2	1.39	39.8
	8.34	121.0	4.27	52.0	1.62	39.6
	8.42	127.8	4.33	52.1	1.54	39.5

Table S7. NMR data of lipopeptide BP475 recorded at 400 MHz in 20 mM phosphate buffer, pH = 6.5 H_2O/D_2O (90:10), containing 30% CF_3CD_2OD

Amino acid	δ (ppm)					
	CO-N*H*	CO-*N*H	α-C*H*	α-*C*H	β-C*H*	β-*C*H
**Lys[1]**	8.22	125.7	3.89	55.8		
**Lys[2]**	8.30	117.5	4.10	55.5	1.94	29.3
**Leu[3]**	7.64	112.0	4.13	54.2		
**Phe[4]**	8.16	116.0	4.04	55.5	2.99 / 3.15	38.9
**Lys[5]**	8.07	119.4	4.03	55.1		
**Lys[6]**	7.81	118.8	4.01	56.6	1.78	29.2
**Ile[7]**	8.09	118.7	3.77	61.4	2.08	34.6
**Leu[8]**	8.18	119.0	4.01	56.6	1.71	
**Lys[9]**	7.92	117.8	4.06	55.55		
**Lys[10]**	7.76	119.4	4.08	55.9	1.95	30.1
**Leu[11]**	8.04	118.4	4.17	53.0	1.83	

BP389

400 MHz. 20 mM phosphate buffer. pH = 6.5 H_2O/D_2O (90:10)

^{1}H-NMR

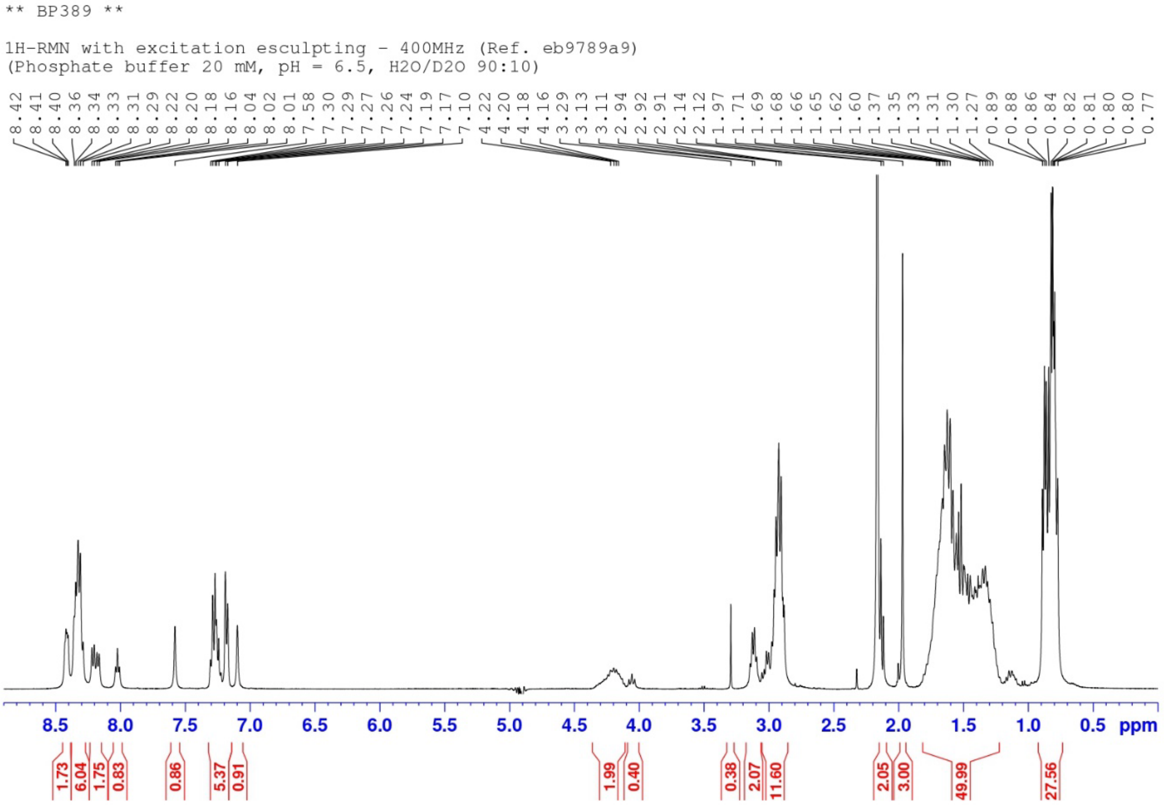

HSQC-TOCSY

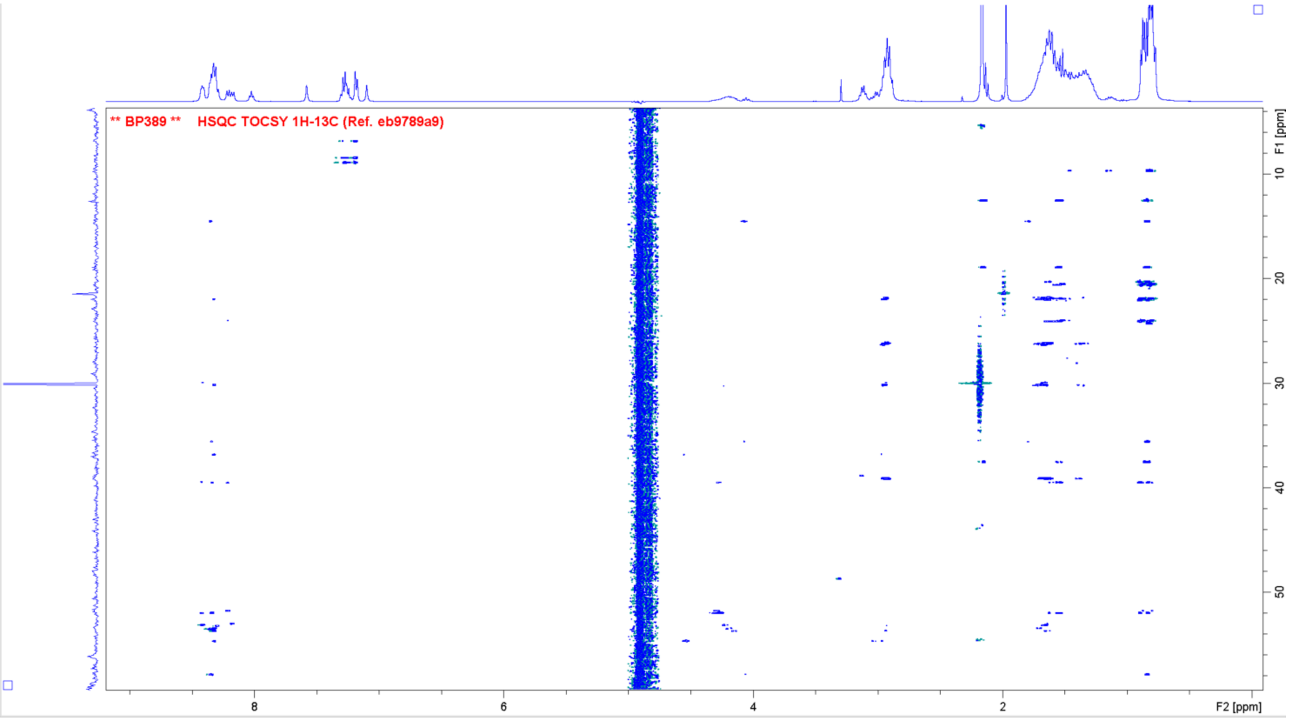

HSQC ^{1}H-^{15}N

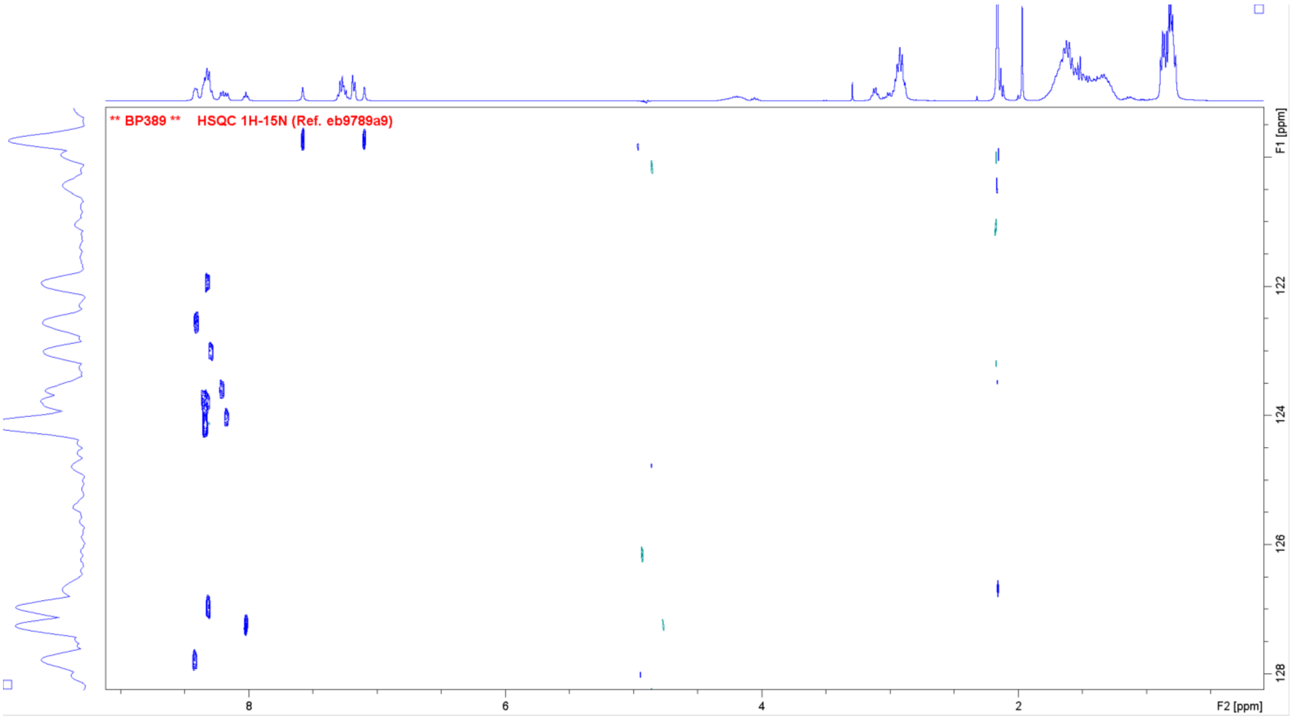

NOESY

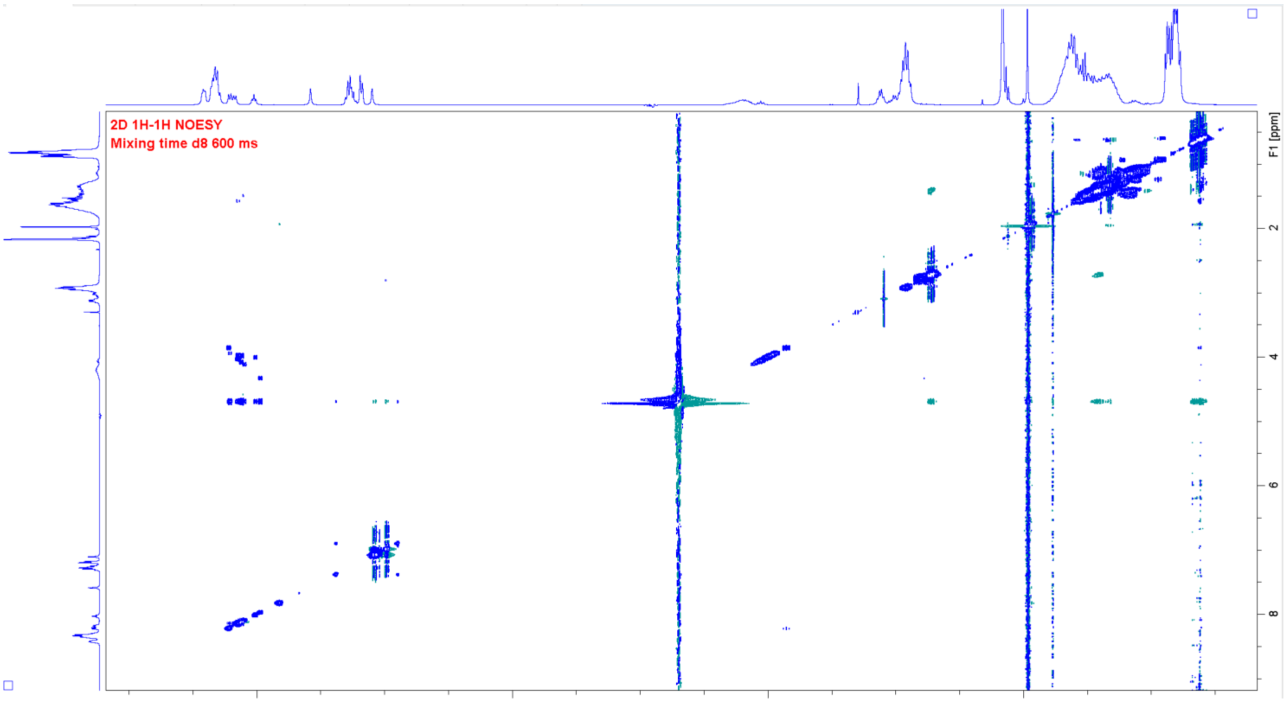

BP389

400 MHz. 20 mM phosphate buffer, pH = 6.5 H_2O/D_2O (90:10), containing 30% CF_3CD_2OD

^{1}H-NMR

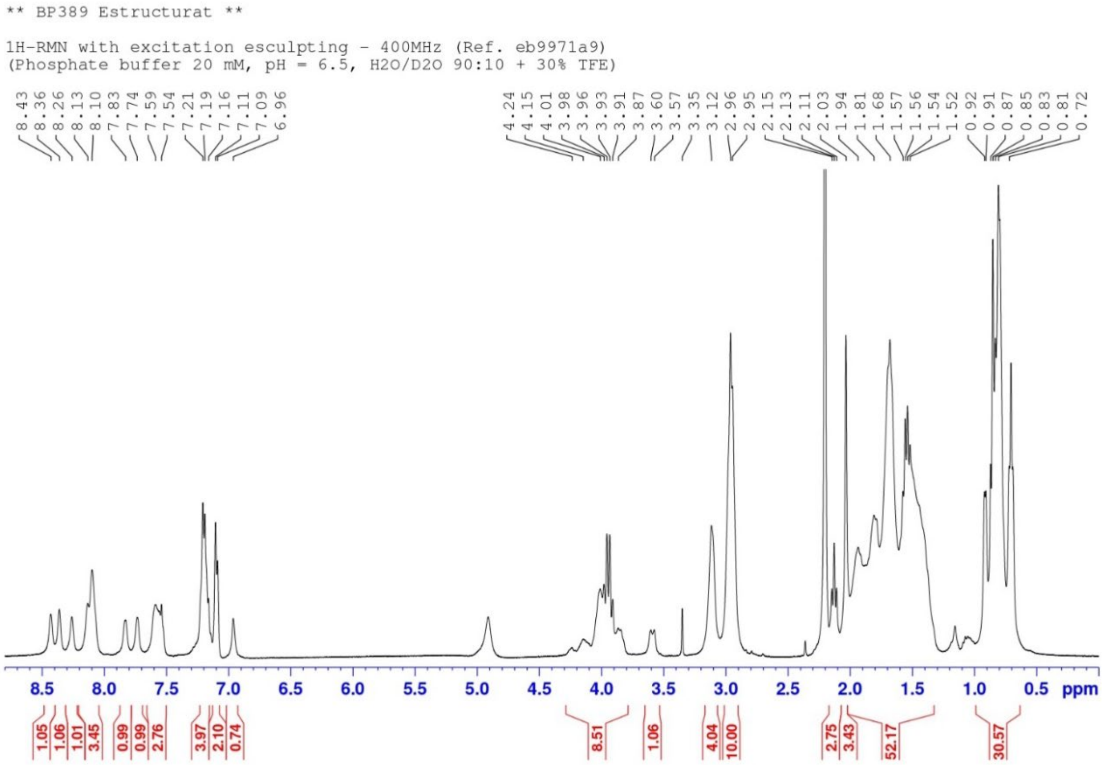

HSQC-TOCSY

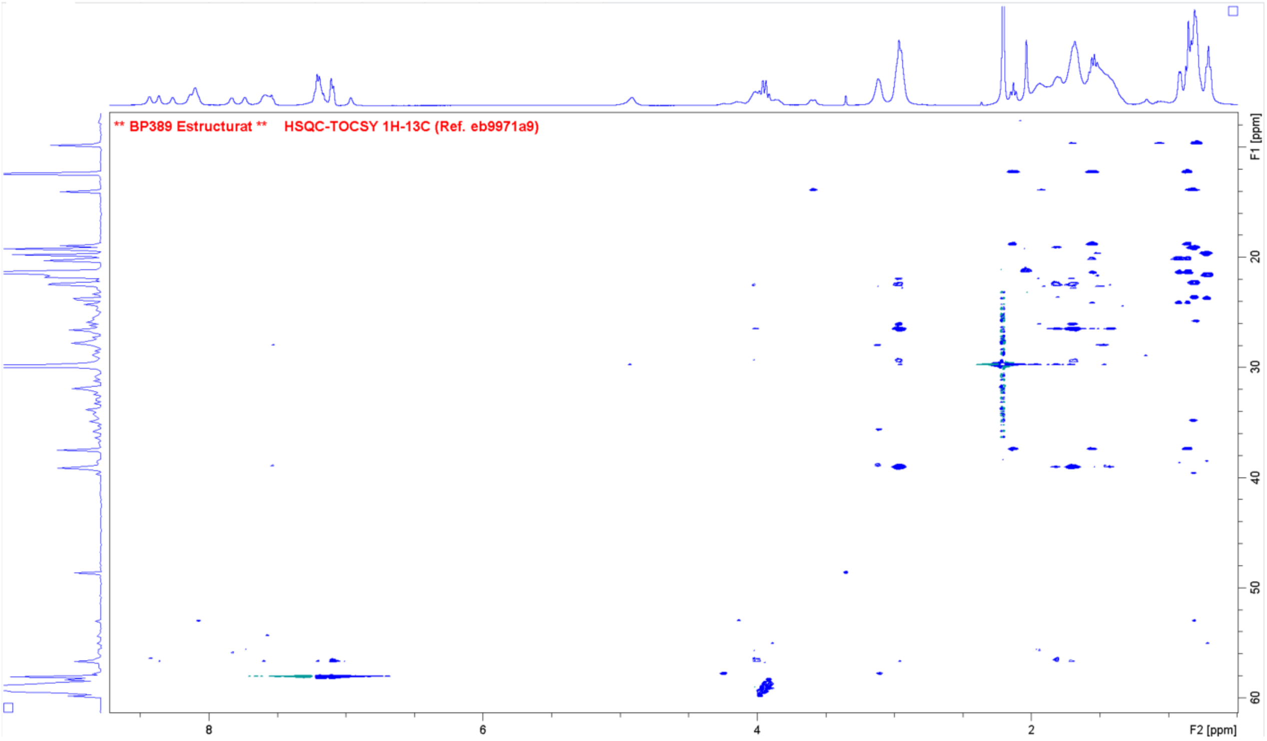

HSQC ^{1}H-^{15}N

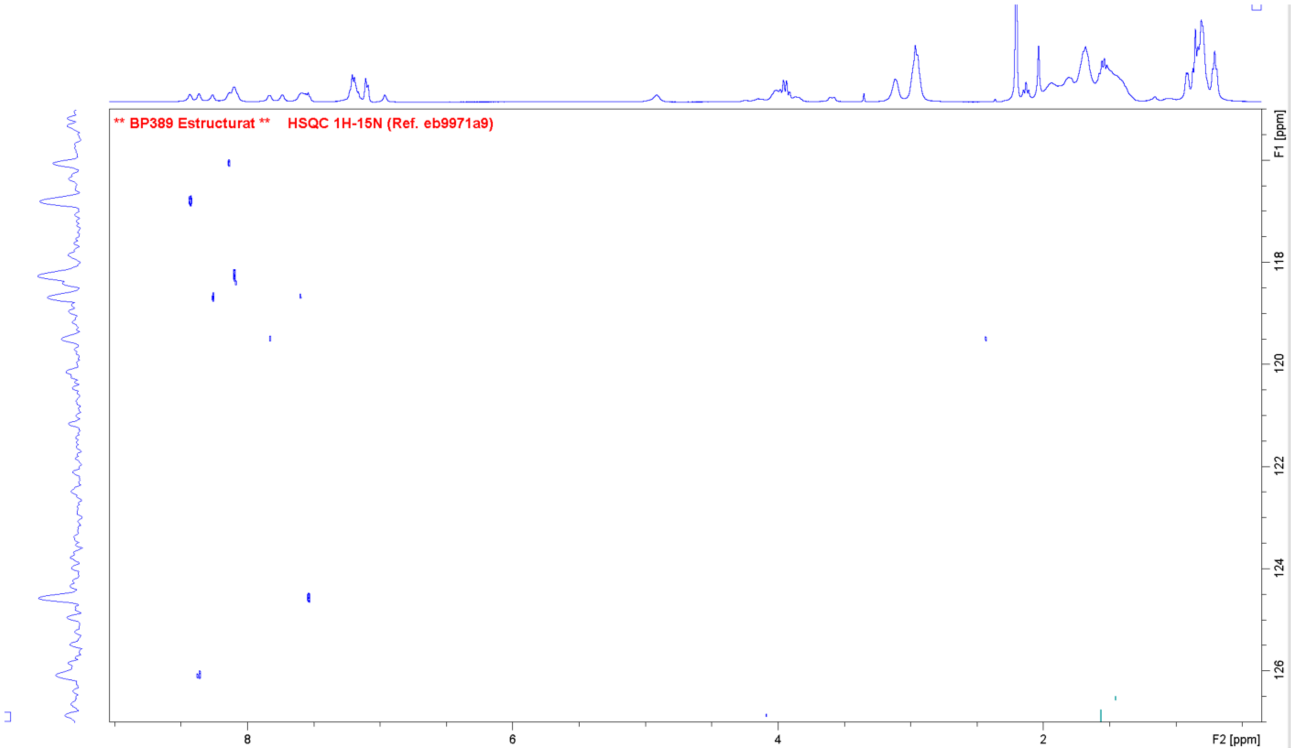

NOESY

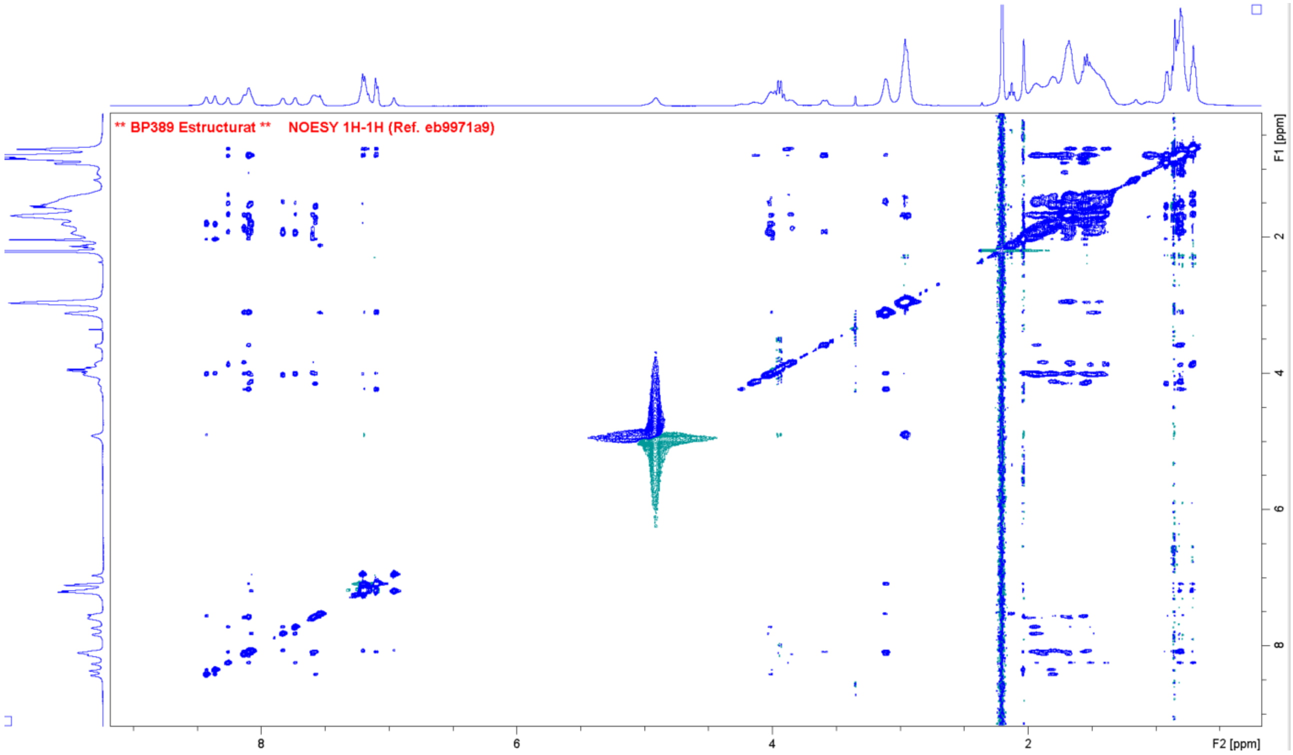

BP475

400 MHz. 20 mM phosphate buffer. pH = 6.5 H_2O/D_2O (90:10)

^{1}H-NMR

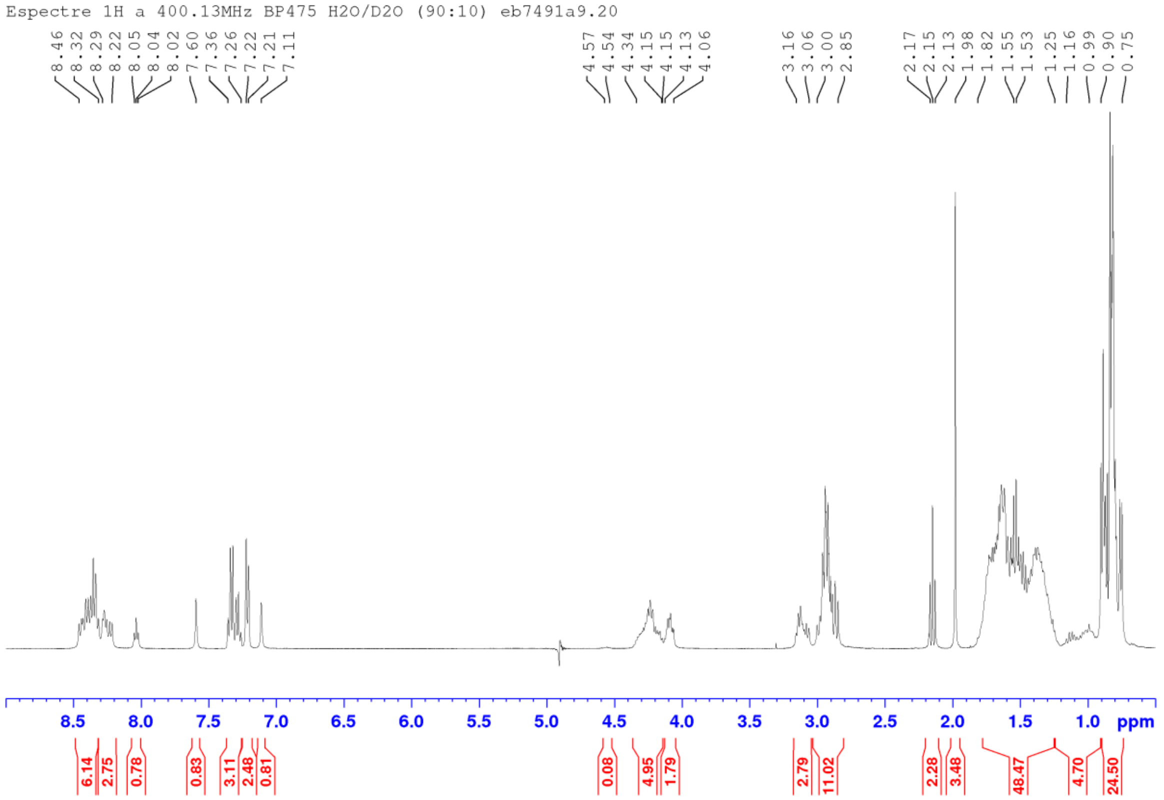

HSQC-TOCSY

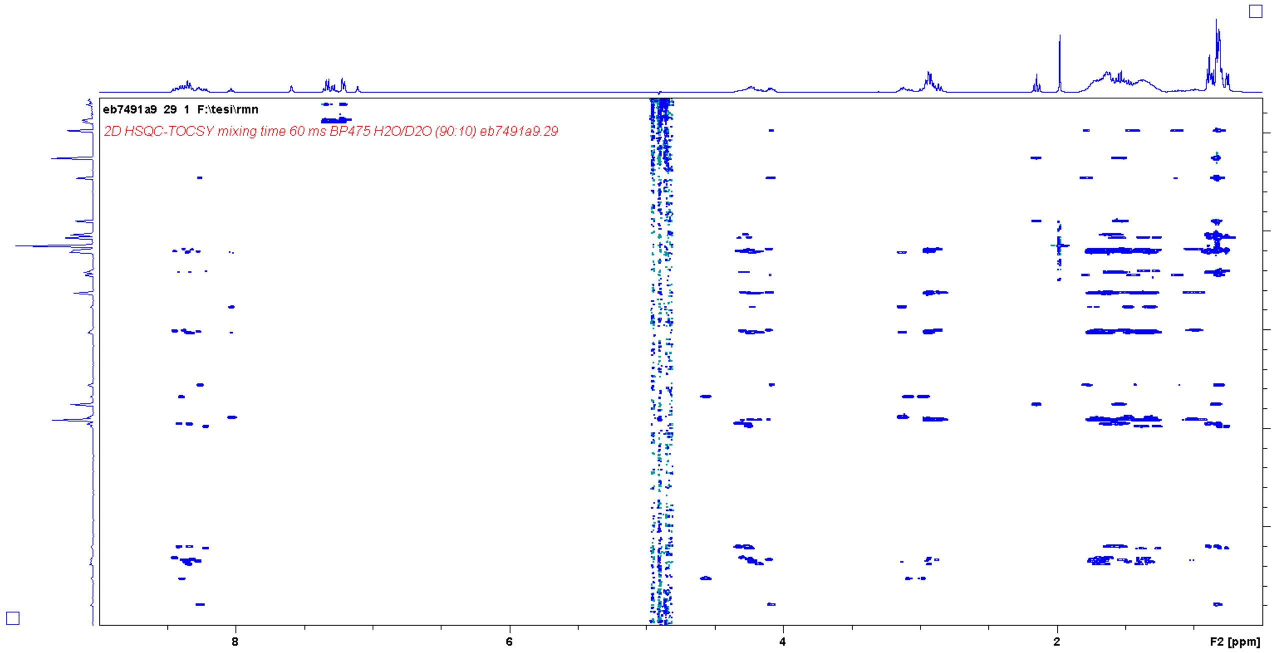

HSQC ^{1}H-^{15}N

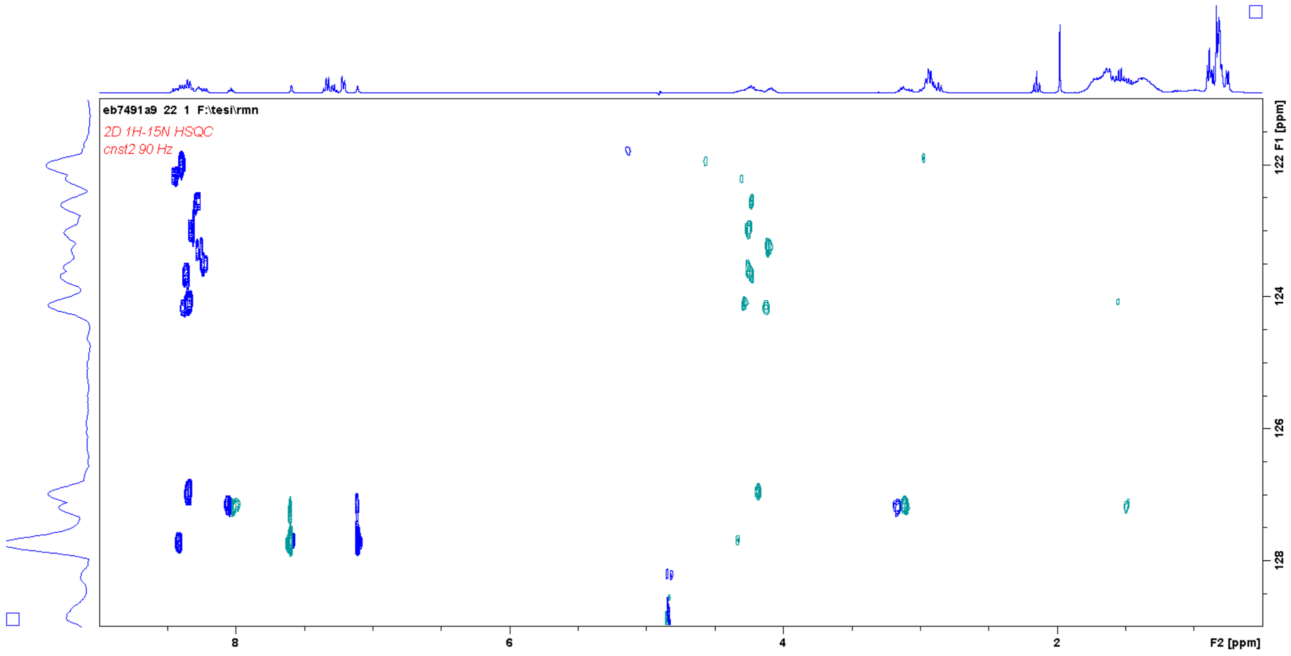

NOESY

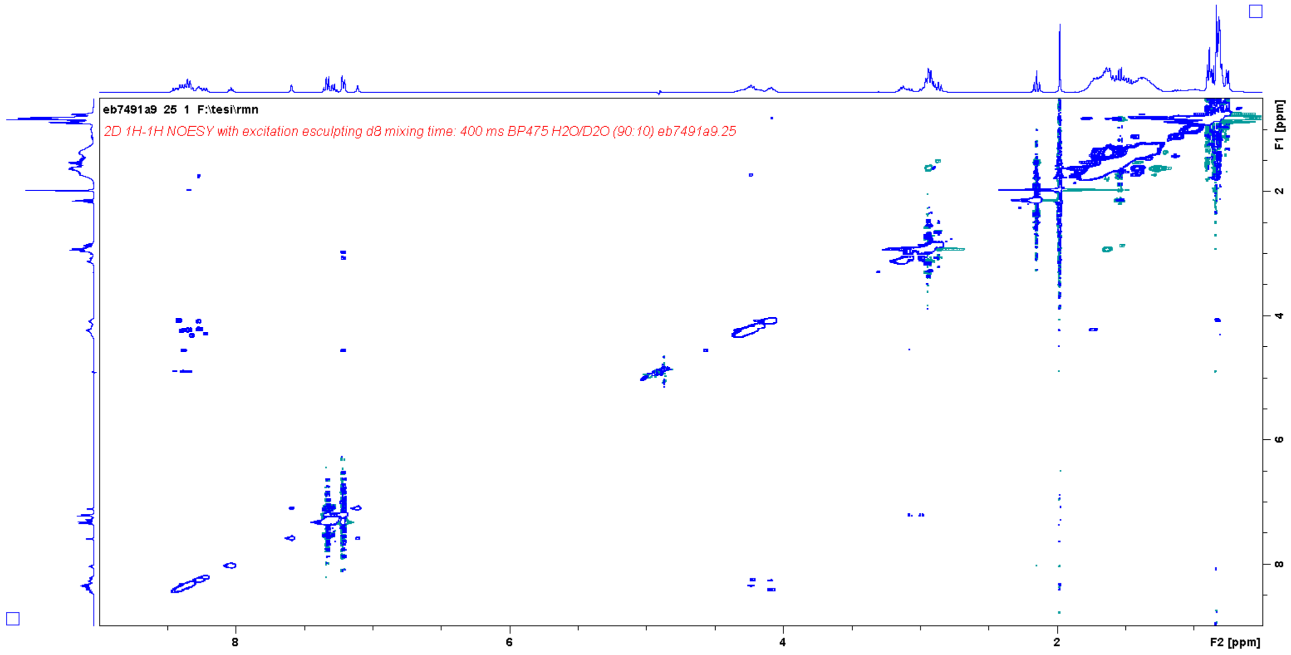

BP475

400 MHz. 20 mM phosphate buffer, pH = 6.5 H_2O/D_2O (90:10), containing 30% CF_3CD_2OD

^{1}H-NMR

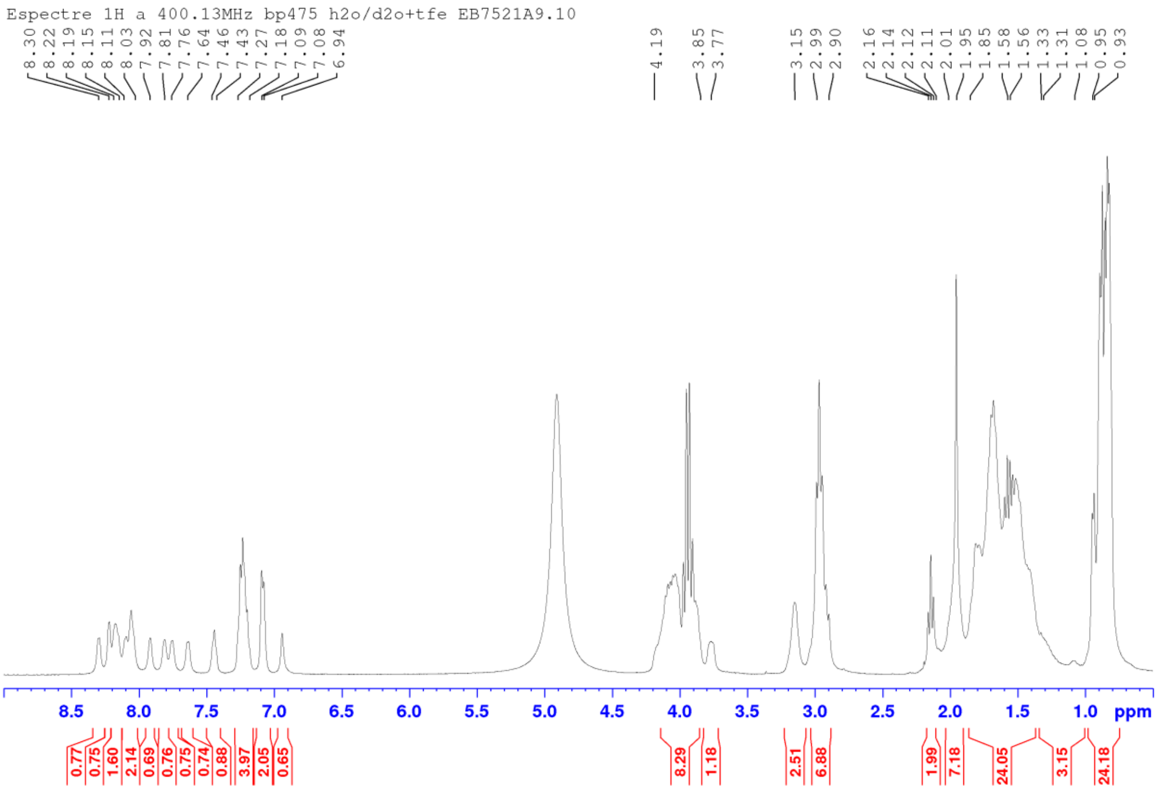

HSQC-TOCSY

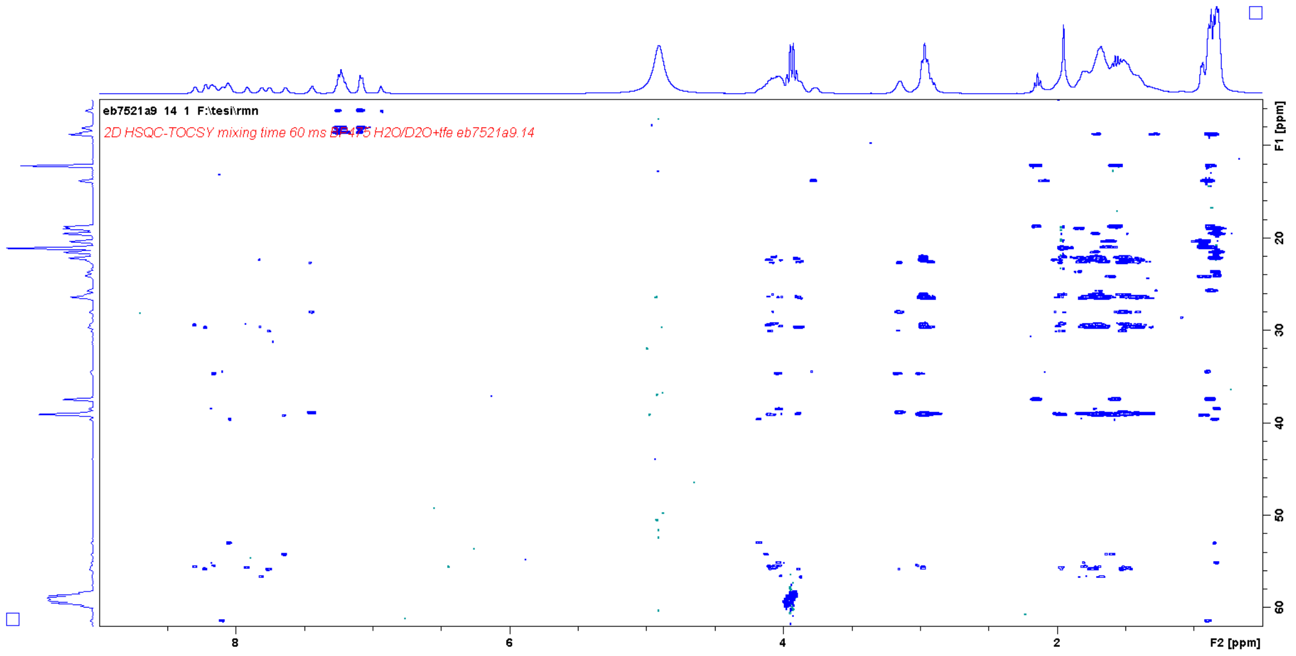

NOESY

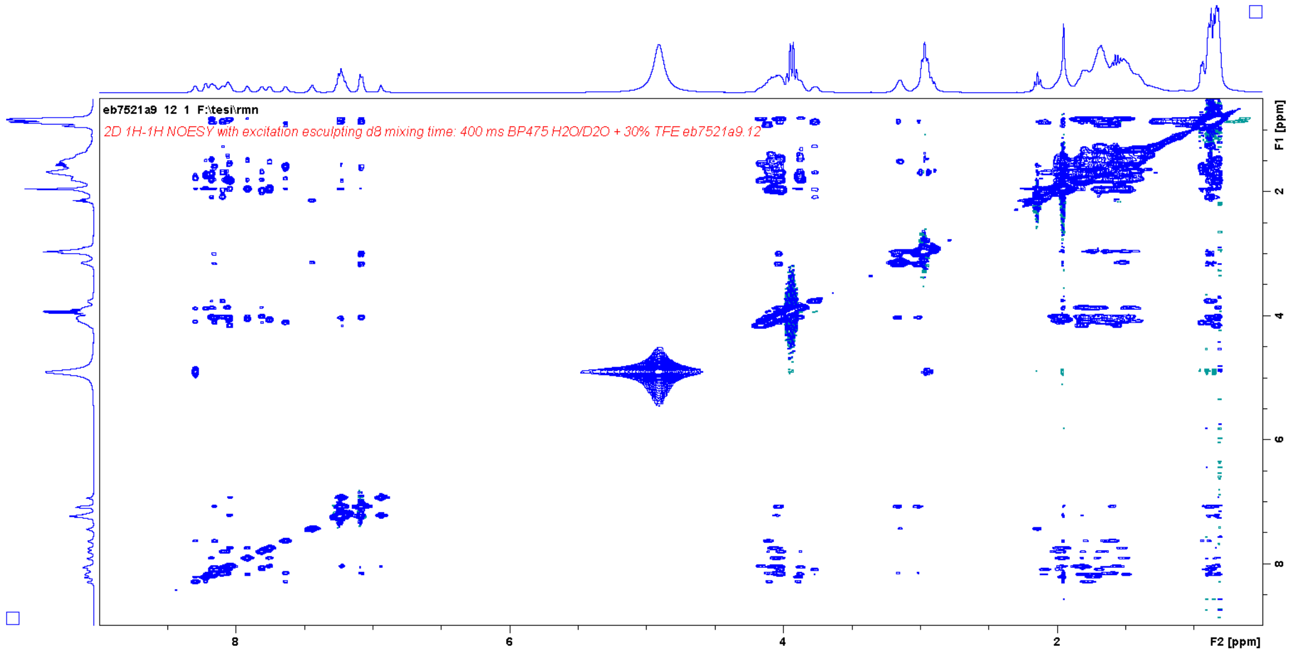

eb7521a9 12 1 F:\tesi\rmn
2D 1H-1H NOESY with excitation esculpting d8 mixing time: 400 ms BP475 H2O/D2O + 30% TFE eb7521a9.12
F1 [ppm]
F2 [ppm]
8
6
4
2
